# A metal-DNA biohybrid as enantioselective artificial photoDNAzyme

**DOI:** 10.1038/s41467-026-72881-z

**Published:** 2026-05-13

**Authors:** Zachary Pastorel, Juliette Zanzi, Mathieu Noël, Alessio Bartocci, Stellios Arseniyadis, Elise Dumont, Yves Canac, Olivier Baslé, Michael Smietana

**Affiliations:** 1 Institut des Biomolécules Max Mousseron, Université de Montpellier, CNRS, ENSCM, Montpellier, France; 2 LCC-CNRS, Université de Toulouse, CNRS, UPS, Toulouse, France; 3 Department of Medical Biotechnology and Translational Medicine, University of Milan, Milan, Italy; 4 Department of Chemistry, Queen Mary University of London, London, UK; 5 Institut de Chimie de Nice UMR 7272, Université Côte d’Azur, CNRS, Nice, France

**Keywords:** Photocatalysis, Asymmetric catalysis, Chemical bonding, Catalytic DNA, Catalyst synthesis

## Abstract

Visible light photocatalysis, which exploits the reactivity of molecules at their excited state, has triggered a paradigm shift in organic synthesis by enabling some very unique chemical transformations. Yet, achieving precise control of the enantioselectivity in such processes remains a major challenge. A promising strategy involves linking a synthetic transition metal photocatalyst within the chiral architecture of a biomolecule to create a highly selective artificial metallo-photoenzyme. However, such an artificial metalloenzyme that integrates the merits of biocatalysis and photocatalysis to promote abiological reactions with high enantioselectivity is still unknown. Here, we report an artificial metallo-photoDNAzyme resulting from the covalent anchoring of a visible light-absorbing iridium-based photocatalyst within a double-stranded (ds)DNA helix, exhibiting efficient triplet-triplet energy transfer and high levels of enantioselectivity in [2 + 2] intramolecular cycloadditions.

## Abstract (web summary)

Visible light photocatalysis enables unique excited-state reactivity but controlling enantioselectivity remains challenging. Here, the authors report an artificial metallo-photoDNAzyme, created by covalently embedding an iridium photocatalyst within double-stranded DNA, achieving efficient triplet energy transfer and highly enantioselective intramolecular [2+2] cycloadditions.

## Introduction

**Fig. 1:**
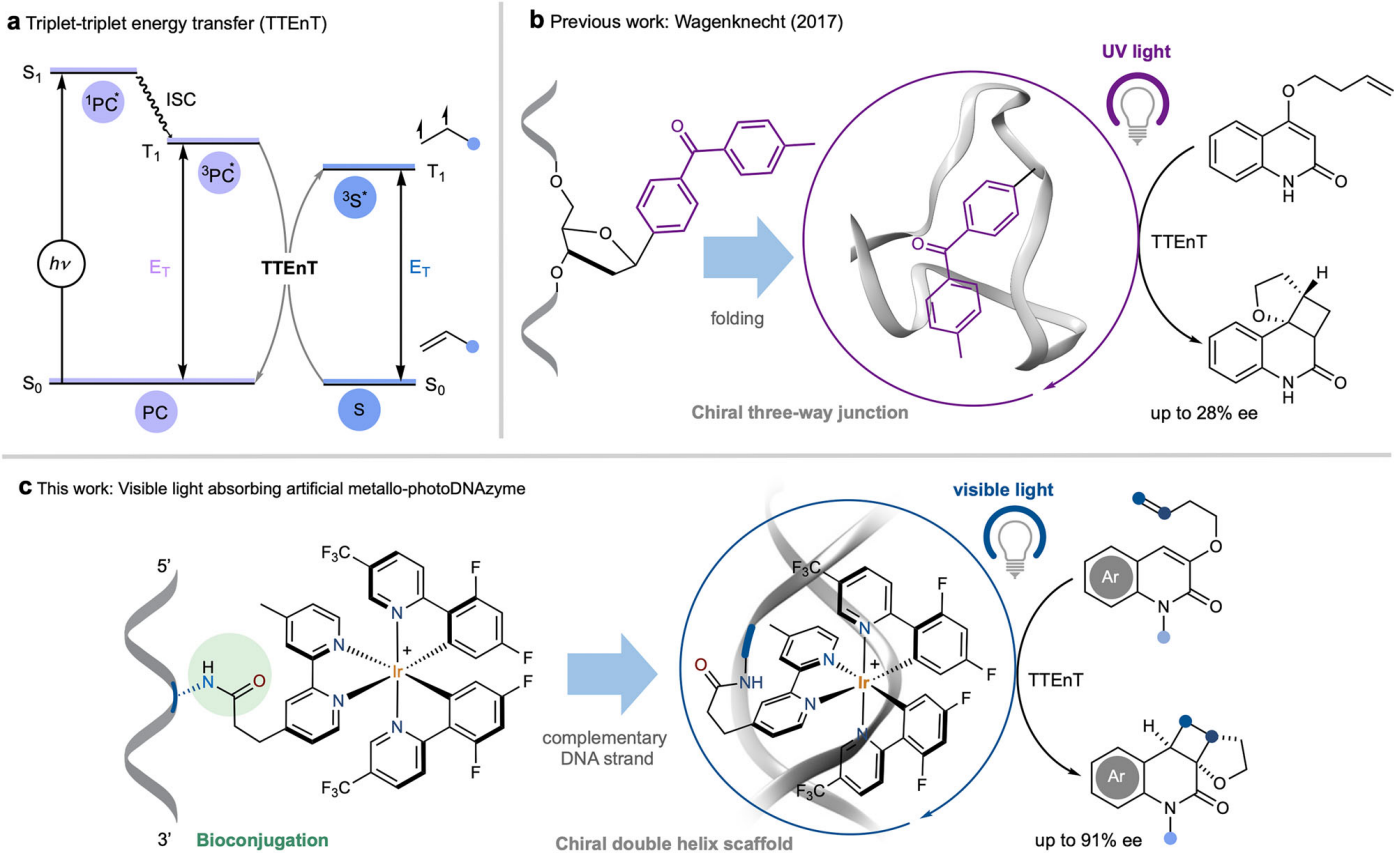
Design of artificial photoDNAzymes. **a** Schematic representation of triplet-triplet energy transfer (TTEnT) mechanism. After absorption of a photon, the photocatalyst (PC) is excited to the singlet state S_1_ and undergoes intersystem crossing (ISC) to the triplet state T_1_. Through TTEnT, the PC returns to ground state while the substate (S) reaches its reactive triplet state T_1_ (e.g., with olefin substrate). **b** The state of the art in the design of enantioselective artificial photoDNAzymes involving installation of an unnatural benzophenone photocatalyst into a chiral three-way junction to promote [2 + 2] cycloaddition by means of triplet-triplet energy transfer under UV light irradiation. **c** The approach of site-specific bioconjugation of a visible-light-absorbing iridium-based photocatalyst onto a chiral dsDNA scaffold to construct a visible light artificial metallo-photoDNAzyme and drive highly enantioselective intramolecular TTEnT [2 + 2] photocycloaddition of quinolone substrates.

The discovery of DNA’s double helix structure in the mid-twentieth century cemented its role as life’s information storage molecule^1,2^. For decades, the scientific community primarily viewed DNA as a passive repository of genetic instructions, but the latter part of that century brought a profound shift in our understanding of DNA’s capabilities. The discovery of RNA enzymes (ribozymes) in the 1980s first challenged the assumption that only proteins could serve as biological catalysts^3^. With the subsequent development of DNAzymes in 1994 by Breaker and Joyce^4^, DNA was no longer confined to its role as a genetic blueprint and emerged as a versatile molecule capable of forming complex three-dimensional structures, sensing environmental changes, and catalyzing reactions^5^. The versatility of DNA was further emphasized by the discovery of photoDNAzymes able to catalyze thymine dimer repair, thereby mirroring the activity of photolyases^6,7^. This breakthrough is all the more important because only a handful of protein-based photoenzymes are known to harness sunlight to trigger chemical reactions^8–10^. While the chemical repertoire of DNAzymes is continuously expanding^11–15^, the use of double-stranded DNA (dsDNA) as a privileged chiral scaffold in the context of asymmetric catalysis has emerged as a valuable method for producing enantioenriched compounds of interest in synthetic chemistry^16–19^. The missing link in fully exploiting DNA’s catalytic properties is now to engineer a photocatalytically active DNAzyme capable of translating the chirality information of DNA to induce high enantioselectivity in abiological transformations. Considering the ease with which modifications can be introduced into synthetic DNA, anchoring a non-natural light-absorbing cofactor within the DNA backbone to create an artificial and enantioselective photoDNAzyme appears as an attractive solution. Recently, attempts toward this approach were reported by Wagenknecht and co-workers, who described an artificial photoDNAzyme relying on a three-way junction incorporating at its branch point a benzophenone-modified nucleotide serving as photosensitizer under high-energy UV light^20^. However, the designed system, which operates by means of triplet–triplet energy transfer (Fig. 1a)^21^, was shown to promote intramolecular [2 + 2] photocycloaddition of a quinolinone derivative with low yield and enantioselectivity (Fig. 1b). These findings indicate that the unique structural and geometrical features of DNA three-way junctions, which sometimes allow specific targeting of small molecules^22^, do not provide a sufficient chiral environment to ensure a highly enantioselective transformation.

To foster the chiral microenvironment needed to induce high enantioselectivity, ideally under visible-light excitation^23^, we hypothesized that the precise covalent conjugation of an organometallic photocatalyst within a dsDNA will provide efficient triplet energy transfer in the vicinity of the chiral double helix and guide reactive photogenerated intermediates towards the required stereoselectivity outcomes. Achieving such transformations with DNA would further enhance the catalytic performance of nucleic acid-based architectures, particularly given that, despite some attempts^24–26^, no enantioselective protein-based metallo-photoenzyme has ever been reported to date^27–32^. Here, we disclose the design of an artificial photoDNAzyme operating under visible light irradiation by the site-specific incorporation of an iridium(III) photocatalyst onto a dsDNA scaffold to achieve high enantioselective control in [2 + 2] photocycloadditions operating by means of triplet–triplet energy transfer (Fig. 1c).

## Results and discussion

**Fig. 2:**
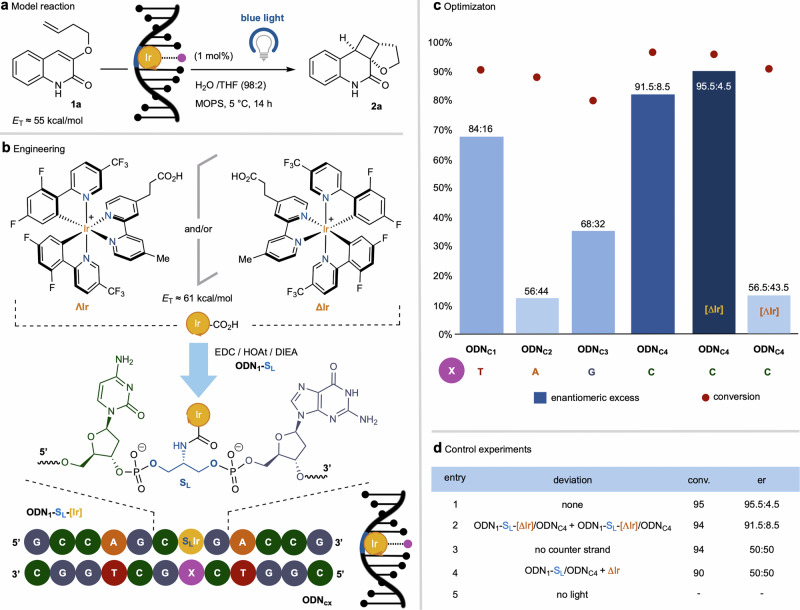
PhotoDNAzyme evolution. **a** Model reaction of intramolecular [2 + 2] cycloaddition reaction of 3-alkoxyquinolone **1a** catalyzed by iridium-based photoDNAzyme. **b** PhotoDNAzyme engineering by site-specifically incorporating a visible light absorbing iridium-based photocatalyst into the chiral environment of a dsDNA scaffold. **c** Optimization campaign by varying the nucleobase X of the complementary DNA strand and by evaluating the impact of the chirality of the covalently linked iridium photocatalyst. **d** Control experiments with changes to the optimized conditions (i.e., using ODN_1_-S_L_-[ΔIr]/ODN_C4_ as PhotoDNAzyme, entry 1).

The development of the optimal artificial photoDNAzyme was guided not only by photophysical considerations but also by a rational design of the DNA-biohybrid system. As a model reaction, we selected the intramolecular [2 + 2] photocycloaddition of 3-alkoxyquinolone **1a** whose triplet excited state energy has been estimated to be ≈55.0 kcal/mol (Fig. 2a), easily reachable through photosensitization by a visible light absorbing Ir(III) catalyst containing both the dFCF_3_-phenyl pyridine moiety and a 4,4-dialkyl bpy ligand^33^. To rapidly evolve our photoDNAzyme and enable facile covalent linking of such a transition-metal-based photocatalyst onto DNA, we synthesized the racemic [**Ir**] complex, which is based around the 4,4’-dMebpy ligand functionalized by an acetic acid moiety on one of the methyl back bond groups. The enantiopure [**ΔIr**] and [**ΛIr**] versions of the carboxylic acid-functionalized cationic complex were prepared by adapting a synthetic route developed by Meggers and co-workers (SI)^34^. These complexes were then coupled via an amide bond to a 12mer sequence (ODN_1_: 5′-GCCAGCS_L_GACCG-3′) modified at its center by a serinol (S_L_) residue (Fig. 2b)^18^.

To initiate the optimization of the reaction conditions, the intramolecular cycloaddition reaction of quinolone **1a** was first conducted in the presence of ODN_1_-S_L_-[Ir]/ODN_C1_ duplex as photoDNAzyme, which contains a thymidine facing the serinol modified by the racemic [Ir] complex. Upon visible light irradiation (*λ*_max_ = 450 nm) with blue light emitting diodes (LEDs) at 5 °C for 14 h in a 3-(morpholin-4-yl)propane-1-sulfonic acid (MOPS) buffer (pH 7.5), ODN_1_-S_L_-[Ir]/ODN_C1_ (1 mol %) catalyzed the formation of the desired cyclobutane product **2a** with a 91% conversion and a promising 84:16 enantiomeric ratio (Fig. 2c). Of note, the highest degree of enantioselectivity achieved under these conditions were obtained using a slight excess of the complementary sequence ODN_C1_ (1.5 mol%) and a high NaCl concentration (1 M) to maximize duplex formation and minimize electrostatic repulsion between the negatively charged phosphate groups along the DNA backbone (Table S5). In addition, the reaction was found to be compatible within a wide range of pH values (5.5–8.5), while increasing the amount of catalyst to 5 mol%, or lengthening ODN_1_ by two base pairs at both ends, did not improve either the conversion or the selectivity (Table S6).

We next turned our attention to the evaluation of the complementary strand and probed the influence of the nature of the residue opposite to the serinol modification bearing the iridium photocatalyst (Fig. 2c). Strikingly, purines led to a significant drop in selectivity down to 68:32 and 56:44 er for G and A, respectively. Similarly, when a simple propane-1,3-diol linker was introduced opposite to the grafted iridium photocatalyst, selectivity decreased to 69:31 er (Table S7). In contrast, the incorporation of a cytosine opposite to the iridium complex led to a remarkable increase in the enantioselectivity (up to 91.5:8.5 er), clearly showcasing the importance of this nucleobase in shaping the active site and optimally accommodating the substrate. On the other hand, keeping a cytosine opposite to the serinol modification and changing the nature of the base pairs upstream and downstream of the photocatalyst (Table S8), or pairing ODN_1_-S_L_-[Ir] with a complementary RNA strand (Table S7), had detrimental effects on the selectivity. Similarly, increasing the length of the linker connecting the photocatalyst to the serinol from an ethyl to a propyl induced a significant drop in selectivity (Table S7).

After optimizing the molecular determinants required for an efficient DNA-based enantioselective photocatalysis, we then decided to evaluate the impact of the chirality of the Ir(III) photocatalyst on the selectivity of the transformations. When paired with the optimal ODN_C4_ complementary strand, ODN_1_-S_L_-[ΔIr] and ODN_1_-S_L_-[ΛIr] sequences demonstrated large selectivity differences when catalyzing the [2 + 2] photocycloaddition (Fig. 2c). In fact, while the photoDNAzyme incorporating the lambda enantiomer [ΛIr] led to a significant drop in selectivity, the delta enantiomer enabled the achievement of an unequaled level of enantioselectivity (95.5:4.5 er). Noteworthy, a control experiment employing an equimolar ratio of the ODN_1_-S_L_-[ΔIr]/ODN_C4_ and ODN_1_-S_L_-[ΛIr]/ODN_C4_ duplex (0.5 mol% each) resulted in a selectivity identical to that observed with the racemic iridium-based ODN_1_-S_L_-[Ir]/ODN_C4_ duplex (Fig. 2d, entry 2), indicating significant kinetic differences between the two diastereomers of the photoDNAzyme (*vide infra*). Additional control experiments confirmed that both the DNA double helix structure (Fig. 2d, entry 3) and covalent grafting of the iridium photocatalyst (Fig. 2d, entry 4) were essential to achieve enantioselectivity. Finally, we also confirmed that light irradiation was mandatory for the reaction to proceed (Fig. 2d, entry 5).

**Fig. 3:**
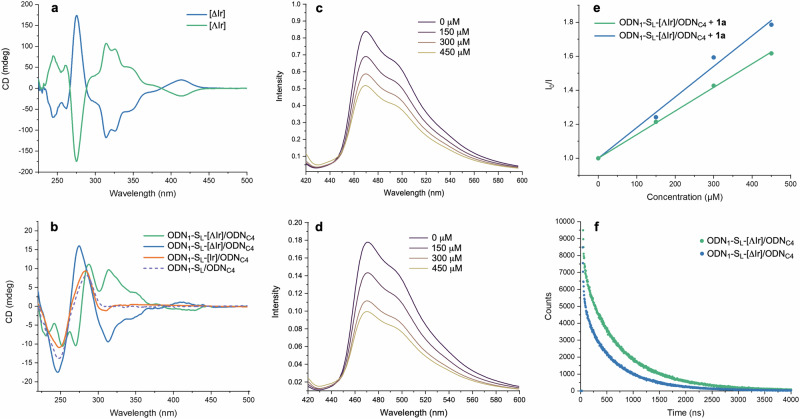
Chiroptical and photophysical properties. **a** Electronic circular dichroism (ECD) spectra of the two enantiomers [ΔIr] and [ΛIr] in CH_2_Cl_2_. **b** ECD spectra of the DNA duplex ODN_1_-S_L_/ODN_C4_ alone and linked to [Ir], [ΔIr] or [ΛIr]. **c** Luminescent quenching of ODN_1_-S_L_-[ΔIr]/ODN_C4_ in the presence of increasing concentration of substrate **1a**. **d** Luminescent quenching of ODN_1_-S_L_-[ΛIr]/ODN_C4_ in the presence of increasing concentration of substrate **1a**. **e** Stern–Volmer plots for excited-state quenching of ODN_1_-S_L_-[ΔIr]/ODN_C4_ (*K*_sv_ = 1.8 × 10^3^ M^−1^) and ODN_1_-S_L_-[ΛIr]/ODN_C4_ (*K*_sv_ = 1.4 × 10^3^ M^−1^) by **1a**. **f** Excited-state lifetime of ODN_1_-S_L_-[ΔIr]/ODN_C4_ (*τ*_o_ = 607 ns) and ODN_1_-S_L_-[ΛIr]/ODN_C4_ (*τ*_o_ = 723 ns). With *K*_sv_ = *k*_q_*τ*_o_, *k*_q_ = 2.96 × 10^9^ M^−1^ s^−1^ for ODN_1_-S_L_-[ΔIr]/ODN_C4_ and *k*_q_ = 1.90 × 10^9^ M^−1^ s^−1^ for ODN_1_-S_L_-[ΛIr]/ODN_C4_.

The observation of cooperative stereoinduction between the chiral iridium photocatalyst and nucleic acid double helix prompted us to investigate in more detail the (photo)physical properties of the diastereomeric photoDNAzymes and their implications on the selectivity of the reaction. We first examined the stability of the ODN_1_-S_L_-[ΔIr]/ODN_C4_ and ODN_1_-S_L_-[ΛIr]/ODN_C4_ duplexes by thermal denaturation. Both displayed very similar melting temperatures (*T*_m_ = 45.2 and 44.4 °C, respectively), demonstrating that this factor does not account for the differences observed in enantioselectivity (Table S3). Electronic circular dichroism (ECD) spectroscopy was then used to directly interrogate possible interactions between [ΔIr] and [ΛIr] and the DNA helix. The CD signals obtained for the Δ and Λ iridium complexes alone in dichloromethane showed expected mirror images with opposite polarization and equal ellipticity intensity (Fig. 3a). Just like the iridium-free duplex ODN_1_-S_L_/ODN_C4_, which exhibits a typical B-type CD spectrum at high energy wavelengths, the ODN_1_-S_L_-[Ir]/ODN_C4_ is spectroscopically silent above 310 nm (Fig. 3b). In contrast, the ODN_1_-S_L_-[ΔIr]/ODN_C4_ and ODN_1_-S_L_-[ΛIr]/ODN_C4_ duplexes exhibit the expected mirror image signatures of the iridium complex enantiomers with two rather strong Cotton effects centered at 315 and 415 nm. These observations align well with previous experimental results (Fig. 2d, entry 2), confirming that the high selectivity achieved by the photoDNAzyme ODN_1_-S_L_-[Ir]/ODN_C4_ incorporating the racemic [Ir] complex is not the result of a resolution of the racemic iridium complex in excess during bioconjugation, but rather a difference in kinetics between the two diastereomeric photoenzymes when catalyzing the energy transfer reaction. To gain further insight into the influence of photoDNAzyme diastereomerism on energy transfer performance, we conducted Stern–Volmer experiments of intermolecular deactivation of DNA-embedded iridium complexes' excited states by an increasing concentration of substrate **1a** as triplet quencher (Fig. 3c, d). The rate constants (*K*_sv_) measured for ODN_1_-S_L_-[ΔIr]/ODN_C4_ (1.8 × 10^3^ M^−1^) and ODN_1_-S_L_-[ΛIr]/ODN_C4_ (1.4 × 10^3^ M^−1^) (Fig. 3e), and respective excited state lifetimes (*τ*_o_) (Fig. 3f), allowed us to calculate the quenching rate coefficients (*k*_q_) that evidenced that the right-handed DNA-duplex incorporating the right-handed [ΔIr] photocatalyst is 55% more efficient than its diastereomer in transferring triplet energy to the substrate **1a**. These results support our initial hypothesis on the formation of a matched pair between the iridium photocatalyst of Δ-configuration and the right-handed DNA helix, which not only accelerates the photocycloaddition reaction but also enhances its enantiocontrol^35^.

**Fig. 4:**
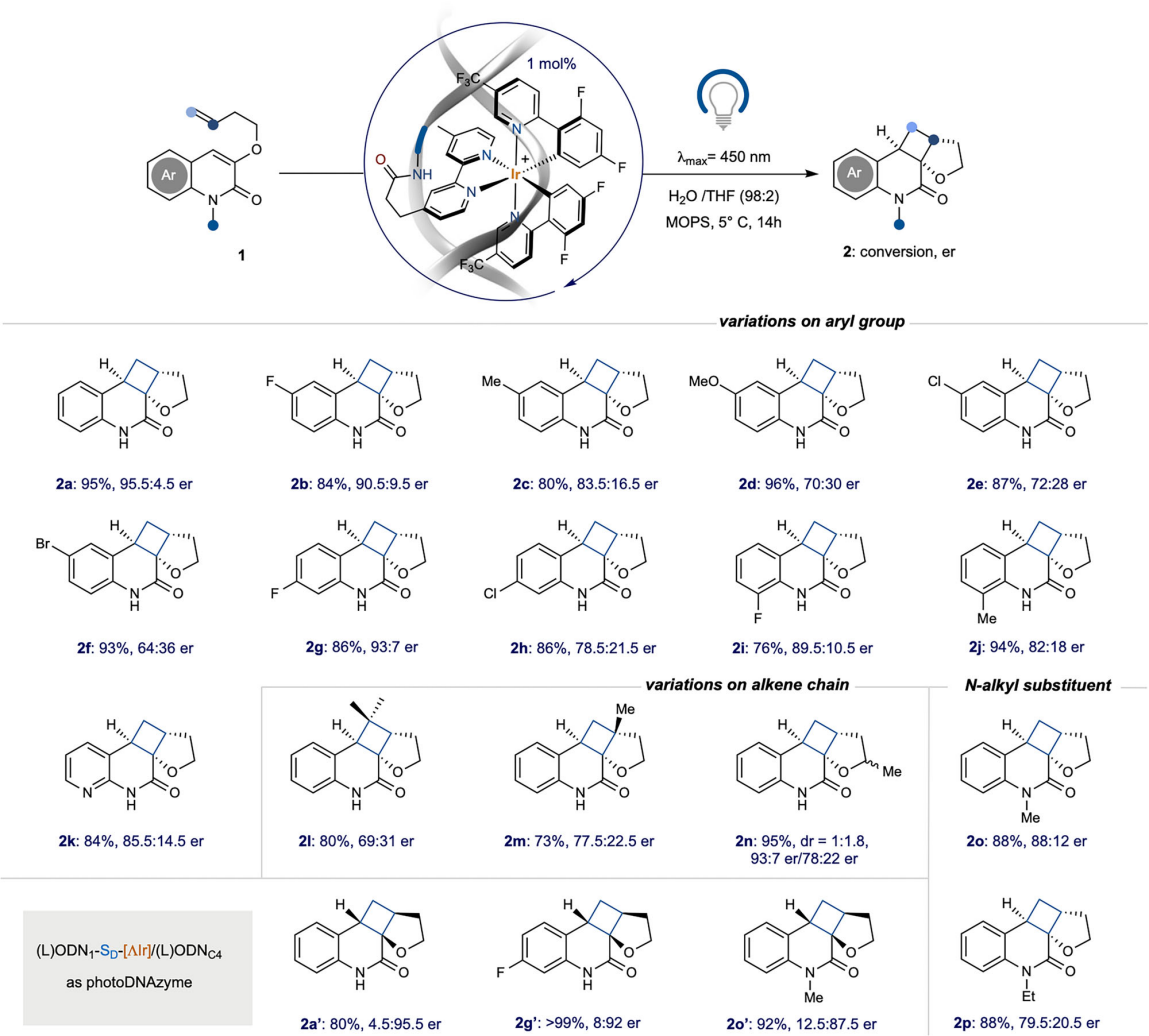
Scope of visible light TTEnT [2 + 2] cycloaddition. General reaction conditions: **1** (1 mM), ODN_1_-S_L_-[ΔIr] (1 mol%), ODNC_4_ (1.5 mol%) MOPS buffer (20 mM MOPS, 1 M NaCl pH 7.5) THF (2% v/v) stirred at 5 °C for 14 h under blue led irradiation (*λ*_max_ = 450 nm). **2a’,** **2g’** and **2o’** were provided by (L)ODN_1_-S_D_-[ΛIr] (1 mol%), (L)ODN_C4_ (1.5 mol%). The absolute stereochemistry of photoadduct **2a** was assessed by comparison with the literature of the specific optical rotation value (Supplementary Information, section SXI)^36^. The absolute stereochemistry of products **2b**–**2p**, were assigned by analogy to **2a**. The absolute stereochemistry of **2a’**, **2g’** and **2o’** was assigned by comparison of HPLC retention times with **2a,** **2g** and **2o**.

The substrate generality of our triplet photoDNAzyme was then evaluated on a range of structurally related homoallyloxy-quinolones (Fig. 4). In all cases ODN_1_-S_L_-[ΔIr]/ODN_C4_ was found to be a superior photoDNAzyme than ODN_1_-S_L_-[Ir]/ODN_C4_ (Fig. S3). The electronic effects of the substituents carried by the aromatic ring have very little influence on the photocatalytic activity, unlike steric effects. In fact, while substrate **1b** bearing a fluorine atom in position 6 is effectively converted to **2b** with a high enantioselectivity (90.5:9.5 er), the reduction in selectivity observed along the series **2c**–**2f** is consistent with increased steric constraints with the photoDNAzyme active site (*vide infra*). Similar trends were observed for **2g**, **2h,** and **2i,** **2j** with substituents at positions 7 and 8 of the quinolone substrates, respectively. Notably, the analogous 1,8-naphthyridone substrate, a potent scaffold in therapeutic and medicinal chemistry^37^, gave the desired product **2k** in high yields and with a good enantiomeric ratio. Modifications of the alkene moiety (**2l,** **2m,** **2n**) are also well tolerated, though with somewhat diminished selectivity. Cycloaddition of *N*-allyl derivatives afforded optically enriched **2o** and **2p** with good enantiomeric ratios, thus indicating that stereocontrol is not dependent on a dual hydrogen-bonding interaction as for previously reported small-molecule-based photocatalysts^33,36^. Finally, to our delight, we demonstrated that inversion of selectivity in the presence of the left-handed photoDNAzyme (L)ODN_1_-S_D_-[ΛIr]/(L)ODN_C4_ is independent of the substitution pattern of the starting quinolone, thereby enabling the production of the opposite enantiomers (**2a’,** **2g’,** **2o’**) with the same level of enantiocontrol as those obtained using its righthanded counterpart. This underscores the remarkable versatility of our approach in generating both enantiomeric forms of the products using mirror-image photoDNAzymes, a capability that remains inaccessible to protein-based photoenzymes^27–32^.

**Fig. 5:**
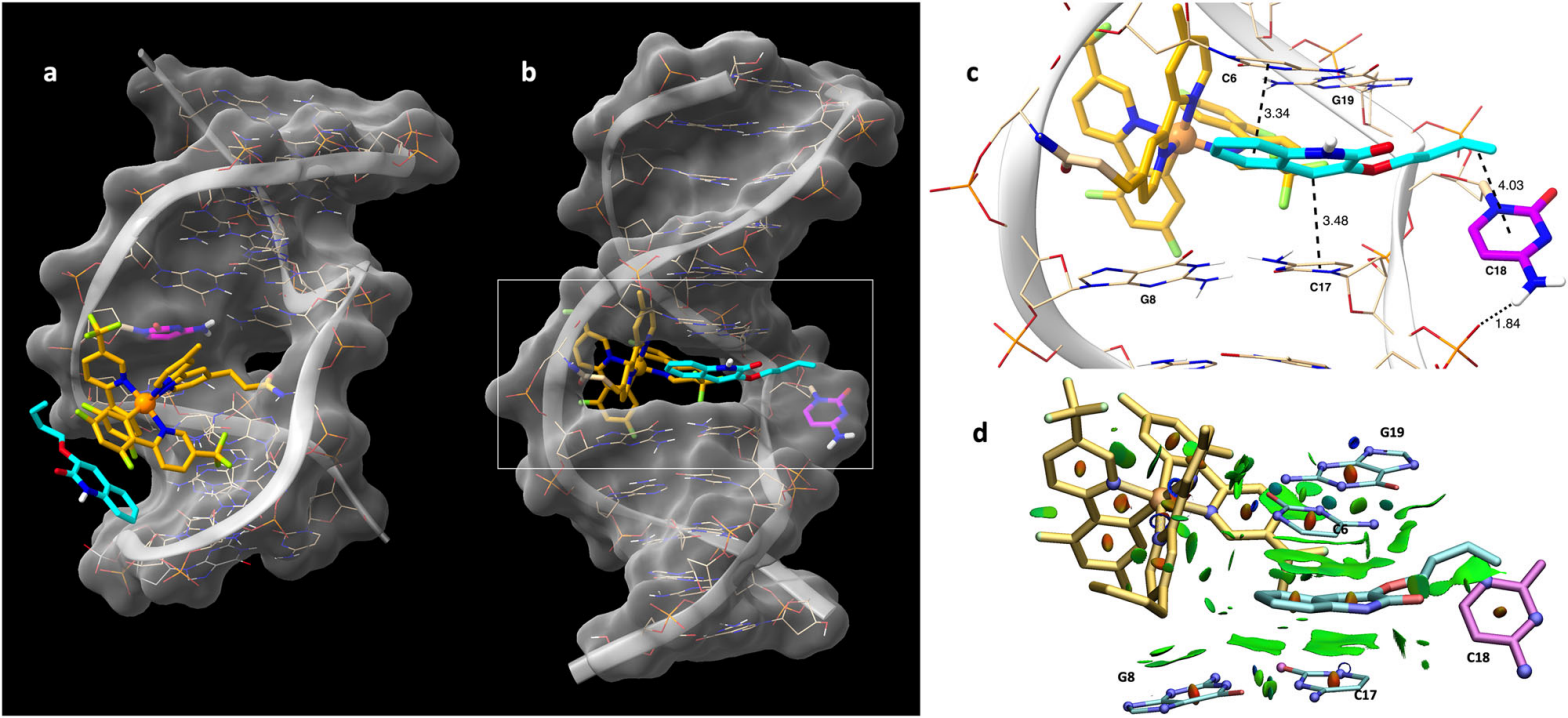
Investigations into substrate binding with molecular dynamics. **a** Full view of ODN_1_-S_L_-[ΛIr]/ODN_C4_ (ΛIr in yellow) in interaction with substrate **1a** (cyan). **b** Full view of ODN_1_-S_L_-[ΔIr]/ODN_C4_ (ΔIr in yellow) in interaction with substrate **1a** (cyan). **c** Front view of the active site of ODN_1_-S_L_-[ΔIr]/ODN_C4_ with substrate **1a** (cyan) intercalating the two adjacent GC base pairs in the major groove. π–π interactions between substrate **1a** and cytosine C17, C6 and C18 are shown as dashed lines. The extrahelical positioning of the orphan cytosine C18 (purple) is stabilized by hydrogen bonding (dotted line) with the phosphate group of C17. **d** NCI analysis for the substrate **1a** within the active-site pocket (density isosurface cut-off set up to 0.3 as the default value).

Microsecond range molecular dynamics (MD) simulations were performed to generate in silico representative structures of the two ODN_1_-S_L_-[ΔIr]/ODN_C4_ and ODN_1_-S_L_-[ΛIr]/ODN_C4_ duplexes. We first evidenced that both [ΔIr] and [ΛIr] iridium complexes preferentially adopt a minor groove binding mode with no alteration of the Watson–Crick network between adjacent base pairs along MD trajectories (Table S9), in agreement with melting temperature measurements (Table S3). We then probe the interaction of the quinolone substrate **1a** with the photoDNAzymes, and major differences were observed along these MD trajectories. While stable structures identify randomized transient π-stacking between the substrate and the iridium complex of ODN_1_-S_L_-[ΛIr]/ODN_C4_ duplex with relatively short residency time of 10–50 ns (Fig. 5a), the ODN_1_-S_L_-[ΔIr]/ODN_C4_ duplex exhibits a specific arrangement with extrahelical flipping of the orphan nucleobase C18 that defines a three dimensional hydrophobic chiral pocket where the substrate inserts rather rapidly leading to a more stable interaction mode for several hundred nanoseconds (Fig. 5b and Supplementary Movie 1). The formation of a hydrogen bond between the amino group of C18 and the 5’-phosphate group of C17 locks the extrahelical positioning along the major groove^38^, while the substrate is intercalated between the two adjacent C6 and C17 nucleobases (Fig. 5c). Noncovalent interaction (NCI) analysis evidences that **1a** is well accommodated within the active-site pocket and in close contact with the iridium complex for efficient triplet energy transfer process (Fig. 5d). This preferential substrate orientation with π–π interaction between the extrahelical C18 and the exocyclic alkene probably favors addition of the later to the *Si*-face of the quinolone moiety during the cycloaddition, leading to the observed stereochemical outcome (Figure S8).

In summary, the results presented here demonstrate how the interplay of a transition-metal photocatalyst with a DNA scaffold gives rise to a photoDNAzyme with remarkable reactivity, and that the chirality of dsDNA can be leveraged to enable asymmetric induction in visible light photocatalysis. This study pioneers an approach for designing artificial DNAzyme architectures that provide an effective enantiodifferentiating environment for phototogenerated reactive intermediates. We anticipate that this disclosure of an enantioselective artificial metallo-photoDNAzyme will stimulate further developments, leading to exciting potential applications across a wide variety of light-driven transformations.

## Methods

### General procedure for enantioselective [2 + 2] photocycloaddition

A 1 mL glass vial was charged with H_2_O (*X* µL, *V*_tot_ = 100 µL, *X* = 100–(*V*_DNAzyme_ + *V*_conter strand_ + *V*_buffer_ + *V*_NaCl_ + *V*_quinolone_)), DNAzyme (1 mol%, 1 nmol, 10 µM) and counter-strand (1.5 mol%, 1.5 nmol, 15 µM) in a MOPS buffer pH 7.5 (10 µL from a 200 mM solution in milli-Q H_2_O, *C*_f_ = 20 mM), NaCl (20 µL from a 5 M solution in milli-Q H_2_O, *C*_f_ = 1 M). The reaction mixture was briefly mixed, heated at 90 °C for 5 min and allowed to cool down at room temperature. Quinolone (2 µL from a 50 mM solution in THF, 100 nmol, *C*_f_ = 1 mM) was then added. The mixture was degassed by bubbling argon for 3 min and the vial was placed under irradiation (*λ*_max_ = 450 nm) and stirred for 14–16 h at 5 °C. The mixture was then transferred into 2 mL Eppendorf^**®**^. The reaction vial was rinsed with milli-Q H_2_O (500 µL) and diethyl ether (500 µL). The organic phase was poured into a new 2 mL Eppendorf^**®**^. The process was repeated twice (2 × 500 µL diethyl ether). After evaporation of diethyl ether, the sample was dissolved in MeOH (100 µL), transferred into HPLC mini vials, and injected in chiral HPLC to measure conversion and enantiomeric ratio.

## Supplementary information

Supplementary Information

Peer Review file

Description of Additional Supplementary Files

Supplementary Movie 1

## Data Availability

Cluster analysis summary and structures from MD simulations can be accessed in the Zenodo repository (10.5281/zenodo.15609087). All other data supporting the findings of this work are available within the article and the Supplementary Information. All data are available from the corresponding authors upon request.
