# Supplementary material for: A metal-DNA biohybrid as enantioselective artificial photoDNAzyme
Source: Nat Commun. 2026 May 13;17:7527. doi: 10.1038/s41467-026-72881-z (PMC13409001; doi:10.1038/s41467-026-72881-z)
Supplement: Supplementary file 1 — Supplementary Information [file 41467_2026_72881_MOESM1_ESM.pdf]

Supplementary Information for

A metal-DNA biohybrid as enantioselective artificial photoDNAzyme

Zachary Pastorel[1†,] Juliette Zanzi[2†], Mathieu Noël[1], Alessio Bartocci[3], Stellios Arseniyadis[4], Elise Dumont[5], Yves Canac[2*], Olivier Baslé[2*], Michael Smietana[1*]

[1] Institut des Biomolécules Max Mousseron, Université de Montpellier, CNRS, ENSCM, 34095 Montpellier, France

[2] LCC-CNRS, Université de Toulouse, CNRS, UPS, 31077 Toulouse, France.

[3] Department of Medical Biotechnology and Translational Medicine, University of Milan, Milan, Italy.

[4] Queen Mary University of London, Department of Chemistry, London E1 4NS, United Kingdom.

[5] Institut de Chimie de Nice UMR 7272, Université Côte d'Azur, CNRS, 06108 Nice, France.

[†] These authors contributed equally.

*Corresponding authors: yves.canac@lcc-toulouse.fr; olivier.basle@lcc-toulouse.fr; michael.smietana@umontpellier.fr

Table of Content

I. General information 4

II. Synthesis of iridium complexes 6

1. Synthesis of iridium dimer 6
2. Synthesis of bipyridine ligands 7
3. Synthesis of iridium racemic complexes 8
4. Synthesis of enantiopur iridium complexes 12

III. Oligonucleotide synthesis 17

1. Synthesis of serinol phosphoramidite 17
2. Oligonucleotide synthesis 20
3. Synthesis of modified oligonucleotides (Oligonucleotide – Ir complex coupling) 21
4. Characterization of oligonucleotides 22
5. Complementary circular dichroism analysis 36

IV. Synthesis of substrates 37

1. General procedure for the synthesis of substrates 37
2. Characterization of substrates 38

V. Reaction optimization 49

VI. [2+2] Photocycloaddition 52

1. General procedure for racemic [2+2] photocycloaddition 52
2. General procedure for enantioselective [2+2] photocycloaddition 52
3. Characterization of cycloaddition products 53
4. Scope of visible light TTEnT [2+2] cycloaddition. 61

VII. Molecular dynamics simulations 62

VIII. Photophysical Measurements 69

1. Excited state lifetime measurements 69
2. Stern-Volmer experiment 70

IX. Cyclic voltammetry data 71

X. Complementay mechanistic tests 72

XI. Scale-up and determination of absolute stereochemistry 72
XII. NMR data 74
1. NMR of bipyridine ligands 74
2. NMR of Iridium complexes 76
3. NMR of substrates 91
4. NMR of [2+2] photocycloaddition products 100
XIII. Chiral HPLC spectra of [2+2] photocycloaddition products 117
1. General information 117
2. (D)-DNA serie 118
3. (L)-DNA serie 134
XIV. Supplementary References 137

I. General information

All reactions were carried out under air with magnetic stirring, unless otherwise stated. Reagent grade solvents were used without purification for all extractions and work-up procedures. Water came from a Milli-Q water system from Millipore Sigma. Thin layer chromatographies (TLC) were made on silica plate 60 F254 Merck and revealed at 254 nm. Flash chromatography was carried out on silica gel 60 (40–63 µM) with various mixtures of cyclohexane/ethyl acetate or cyclohexane/acetone.

Photocatalysis was carried out in an EvoluChem (HepatoChem) photoreactor with 450 nm blue LED lamp (HCK1012-02-002). 1mL glass reaction vials were immersed in water regulated at 5°C by an external chiller connected to the reactor.

NMR analyses were acquired at 25 °C on a Bruker Avance spectrometer operating at 400 MHz as well as on 600 MHz and 500 MHz Bruker Avance III spectrometers equipped with TCI and BBO cryo-probeheads respectively at LMP Montpellier. Chemical shifts were given in parts per million (ppm, *δ*), referenced to the solvent peak of $CDCl_3$ (δ = 7.26 ppm (^{1}H NMR) and δ = 77.16 (^{13}C NMR)), or DMSO-d6 (δ = 2.50 ppm (^{1}H NMR) and δ = 39.52 (^{13}C NMR)). Coupling constants are quoted in Hz (*J*). Selected ^{13}C NMR spectra were recorded using the attached proton test (APT) to facilitate the confirmation and assignment of the structure. HRMS were recorded on a Micromass Q-TOF spectrometer with an electrospray ionization (ESI) in negative or positive mode.

Analytical high-performance liquid chromatographies (HPLC) were made on a HPLC Dionex 600 system with UV detection at 260 nm. Enantiomeric excess determinations were performed by amylose-based column chromatography analysis on chiral phase (Chiralpak IA column) with a UV/visible detector. Elution was performed using *n*-hexane (0.1 % diethylamine) / *i*-PrOH (0.1 % diethylamine): isocratic in various ratio from 60:40 to 98:2.

All the oligonucleotides' syntheses were conducted on an ABI 394 DNA/RNA synthesizer by classical phosphoramidite chemistry with commercial phosphoramidites and CPG (Control Pore Glass) solid support using standard conditions, at 1 µmol scale. DNA samples were analysed by analytic HPLC with a gradient of buffer B (TEAAc 0.05M + 80% MeCN) in buffer A (TEAAc 0.05M + 1% MeCN) at a flow of 1 mL/min. MALDI-TOF mass spectra were recorded in negative mode on an Axima assurance spectrometer (Shimadzu Biotech) using 1 µL of purified DNA sample mixed with 5 µL of a 2',4',6'-trihydroxyacetophenone monohydrate (THAP) saturated matrix and ammonium citrate (0.1 M) as co-matrix. Samples were then spotted on a stainless-steel plate and air dried before analysis. Oligonucleotides dosages were made on a Varian Cary 300 Bio UV spectrometer with a UV detection at 260 nm.

Melting temperature studies were performed on an Agilent Cary 3500 Multicell UV-Vis spectrophotometer at a concentration of 2 µM for each oligonucleotide in 1 mL milli-Q H_2O (100 mM NaCl and 20 mM MOPS buffer pH 7.5), using a 1 cm pathlength quartz UV cuvette. The melting and annealing transitions were performed twice at a slope of 0.5°C/min from 5 to 90 °C. The curves showed reversible transitions. The Tm value corresponds to the average of the temperatures at the inflection point of each transition with a standard error around 0.5°C.

Circular dichroism and fluorescence experiments were both performed on JASCO J-815 at Synbio3 platform supported by GIS IBISA. CD spectra were typically recorded with 2 µM of oligonucleotide dissolved in milli-Q H_2O (20 mM MOPS buffer pH 7.5 and 100 mM NaCl), using a 1 cm pathlength CD cuvette at 20 °C, over a wavelength range of 190-600 nm. Continuous scanning mode was used, with a response of 1.0 s with 1 nm steps and a bandwidth of 1 nm. The signal to noise ratio was improved by acquiring each spectrum over an average of three scans. Baseline was corrected by subtracting the background from the sample spectrum. Fluorescence spectra were recorded with 10 µM oligonucleotides in milli-Q H_2O using a 100 µL fluorescence cuvette at 20 °C, operated at λ_{ex} = 400 nm and scanning between 420-600 nm. Continuous scanning mode was used, with a response of 1.0 s with 1 nm steps and a bandwidth of 1 nm. The signal to noise ratio was improved by acquiring each spectrum over an average of two scans. Stern-Volmer quenching experiments were made by adding 0.5 µL of quinolonesubstrate solution (30 mM in THF) several times to give the appropriate spectra.

Luminescence lifetimes were recorded on Horiba DeltaFlex Modular Fluorescence Lifetime System (Time Correlated Single Photon Counting lifetime measurement). Samples were excited with a λ = 449 nm DeltaDiode (Horiba), an emission at 470 nm, a measurement range of 13 microseconds and with max signal bellow than 2.00%. A 0.01% solution of LUDOX AS-40 colloidal silica (40 wt. % suspensions in H_2O) in purified water was used as reference. Photocatalyst solutions were prepared with a concentration of 1.6 µM in H_2O and placed in a quartz cuvette (10x10mm light path) with a screw-cap. Samples were degassed for 20 minutes to remove oxygen and avoid the triplet excited-state quenching. The software DAS 6 was used to perform a monoexponential fitting analysis to calculate the lifetime (Figure S1 and S2).

Specific rotation experiment was performed on an Anton Paar MCP 4100 using a 10 cm quartz cuvette. Enantioenrichied product was diluted in 1 mL MeOH at a concentration of 0.75 mg/mL.

II. Synthesis of iridium complexes

1. Synthesis of iridium dimer

2-(2, 4-Difluorophenyl)-5-(trifluoromethyl)pyridine

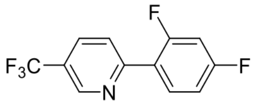

The title compound was prepared according to the previous procedure reported in the literature.[1] A dried-oven Schlenk was charged with 2-chloro-5-(trifluoromethyl)pyridine (1 equiv., 1.030 g), 2,4-difluorophenyl boronic acic (1.1 equiv., 960 mg), $Pd(PPh_3)_4$ (0.060 equiv., 188 mg), Na_2CO_3 (2 equiv., 1.060 g) [2M] solution in H_2O, toluene (6 mL) and ethanol (1.2 mL) and the reaction mixture was refluxed overnight. After this time, H_2O was added and the organic layer was extracted with CH_2Cl_2 (3 times), dried over $MgSO_4$, filtered and evaporated to dryness. Finally, the product was purified by flash column chromatography on silica gel (Hexane/EtOAc 95:05) to afford the desired product (1.311 g, 89 %). **^{1}H NMR** (400 MHz, $CDCl_3$) δ 8.99 – 8.93 (m, 1H), 8.10 (td, *J* = 8.9, 6.6 Hz, 1H), 7.99 (dd, *J* = 8.3, 2.5 Hz, 1H), 7.91 (ddt, *J* = 8.4, 1.8, 0.8 Hz, 1H), 7.04 (tdd, *J* = 7.7, 2.5, 1.0 Hz, 1H), 6.95 (ddd, *J* = 11.3, 8.7, 2.5 Hz, 1H). Spectroscopic data were consistent with the literature data.[1]

[Ir(dF(CF$_3$)ppy)$_2$Cl]$_2$

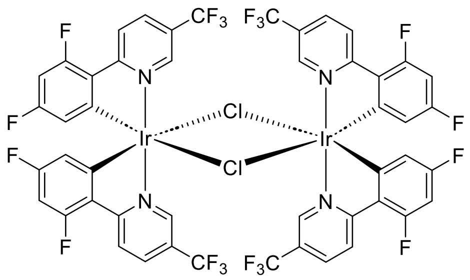

The title compound was prepared according to the previous procedure reported in the literature.[1] A dried-oven Schlenk was charged with 2-ethoxyethanol and H_2O 1:05, [0.055 M] with respect to substrate, and the solution was degassed. Then, $IrCl_3.xH_2O$ (1 equiv., 193 mg) and 2-(2,4-difluorophenyl)-5-(trifluoromethyl)pyridine (2.2 equiv., 370 mg) were added and the reaction mixture was refluxed overnight. After this time, the solution was filtered and the yellow precipitate was washed with H_2O, Et_2O and a minimum amount of CH_2Cl_2. The solid was dried under high *vaccum* to afford the desired product (382 mg, 79 %). **^{1}H NMR** (400 MHz, Acetone-*d*$_6$) δ 9.63 (d, *J* = 2.2 Hz, 4H), 8.68 (dd, *J* = 8.7, 2.5 Hz, 4H), 8.52 (dd, *J* = 8.7, 2.0 Hz, 4H), 6.69 (ddd, *J* = 12.6, 9.2, 2.3 Hz, 4H), 5.21 (ddd, *J* = 9.1, 2.3, 0.6 Hz, 4H). Spectroscopic data were consistent with the literature data.[1]

2. Synthesis of bipyridine ligands

4'-Methyl-[2,2'-bipyridine]-4-carboxylic acid

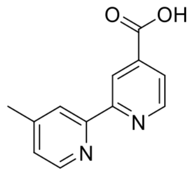

The title compound was prepared according to the previous procedure reported in the literature.[2] **^{1}H NMR** (400 MHz, DMSO-d_6) δ 8.85 (d, J = 5.0 Hz, 1H), 8.81 (s, 1H), 8.58 (d, J = 4.9 Hz, 1H), 8.27 (s, 1H), 7.86 (dd, J = 5.0, 1.7 Hz, 1H), 7.33 (d, J = 4.9 Hz, 1H), 2.43 (s, 3H). Spectroscopic data were consistent with the literature data.[2]

3-(4'-Methyl-[2,2'-bipyridin]-4-yl)propanoic acid

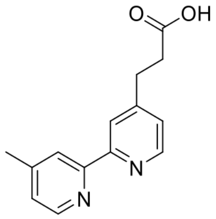

The title compound was prepared according to the previous procedure reported in the literature.[3] **^{1}H NMR** (400 MHz, DMSO-d_6) δ 12.22 (s, 1H), 8.54 (dd, J = 8.5, 5.2 Hz, 2H), 8.24 (d, J = 10.7 Hz, 2H), 7.33 (dd, J = 5.0, 1.7 Hz, 2H), 7.29 (dd, J = 4.9, 1.7 Hz, 2H), 2.93 (t, J = 7.4 Hz, 2H), 2.65 (t, J = 7.4 Hz, 2H), 2.41 (s, 3H). Spectroscopic data were consistent with the literature data.[3]

4-(4'-Methyl-[2,2'-bipyridin]-4-yl)butanoic acid

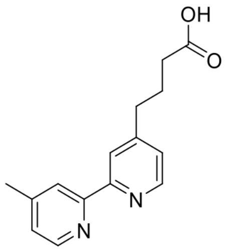

The title compound was prepared according to the previous procedure reported in the literature.[3] **^{1}H NMR** (400 MHz, D_2O) δ 8.69 (d, J = 5.5 Hz, 1H), 8.65 (d, J = 5.7 Hz, 1H), 8.29 (s, 1H), 8.23 (s, 1H), 7.77 (d, J = 5.7 Hz, 1H), 7.72 (d, J = 5.3 Hz, 1H), 2.95 (t, J = 7.6 Hz, 2H), 2.66 (s, 3H), 2.48 (t, J = 7.3 Hz, 2H), 2.07 (p, J = 7.5 Hz, 2H). Spectroscopic data were consistent with the literature data.[3]

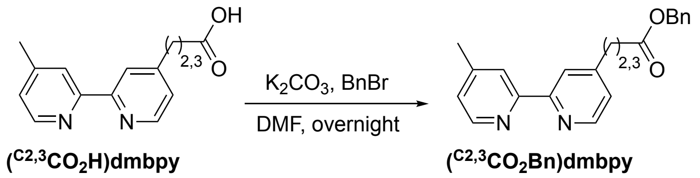

General benzylation procedure: In a dried-oven Schlenk, the corresponding **($^{C2,3}CO_2H$)dmbpy** (1 equiv.) was added, followed by dry K_2CO_3 (1.2 equiv.) and DMF [0.07 M]. The mixture was stirred few minutes, then benzyl bromide (2 equiv.) was added and the reaction was stirred overnight. After this time, solvent was removed under reduced pressure, H_2O was added and the organic layer was extracted three times with CH_2Cl_2. The combined organic layer was dried over $MgSO_4$, filtered and evaporated under *vaccum* to give the corresponding **($^{C2,3}CO_2Bn$)dmbpy** without any further purification.

Benzyl 3-(4'-methyl-[2,2'-bipyridin]-4-yl)propanoate

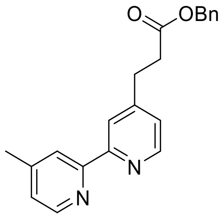

The title compound was obtained according to the general procedure above (127 mg, 62 %) from 3-(4'-methyl-[2,2'-bipyridin]-4-yl)propanoic acid (1 equiv., 150 mg). **^{1}H NMR** (400 MHz, $CDCl_3$) δ 8.51 (dd, *J* = 7.5, 5.0 Hz, 2H, CH_{Ar}), 8.25 (s, 1H, CH_{Ar}), 8.20 (s, 1H, CH_{Ar}), 7.31 – 7.27 (m, 5H, $CH_{Ar\text{-}benzyl}$), 7.09 (dt, *J* = 4.5, 2.0 Hz, 2H, CH_{Ar}), 5.09 (s, 2H, $CH_{2\text{-}benzyl}$), 3.02 (t, *J* = 7.7 Hz, 2H, CH_2), 2.75 (t, *J* = 7.7 Hz, 2H, CH_2), 2.39 (s, 3H, CH_3). **^{13}C NMR** (101 MHz, $CDCl_3$) δ 172.1 (C=O), 156.3 (C_{Ar}), 155.8 (C_{Ar}), 150.3 (C_{Ar}), 149.2 ($CH_{Ar\text{-}bpy}$), 148.9 ($CH_{Ar\text{-}bpy}$), 148.1 (C_{Ar}), 135.7 (C_{Ar}), 128.5 ($CH_{Ar\text{-}benzyl}$), 128.2 ($CH_{Ar\text{-}benzyl}$), 128.2 ($CH_{Ar\text{-}benzyl}$), 124.7 ($CH_{Ar\text{-}bpy}$), 123.7 ($CH_{Ar\text{-}bpy}$), 122.0 ($CH_{Ar\text{-}py}$), 121.0 ($CH_{Ar\text{-}bpy}$), 66.4 ($CH_{2\text{-}benzyl}$), 34.5 (CH_2), 30.3 (CH_2), 21.1 (CH_3). **HRMS** (ESI+) *m/z*: Calcd for $C_{21}H_{21}N_2O_2$ $[M+H]^+$: 333.1603 found 333.1609.

Benzyl 4-(4'-methyl-[2,2'-bipyridin]-4-yl)butanoate

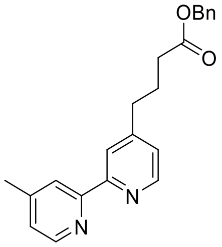

The title compound was obtained according to the general procedure above (42 mg, 40 %) from 4-(4'-methyl-[2,2'-bipyridin]-4-yl)butanoic acid (1 equiv., 80 mg). **^{1}H NMR** (400 MHz, $CDCl_3$) δ 8.54 (dd, *J* = 10.0, 5.0 Hz, 2H, CH_{Ar}), 8.22 (bs, 2H, CH_{Ar}), 7.41 – 7.27 (m, 5H, $CH_{Ar\text{-}benzyl}$), 7.17 – 7.08 (m, 2H, CH_{Ar}), 5.12 (s, 2H, $CH_{2\text{-}benzyl}$), 2.74 (t, *J* = 7.7 Hz, 2H, CH_2), 2.46 – 2.38 (m, 5H, CH_3 + CH_2), 2.06 (p, *J* = 7.5 Hz, 2H, CH_2). **^{13}C NMR** (101 MHz, $CDCl_3$) δ 173.0 (C=O), 156.4 (C_{Ar}), 156.1 (C_{Ar}), 151.4 (C_{Ar}), 149.2 ($CH_{Ar\text{-}bpy}$), 149.0 ($CH_{Ar\text{-}bpy}$), 148.2 (C_{Ar}), 136.1 (C_{Ar}), 128.7 ($CH_{Ar\text{-}benzyl}$), 128.3 (3 $CH_{Ar\text{-}benzyl}$), 124.8 ($CH_{Ar\text{-}bpy}$), 124.0 ($CH_{Ar\text{-}bpy}$), 122.1 ($CH_{Ar\text{-}bpy}$), 121.4 ($CH_{Ar\text{-}bpy}$), 66.4 ($CH_{2\text{-}benzyl}$), 34.7 (CH_2), 33.7 (CH_2), 25.5 (CH_2), 21.3 (CH_3). **HRMS** (ESI+) *m/z*: Calcd for $C_{22}H_{23}N_2O_2$ $[M+H]^+$: 347.1760 found 347.1760.

3. Synthesis of iridium racemic complexes

For [Ir(dF(CF_3)ppy)$_2$((^{C0}CO_2H)dmbpy)](PF_6) and [Ir(dF(CF_3)ppy)$_2$((^{C2}CO_2H)dmbpy)](PF_6) – General procedure A: In a dried-oven Schlenk, [Ir(dF(CF_3)ppy)$_2$Cl]$_2$ (0.5 equiv.) and the corresponding 4'-methyl-[2,2'-bipyridine]-4-carboxylic acid (^{C0}CO_2H)dmbpy) or 3-(4'-methyl-[2,2'-bipyridin]-4-yl)propanoic acid (^{C2}CO_2H)dmbpy) (1.1 equiv.) were solubilized in a mixture of MeOH/CH_2Cl_2, and the reaction was placed in an oil bath heated at 40°C overnight. After cooling to room temperature, NH_4PF_6 (2 equiv.) was added then solvent was removed under reduced pressure. The yellow solid was solubilized in CH_2Cl_2 and filtered to remove the salts. The desired pure complex was finally obtained by recrystallization in acetone/Et_2O.

For [Ir(dF(CF$_3$)ppy)$_2$((^{C3}CO$_2$H)dmbpy)](PF$_6$) – General procedure B: Step 1: In a dried-oven Schlenk, [Ir(dF(CF$_3$)ppy)$_2$Cl]$_2$ (0.5 equiv.) and the corresponding benzyl 4-(4'-methyl-[2,2'-bipyridin]-4-yl)butanoate (1.1 equiv.) were solubilized in a mixture of MeOH/CH$_2$Cl$_2$, and the reaction was placed in an oil bath heated at 40°C overnight. After cooling to room temperature, NH$_4$PF$_6$ (2 equiv.) was added then solvents were removed under reduced pressure and the desired complex was purified by silica gel flash chromatography (Hex/EtOAc 50:50). In some cases, a reprecipitation in CH$_2$Cl$_2$/Et$_2$O was necessary to remove the remaining [Ir(dF(CF$_3$)ppy)$_2$Cl]$_2$. Step 2: In a dried-oven flask, **[Ir(dF(CF$_3$)ppy)$_2$((^{C3}CO$_2$Bn)dmbpy)](PF$_6$)** (1.0 equiv.), Pd/C (10 wt. %), EtOH and CH$_2$Cl$_2$ were added. A balloon of H$_2$ was bubbled into the reaction mixture for 5 minutes then the reaction was stirred 2h under H$_2$ atmosphere. The mixture was filtered through Celite, and the solvent was removed under reducing pressure affording the corresponding desired deprotected product.

[Ir(dF(CF$_3$)ppy)$_2$((^{C0}CO$_2$H)dmbpy)](PF$_6$)

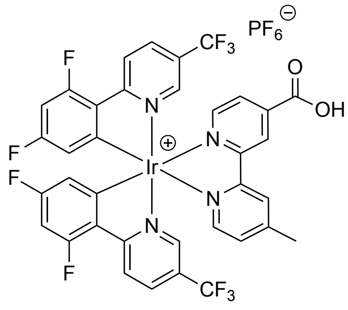

The title complex was obtained according to the general procedure A (90 mg, 78 %) from [Ir(dF(CF$_3$)ppy)$_2$Cl]$_2$ (1 equiv., 80 mg) and 4'-methyl-[2,2'-bipyridine]-4-carboxylic acid (2.1 equiv., 24 mg). **^{1}H NMR** (400 MHz, Acetone-*d*$_6$) δ 9.15 (s, 1H, CH$_{Ar}$), 9.04 (s, 1H, CH$_{Ar}$), 8.68 – 8.57 (m, 2H, CH$_{Ar}$), 8.47 (dd, *J* = 5.6, 0.8 Hz, 1H, CH$_{Ar}$), 8.45 – 8.36 (m, 2H, CH$_{Ar}$), 8.15 – 8.11 (m, 2H, CH$_{Ar}$), 8.09 (bs, 1H, CH$_{Ar}$), 7.99 (bs, 1H, CH$_{Ar}$), 7.70 – 7.65 (m, 1H, CH$_{Ar}$), 6.90 – 6.80 (m, 2H, CH$_{Ar}$), 5.97 (dd, *J* = 18.4, 2.4 Hz, 2H, CH$_{Ar}$), 2.68 (s, 3H, CH$_3$). **^{13}C NMR** (101 MHz, Acetone-*d*$_6$) δ 168.5 (t, J_{C-F} = 8.2 Hz, C$_{Ar-ppy}$), 165.5 (dd, J_{C-F} = 258.5, 4.4 Hz, C-F), 165.3 (dd, J_{C-F} = 258.4, 4.5 Hz, C-F), 164.6 (s, COOH), 163.4 (dd, J_{C-F} = 261.8, 3.4 Hz, C-F), 163.2 (dd, J_{C-F} = 261.9, 3.0 Hz, C-F), 158.3 (s, C$_{Ar-bpy}$), 155.8 (s, C$_{Ar-bpy)}$), 155.8 (dd, J = 20.2, 7.5 Hz, C$_{Ar-ppy}$), 154.5 (s, C$_{Ar-bpy}$), 153.5 (s, CH$_{Ar-bpy}$), 151.7 (s, CH$_{Ar-bpy}$), 147.3 (q, J_{C-F} = 4.6 Hz, CH$_{Ar}$), 147.1 (q, J_{C-F} = 4.7 Hz, CH$_{Ar}$), 142.4 (s, C$_{Ar-bpy}$), 138.2 (q, *J* = 3.3 Hz, CH$_{Ar}$), 131.0 (s, CH$_{Ar-bpy}$), 129.0 (s, CH$_{Ar-bpy}$), 127.8 (m, C$_{Ar-ppy}$), 127.6 (m, C$_{Ar-ppy}$), 127.6 (s, CH$_{Ar-bpy}$), 126.4 (q, J_{C-F} = 34.6 Hz, C-CF$_3$), 125.3 (s, CH$_{Ar-bpy}$), 124.9 (d, J_{C-F} = 3.0 Hz, CH$_{Ar-ppy}$), 124.7 (d, J_{C-F} = 3.0 Hz, CH$_{Ar-ppy}$), 123.0 (q, J_{C-F} = 271.7 Hz, CF$_3$), 123.0 (q, J_{C-F} = 271.5 Hz, CF$_3$), 115.4 (t, J_{C-F} = 18.5 Hz, CH$_{Ar-ppy}$), 115.3 (t, J_{C-F} = 18.2 Hz, CH$_{Ar-ppy}$), 100.3 (t, J_{C-F} = 27.1 Hz, CH$_{Ar-ppy}$), 100.3 (t, J_{C-F} = 27.1 Hz, CH$_{Ar-ppy}$), 21.3 (CH$_3$). **^{19}F NMR** (377 MHz, Acetone-*d*$_6$) δ -63.35 (s, CF3), -63.50 (s, CF3), -72.61 (d, PF$_6$, *J* = 707.8 Hz), -104.67 (m, 2CF), -107.88 (m, 2CF). **HRMS** (ESI+) *m/z*: Calcd for C$_{36}$H$_{20}$N$_4$O$_2$F$_{10}$Ir [M-PF$_6$]$^+$: 923.1072 found 923.1058.

[Ir(dF(CF$_3$)ppy)$_2$((^{C2}CO$_2$H)dmbpy)](PF$_6$)

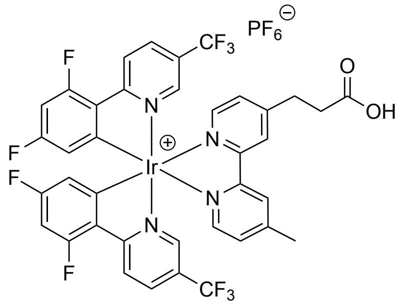

The title complex was obtained according to the general procedure A (72 mg, 49 %) from [Ir(dF(CF$_3$)ppy)$_2$Cl]$_2$ (1 equiv., 100 mg) and 3-(4'-methyl-[2,2'-bipyridin]-4-yl)propanoic acid (2.1 equiv., 34 mg). **^{1}H NMR** (400 MHz, Acetone-*d*$_6$) δ 8.84 (s, 1H, CH$_{Ar}$), 8.80 (s, 1H, CH$_{Ar}$), 8.61 (d, *J* = 8.9 Hz, 2H, CH$_{Ar}$), 8.39 (d, *J* = 8.8 Hz, 2H, CH$_{Ar}$), 8.12 (dd, *J* = 12.1, 5.7 Hz, 2H, CH$_{Ar}$), 8.02 (s, 1H, CH$_{Ar}$), 7.90 (s, 1H, CH$_{Ar}$), 7.69 (dd, *J* = 5.7, 1.7 Hz, 1H, CH$_{Ar}$), 7.62 (dd, *J* = 5.9, 1.7 Hz, 1H, CH$_{Ar}$), 6.82 (ddd, *J* = 12.8, 9.3, 2.4 Hz, 2H, CH$_{Ar}$), 5.95 (ddd, *J* = 8.0, 5.2, 2.3 Hz, 2H, CH$_{Ar}$), 3.19 (t, *J* = 7.4 Hz, 2H, CH$_2$), 2.80 (t, *J* = 7.4 Hz, 2H, CH$_2$), 2.63 (s, 3H, CH$_3$). **^{13}C NMR** (101 MHz, Acetone-*d*$_6$) δ 173.4 (s, COOH), 168.6 (d, J_{C-F} = 6.7 Hz, C$_{Ar-ppy}$), 165.4 (dd, J_{C-F} = 258.5, 12.6 Hz, C-F), 163.3 (dd, J_{C-F} = 262.0, 13.3 Hz, C-F), 157.1 (s, C$_{Ar-bpy}$), 156.6 (s, C$_{Ar-bpy}$), 156.5 (s, C$_{Ar-bpy}$), 156.4 (d, J_{C-F} = 6.9 Hz, C$_{Ar-ppy}$), 154.1 (s, C$_{Ar-bpy}$), 151.7 (s, CH$_{Ar-bpy}$), 151.4 (s, CH$_{Ar-bpy}$), 147.0 (q, J_{C-F} = 4.7 Hz, CH$_{Ar-ppy}$), 146.7 (q, J_{C-F} = 4.8 Hz, CH$_{Ar-ppy}$), 138.1 (q, J_{C-F} = 3.3 Hz, CH$_{Ar-ppy}$), 130.5 (s, CH$_{Ar-bpy}$), 129.9 (s, CH$_{Ar-bpy}$), 127.7 (m, C$_{Ar-ppy}$), 126.9 (s, CH$_{Ar-bpy}$), 126.3 (q, J_{C-F} = 34.7 Hz, C-CF$_3$), 126.2 (q, J_{C-F} = 34.3 Hz, C-CF$_3$), 126.2 (s, CH$_{Ar-bby}$), 124.7 (d, J_{C-F} = 21.2 Hz, CH$_{Ar-ppy}$), 123.0 (q, J_{C-F} = 271.8 Hz, CF$_3$), 123.0 (q, J_{C-F} = 271.8 Hz, CF$_3$), 115.4 (dd, J_{C-F} = 7.5, 3.0 Hz, CH$_{Ar-ppy}$), 115.2 (dd, J_{C-F} = 7.4, 3.0 Hz, CH$_{Ar-ppy}$), 100.1 (t, J_{C-F} = 27.1 Hz, CH$_{Ar-ppy}$), 34.0 (s, CH$_2$), 30.9 (s, CH$_2$), 21.3 (s, CH$_3$). **^{19}F NMR (**377 MHz, Acetone-*d*$_6$) δ -63.44 (s, CF$_3$), -63.49 (s, CF$_3$), -72.41 (d, J = 707.3 Hz, PF$_6$), -104.62 – -104.90 (m, 2CF), -107.88 – -108.05 (m, 2CF). **HRMS** (ESI+) *m/z*: Calcd for $C_{38}H_{24}N_4O_2F_{10}Ir$ [M-PF$_6$]$^+$: 951.1369 found 951.1378.

[Ir(dF(CF$_3$)ppy)$_2$((^{C3}CO$_2$Bn)dmbpy)](PF$_6$)

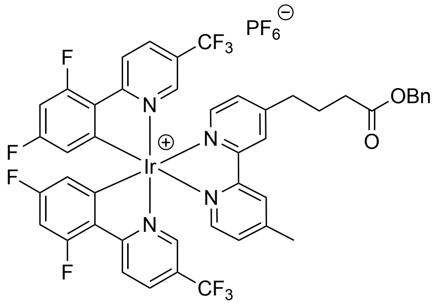

The title complex was obtained according to the general procedure B-step1 (75 mg, 82 %) from [Ir(dF(CF$_3$)ppy)$_2$Cl]$_2$ (1 equiv., 75 mg) and benzyl 4-(4'-methyl-[2,2'-bipyridin]-4-yl)butanoate (1.5 equiv., 40 mg). **^{1}H NMR** (400 MHz, Acetone-*d*$_6$) δ 8.79 (s, 2H, CH$_{Ar}$), 8.60 (dd, *J* = 8.9, 2.7 Hz, 2H, CH$_{Ar}$), 8.39 (dd, *J* = 8.9, 2.1 Hz, 2H, CH$_{Ar}$), 8.13 (d, *J* = 5.7 Hz, 1H, CH$_{Ar}$), 8.10 (d, *J* = 5.6 Hz, 1H, CH$_{Ar}$), 8.00 (s, 1H, CH$_{Ar}$), 7.92 (s, 1H, CH$_{Ar}$), 7.63 (t, *J* = 7.0 Hz, 2H, CH$_{Ar}$), 7.39 – 7.30 (m, 5H, CH$_{Benzyl}$), 6.90 – 6.77 (m, 2H, CH$_{Ar}$), 6.00 – 5.89 (m, 2H, CH$_{Ar}$), 5.10 (s, 2H, CH$_{2-Benzyl}$), 2.97 (t, *J* = 7.8 Hz, 2H, CH$_2$), 2.85 – 2.79 (m, 2H, CH$_2$), 2.62 (s, 3H, CH$_3$), 2.46 (t, *J* = 7.4 Hz, 2H, CH$_2$). **^{13}C NMR** (101 MHz, Acetone-*d*$_6$) δ 173.0 (s, COOH), 168.6 (d, J_{C-F} = 6.9 Hz, C$_{Ar-ppy}$), 165.4 (dd, J_{C-F} = 258.4, 12.6 Hz, C-F), 163.3 (dd, J_{C-F} = 261.8, 13.2 Hz, C-F), 157.4 (s, C$_{Ar-bpy}$), 156.8 (s, C$_{Ar-bpy}$), 156.5

(s, $C_{Ar\text{-}bpy}$), 156.4 (d, $J_{C\text{-}F}$ = 7.0 Hz, $C_{Ar\text{-}ppy}$), 154.1 (s, $C_{Ar\text{-}bpy}$), 151.9 (s, $CH_{Ar\text{-}bpy}$), 151.5 (s, $CH_{Ar\text{-}bpy}$), 147.0 (q, $J_{C\text{-}F}$ = 4.8 Hz, $CH_{Ar\text{-}ppy}$), 146.8 (q, $J_{C\text{-}F}$ = 4.7 Hz, $CH_{Ar\text{-}ppy}$), 138.2 – 137.9 (m, $CH_{Ar\text{-}ppy}$), 137.4 (s, $C_{Ar\text{-}benzyl}$), 130.5 (s, $CH_{Ar\text{-}bpy}$), 129.9 (s, $CH_{Ar\text{-}bpy}$), 129.3 (s, $CH_{Ar\text{-}benzyl}$), 128.9 (s, $CH_{Ar\text{-}benzyl}$), 128.9 (s, $CH_{Ar\text{-}benzyl}$), 127.7 (m, $C_{Ar\text{-}ppy}$), 126.9 (s, $CH_{Ar\text{-}bpy}$), 126.2 (q, $J_{C\text{-}F}$ = 34.3 Hz, C-CF_3), 126.2 (s, $CH_{Ar\text{-}bpy}$), 124.7 (d, $J_{C\text{-}F}$ = 20.9 Hz, $CH_{Ar\text{-}ppy}$), 123.0 (q, $J_{C\text{-}F}$ = 272.7 Hz, CF_3), 115.4 (dd, $J_{C\text{-}F}$ = 7.6, 3.0 Hz, $CH_{Ar\text{-}ppy}$), 115.2 (dd, $J_{C\text{-}F}$ = 7.6, 3.0 Hz, $CH_{Ar\text{-}ppy}$), 100.1 (t, $J_{C\text{-}F}$ = 27.1 Hz, $CH_{Ar\text{-}ppy}$), 66.5 (s, $CH_{2\text{-}benzyl}$), 35.0 (s, CH_2), 33.6 (s, CH_2), 26.0 (s, CH_2), 21.4 (s, CH_3). **^{19}F NMR** (377 MHz, Acetone-d_6) δ -63.45 (s, CF_3), -63.48 (s, CF_3), -72.47 (d, J = 707.5 Hz, PF_6), -104.76 (m, 2CF), -107.97 (m, 2CF). **HRMS** (ESI+) *m/z*: Calcd for $C_{46}H_{32}N_4O_2F_{10}Ir$ [M-PF_6]$^+$: 1055.1995 found 1055.1990.

[Ir(dF(CF$_3$)ppy)$_2$((^{C3}CO$_2$H)dmbpy)](PF$_6$)

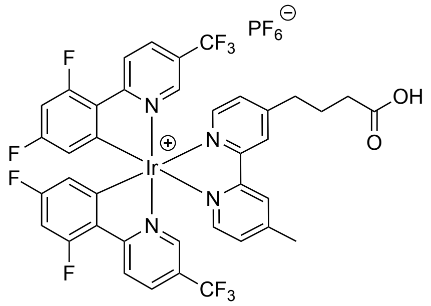

The title complex was obtained according to the general procedure B-step2 (26 mg, 80 %) from **[Ir(dF(CF$_3$)ppy)$_2$((^{C3}CO$_2$Bn)dmbpy)](PF$_6$)** (1 equiv., 36 mg). **^{1}H NMR** (400 MHz, Acetone-d_6) δ 8.83 (s, 1H, CH_{Ar}), 8.81 (s, 1H, CH_{Ar}), 8.64 – 8.55 (m, 2H, CH_{Ar}), 8.43 – 8.36 (m, 2H, CH_{Ar}), 8.12 (dd, J = 14.8, 5.4 Hz, 2H, CH_{Ar}), 8.00 (s, 1H, CH_{Ar}), 7.92 (s, 1H, CH_{Ar}), 7.64 (dd, J = 17.9, 5.5 Hz, 2H, CH_{Ar}), 6.88 – 6.79 (m, 2H, CH_{Ar}), 5.99 – 5.90 (m, 2H, CH_{Ar}), 3.02 – 2.93 (m, 2H, CH_2), 2.63 (s, 3H, CH_3), 2.37 (t, J = 7.3 Hz, 2H, CH_2), 2.00 (t, J = 7.8 Hz, 2H, CH_2). **^{13}C NMR** (101 MHz, Acetone-d_6) δ 174.2 (s, COOH), 168.6 (d, $J_{C\text{-}F}$ = 7.3 Hz, $C_{Ar\text{-}ppy}$), 165.4 (dd, $J_{C\text{-}F}$ = 258.5, 12.7 Hz, C-F), 163.3 (dd, $J_{C\text{-}F}$ = 261.8, 13.2 Hz, C-F), 157.6 (s, $C_{Ar\text{-}bpy}$), 156.8 (s, $C_{Ar\text{-}bpy}$), 156.5 (s, $C_{Ar\text{-}bpy}$), 156.5 (d, $J_{C\text{-}F}$ = 7.1 Hz, $C_{Ar\text{-}ppy}$), 156.5 (d, $J_{C\text{-}F}$ = 6.8 Hz, $C_{Ar\text{-}ppy}$), 154.1 (s, $C_{Ar\text{-}bpy}$), 151.9 (s, $CH_{Ar\text{-}bpy}$), 151.5 (s, $CH_{Ar\text{-}bpy}$), 147.0 (q, $J_{C\text{-}F}$ = 4.8 Hz, $CH_{Ar\text{-}ppy}$), 146.8 (q, $J_{C\text{-}F}$ = 4.8 Hz, $CH_{Ar\text{-}ppy}$), 138.1 (bs, $CH_{Ar\text{-}ppy}$), 130.5 (s, $CH_{Ar\text{-}bpy}$), 129.9 (s, $CH_{Ar\text{-}bpy}$), 127.8 (m, $C_{Ar\text{-}ppy}$), 127.0 (s, $CH_{Ar\text{-}bpy}$), 126.3 (q, $J_{C\text{-}F}$ = 33.6 Hz, C-CF_3), 126.2 (s, $CH_{Ar\text{-}bpy}$), 124.7 (d, $J_{C\text{-}F}$ = 20.9 Hz, $CH_{Ar\text{-}ppy}$), 123.0 (q, $J_{C\text{-}F}$ = 272.3 Hz, CF_3), 115.4 (dd, $J_{C\text{-}F}$ = 7.2, 3.1 Hz, $CH_{Ar\text{-}ppy}$), 115.3 (dd, $J_{C\text{-}F}$ = 7.0, 2.9 Hz, $CH_{Ar\text{-}ppy}$), 100.1 (t, $J_{C\text{-}F}$ = 27.1 Hz, $CH_{Ar\text{-}ppy}$), 35.1 (CH_2), 33.4 (CH_2), 26.1 (CH_2), 21.4 (CH_3). **^{19}F NMR** (377 MHz, Acetone-d_6) δ -63.49 (s, CF_3), -63.54 (s, CF_3), -72.56 (d, J = 709.0 Hz, PF_6), -104.74 – -104.91 (m, 2CF), -108.00 – -108.16 (m, 2CF). **HRMS** (ESI+) *m/z*: Calcd for $C_{39}H_{26}N_4O_2F_{10}Ir$ [M-PF_6]$^+$: 965.1525 found 965.1525.

4. Synthesis of enantiopur iridium complexes

(*S*)-2-(4-isopropyl-4,5-dihydrooxazol-2-yl)phenol

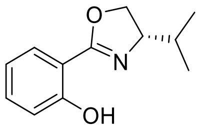

The title compound (*S*-oxazoline) was prepared according to the previous procedure reported in the literature.[4] **^{1}H NMR** (400 MHz, $CDCl_3$) δ 7.63 (d, *J* = 7.8 Hz, 1H), 7.37 (t, *J* = 7.8 Hz, 1H), 7.02 (d, *J* = 8.3 Hz, 1H), 6.86 (t, *J* = 7.5 Hz, 1H), 4.51 – 4.37 (m, 1H), 4.18 – 4.08 (m, 2H), 1.81 (h, *J* = 6.6 Hz, 1H), 1.02 (d, *J* = 6.8 Hz, 3H), 0.95 (d, *J* = 6.7 Hz, 3H). Spectroscopic data were consistent with the literature data.[4]

The complexes **Λ[Ir(dF(CF$_3$)ppy)$_2$(*S*-oxazoline)]** and **Δ[Ir(dF(CF$_3$)ppy)$_2$(*S*-oxazoline)]** were prepared according to the previous procedure reported in the literature[5]. In a dried-oven Schlenk, [Ir(dF(CF$_3$)ppy)$_2$Cl]$_2$ (1.0 equiv.), EtOH (0.025 M), **(S)-2-(4-isopropyl-4,5-dihydrooxazol-2-yl)phenol** (2.5 equiv.) were added, followed by NEt_3 (10 equiv.) and the reaction was refluxed (95°C) overnight. After this time, the solvent was removed under *vaccum* and the two diastereoisomers were separated by silica gel flash chromatography (Hex/EtOAc 95:05).

Λ[Ir(dF(CF$_3$)ppy)$_2$(*S*-oxazoline)]

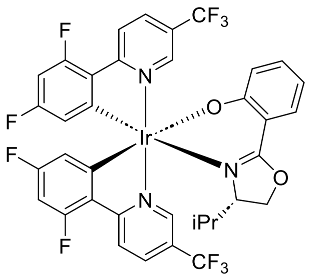

The title complex was obtained according to the procedure above (53 mg, 90 %) from [Ir(dF(CF$_3$)ppy)$_2$Cl]$_2$ (1.0 equiv., 95 mg) and (S)-2-(4-isopropyl-4,5-dihydrooxazol-2-yl)phenol (2.8 equiv., 37 mg).
^{1}H NMR (300 MHz, $CDCl_3$) δ 9.37 (s, 1H, CH_{Ar}), 8.65 (s, 1H, CH_{Ar}), 8.42 (dd, *J* = 8.7, 2.4 Hz, 1H, CH_{Ar}), 8.32 (dd, *J* = 8.8, 3.2 Hz, 1H, CH_{Ar}), 8.03 (dd, *J* = 8.8, 2.2 Hz, 1H, CH_{Ar}), 7.88 (dd, *J* = 8.8, 2.1 Hz, 1H, CH_{Ar}), 7.62 (dd, *J* = 8.2, 1.9 Hz, 1H, CH_{Ar}), 7.14 (ddd, *J* = 8.7, 6.8, 1.9 Hz, 1H, CH_{Ar}), 6.64 (dd, *J* = 8.7, 1.1 Hz, 1H, CH_{Ar}), 6.52 – 6.31 (m, 3H, CH_{Ar}), 5.84 (dd, *J* = 8.5, 2.3 Hz, 1H, CH_{Ar}), 5.46 (dd, *J* = 8.6, 2.3 Hz, 1H, CH_{Ar}), 4.34 (dd, *J* = 9.3, 3.9 Hz, 1H, CH_2), 4.26 (t, *J* = 9.2 Hz, 1H, CH_2), 3.84 (dt, *J* = 9.1, 3.7 Hz, 1H, CH*), 0.53 (qd, *J* = 6.7, 2.9 Hz, 1H, CH_{iPr}), 0.41 (d, *J* = 6.9 Hz, 3H, CH_3), 0.15 (d, *J* = 6.7 Hz, 3H, CH_3). **^{13}C NMR** (101 MHz, $CDCl_3$) δ 169.5 (s, C_{Ar}), 169.3 (d, $J_{C\text{-}F}$ = 7.0 Hz, $C_{Ar\text{-}ppy}$), 168.8 (d, $J_{C\text{-}F}$ = 7.0 Hz, $C_{Ar\text{-}ppy}$), 164.4 (dd, $J_{C\text{-}F}$ = 259.0, 12.7 Hz, C-F), 164.0 (dd, $J_{C\text{-}F}$ = 258.6, 12.6 Hz, C-F), 163.6 (s, C_{Ar}), 162.4 (dd, $J_{C\text{-}F}$ = 261.5, 13.4 Hz, C-F), 162.1 (dd, $J_{C\text{-}F}$ = 261.1, 13.1 Hz, C-F), 157.9 (d, $J_{C\text{-}F}$ = 6.8 Hz, $C_{Ar\text{-}ppy}$), 155.3 (d, $J_{C\text{-}F}$ = 6.9 Hz, $C_{Ar\text{-}ppy}$), 145.9 (q, $J_{C\text{-}F}$ = 4.5 Hz, $CH_{Ar\text{-}ppy}$), 145.0 (q, $J_{C\text{-}F}$ = 4.8 Hz, $CH_{Ar\text{-}ppy}$), 135.3 (q, $J_{C\text{-}F}$ = 3.5 Hz, $CH_{Ar\text{-}ppy}$), 134.9 (q, $J_{C\text{-}F}$ = 3.3 Hz, $CH_{Ar\text{-}ppy}$), 134.61 (s, CH_{Ar}), 129.9 (s, CH_{Ar}), 127.6 – 127.5 (m, $C_{Ar\text{-}ppy}$), 127.0 – 126.9 (m, $C_{Ar\text{-}ppy}$), 124.7 (q, $J_{C\text{-}F}$ = 34.8 Hz, C-CF$_3$), 124.3 (q, $J_{C\text{-}F}$ = 34.3 Hz, C-CF$_3$), 124.3 (s, CH_{Ar}), 122.7 (d, $J_{C\text{-}F}$ = 21.0 Hz, $CH_{Ar\text{-}ppy}$), 122.2 (d, $J_{C\text{-}F}$ = 19.9 Hz, $CH_{Ar\text{-}ppy}$), 122.6 (q, $J_{C\text{-}F}$ = 272.1 Hz, CF_3), 122.5 (q, $J_{C\text{-}F}$ = 272.4 Hz, CF_3), 114.9 (dd, $J_{C\text{-}F}$ =

16.6, 2.9 Hz, CH_{Ar-ppy}), 114.3 (s, CH_{Ar}), 113.8 (dd, J_{C-F} = 17.2, 2.9 Hz, CH_{Ar-ppy}), 110.0 (s, C_{Ar}), 98.5 (t, J_{C-F} = 26.9 Hz, CH_{Ar-ppy}), 97.4 (t, J_{C-F} = 26.9 Hz, CH_{Ar-ppy}), 71.7 (CH^*), 67.4 (CH_2), 29.6 (CH_{iPr}), 18.6 (CH_3), 12.9 (CH_3). **^{19}F NMR** (282 MHz, $CDCl_3$) δ -62.38 (s, CF_3), -62.68 (s, CF_3), -104.87 (ddt, *J* = 18.0, 11.8, 8.9 Hz, 2CF), -108.43 (m, CF), -108.79 (m, CF). **HRMS** (ESI+) *m/z*: Calcd for $C_{36}H_{25}N_3O_2F_{10}Ir$ $[M+H]^+$: 914.1416 found 914.1432.

Δ[Ir(dF(CF$_3$)ppy)$_2$(*S*-oxazoline)]

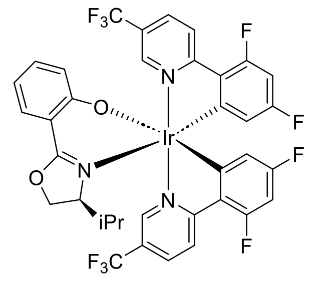

The title complex was obtained according to the procedure above (51 mg, 87 %) from $[Ir(dF(CF_3)ppy)_2Cl]_2$ (1.0 equiv., 95 mg) and (S)-2-(4-isopropyl-4,5-dihydrooxazol-2-yl)phenol (2.8 equiv., 37 mg). **^{1}H NMR** (400 MHz, $CDCl_3$) δ 9.22 (s, 1H, CH_{Ar}), 8.79 (s, 1H, CH_{Ar}), 8.42 (dd, *J* = 8.7, 2.4 Hz, 1H, CH_{Ar}), 8.34 (dd, *J* = 8.8, 2.9 Hz, 1H, CH_{Ar}), 8.04 (dd, *J* = 8.7, 2.2 Hz, 1H, CH_{Ar}), 7.90 (dd, *J* = 8.7, 2.1 Hz, 1H, CH_{Ar}), 7.53 (dd, *J* = 8.0, 1.9 Hz, 1H, CH_{Ar}), 7.10 (ddd, *J* = 8.7, 6.9, 1.9 Hz, 1H, CH_{Ar}), 6.53 (dd, *J* = 8.6, 1.1 Hz, 1H, CH_{Ar}), 6.49 – 6.29 (m, 3H, CH_{Ar}), 5.93 (dd, *J* = 8.5, 2.3 Hz, 1H, CH_{Ar}), 5.54 (dd, *J* = 8.5, 2.3 Hz, 1H, CH_{Ar}), 4.33 (dd, *J* = 9.1, 3.7 Hz, 1H, CH_2), 3.80 (t, *J* = 9.3 Hz, 1H, CH_2), 3.02 (ddd, *J* = 9.5, 3.7, 2.0 Hz, 1H, CH^*), 1.65 – 1.53 (m, 1H, CH_{iPr}), 0.86 (d, *J* = 6.8 Hz, 3H, CH_3), 0.38 (d, *J* = 7.0 Hz, 3H, CH_3). **^{13}C NMR** (101 MHz, $CDCl_3$) δ 169.1 (s, C_{Ar}), 168.9 (d, J_{C-F} = 7.1 Hz, C_{Ar-ppy}), 168.6 (d, J_{C-F} = 6.7 Hz, C_{Ar-ppy}), 164.0 (dd, J_{C-F} = 259.4, 12.8 Hz, C-F), 163.5 (s, C_{Ar}), 162.5 (dd, J_{C-F} = 261.5, 13.3 Hz, C-F), 162.3 (dd, J_{C-F} = 261.6, 12.9 Hz, C-F), 157.0 (d, J_{C-F} = 7.9 Hz, C_{Ar-ppy}), 156.9 (d, J_{C-F} = 8.1 Hz, C_{Ar-ppy}), 147.4 (q, J_{C-F} = 4.3 Hz, CH_{Ar-ppy}), 146.1 (q, J_{C-F} = 4.5 Hz, CH_{Ar-ppy}), 135.5 (q, J_{C-F} = 3.3 Hz, CH_{Ar-ppy}), 134.9 (q, J_{C-F} = 3.6 Hz, CH_{Ar-ppy}), 134.2 (s, CH_{Ar}), 128.9 (s, CH_{Ar}), 127.2 (m, C_{Ar-ppy}), 126.92 (m C_{Ar-ppy}), 125.0 (q, J_{C-F} = 35.0 Hz, C-CF_3), 124.1 (s, CH_{Ar}), 124.1 (q, J_{C-F} = 34.5 Hz, C-CF_3), 122.4 (q, J_{C-F} = 274.3 Hz, CF_3), 122.4 (q, J_{C-F} = 274.2 Hz, CF_3), 122.2 (d, J_{C-F} = 20.7 Hz, CH_{Ar-ppy}), 122.0 (d, J_{C-F} = 19.6 Hz, CH_{Ar-ppy}), 116.5 (dd, J_{C-F} = 16.7, 2.9 Hz, CH_{Ar-ppy}), 114.6 (dd, J_{C-F} = 17.0, 2.9 Hz, CH_{Ar-ppy}), 114.4 (s, CH_{Ar}), 111.1 (s, C_{Ar}), 98.4 (t, J_{C-F} = 26.9 Hz, CH_{Ar-ppy}), 97.4 (t, J_{C-F} = 26.8 Hz, CH_{Ar-ppy}), 10.0 (s, CH*), 67.6 (s, CH_2), 29.4 (s, CH_{iPr}), 19.8 (s, CH_3), 14.2 (s, CH_3). **^{19}F NMR** (377 MHz, $CDCl_3$) δ -62.15 (s, CF_3), -62.18 (s, CF_3), -105.18 (q, *J* = 8.8 Hz, CF), -105.45 (q, *J* = 10.3, 9.7 Hz, CF), -108.45 (t, *J* = 11.7 Hz, CF), -108.99 (t, *J* = 12.3 Hz, CF). **HRMS** (ESI+) *m/z*: Calcd for $C_{36}H_{25}N_3O_2F_{10}Ir$ $[M+H]^+$: 914.1416 found 914.1433.

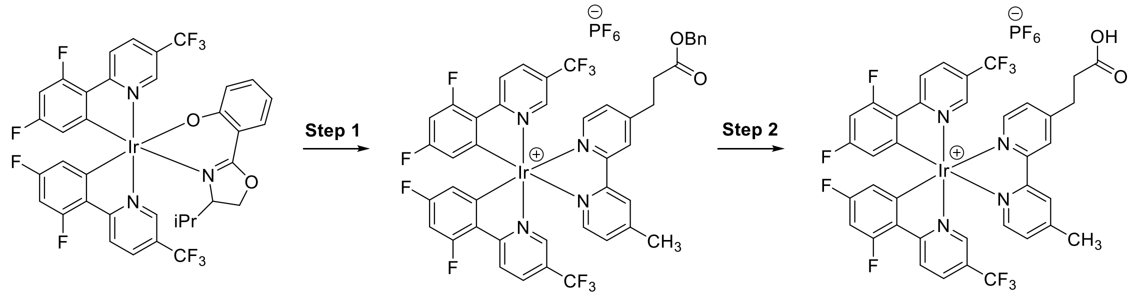

Step 1: In a dried-oven Schlenk, **Λ[Ir(dF(CF$_3$)ppy)$_2$(*S*-oxazoline)]** or **Δ[Ir(dF(CF$_3$)ppy)$_2$(*S*-oxazoline)]** (1.0 equiv.), NH_4PF_6 (5.0 equiv.), **benzyl 3-(4'-methyl-[2,2'-bipyridin]-4-yl)propanoate** ligand (2.0 equiv.) and dry MeCN (0.01 M) were added. A solution of trifluoroacetic acid (5.0 equiv.) in MeCN (0.2 M) was added slowly, and the solution was strirred at room temperature overnight. After this time, the reaction was concentrated under *vaccum* and the crude residue was precipitated by vapor diffusion in CH_2Cl_2 with an excess of Et_2O to remove most of the impurities. Finally, the product was purified by flash column chromatography on silica gel (CH_2Cl_2:Acetone 100:00 to 95:05) to afford the corresponding enantiopurs iridium complexes **Λ[Ir(dF(CF$_3$)ppy)$_2$((^{C2}CO$_2$Bn)dmbpy)](PF$_6$)** or **Δ[Ir(dF(CF$_3$)ppy)$_2$((^{C2}CO$_2$Bn)dmbpy)](PF$_6$)**.

Step 2: In a round-bottomed flask, **Λ[Ir(dF(CF$_3$)ppy)$_2$((^{C2}CO$_2$Bn)dmbpy)](PF$_6$)** or **Δ[Ir(dF(CF$_3$)ppy)$_2$((^{C2}CO$_2$Bn)dmbpy)](PF$_6$)** (1.0 equiv.), Pd/C (10 wt. %), EtOH and CH_2Cl_2 were added. A balloon of H_2 was bubbled into the reaction mixture for 5 minutes then the reaction was stirred 2h under H_2 atmosphere. The mixture was filtered through Celite, and the solvent was removed under reducing pressure affording the corresponding desired deprotected product **Λ[Ir(dF(CF$_3$)ppy)$_2$((^{C2}CO$_2$H)dmbpy)](PF$_6$)** or **Δ[Ir(dF(CF$_3$)ppy)$_2$((^{C2}CO$_2$H)dmbpy)](PF$_6$)**.

Λ[Ir(dF(CF$_3$)ppy)$_2$((^{C2}CO$_2$Bn)dmbpy)](PF$_6$)

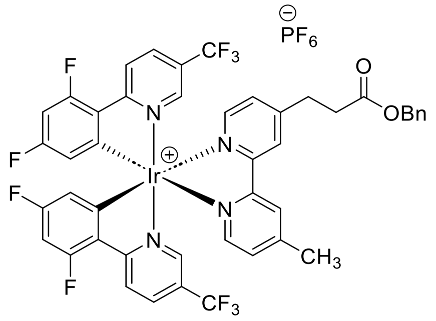

The title complex was obtained according to the procedure above (step 1) (32 mg, 68 %) from **Λ[Ir(dF(CF$_3$)ppy)$_2$(*S*-oxazoline)](PF$_6$)** (1.0 equiv., 37 mg) and 3-(4'-methyl-[2,2'-bipyridin]-4-yl)propanoic acid (2.5 equiv., 33 mg). **^{1}H NMR** (400 MHz, Acetone-*d*$_6$) δ 8.83 (s, 1H, CH_{Ar}), 8.76 (s, 1H, CH_{Ar}), 8.66 – 8.57 (m, 2H, CH_{Ar}), 8.40 (dt, *J* = 8.8, 2.8 Hz, 2H, CH_{Ar}), 8.10 (d, *J* = 5.7 Hz, 2H, CH_{Ar}), 8.03 – 7.97 (m, 1H, CH_{Ar}), 7.91 – 7.86 (m, 1H, CH_{Ar}), 7.68 – 7.59 (m, 2H, CH_{Ar}), 7.39 – 7.29 (m, 5H, $CH_{Ar\text{-}benzyl}$), 6.84 (dddd, *J* = 12.8, 9.3, 5.3, 2.3 Hz, 2H, CH_{Ar}), 5.98 – 5.90 (m, 2H, CH_{Ar}), 5.10 (s, 2H, $CH_{2\text{-}}$

benzyl), 3.24 (t, *J* = 7.4 Hz, 2H, CH_2), 2.90 (t, *J* = 7.4 Hz, 2H, CH_2), 2.62 (s, 3H, CH_3). **^{13}C NMR** (101 MHz, Acetone-d_6) δ 172.1 (s, COOH), 168.6 (d, J_{C-F} = 7.0 Hz, C_{Ar-ppy}), 165.4 (dd, J_{C-F} = 258.6, 12.5 Hz, C-F), 163.3 (dd, J_{C-F} = 261.8, 13.3 Hz, C-F), 156.7 (s, C_{Ar-bpy}), 156.6 (s, C_{Ar-bpy}), 156.4 (s, C_{Ar-bpy}), 156.3 (d, J_{C-F} = 7.9 Hz, C_{Ar-ppy}), 154.1 (s, C_{Ar-bpy}), 151.8 (s, CH_{Ar-bpy}), 151.5 (s, CH_{Ar-bpy}), 147.0 (q, J_{C-F} = 4.7 Hz, CH_{Ar-ppy}), 146.7 (q, J_{C-F} = 4.9 Hz, CH_{Ar-ppy}),138.1 (q, J_{C-F} = 3.3 Hz, CH_{Ar-ppy}), 137.2 (s, $C_{Ar-benzyl}$), 130.6 (s, CH_{Ar-bpy}), 129.8 (s, CH_{Ar-bpy}), 129.3 (s, $CH_{Ar-benzyl}$), 129.0 (s, $CH_{Ar-benzyl}$), 128.9 (s, $CH_{Ar-benzyl}$), 127.7 (m, C_{Ar-ppy}), 126.9 (s, CH_{Ar-bpy}), 126.3 (q, J_{C-F} = 34.7 Hz, C-CF_3), 126.2 (s, CH_{Ar-bpy}), 124.7 (d, J_{C-F} = 21.1 Hz, CH_{Ar-ppy}), 123.0 (q, J_{C-F} = 271.9 Hz, CF_3), 115.4 (dd, J_{C-F} = 8.1, 3.0 Hz, CH_{Ar-ppy}), 115.2 (dd, J_{C-F} = 8.1, 3.0 Hz CH_{Ar-ppy}), 100.1 (t, J_{C-F} = 27.2 Hz, CH_{Ar-ppy}), 66.8 (s, $CH_{2-benzyl}$), 34.1 (s, CH_2), 30.8 (s, CH_2), 21.4 (s, CH_3). **^{19}F NMR** (377 MHz, Acetone-d_6) δ -63.46 (s, CF_3), -63.47 (s, CF_3), -72.52 (d, *J* = 707.5 Hz, PF_6), -104.24 – -105.55 (m, 2CF), -107.99 (m, 2CF). **HRMS** (ESI+) *m/z*: Calcd for $C_{45}H_{30}N_4O_2F_{10}Ir$ $[M-PF_6]^+$: 1041.1838 found 1041.1837.

Δ[Ir(dF(CF$_3$)ppy)$_2$((^{C2}CO$_2$Bn)dmbpy)](PF$_6$)

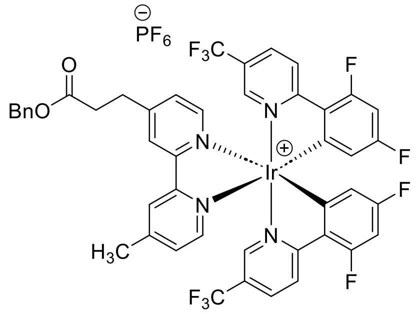

The title complex was obtained according to the procedure above (step 1) (20 mg, 77 %) from **Δ[Ir(dF(CF$_3$)ppy)$_2$(*S*-oxazoline)PF$_6$)** (1.0 equiv., 20 mg) and 3-(4'-methyl-[2,2'-bipyridin]-4-yl)propanoic acid (2.5 equiv., 18 mg). **^{1}H NMR** (400 MHz, Acetone-d_6) δ 8.83 (s, 1H, CH_{Ar}), 8.76 (s, 1H, CH_{Ar}), 8.66 – 8.57 (m, 2H, CH_{Ar}), 8.44 – 8.36 (m, 2H, CH_{Ar}), 8.11 (d, *J* = 5.6 Hz, 2H, CH_{Ar}), 8.01 (s, 1H, CH_{Ar}), 7.89 (s, 1H, CH_{Ar}), 7.68 – 7.59 (m, 2H, CH_{Ar}), 7.37 – 7.31 (m, 5H, CH_{Ar}), 6.89 – 6.79 (m, 2H, $CH_{Ar-benzyl}$), 5.95 (dddt, *J* = 8.4, 7.2, 2.3, 1.1 Hz, 2H, CH_{Ar}), 5.11 (s, 2H, $CH_{2-benzyl}$), 3.24 (t, *J* = 7.4 Hz, 2H, CH_2), 2.96 – 2.88 (m, 2H, CH_2), 2.62 (s, 3H, CH_3). **^{13}C NMR** (101 MHz, Acetone-d_6) δ 172.1 (s, COOH), 168.6 (d, J_{C-F} = 7.1 Hz, C_{Ar-ppy}), 165.4 (dd, J_{C-F} = 258.5, 12.5 Hz, C-F), 163.3 (dd, J_{C-F} = 261.9, 13.2 Hz, C-F), 156.7 (s, C_{Ar-bpy}), 156.6 (s, C_{Ar-bpy}), 156.4 (s, C_{Ar-bpy}), 156.3 (m, C_{Ar-ppy}), 154.1 (s, C_{Ar-bpy}), 151.8 (s, C_{Ar-bpy}), 151.5 (s, C_{Ar-bpy}), 146.9 (q, J_{C-F} = 4.8 Hz, CH_{Ar-ppy}), 146.7 (q, J_{C-F} = 4.8 Hz, CH_{Ar-ppy}), 138.1 (q, J_{C-F} = 3.3 Hz, CH_{Ar-ppy}), 137.2 (s, $C_{Ar-benzyl}$), 130.6 (s, CH_{Ar-bpy}), 129.8 (s, CH_{Ar-bpy}), 129.3 (s, $CH_{Ar-benzyl}$), 129.0 (s, $CH_{Ar-benzyl}$), 128.9 (s, $CH_{Ar-benzyl}$), 127.7 (m, CH_{Ar-ppy}), 126.9 (s, CH_{Ar-bpy}), 126.3 (q, J_{C-F} = 34.2 Hz, C-CF_3),126.2 (s, CH_{Ar-bpy}), 124.8 (d, J_{C-F} = 21.0 Hz, CH_{Ar-ppy}), 123.0 (q, J_{C-F} = 271.7 Hz, CF_3), 115.4 (dd, J_{C-F} = 8.1, 2.9 Hz, CH_{Ar-ppy}), 115.2 (dd, J_{C-F} = 8.1, 3.0 Hz, CH_{Ar-ppy}), 100.1 (t, J_{C-F} = 27.1 Hz), 66.8 ($CH_{2-benzyl}$), 34.1 (s, CH_2), 30.8 (s, CH_2), 21.4 (s, CH_3). **^{19}F NMR** (377 MHz, Acetone-d_6) δ -63.44 (s, CF_3), -63.46 (s, CF_3), -72.43 (d, *J* = 703.2 Hz, PF_6), -104.75 (m, 2CF), -107.98 (m, 2CF). **HRMS** (ESI+) *m/z*: Calcd for $C_{45}H_{30}N_4O_2F_{10}Ir$ $[M-PF_6]^+$: 1041.1838 found 1041.1840.

Λ[Ir(dF(CF$_3$)ppy)$_2$((^{C2}CO$_2$H)dmbpy)](PF$_6$)

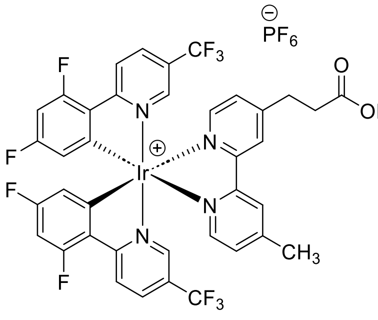

The title complex was obtained according to the procedure above (step 2) (28 mg, 99 %) from **Λ[Ir(dF(CF$_3$)ppy)$_2$((^{C2}CO$_2$Bn)dmbpy)](PF$_6$)** (1.0 equiv., 28 mg). **^{1}H NMR** (400 MHz, Acetone-*d*$_6$) δ 8.84 (s, 1H, CH$_{Ar}$), 8.81 (s, 1H, CH$_{Ar}$), 8.61 (dt, *J* = 8.8, 3.0 Hz, 2H, CH$_{Ar}$), 8.40 (dt, *J* = 8.8, 2.5 Hz, 2H, CH$_{Ar}$), 8.13 (d, *J* = 5.6 Hz, 1H, CH$_{Ar}$), 8.10 (d, *J* = 5.7 Hz, 1H, CH$_{Ar}$), 8.01 (s, 1H, CH$_{Ar}$), 7.89 (s, 1H, CH$_{Ar}$), 7.69 (d, *J* = 5.5 Hz, 1H, CH$_{Ar}$), 7.62 (d, *J* = 5.7 Hz, 1H, CH$_{Ar}$), 6.84 (ddd, *J* = 12.3, 9.3, 2.3 Hz, 2H, CH$_{Ar}$), 5.95 (ddd, *J* = 8.1, 5.4, 2.3 Hz, 2H, CH$_{Ar}$), 3.19 (t, *J* = 7.3 Hz, 2H, CH$_2$), 2.81 (t, *J* = 7.3 Hz, 2H, CH$_2$), 2.63 (s, 3H, CH$_3$). **^{13}C NMR** (101 MHz, Acetone-*d*$_6$) δ 173.3 (s, COOH), 168.6 (d, $J_{C\text{-}F}$ = 7.0 Hz, C$_{Ar\text{-}ppy}$), 165.4 (dd, $J_{C\text{-}F}$ = 258.5, 12.7 Hz, C-F), 163.3 (dd, $J_{C\text{-}F}$ = 261.8, 13.2 Hz, C-F), 157.0 (s, C$_{Ar\text{-}bpy}$), 156.7 (s, C$_{Ar\text{-}bpy}$), 156.5 (s, C$_{Ar\text{-}bpy}$), 156.4 (d, $J_{C\text{-}F}$ = 7.8 Hz, C$_{Ar\text{-}ppy}$), 154.1 (s, C$_{Ar\text{-}bpy}$), 151.8 (s, CH$_{Ar\text{-}bpy}$), 151.5 (s, CH$_{Ar\text{-}bpy}$), 147.0 (q, $J_{C\text{-}F}$ = 4.7 Hz, CH$_{Ar\text{-}ppy}$), 146.7 (q, $J_{C\text{-}F}$ = 4.8 Hz, CH$_{Ar\text{-}ppy}$), 138.1 (q, $J_{C\text{-}F}$ = 3.4 Hz, CH$_{Ar\text{-}ppy}$), 130.5 (s, CH$_{Ar\text{-}bpy}$), 129.9 (s, CH$_{Ar\text{-}bpy}$), 127.8 – 127.6 (m, C$_{Ar\text{-}ppy}$), 126.9 (s, CH$_{Ar\text{-}bpy}$), 126.3 (q, $J_{C\text{-}F}$ = 34.6 Hz, C-CF$_3$), 126.2 (q, $J_{C\text{-}F}$ = 34.6 Hz, C-CF$_3$), 126.2 (s, CH$_{Ar\text{-}bpy}$), 124.7 (d, $J_{C\text{-}F}$ = 21.0 Hz, CH$_{Ar\text{-}ppy}$), 123.0 (q, $J_{C\text{-}F}$ = 271.8 Hz, CF$_3$), 123.0 (q, $J_{C\text{-}F}$ = 271.7 Hz, CF$_3$), 115.4 (dd, $J_{C\text{-}F}$ = 7.9, 3.0 Hz, CH$_{Ar\text{-}ppy}$), 115.2 (dd, $J_{C\text{-}F}$ = 7.9, 3.0 Hz, CH$_{Ar\text{-}ppy}$), 100.1 (t, $J_{C\text{-}F}$ = 27.1 Hz, CH$_{Ar\text{-}ppy}$), 33.9 (s, CH$_2$), 30.9 (s, CH$_2$), 21.4 (s, CH$_3$). **^{19}F NMR** (377 MHz, Acetone-*d*$_6$) δ -63.47 (s, CF$_3$), -63.52 (s, CF$_3$), -72.54 (d, *J* = 708.1 Hz, PF$_6$), -104.63 – -104.99 (m, 2CF), -107.95 – -108.11 (m, 2CF). **HRMS** (ESI+) *m/z*: Calcd for C$_{38}$H$_{24}$N$_4$O$_2$F$_{10}$Ir [M-PF$_6$]$^+$: 951.1369 found 951.13698.

Δ[Ir(dF(CF$_3$)ppy)$_2$((^{C2}CO$_2$H)dmbpy)](PF$_6$)

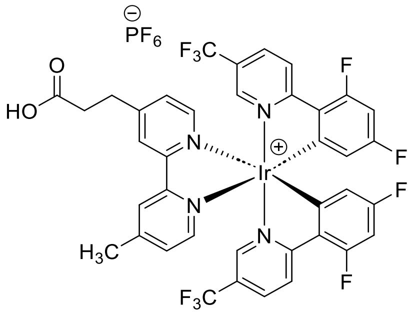

The title complex was obtained according to the procedure above (step 2) (14 mg, quant.) from **Δ[Ir(dF(CF$_3$)ppy)$_2$((^{C2}CO$_2$Bn)dmbpy)](PF$_6$)** (1.0 equiv., 14 mg). **^{1}H NMR** (400 MHz, Acetone-*d*$_6$) δ 8.83 (s, 1H, CH$_{Ar}$), 8.80 (s, 1H, CH$_{Ar}$), 8.61 (d, *J* = 8.8 Hz, 2H, CH$_{Ar}$), 8.40 (d, *J* = 8.9 Hz, 2H, CH$_{Ar}$), 8.12 (dd, *J* = 13.4, 5.6 Hz, 2H, CH$_{Ar}$), 8.01 (s, 1H, CH$_{Ar}$), 7.89 (s, 1H, CH$_{Ar}$), 7.69 (d, *J* = 5.6 Hz, 1H CH$_{Ar}$), 7.62 (d, *J* = 5.7 Hz, 1H, CH$_{Ar}$), 6.89 – 6.77 (m, CH$_{Ar}$), 5.98 – 5.88 (m, CH$_{Ar}$), 3.19 (t, *J* = 7.3 Hz, 2H, CH$_2$), 2.84 (t, *J* = 7.3 Hz, 1H, CH$_2$), 2.63 (s, 3H, CH$_3$). **^{13}C NMR** (101 MHz, Acetone-*d*$_6$) δ 173.1 (s, COOH), 168.8 – 168.5 (m, C$_{Ar\text{-}ppy}$), 165.4 (dd, $J_{C\text{-}F}$ = 258.4, 12.7 Hz, C-F), 163.3 (dd, $J_{C\text{-}F}$ = 261.7, 13.2 Hz, C-F), 156.9 (s, C$_{Ar\text{-}bpy}$), 156.7 (s, C$_{Ar\text{-}bpy}$), 156.4 (s, C$_{Ar\text{-}bpy}$), 156.4 (d, $J_{C\text{-}F}$ = 7.1 C$_{Ar\text{-}ppy}$), 156.4 (d, $J_{C\text{-}F}$ = 7.1 C$_{Ar\text{-}ppy}$),154.1 (s, C$_{Ar\text{-}bpy}$), 151.8 (s, CH$_{Ar\text{-}bpy}$), 151.5 (s,

$CH_{Ar\text{-}bpy}$), 147.0 (q, $J_{C\text{-}F}$ = 4.8 Hz, $CH_{Ar\text{-}ppy}$), 146.7 (q, $J_{C\text{-}F}$ = 4.8 Hz, $CH_{Ar\text{-}ppy}$), 138.1 (q, $J_{C\text{-}F}$ = 3.3 Hz, $CH_{Ar\text{-}ppy}$), 130.5 (s, $CH_{Ar\text{-}bpy}$), 129.9 (s, $CH_{Ar\text{-}bpy}$), 127.8 – 127.6 (m, $C_{Ar\text{-}ppy}$), 126.9 (s, $CH_{Ar\text{-}bpy}$), 126.3 (q, $J_{C\text{-}F}$ = 34.3 Hz, C-CF_3), 126.2 (q, $J_{C\text{-}F}$ = 34.5 Hz, C-CF_3), 126.2 (s, $CH_{Ar\text{-}bpy}$), 124.7 (d, $J_{C\text{-}F}$ = 20.9 Hz, $CH_{Ar\text{-}ppy}$), 123.0 (q, $J_{C\text{-}F}$ = 271.8 Hz, CF_3), 123.0 (q, $J_{C\text{-}F}$ = 271.8 Hz, CF_3), 115.4 (dd, $J_{C\text{-}F}$ = 7.9, 3.0 Hz, $CH_{Ar\text{-}ppy}$), 115.2 (dd, $J_{C\text{-}F}$ = 8.0, 2.9 Hz, $CH_{Ar\text{-}ppy}$), 100.1 (t, $J_{C\text{-}F}$ = 27.1 Hz, $CH_{Ar\text{-}ppy}$), 33.7 (s, CH_2), 30.8 (s, CH_2), 21.4 (s, CH_3). **^{19}F NMR** (377 MHz, Acetone-d_6) δ -63.46 (s, CF_3), -63.52 (s, CF_3), -72.53 (d, J = 708.4 Hz, PF_6), -104.80 (m, 2CF), -108.02 (t, J = 12.6 Hz, 2CF). **HRMS** (ESI+) *m/z*: Calcd for $C_{38}H_{24}N_4O_2F_{10}Ir$ [M-PF_6]$^+$: 951.1369 found 951.1365.

III. Oligonucleotide synthesis

1. Synthesis of serinol phosphoramidite

General procedure C

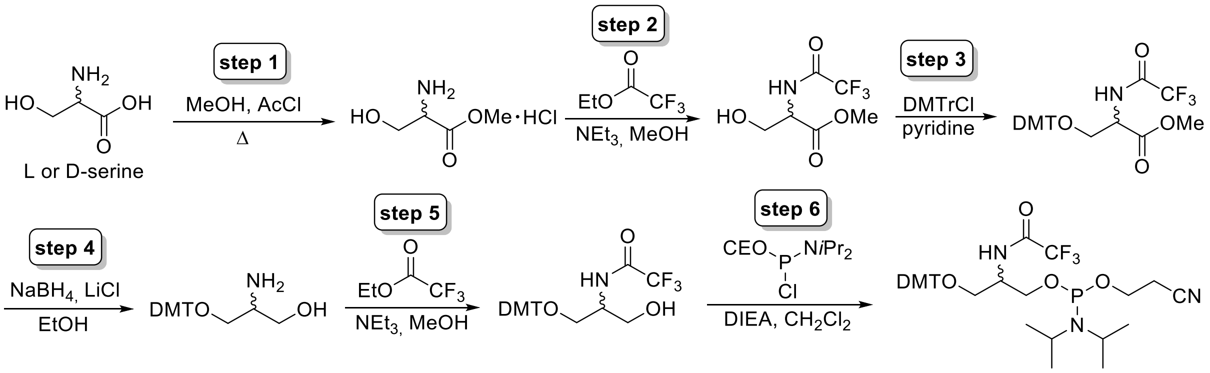

Serinol phosphoramidite was synthesized over 6 steps following the procedure described by Duchemin *et al.*[3]

⇨ Synthesis of L-serinol phosphoramidite

L-Serine Methyl Ester Hydrochloride (I)

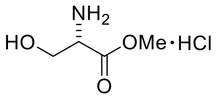

Following the step 1 of the described general procedure, title compound **I** was synthesized using L-serine (95 mmol, 1.0 equiv.) and acetyl chloride (257 mmol, 2.7 equiv.) in methanol (140 mL). The desired product was obtained as a white solid (94 mmol, 99 % yield) and used without purification. Spectroscopic data were consistent with the literature data.[3]

N-(2,2,2-Trifluoroacetyl)-L-serine methyl ester (II)

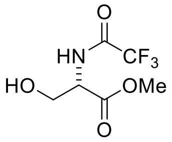

Following the step 2 of the described general procedure, title compound **II** was synthesized using **I** (12.9 mmol, 1.0 equiv.), ethyl trifluoroacetate (21.9 mmol, 1.7 equiv.) and triethylamine (32.3 mmol, 2.5 equiv.) in methanol (45 mL). The desired product was isolated by flash chromatography (gradient: cyclohexane/EtOAc 1:1) as a yellow oil (9.7 mmol, 75 % yield). Spectroscopic data were consistent with the literature data.[3]

N-(2,2,2-trifluoroacetyl)-*O*-dimethoxytrityl-L-serine methyl ester (III)

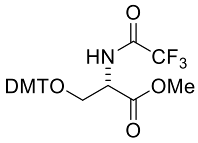

Following the step 3 of the described general procedure, title compound **III** was synthesized using **II** (9.3 mmol, 1.0 equiv.) and DMTrCl (11.2 mmol, 1.2 equiv.) in pyridine (20 mL). The desired product was isolated by flash chromatography (gradient: cyclohexane/EtOAc 7:3, 1 % NEt_3) as a pale yellow oil (9.2 mmol, 99 % yield). Spectroscopic data were consistent with the literature data.[3]

O-dimethoxytrityl-L-serinol (IV)

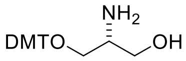

Following the step 4 of the described general procedure, title compound **IV** was synthesized using **III** (9.1 mmol, 1.0 equiv.), $NaBH_4$ (109 mmol, 12 equiv.) and LiCl (109 mmol, 12 equiv.) in ethanol (40 mL). The desired product was isolated by flash chromatography (gradient: CH_2Cl_2/MeOH 0 to 5 %, 1 % NEt_3) as a colorless foam (7.6 mmol, 84 % yield). Spectroscopic data were consistent with the literature data.[3]

N-(2,2,2-trifluoroacetyl)-*O*-dimethoxytrityl-L-serinol (V)

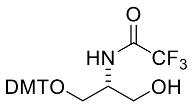

Following the step 5 of the described general procedure, title compound **V** was synthesized using **IV** (6.7 mmol, 1.0 equiv.), ethyl trifluoroacetate (7.4 mmol, 1.1 equiv.) and NEt_3 (7.4 mmol, 1.1 equiv.) in methanol (70 mL). The desired product was obtained as a colorless oil (5.7 mmol, 81 % yield) and used without purification. Spectroscopic data were consistent with the literature data.[3]

***N*-(2,2,2-trifluoroacetyl)-*O*-dimethoxytrityl-L-serinol-(2-cyanoethyl)-diisopropylphosphoramidite (VI)**

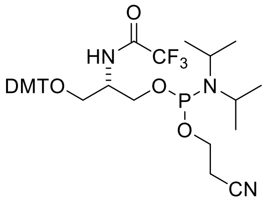

Following the step 6 of the described general procedure, title compound **VI** was synthesized using **V** (5.3 mmol, 1.0 equiv.), (2-cyanoethyl)-diisopropylchlorophosphoramidite (6.4 mmol, 1.2 equiv.) and DIEA (7.4 mmol, 1.4 equiv.) in dichloromethane (35 mL). The desired product was isolated by flash chromatography (gradient: cyclohexane/EtOAc 8:2, 1 % NEt_3) as a colorless foam (3.6 mmol, 67 % yield). Spectroscopic data were consistent with the literature data.[3]

⇨ **Synthesis of D-serinol phosphoramidite**

D-serine methyl ester hydrochloride (VII)

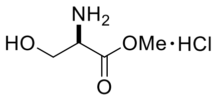

Following the step 1 of the described general procedure, title compound **VII** was synthesized using D-serine (53.9 mmol, 1.0 equiv.) and acetyl chloride (145 mmol, 2.7 equiv.) in methanol (80 mL). The desired product was obtained as a white solid (53.7 mmol, 99 % yield) and used without purification. Spectroscopic data were consistent with the literature data.[3]

***N*-(2,2,2-trifluoroacetyl)-D-serine methyl ester (VIII)**

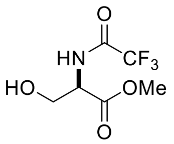

Following the step 2 of the described general procedure, title compound **VIII** was synthesized using **VII** (12.9 mmol, 1.0 equiv.), ethyl trifluoroacetate (21.9 mmol, 1.7 equiv.) and triethylamine (32.3 mmol, 2.5 equiv.) in methanol (45 mL). The desired product was isolated by flash chromatography (gradient: cyclohexane/EtOAc 1:1) as a yellow oil (8.9 mmol, 69 % yield). Spectroscopic data were consistent with the literature data.[3]

***N*-(2,2,2-trifluoroacetyl)-*O*-dimethoxytrityl-D-serine methyl ester (IX)**

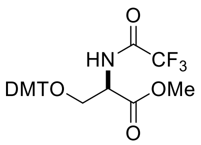

Following the step 3 of the described general procedure, title compound **IX** was synthesized using **VIII** (8.9 mmol, 1.0 equiv.) and DMTrCl (10.8 mmol, 1.2 equiv.) in pyridine (18 mL). The desired product was isolated by flash chromatography (gradient: cyclohexane/EtOAc 7:3, 1 % NEt_3) as a pale yellow oil (8.2 mmol, 92 % yield). Spectroscopic data were consistent with the literature data.[3]

_O_-dimethoxytrityl-D-serinol (X)

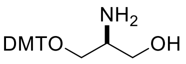

Following the step 4 of the described general procedure, title compound **X** was synthesised using **IX** (7.9 mmol, 1.0 equiv.), $NaBH_4$ (95 mmol, 12 equiv.) and LiCl (95 mmol, 12 equiv.) in ethanol (55 mL). The desired product was isolated by flash chromatography (gradient: CH_2Cl_2/MeOH 0 to 5 %, 1 % NEt_3) as a colorless foam (5.3 mmol, 68 % yield). Spectroscopic data were consistent with the literature data.[3]

_N_-(2,2,2-trifluoroacetyl)-_O_-dimethoxytrityl-D-serinol (XI)

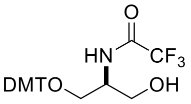

Following the step 5 of the described general procedure, title compound **XI** was synthesised using **X** (2 mmol, 1.0 equiv.), ethyl trifluoroacetate (2.2 mmol, 1.1 equiv.) and NEt_3 (2.2 mmol, 1.1 equiv.) in methanol (20 mL). The desired product was obtained as a pale yellow foam (1.7 mmol, 86 % yield) and used without purification. Spectroscopic data were consistent with the literature data.[3]

_N_-(2,2,2-trifluoroacetyl)-_O_-dimethoxytrityl-D-serinol-(2-cyanoethyl)-diisopropylphosphoramidite (XII)

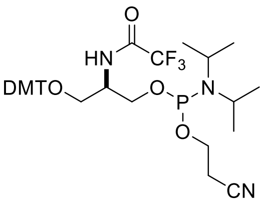

Following the step 6 of the described general procedure, title compound **XII** was synthesised using **XI** (1.7 mmol, 1.0 equiv.), (2-cyanoethyl)-diisopropylchlorophosphoramidite (2.0 mmol, 1.2 equiv.) and DIEA (2.4 mmol, 1.4 equiv.) in dichloromethane (13 mL). The desired product was isolated by flash chromatography (gradient: cyclohexane/EtOAc 8:2, 1 % NEt_3) as a colorless foam (1.5 mmol, 88 % yield). Spectroscopic data were consistent with the literature data.[3]

2. Oligonucleotide synthesis

Oligonucleotide synthesis was performed on solid support (CPG beads, 500 A). Commercially available phosphoramidite synthons dA, dT, dG and dC (rA, rU, rG and rC for RNA sequences) and synthesized serinol phosphoramidite (see III-a) were used to afford the corresponding desired ODN sequences (DMT-off synthesis, see Table S1 for reaction informations). After the supported synthesis, CPG beads were washed with H_2O (3 x 2mL), anhydrous MeCN (3 × 2 mL) and then dried by flushing compressed air. Supported oligonucleotide was percolated with a DBU solution (3 min with 2 mL, 1 mM in anhydrous MeCN) to remove cyanoethyle groups

during ARN synthesis and when TFA-protected serinol was incorporated, washed with anhydrous MeCN (3 x 10 mL) and then dried by flushing compressed air. Ammonia (30% in H_2O, 1.00 mL/µmol oligonucleotide) was added to the solid-supported oligonucleotide to cleave the support overnight at 40 °C, followed by filtration of the beads. The crude was purified by RP chromatography (using HPLC gradiant described in Table S2) or used without purification for coupling reactions. The oligonucleotides were characterized by MALDI and HPLC.

Table S1 *Standard conditions for oligonucleotide synthesis*

Step	Reaction	Reagent	Time (s)
1	Detritylation	3% TCA/CH_2Cl_2	65
2	Coupling	0.075M amidite/MeCN + 0.3M BMT/MeCN	30
2’	Coupling of serinol amidite	0.1M amidite/MeCN + 0.3M BMT/MeCN	90
3	Capping	Ac_2O/THF/Pyridine + 10% NMI/THF	20
4	Oxidation	0.1M I_2/THF/Pyridine/H_2O	15

3. Synthesis of modified oligonucleotides (Oligonucleotide – iridium complex coupling)

To a solution of acid-photocatalyst $[Ir(dF(CF_3)ppy)_2(dmbpy(^{cn}CO_2H)]^+$ in DMSO (50 equiv., 5 mg in 50 µL) was added EDC (44 equiv., 146 µL from a 30 mM solution in DMSO), HOAt (8.8 equiv., 75 µL from a 12 mM solution in DMSO) and DIEA (44 equiv., 146 µL from a 30 mM solution in DMSO). The mixture was allowed to incubate at 25°C for 15 min. An aliquot of the lyophilised serinol-oligonucleotide (100 nmol) was then diluted in MOPS-buffer (100 µL of 200 mM stock solution, pH 8.5) and the activated acid-photocatalyst solution was added to the mixture. The reaction was allowed to proceed for 1 h at 25°C under orbital agitation. After 1h, fresh solution of activated acid photocatalyst was added to the reaction mixture. 2h after the last addition of activated acid, an analytic reversed-phase HPLC was performed and if the reaction was complete, the mixture was then diluted in H_2O (~12 mL) and lyophilised to remove most of the DMSO. The crude was recovered in water (1 mL) and purified on preparative reversed-phase HPLC. The coupled PC-oligonucleotide* was characterized by MALDI and HPLC.

* *The photocatalyst $[Ir(dF(CF_3)ppy)_2(dmbpy(^{C0}CO_2H)]^+$ could not be coupled.*

4. Characterization of oligonucleotides

***Table S2** Oligonucleotides used in this study*

Name[a]	Sequence	Calcd m/z	Found m/z	Yield[d]
ODN$_1$-S$_L$[b]	5'-GCC AGC L-SerGA CCG-3'	3479.3	3478.8	51 %
ODN$_1$-S$_D$[b]	5'-GCC AGC D-SerGA CCG-3'	3479.3	3480.0	45 %
(L)ODN$_1$-S$_L$[b]	(L)5'-GCC AGC L-SerGA CCG-3'	3479.3	3479.0	34 %
(L)ODN$_1$-S$_D$[b]	(L)5'-GCC AGC D-SerGA CCG-3'	3479.3	3478.5	37 %
ODN$_2$-S$_L$[b]	5'-GAG CCA GCL-Ser GAC CGT C-3'	4715.1	4716.8	58 %
ODN$_3$-S$_L$[b]	5'-GCC AGG L-SerCA CCG-3'	3479.3	3479.4	18 %
ODN$_4$-S$_L$[b]	5'-GCC AGG L-SerGA CCG-3'	3519.3	3519.4	18 %
ODN$_5$-S$_L$[b]	5'-GCC AGC L-SerCA CCG-3'	3439.3	3439.7	13 %
ODN$_6$-S$_L$[b]	5'-GCC AGT L-SerGA CCG-3'	3494.3	3494.4	36 %
ODN$_7$-S$_L$[b]	5'-GCC AGC L-SerAA CCG-3'	3463.3	3463.9	30 %
ODN$_8$-S$_L$[b]	5'-GCC AGA L-SerTA CCG-3'	3478.3	3478.6	42 %
ODN$_{C1}$[b]	5'-CGG TCT GCT GGC-3'	3652.4	3653.5	42 %
ODN$_{C2}$[b]	5'-CGG TCA GCT GGC-3'	3661.4	3662.4	38 %
ODN$_{C3}$[b]	5'-CGG TCG GCT GGC-3'	3677.4	3678.2	40 %
ODN$_{C4}$[b]	5'-CGG TCC GCT GGC-3'	3637.4	3637.9	31 %
ODN$_{C5}$[b]	5'-CGG TCC$_3$ GCT GGC-3'	3486.3	3485.8	47 %
ORN$_{C6}$[b]	(r)5'-CGG TCU GCT GGC-3'	3802.3	3802.2	28 %
ORN$_{C7}$[b]	(r)5'-CGG TCC GCT GGC-3'	3801.3	3802.0	12 %
ORN$_{C8}$[b]	(r)5'-CGG TCA GCT GGC-3'	3825.3	3825.6	18 %
ORN$_{C9}$[b]	(r)5'-CGG TCG GCT GGC-3'	3841.3	3841.1	27 %
ODN$_{C10}$[b]	5'-GAC GGT CTG CTG GCT C-3'	4888.2	4888.7	37 %
ODN$_{C11}$[b]	5'-CGG TGC CCT GGC-3'	3637.4	3637.9	28 %
ODN$_{C12}$[b]	5'-CGG TCC CCT GGC-3'	3597.4	3597.5	33 %
ODN$_{C13}$[b]	5'-CGG TGC GCT GGC-3'	3677.4	3677.4	24 %
ODN$_{C14}$[b]	5'-CGG TCC ACT GGC-3'	3621.4	3621.5	43 %
ODN$_{C15}$[b]	5'-CGG TTC GCT GGC-3'	3652.4	3652.2	40 %
ODN$_{C16}$[b]	5'-CGG TAC TCT GGC-3'	3636.4	3636.0	43 %
ODN$_{14}$[b]	5'-GCC AGC GGA CCG-3'	3655.4	3655.0	38 %
(L)ODN$_{C1}$[b]	(L)5'-CGG TCT GCT GGC-3'	3652.4	3652.9	38 %
(L)ODN$_{C4}$[b]	(L)5'-CGG TCC GCT GGC-3'	3637.4	3637.4	47 %
ODN$_1$-S$_L$-[Ir][c]	5'-GCC AGC L-Ser-[Ir]GA CCG-3'	4412.1	4412.1	49 %
ODN$_1$-S$_D$-[Ir][c]	5'-GCC AGC D-Ser-[Ir]GA CCG-3'	4412.1	4412.8	10 %
ODN$_1$-S$_L$-[Ir(C$_3$)][c]	5'-GCC AGC L-Ser-[Ir(C$_3$)]GA CCG-3'	4426.1	4424.2	20 %
ODN$_1$-S$_L$-[ΔIr][c]	5'-GCC AGC L-Ser-[ΔIr]GA CCG-3'	4412.1	4413.1	32 %
ODN$_1$-S$_L$-[ΛIr][c]	5'-GCC AGC L-Ser-[ΛIr]GA CCG-3'	4412.1	4412.6	25 %
(L)ODN$_1$-S$_D$-[ΛIr][c]	(L)5'-GCC AGC D-Ser-[ΛIr]GA CCG-3'	4412.1	4412.7	50 %
ODN$_2$-S$_L$-[Ir][c]	5'-GAG CCA GCL-Ser-[Ir] GAC CGT C-3'	5647.9	5647.6	7 %
ODN$_3$-S$_L$-[Ir][c]	5'-GCC AGG L-Ser-[Ir]CA CCG-3'	4412.1	4412.3	34 %
ODN$_4$-S$_L$-[Ir][c]	5'-GCC AGG L-Ser-[Ir]GA CCG-3'	4452.1	4452.4	41 %
ODN$_5$-S$_L$-[Ir][c]	5'-GCC AGC L-Ser-[Ir]CA CCG-3'	4372.1	4371.9	48 %
ODN$_6$-S$_L$-[Ir][c]	5'-GCC AGT L-Ser-[Ir]GA CCG-3'	4427.1	4427.8	75 %
ODN$_7$-S$_L$-[Ir][c]	5'-GCC AGC L-Ser-[Ir]AA CCG-3'	4396.1	4396.2	68 %
ODN$_8$-S$_L$-[Ir][c]	5'-GCC AGA L-Ser-[Ir]TA CCG-3'	4411.1	4411.4	73 %

[a]S = serinol. [b]HPLC Gradient: H_2O/MeCN 100:0 to 76:24 in 20 minutes, 50 mM TEAAc. [c]HPLC Gradient: H_2O/MeCN 84:16 to 40:60 in 20 minutes, 50 mM TEAAc. [d]Isolated yields.

MALDI-TOF analysis and HPLC profile of ODN$_1$-S$_L$

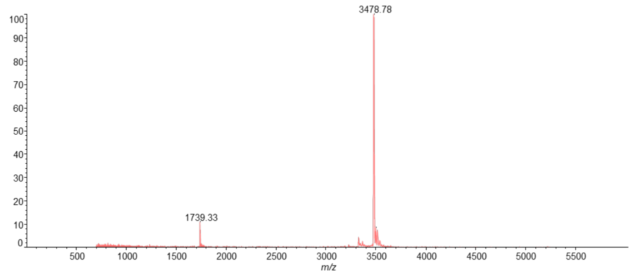

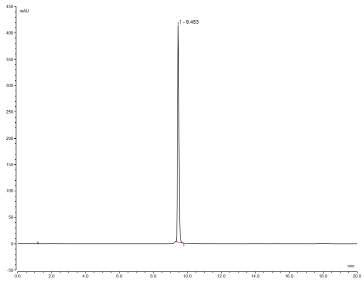

MALDI-TOF analysis and HPLC profile of ODN$_1$-S$_D$

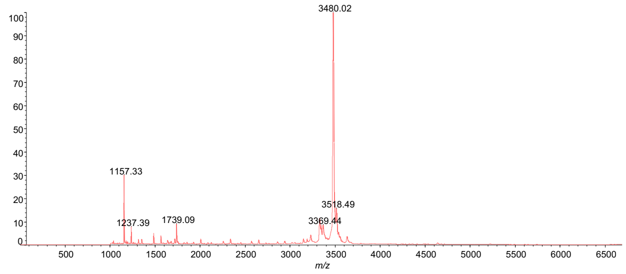

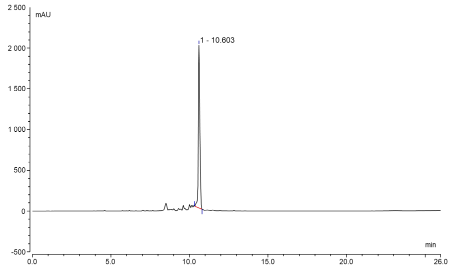

MALDI-TOF analysis and HPLC profile of (L)ODN$_1$-S$_L$

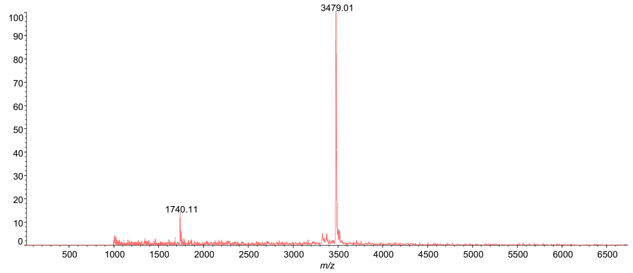

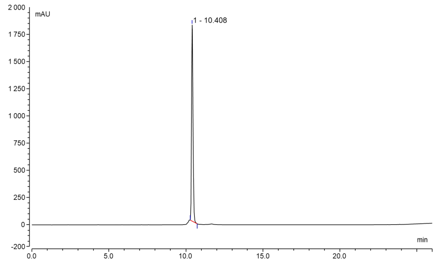

MALDI-TOF analysis and HPLC profile of (L)ODN$_1$-S$_D$

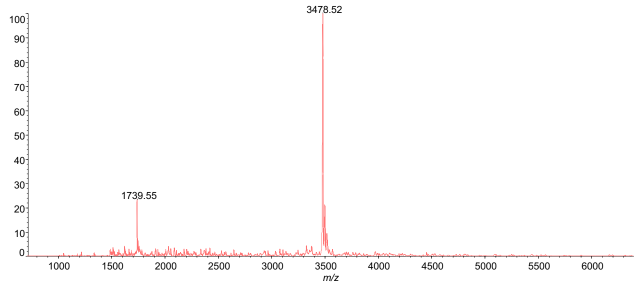

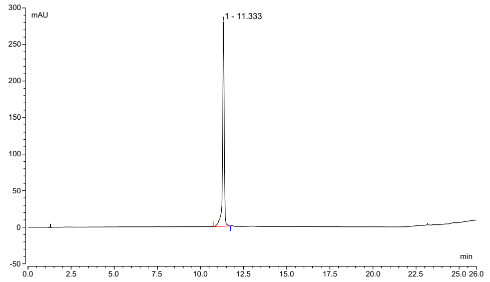

MALDI-TOF analysis and HPLC profile of ODN$_2$-S$_L$

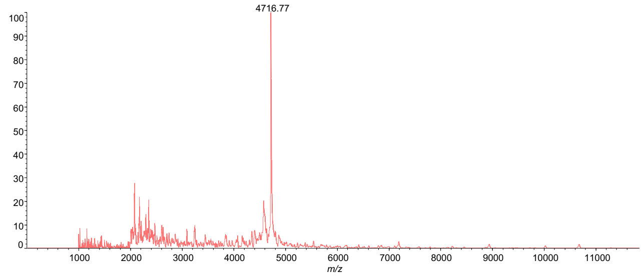

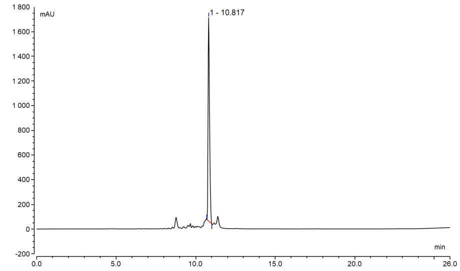

MALDI-TOF analysis and HPLC profile of ODN$_3$-S$_L$

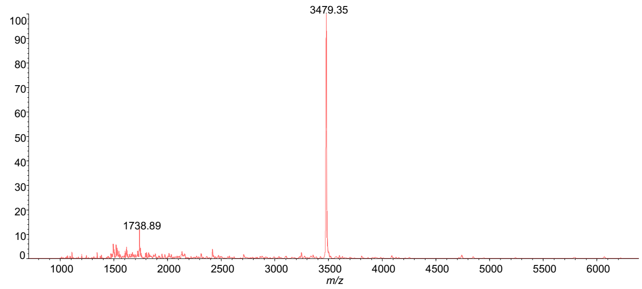

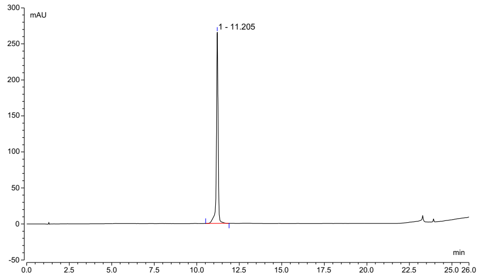

MALDI-TOF analysis and HPLC profile of ODN$_4$-S$_L$

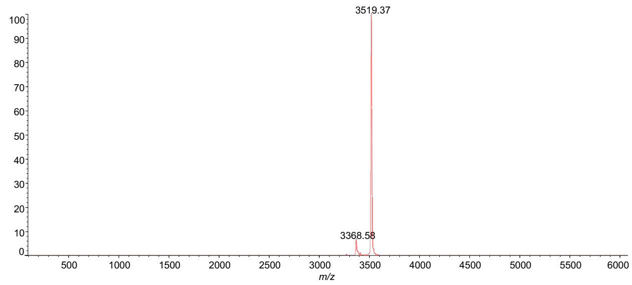

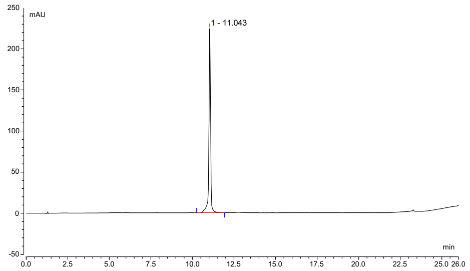

MALDI-TOF analysis and HPLC profile of ODN$_5$-S$_L$

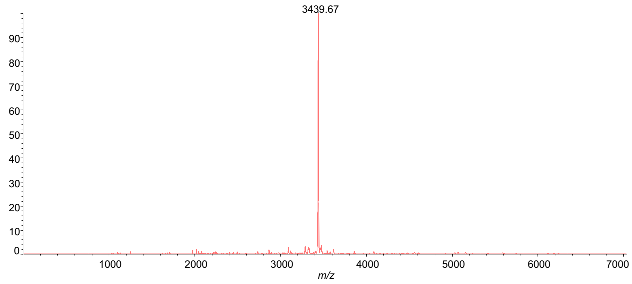

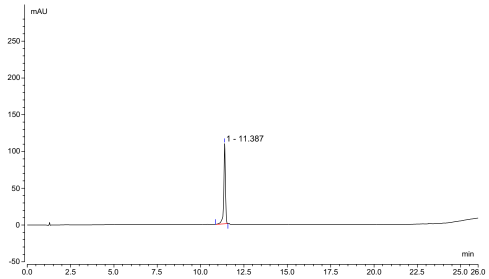

MALDI-TOF analysis and HPLC profile of ODN$_6$-S$_L$

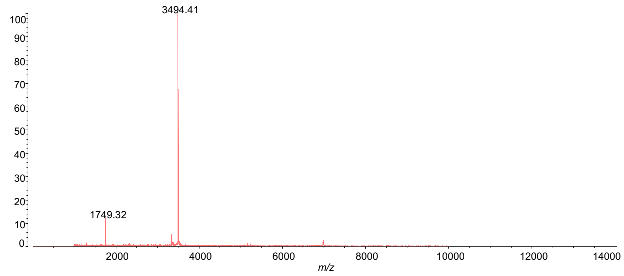

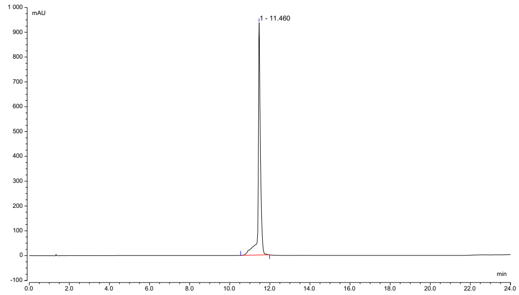

MALDI-TOF analysis and HPLC profile of ODN$_7$-S$_L$

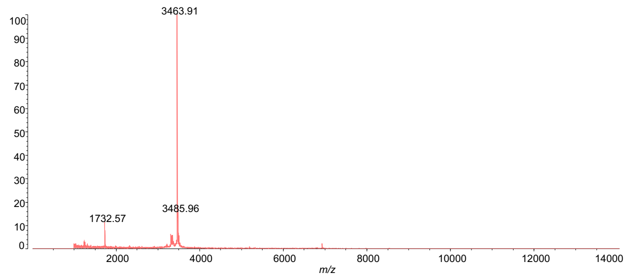

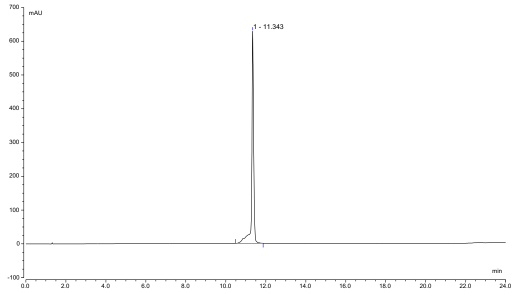

MALDI-TOF analysis and HPLC profile of ODN$_8$-S$_L$

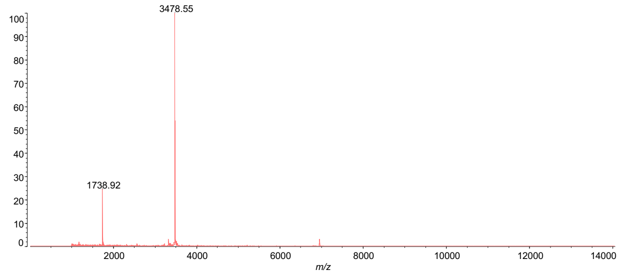

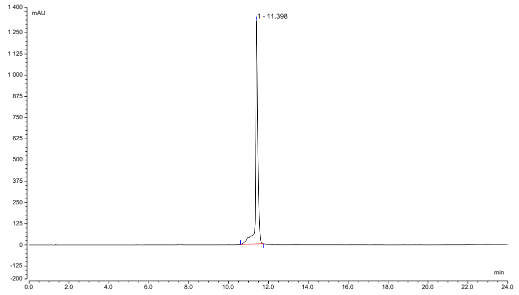

MALDI-TOF analysis and HPLC profile of ODN$_{C1}$

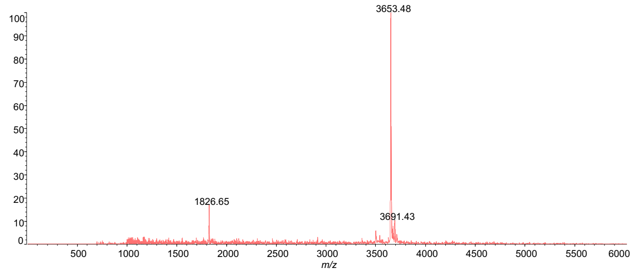

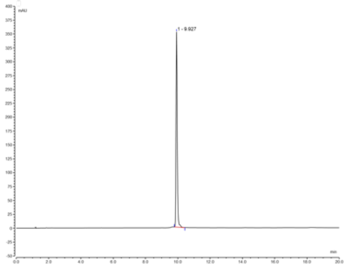

MALDI-TOF analysis and HPLC profile of ODN$_{C2}$

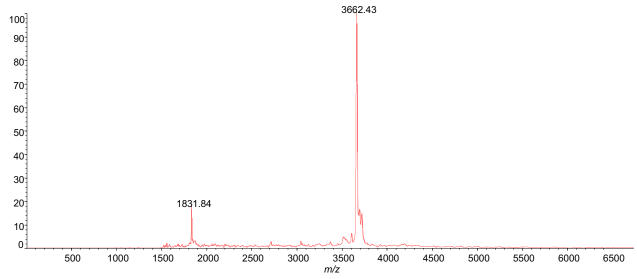

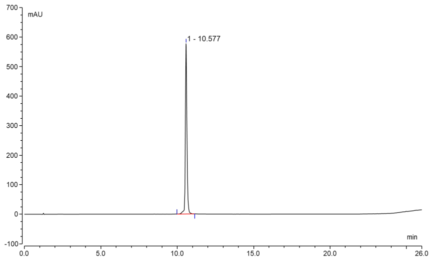

MALDI-TOF analysis and HPLC profile of ODN$_{C3}$

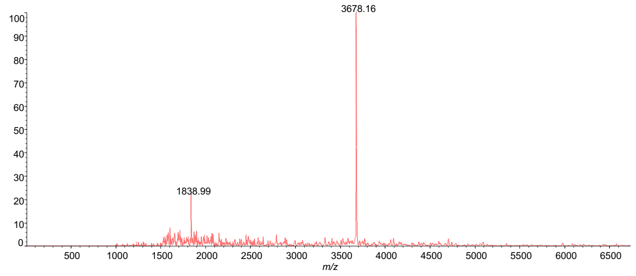

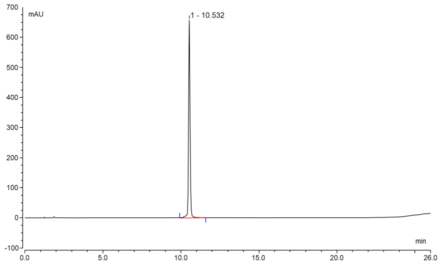

MALDI-TOF analysis and HPLC profile of ODN$_{C4}$

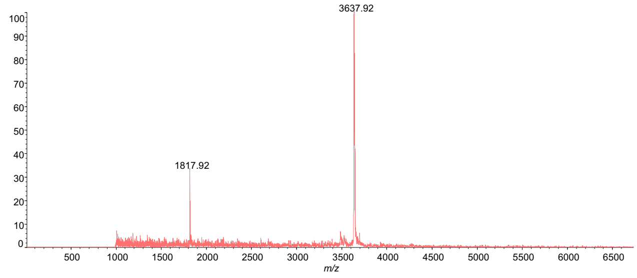

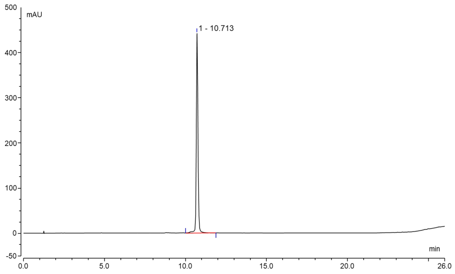

MALDI-TOF analysis and HPLC profile of ODN$_{C5}$

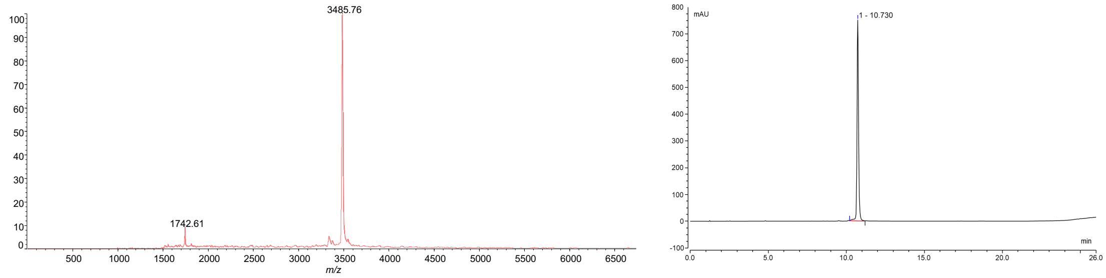

MALDI-TOF analysis and HPLC profile of ORN$_{C6}$

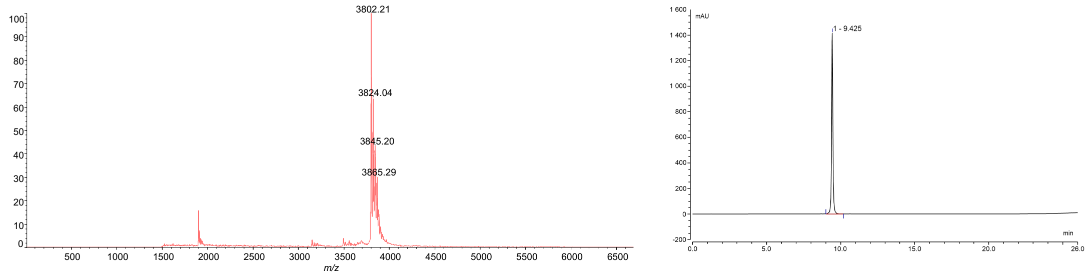

MALDI-TOF analysis and HPLC profile of ORN$_{C7}$

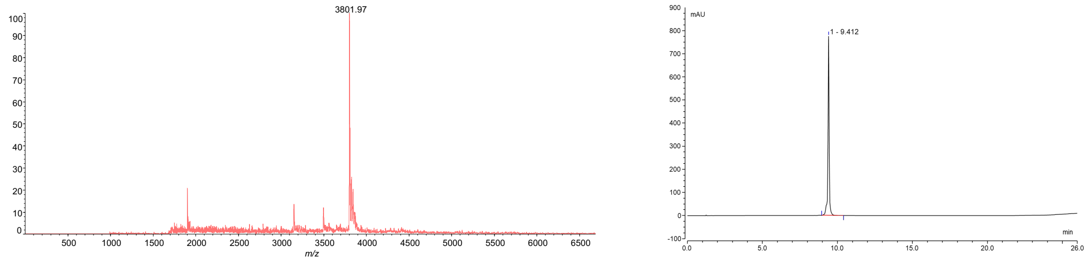

MALDI-TOF analysis and HPLC profile of ORN$_{C8}$

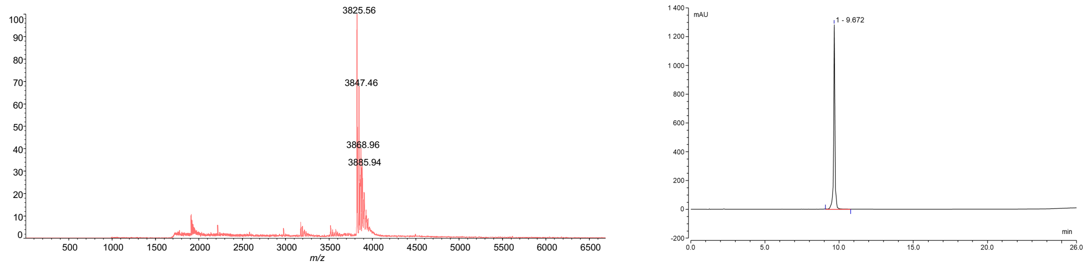

MALDI-TOF analysis and HPLC profile of ORN$_{C9}$

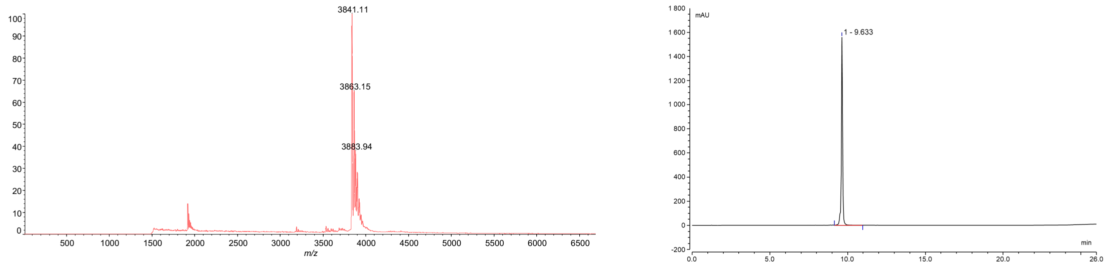

MALDI-TOF analysis and HPLC profile of ODN_{C10}

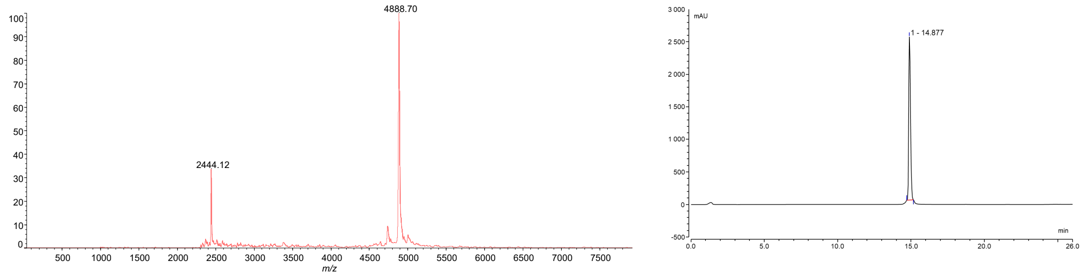

MALDI-TOF analysis and HPLC profile of ODN_{C11}

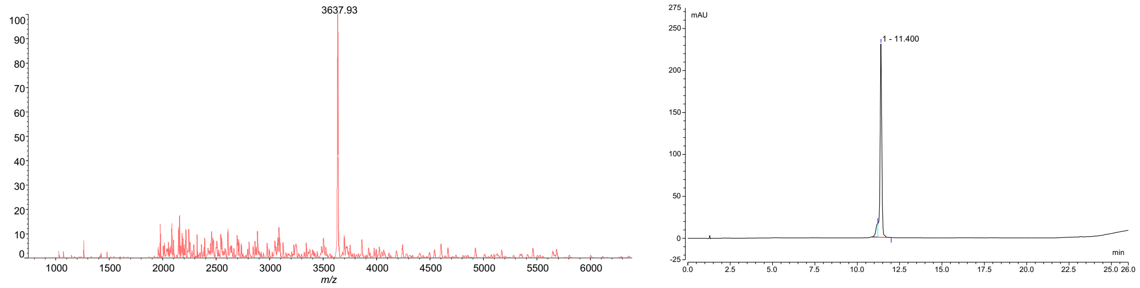

MALDI-TOF analysis and HPLC profile of ODN_{C12}

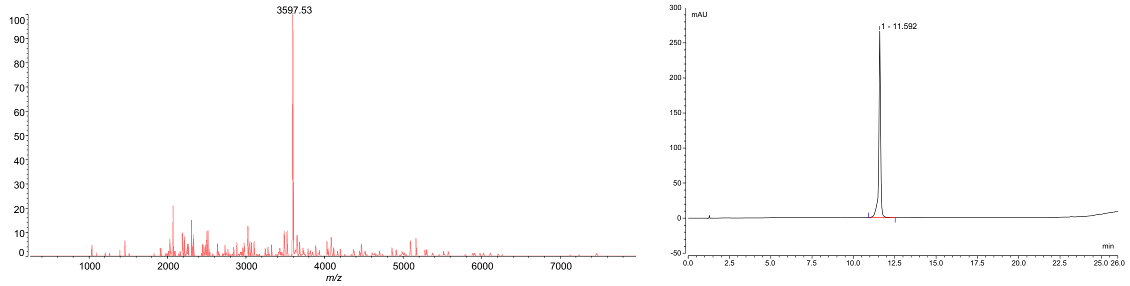

MALDI-TOF analysis and HPLC profile of ODN_{C13}

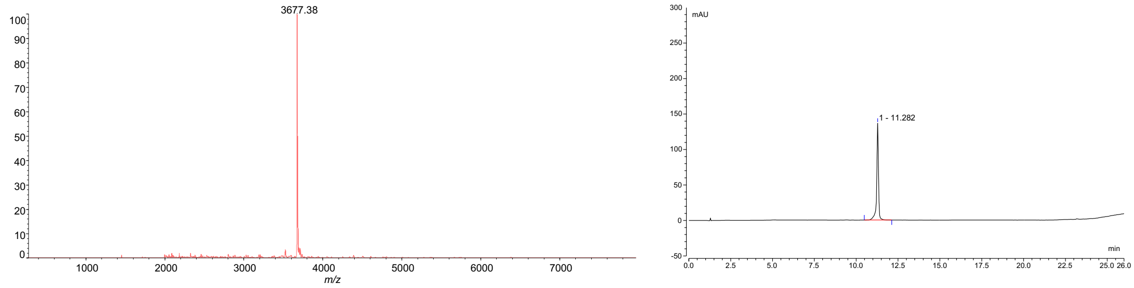

MALDI-TOF analysis and HPLC profile of ODN_{C14}

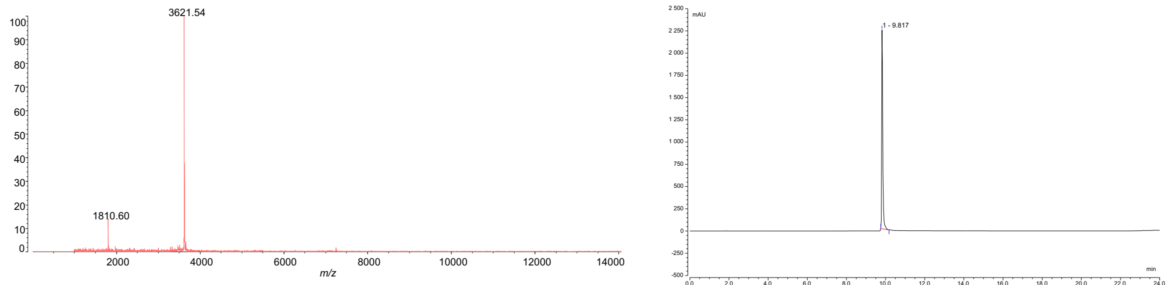

MALDI-TOF analysis and HPLC profile of ODN$_{C15}$

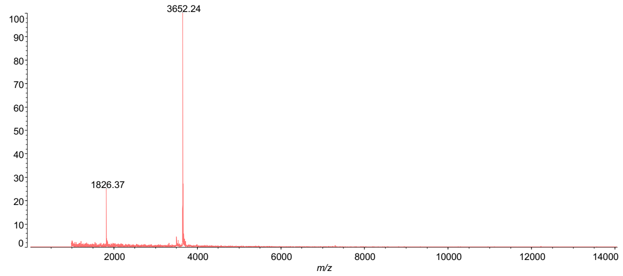

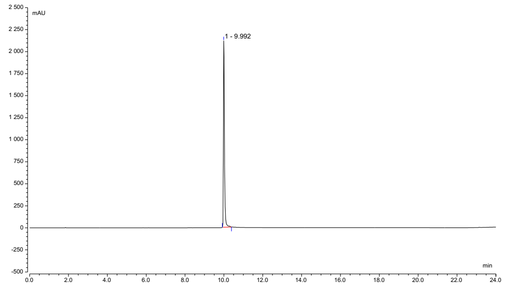

MALDI-TOF analysis and HPLC profile of ODN$_{C16}$

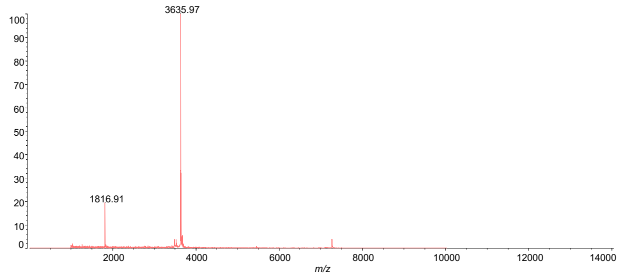

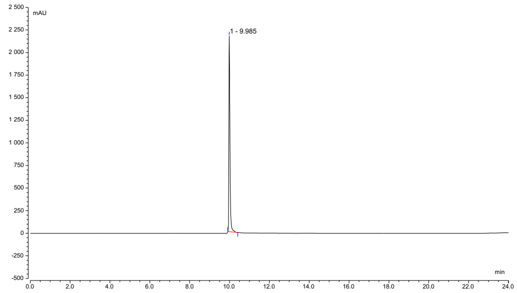

MALDI-TOF analysis and HPLC profile of ODN$_{14}$

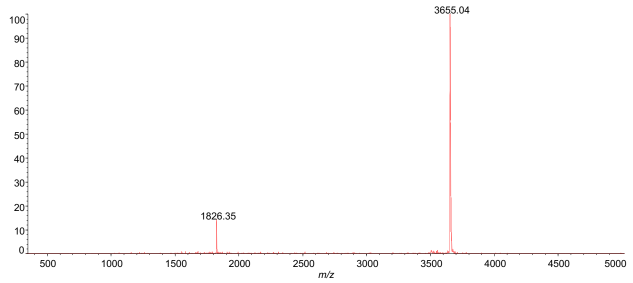

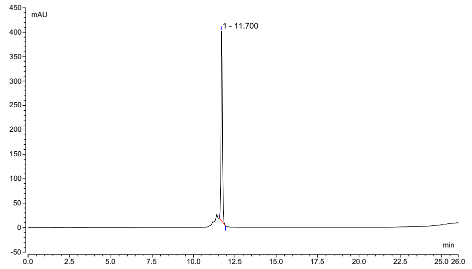

MALDI-TOF analysis and HPLC profile of (L)ODN$_{C1}$

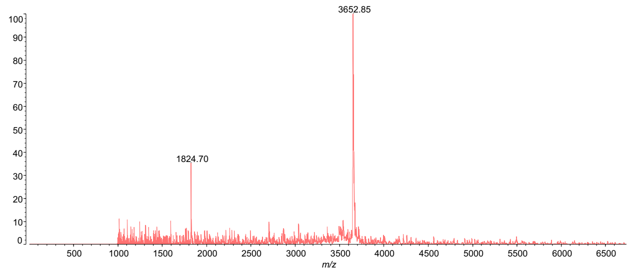

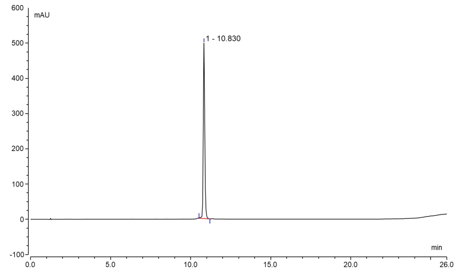

MALDI-TOF analysis and HPLC profile of (L)ODN$_{C4}$

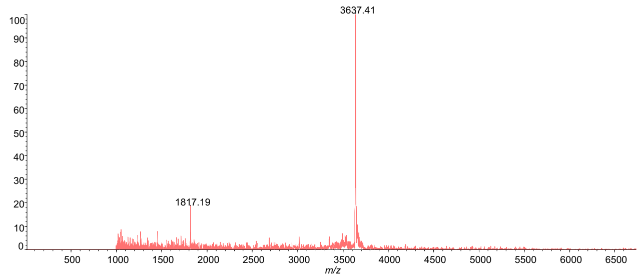

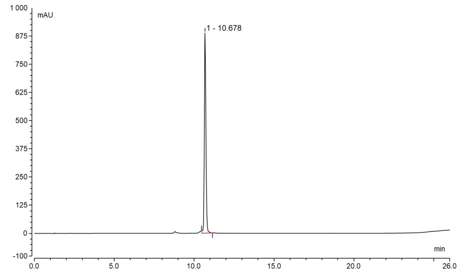

MALDI-TOF analysis and HPLC profile of ODN$_1$-S$_L$-[Ir]

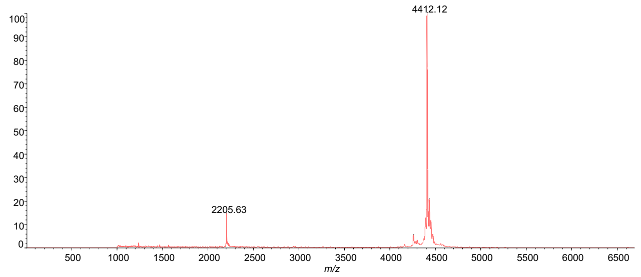

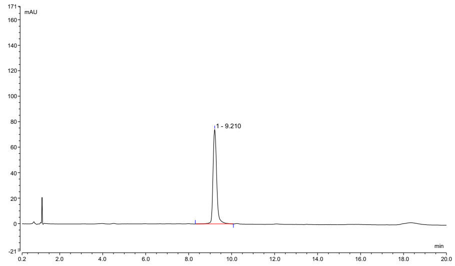

MALDI-TOF analysis and HPLC profile of ODN$_1$-S$_D$-[Ir]

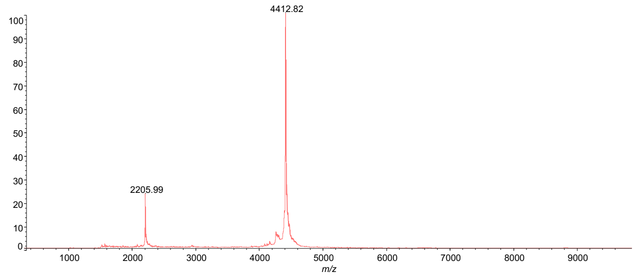

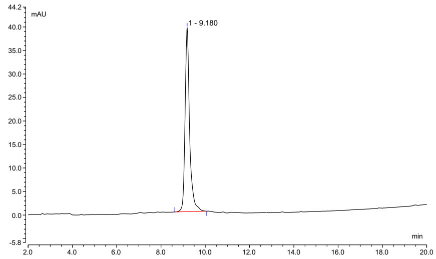

MALDI-TOF analysis and HPLC profile of ODN$_1$-S$_L$-[Ir(C$_3$)]

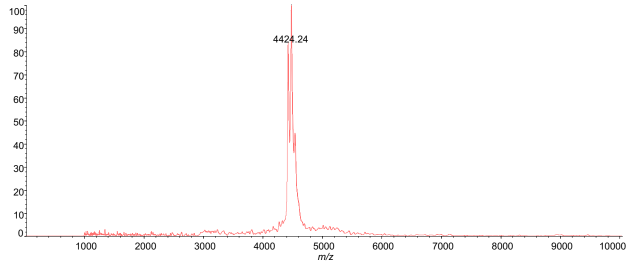

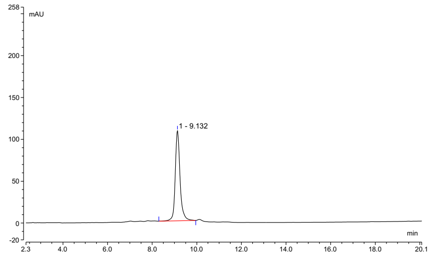

MALDI-TOF analysis and HPLC profile of ODN$_1$-S$_L$-[ΔIr]

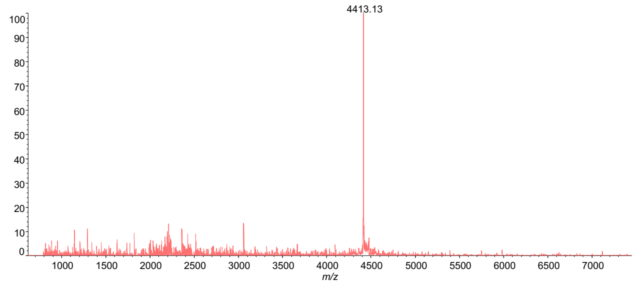

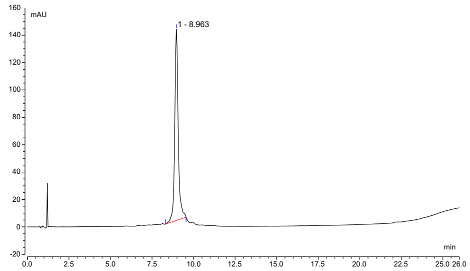

MALDI-TOF analysis and HPLC profile of ODN$_1$-S$_L$-[ΛIr]

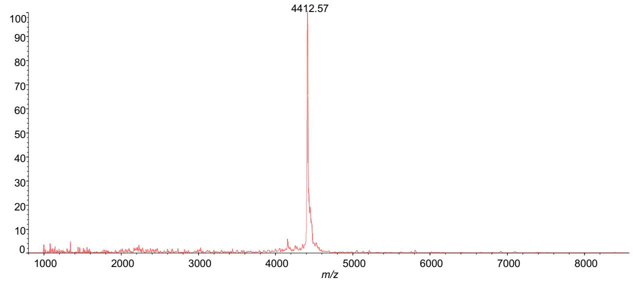

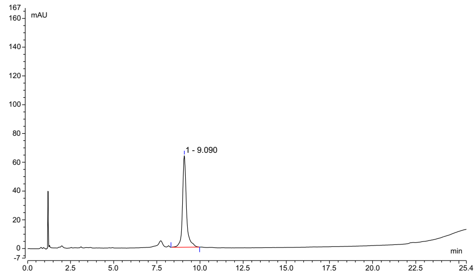

MALDI-TOF analysis and HPLC profile of (L)ODN$_1$-S$_D$-[ΛIr]

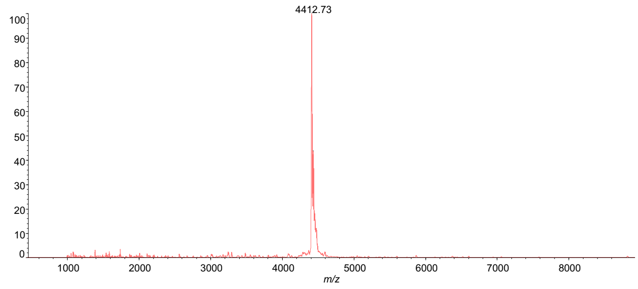

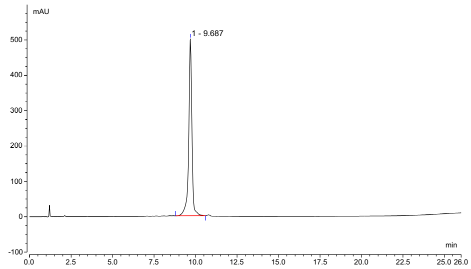

MALDI-TOF analysis and HPLC profile of ODN$_2$-S$_L$-[Ir]

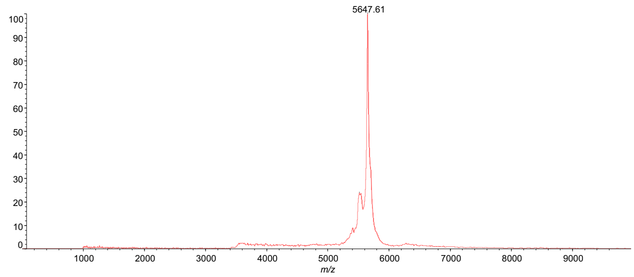

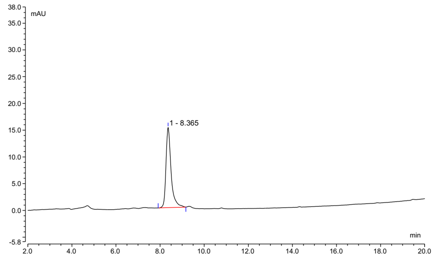

MALDI-TOF analysis and HPLC profile of ODN$_3$-S$_L$-[Ir]

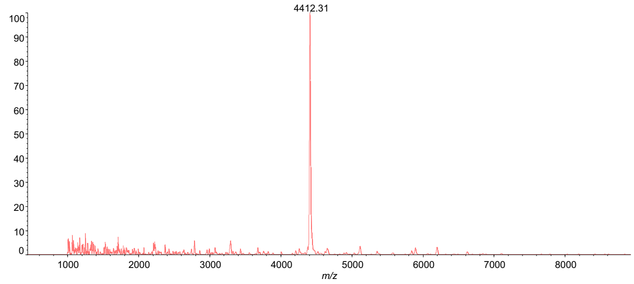

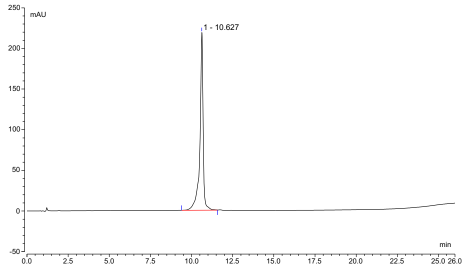

MALDI-TOF analysis and HPLC profile of ODN$_4$-S$_L$-[Ir]

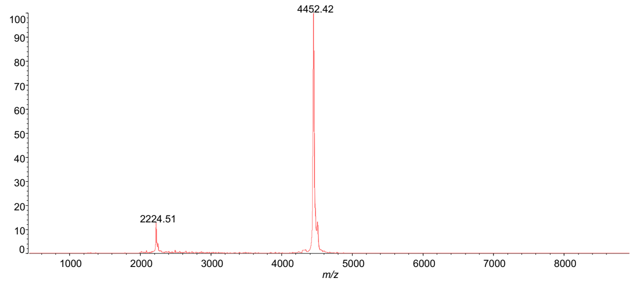

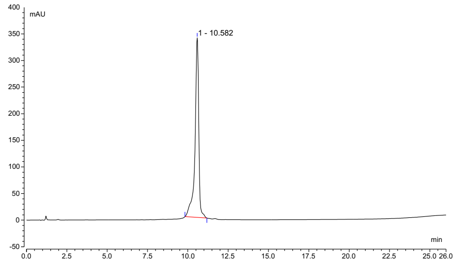

MALDI-TOF analysis and HPLC profile of ODN$_5$-S$_L$-[Ir]

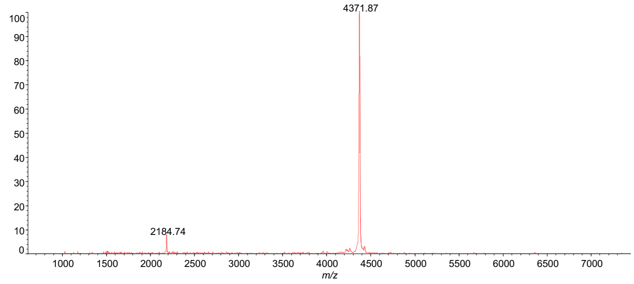

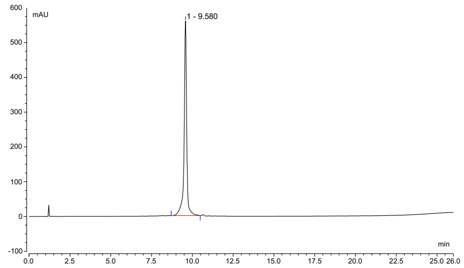

MALDI-TOF analysis and HPLC profile of ODN$_6$-S$_L$-[Ir]

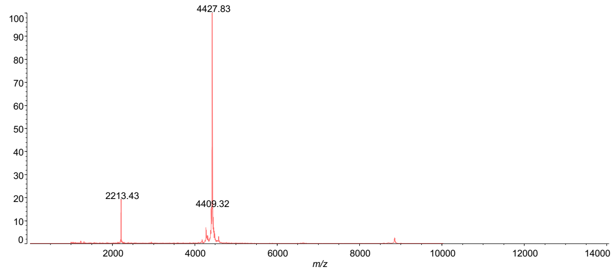

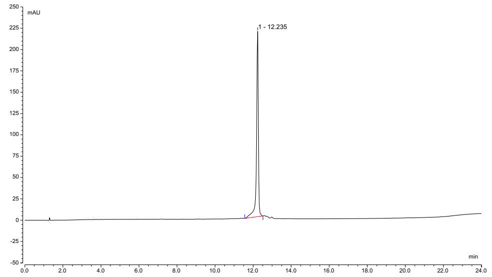

MALDI-TOF analysis and HPLC profile of ODN$_7$-S$_L$-[Ir]

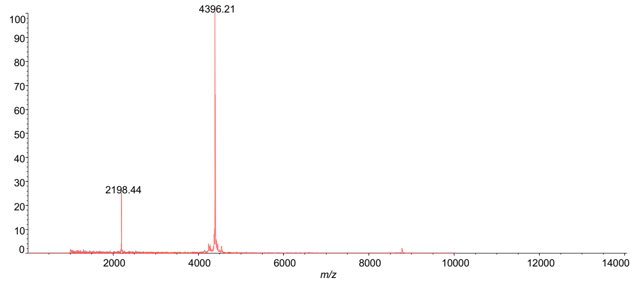

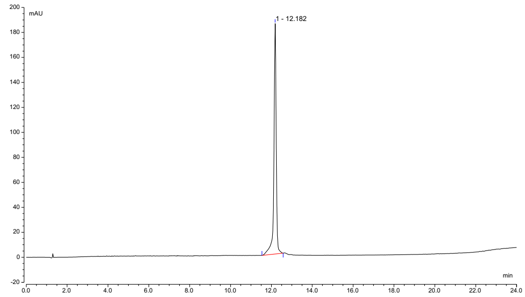

MALDI-TOF analysis and HPLC profile of ODN$_8$-S$_L$-[Ir]

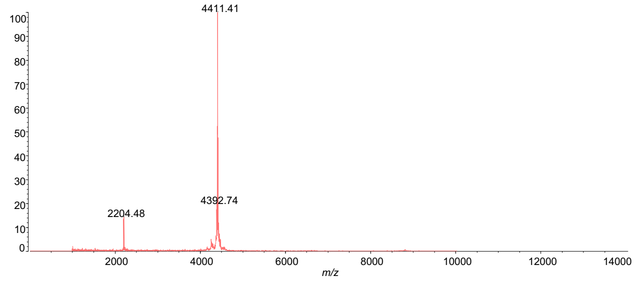

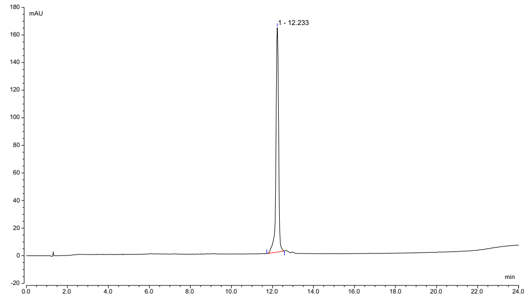

Thermal denaturation spectra of duplexes

Tm spectra were obtained on an Agilent Cary 3500 Multicell UV-Vis spectrophotometer as described in the General informations.

***Table S3** Tm of oligonucleotides used in this study*

Entry	Duplex	*Tm* (°C)	Entry	Duplex	*Tm* (°C)
1	ODN$_{14}$ / ODN$_{C4}$	61.5	9	ODN$_{1}$-S$_{L}$-[Ir] / ORN$_{C6}$	45.7
2	ODN$_{1}$-S$_{L}$ / ODN$_{C4}$	38.4	10	ODN$_{1}$-S$_{L}$-[Ir] / ORN$_{C7}$	44.5
3	ODN$_{1}$-S$_{L}$-[Ir] / ODN$_{C4}$	44.5	11	ODN$_{1}$-S$_{L}$-[Ir] / ORN$_{C8}$	47.6
4	ODN$_{1}$-S$_{D}$-[Ir] / ODN$_{C4}$	44.3	12	ODN$_{1}$-S$_{L}$-[Ir] / ORN$_{C9}$	48.5
5	ODN$_{1}$-S$_{L}$-[Ir] / ODN$_{C1}$	45.3	13	ODN$_{1}$-S$_{L}$-[ΔIr] / ODN$_{C4}$	45.2
6	ODN$_{1}$-S$_{L}$-[Ir] / ODN$_{C2}$	46.2	14	ODN$_{1}$-S$_{L}$-[ΛIr] / ODN$_{C4}$	44.4
7	ODN$_{1}$-S$_{L}$-[Ir] / ODN$_{C3}$	46.9	15	(L)ODN$_{1}$-S$_{D}$-[ΛIr] / ODN$_{C4}$	44.7
8	ODN$_{1}$-S$_{L}$-[Ir] / ODN$_{C5}$	44.5			

Thermal denaturation and first derivative analysis of ODN$_{14}$ / ODN$_{C4}$ (1)

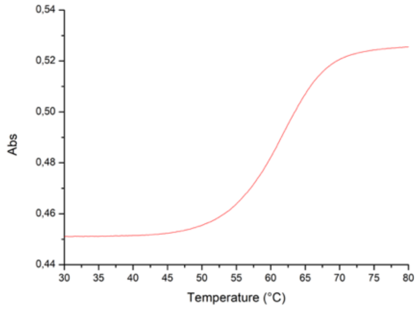

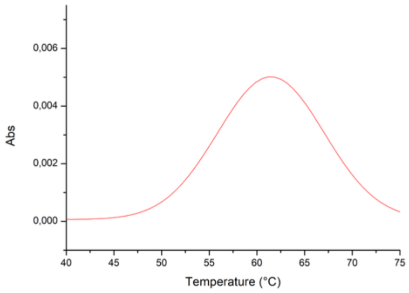

Thermal denaturation and first derivative analysis of ODN$_{1}$-S$_{L}$ / ODN$_{C4}$ (2)

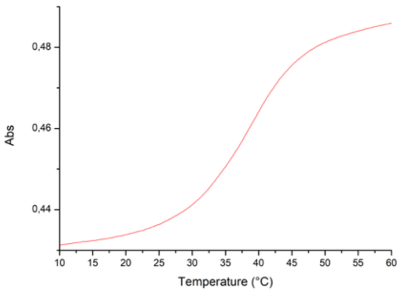

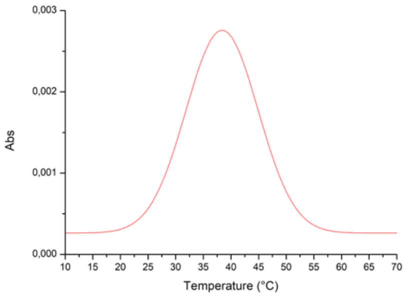

Thermal denaturation and first derivative analysis of ODN$_{1}$-S$_{L}$-[Ir] / ODN$_{C4}$ (3)

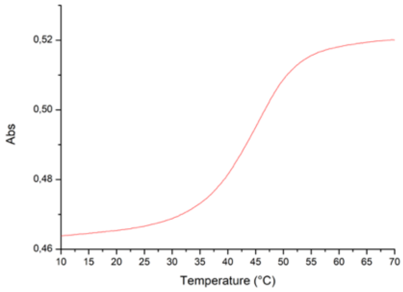

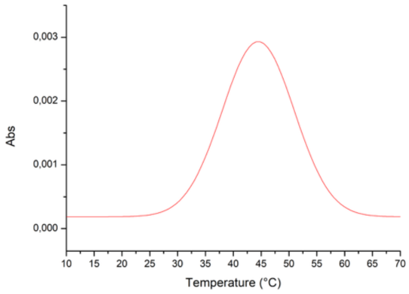

Thermal denaturation and first derivative analysis of ODN$_1$-S$_D$-[Ir] / ODN$_{C4}$ (4)

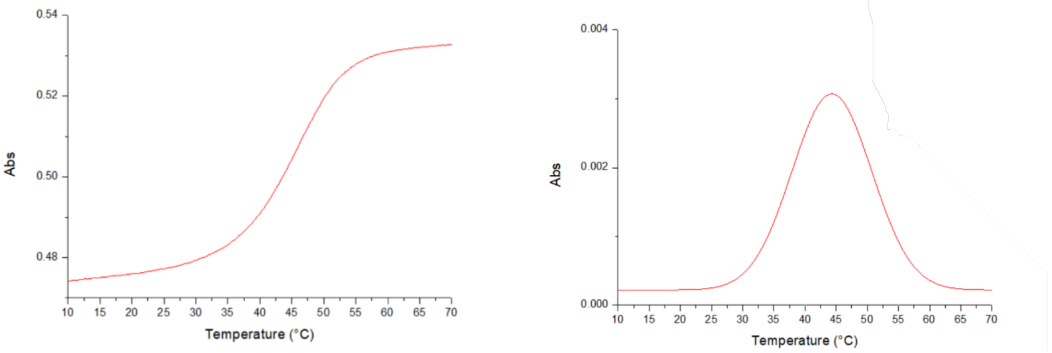

Thermal denaturation and first derivative analysis of ODN$_1$-S$_L$-[Ir] / ODN$_{C1}$ (5)

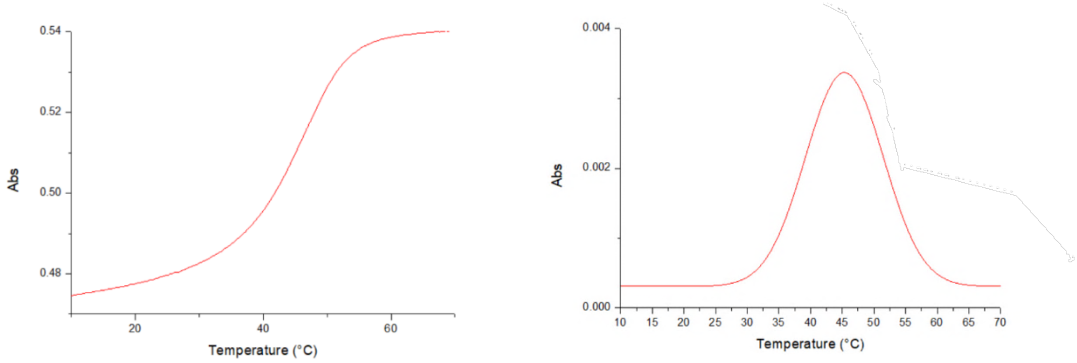

Thermal denaturation and first derivative analysis of ODN$_1$-S$_L$-[Ir] / ODN$_{C2}$ (6)

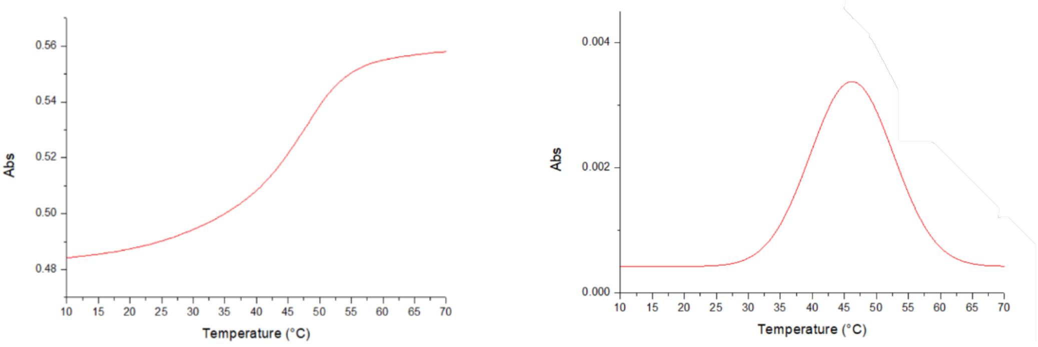

Thermal denaturation and first derivative analysis of ODN$_1$-S$_L$-[Ir] / ODN$_{C3}$ (7)

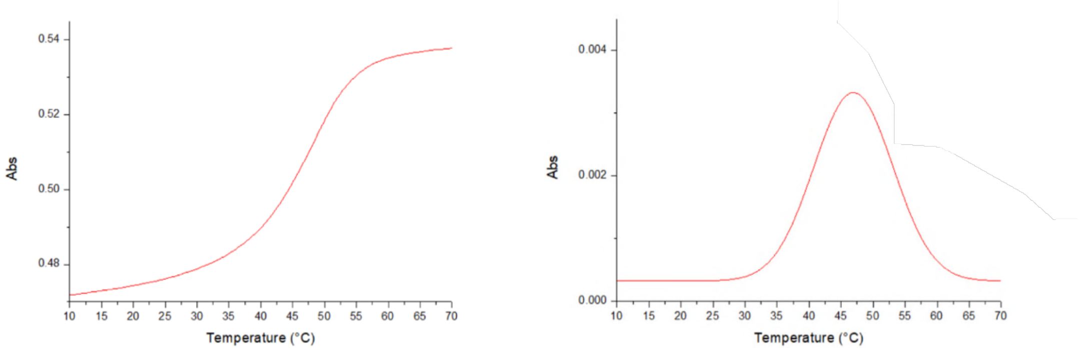

Thermal denaturation and first derivative analysis of ODN$_1$-S$_L$-[Ir] / ODN$_{C5}$ (8)

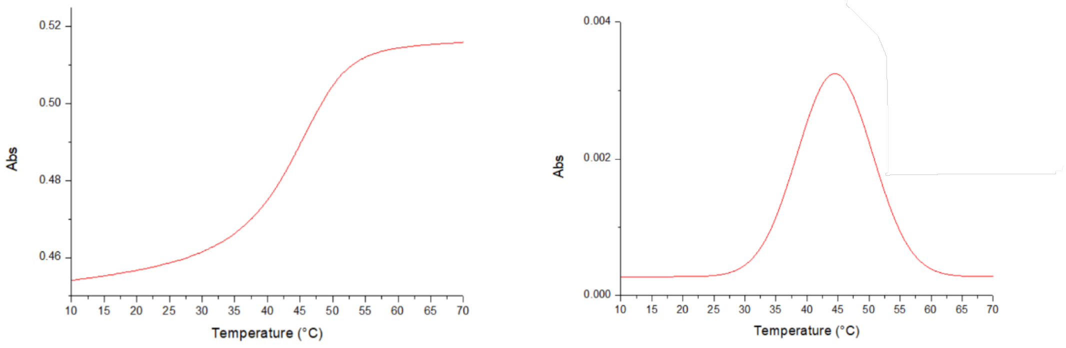

Thermal denaturation and first derivative analysis of ODN$_1$-S$_L$-[Ir] / ORN$_{C6}$ (9)

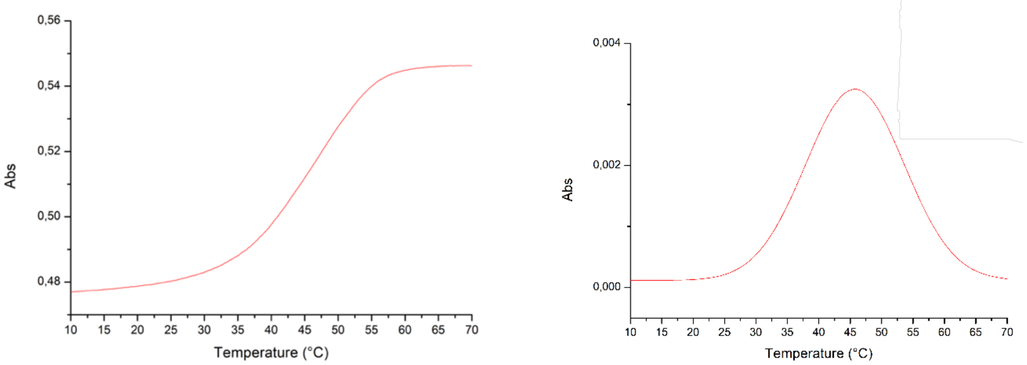

Thermal denaturation and first derivative analysis of ODN$_1$-S$_L$-[Ir]$_{C2}$ / ORN$_{C7}$ (10)

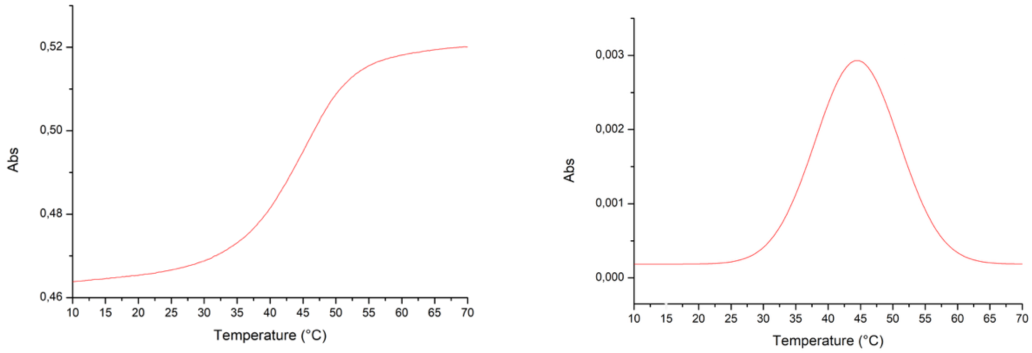

Thermal denaturation and first derivative analysis of ODN$_1$-S$_L$-[Ir]$_{C2}$ / ORN$_{C8}$ (11)

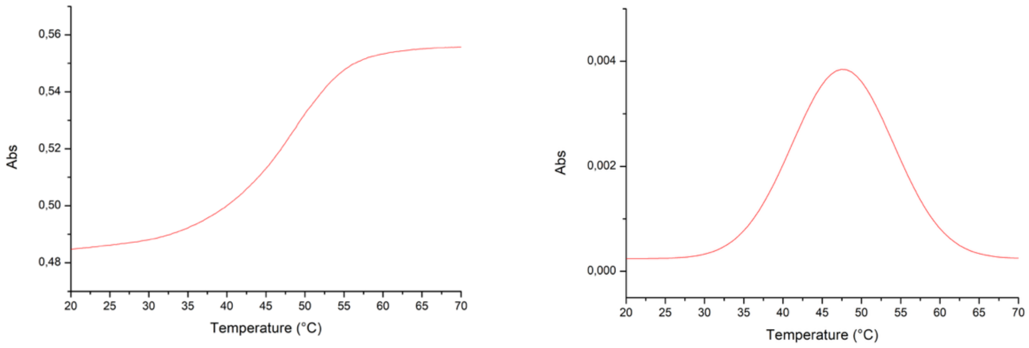

Thermal denaturation and first derivative analysis of ODN$_1$-S$_L$-[Ir]$_{C2}$ / ORN$_{C9}$ (12)

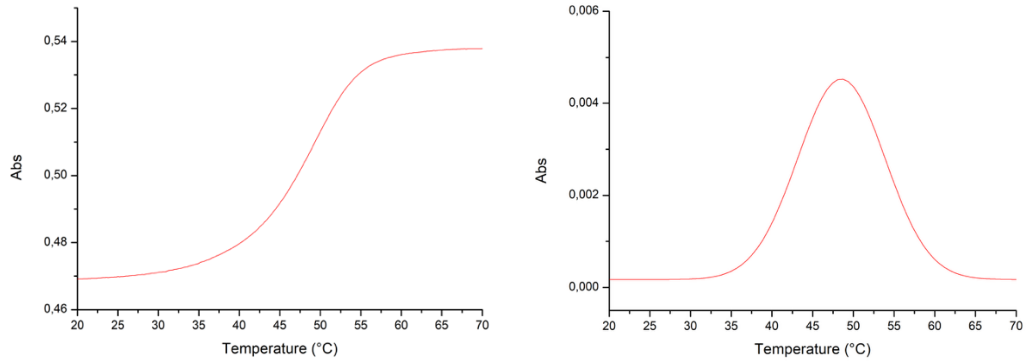

Thermal denaturation and first derivative analysis of ODN$_1$-S$_L$-[ΔIr] / ODN$_{C4}$ (13)

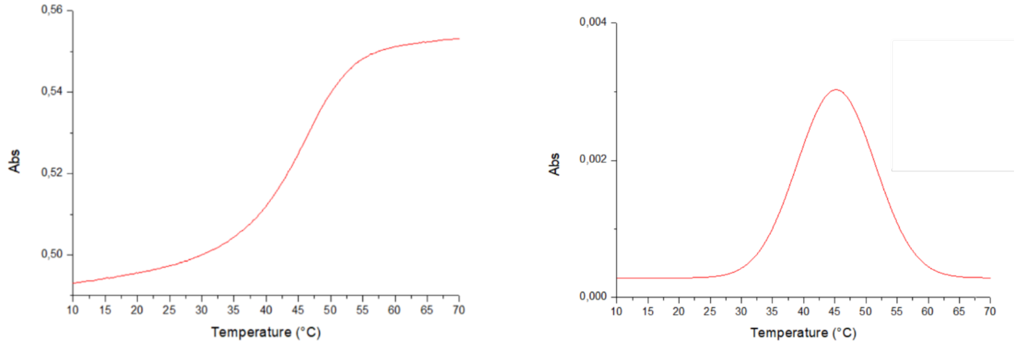

Thermal denaturation and first derivative analysis of ODN$_1$-S$_L$-[ΛIr] / ODN$_{C4}$ (14)

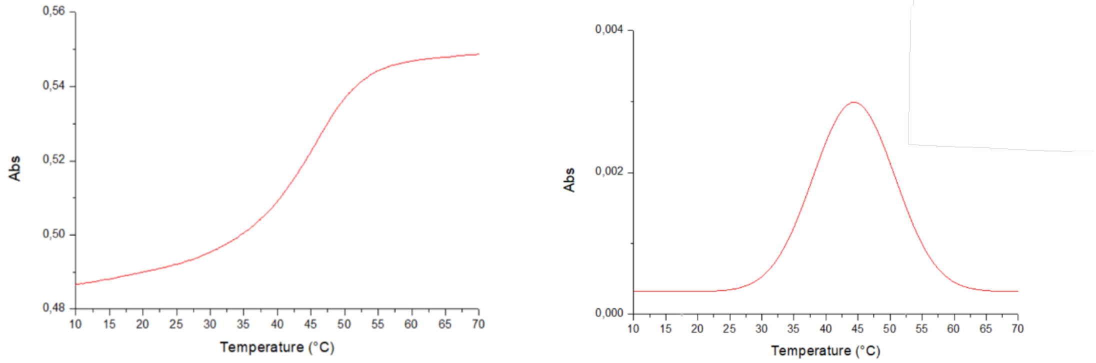

Thermal denaturation and first derivative analysis of (L)ODN$_1$-S$_D$-[ΛIr] / (L)ODN$_{C4}$ (15)

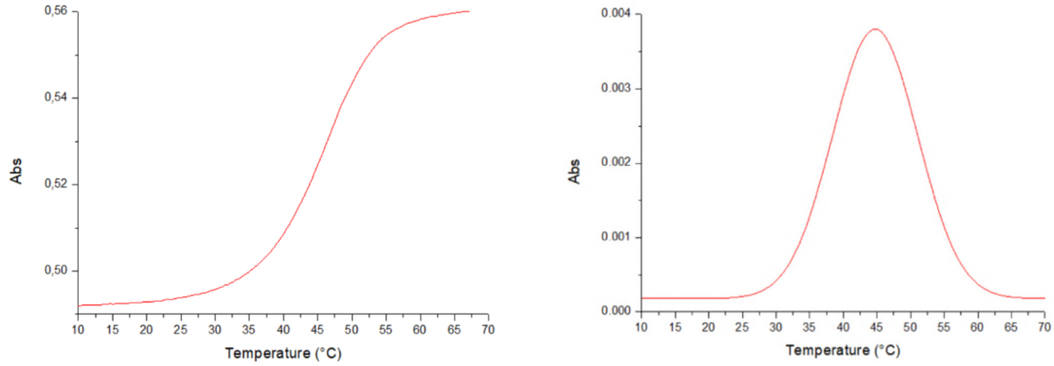

5. Complementary circular dichroism analysis

CD spectra were obtained on a JASCO J-815 as described in the General Information. Iridium complexes spectra were recorded once in CH_2Cl_2 at a 66 µM concentration, then experiments were done at 5 µM under the described conditions (see Figure 2B and 2C of the main text) to minimize DNAzyme consumption.

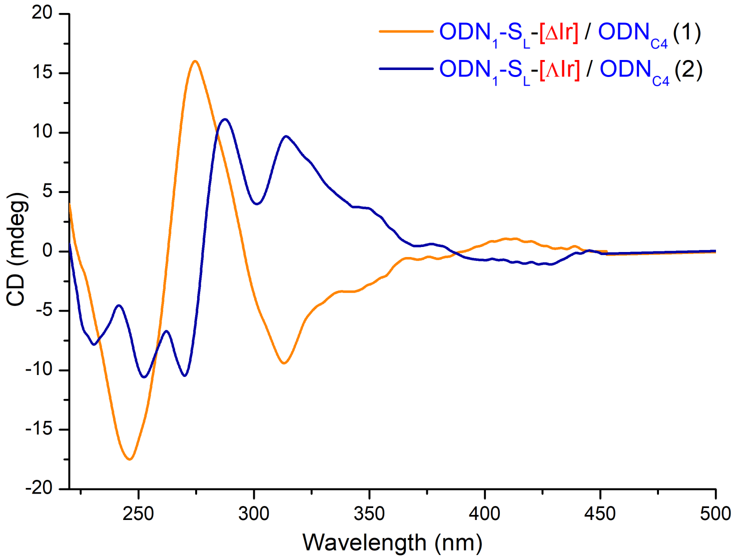

Figure S1 *Circular dichroism spectra of duplexes ODN$_1$-S$_L$-[ΔIr]/ODN$_{C4}$ and ODN$_1$-S$_L$-[ΛIr]/ODN$_{C4}$ (5µM)*

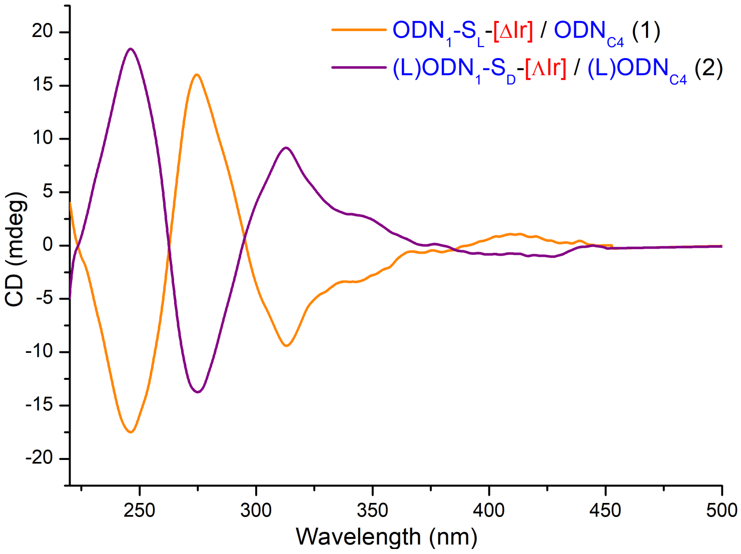

Figure S2 *Circular dichroism spectra of duplexes ODN$_1$-S$_L$-[ΔIr]/ODN$_{C4}$ and (L)ODN$_1$-S$_D$-[ΛIr]/(L)ODN$_{C4}$ (5µM)*

IV. Synthesis of substrates

1. General procedure for the synthesis of substrates

General procedure D

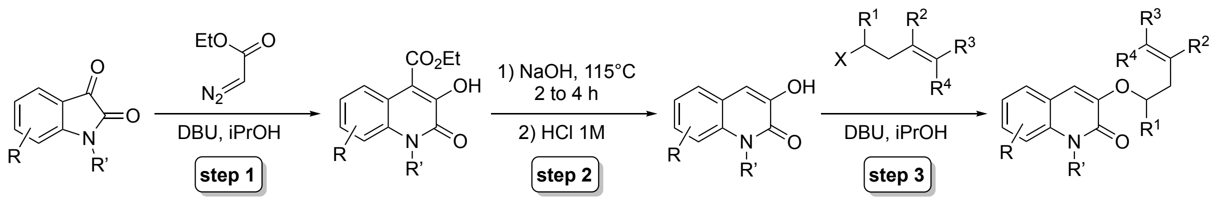

The quinolone substrates **1a-p** were synthesized according to the procedure reported by Yoon team[4] over 3 steps.

Step 1: A round bottom flask was charged with isatin (2 mmol, 1.0 equiv.) and EtOH/DMF (5:1, 0.2 M with respect to substrate). DBU (0.3 mmol, 0.15 equiv.) was added quickly dropwise. Ethyl diazoacetate (85 wt% in CH_2Cl_2, 4 mmol, 2.0 equiv.) was then added, and the mixture was stirred at room temperature for 15 h. The reaction was concentrated in vacuo to remove most of the ethanol. Aqueous 1 M HCl (7 mL) was added slowly, resulting in vigorous evolution of N_2. After 2 h of stirring, the resultant precipitate was collected by suction filtration, rinsed with H_2O (2 x 10 mL) and Et_2O (2 x 10 mL), then dried under high vacuum to give the desired product without further purification.

Step 2: NaOH (4 mmol, 2 equiv.) was added to a round bottom flask and dissolved in H_2O (0.1 M with respect to substrate). The appropriate hydroxyquinoloneethyl ester (2 mmol, 1 equiv.) was then added, and the solution brought to 115 °C (reflux), stirring vigorously for 24 h. After cooling, the reaction was acidified to pH 2 using 1 M HCl. The resulting slurry was collected by suction filtration on a frit, rinsed with water (2 x 10 mL) and Et_2O (2 x 10 mL), then dried under high vacuum to afford the decarboxylated quinolinone.

Step 3: (alkylation with homoallylbromide/tosyl): A round-bottomed flask was charged with hydroxyquinolone (2.0 mmol, 1.0 equiv.) and *i*-PrOH (0.25 M with respect to substrate). DBU (3.2 mmol, 1.6 equiv.) was then added to this slurry. Next, the homoallylbromide/tosyl (1.5 mmol, 1.25 equiv.) was added dropwise, and the solution was heated at reflux (100 °C) under N_2 for 4 h. Following this period, the mixture was cooled to room temperature and concentrated in vacuo. The crude residue was dissolved in CH_2Cl_2 (40 mL) and sequentially washed with 1 M NaOH (30 mL) and 0.1 M HCl (30 mL). The organic phase was dried over Na_2SO_4, filtered, and concentrated. The crude product was purified by flash column chromatography on silica gel to afford the alkylated quinolinone.

2. Characterization of substrates

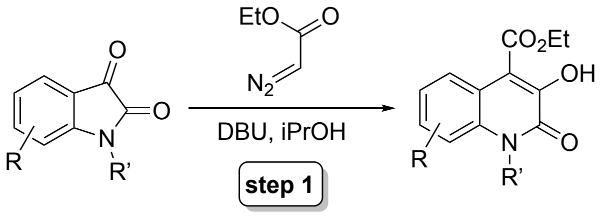

Ethyl 3-hydroxy-2-oxo-1,2-dihydroquinoline-4-carboxylate

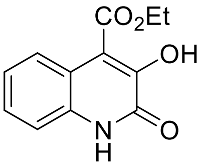

Following the general procedure D (step 1), the title compound (930 mg, 80 % yield) was isolated without purification as an orange solid from isatin (736 mg, 5 mmol) and diazoacetate (1.05 mL, 10 mmol). Spectroscopic data were consistent with the literature data.[4]

Ethyl 6-fluoro-3-hydroxy-2-oxo-1,2-dihydroquinoline-4-carboxylate

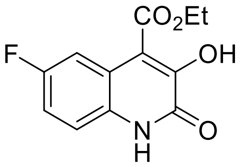

Following the general procedure D (step 1), the title compound (1.21 g, 88 % yield) was isolated without purification as an orange solid from 5-fluoroisatin (800 mg, 4.8 mmol) and diazoacetate (1 mL, 9.6 mmol). Spectroscopic data were consistent with the literature data.[5]

Ethyl 6-methyl-3-hydroxy-2-oxo-1,2-dihydroquinoline-4-carboxylate

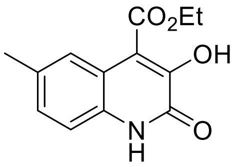

Following the general procedure D (step 1), the title compound (1.34 g, 88 % yield) was isolated without purification as a beige solid from 5-methylisatin (1 g, 6.2 mmol) and diazoacetate (1.3 mL, 12.4 mmol). Spectroscopic data were consistent with the literature data.[4]

Ethyl 6-methoxy-3-hydroxy-2-oxo-1,2-dihydroquinoline-4-carboxylate

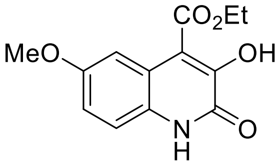

Following the general procedure D (step 1), the title compound (1.16 g, 79 % yield) was isolated without purification as a brown solid from 5-methoxyisatin (1 g, 5.6 mmol) and diazoacetate (1.17 mL, 11.2 mmol). Spectroscopic data were consistent with the literature data.[4]

Ethyl 6-chloro-3-hydroxy-2-oxo-1,2-dihydroquinoline-4-carboxylate

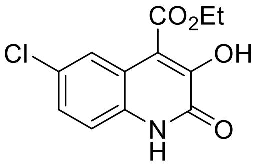

Following the general procedure D (step 1), the title compound (1.25 g, 85 % yield) was isolated without purification as a beige solid from 5-chloroisatin (1 g, 5.5 mmol) and diazoacetate (1.15 mL, 11 mmol). Spectroscopic data were consistent with the literature data.[4]

Ethyl 6-bromo-3-hydroxy-2-oxo-1,2-dihydroquinoline-4-carboxylate

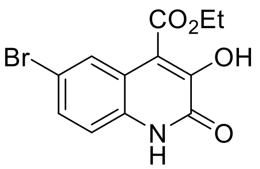

Following the general procedure D (step 1), the title compound (1.04 g, 76 % yield) was isolated without purification as an orange solid from 5-bromoisatin (1 g, 4.4 mmol) and diazoacetate (920 µL, 8.8 mmol). Spectroscopic data were consistent with the literature data.[4]

Ethyl 7-fluoro-3-hydroxy-2-oxo-1,2-dihydroquinoline-4-carboxylate

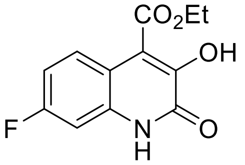

Following the general procedure D (step 1), the title compound (1.51 g, 68 % yield) was isolated without purification as an orange solid from 6-fluoroisatin (1 g, 6 mmol) and diazoacetate (1.26 mL, 12 mmol). Spectroscopic data were consistent with the literature data.[5]

Ethyl 7-chloro-3-hydroxy-2-oxo-1,2-dihydroquinoline-4-carboxylate

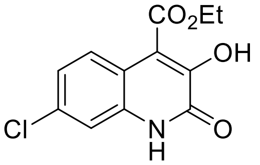

Following the general procedure D (step 1), the title compound (2.30 g, 86 % yield) was isolated without purification as an orange solid from 6-chloroisatin (1.81 mg, 10 mmol) and diazoacetate (3.5 mL, 28 mmol). Spectroscopic data were consistent with the literature data.[4]

Ethyl 8-fluoro-3-hydroxy-2-oxo-1,2-dihydroquinoline-4-carboxylate

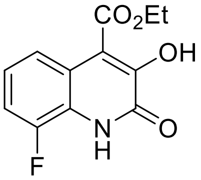

Following the general procedure D (step 1), the title compound (1.61 g, 64 % yield) was isolated without purification as an orange solid from 7-fluoroisatin (1.65 mg, 10 mmol) and diazoacetate (2.5 mL, 20.2 mmol). Spectroscopic data were consistent with the literature data.[4]

Ethyl 8-methyl-3-hydroxy-2-oxo-1,2-dihydroquinoline-4-carboxylate

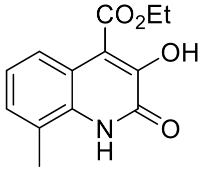

Following the general procedure D (step 1), the title compound (1.24 g, 99 % yield) was isolated without purification as an orange solid from 7-methylisatin (800 mg, 5 mmol) and diazoacetate (1 mL, 10 mmol). Spectroscopic data were consistent with the literature data.[5]

Ethyl 3-hydroxy-2-oxo-1,2-dihydro-1,8-naphthyridine-4-carboxylate

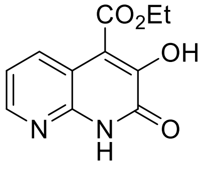

Following the general procedure D (step 1), the title compound (1.04 g, 82 % yield) was isolated without purification as a yellow solid from 7-azaisatin (800 mg, 5.4 mmol) and diazoacetate (1.14 mL, 10.8 mmol). **^{1}H NMR** (400 MHz, DMSO-d_6) δ 12.72 (s, 1H), 10.64 (s, 1H), 8.39 (dd, J = 4.7, 1.6 Hz, 1H), 7.88 (dd, J = 8.0, 1.7 Hz, 1H), 7.26 (dd, J = 8.0, 4.7 Hz, 1H), 4.40 (q, J = 7.1 Hz, 2H), 1.32 (t, J = 7.1 Hz, 3H). **^{13}C NMR** (151 MHz, DMSO-d_6) δ 164.8, 158.8, 146.9, 145.2, 144.4, 132.0, 118.9, 114.5, 112.8, 61.5, 14.1. **HRMS** (ESI+) *m/z*: Calcd for $C_{11}H_{11}N_2O_4$ $[M+H]^+$: 235.0719 found 235.0720.

Ethyl 1-ethyl-3-hydroxy-2-oxo-1,2-dihydroquinoline-4-carboxylate

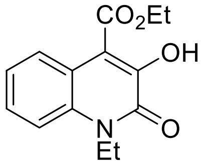

Following the general procedure D (step 1), the title compound (1.2 g, 99 % yield) was isolated without purification as a yellow solid from 1-ethylisatin (800 mg, 4.6 mmol) and diazoacetate (960 µL, 9.2 mmol). **^{1}H NMR** (400 MHz, DMSO-d_6) δ 10.22 (s, 1H), 7.62 (dd, J = 8.7, 1.1 Hz, 1H), 7.50 (ddd, J = 8.6, 7.1, 1.5 Hz, 1H), 7.41 (dd, J = 8.0, 1.5 Hz, 1H), 7.28 (ddd, J = 8.1, 7.1, 1.0 Hz, 1H), 4.46 – 4.31 (m, 4H), 1.32 (t, J = 7.1 Hz, 3H), 1.25 (t, J = 7.1 Hz, 3H). **^{13}C NMR** (151 MHz, DMSO-d_6) δ 165.3, 157.3, 142.3, 133.0, 127.6, 124.1, 123.0, 117.7, 116.4, 114.9, 61.4, 37.7, 14.1, 12.6. **HRMS** (ESI+) *m/z*: Calcd for $C_{14}H_{16}NO_4$ $[M+H]^+$: 262.1076 found 262.1074.

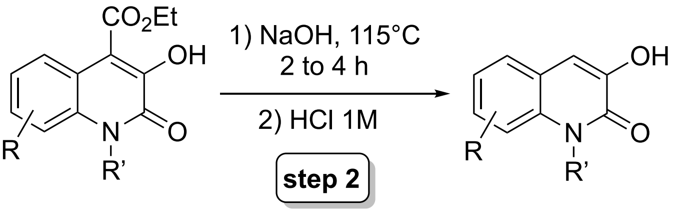

3-Hydroxyquinolin-2(1H)-one

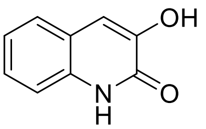

Following the general procedure D (step 2), the title compound (950 mg, 78 % yield) was isolated without purification as an orange solid from ethyl 3-hydroxy-2-oxo-1,2-dihydroquinoline-4-carboxylate (1.77 g, 7.6 mmol) and NaOH (607 mg, 15.2 mmol). The resulting solid was then acidified with HCl 1 M (10 – 12 mL). Spectroscopic data were consistent with the literature data.[4]

6-Fluoro-3-hydroxyquinolin-2(1H)-one

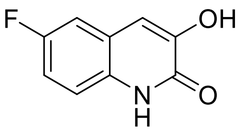

Following the general procedure D (step 2), the title compound (717 mg, 82 % yield) was isolated without purification as a beige solid from ethyl 6-fluoro-3-hydroxy-2-oxo-1,2-dihydroquinoline-4-carboxylate (1 g, 4 mmol) and NaOH (320 mg, 8 mmol). The resulting solid was then acidified with HCl 1 M (15 mL). Spectroscopic data were consistent with the literature data.[5]

6-Methyl-3-hydroxyquinolin-2(1H)-one

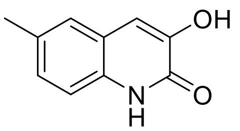

Following the general procedure D (step 2), the title compound (601 mg, 64 % yield) was isolated without purification as a brown solid from ethyl 6-methyl-3-hydroxy-2-oxo-1,2-dihydroquinoline-4-carboxylate (1.34 g, 5.4 mmol) and NaOH (432 mg, 10.8 mmol). The resulting solid was then acidified with HCl 1 M (10 mL). Spectroscopic data were consistent with the literature data.[4]

6-Methoxy-3-hydroxyquinolin-2(1H)-one

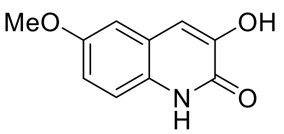

Following the general procedure D (step 2), the title compound (627 mg, 75 % yield) was isolated without purification as a brown solid from ethyl 6-methoxy-3-hydroxy-2-oxo-1,2-dihydroquinoline-4-carboxylate (1.15 g, 4.4 mmol) and NaOH (352 mg, 8.8 mmol). The resulting solid was then acidified with HCl 1 M (10 mL). Spectroscopic data were consistent with the literature data.[4]

6-Chloro-3-hydroxyquinolin-2(1H)-one

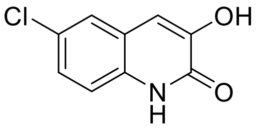

Following the general procedure D (step 2), the title compound (950 mg, 78 % yield) was isolated without purification as an orange solid from ethyl 6-chloro-3-hydroxy-2-oxo-1,2-dihydroquinoline-4-carboxylate (1.25 g, 4.7 mmol) and NaOH (375 mg, 9.4 mmol). The resulting solid was then acidified with HCl 1 M (10 – 12 mL). Spectroscopic data were consistent with the literature data.[4]

6-Bromo-3-hydroxyquinolin-2(1H)-one

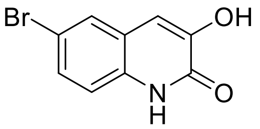

Following the general procedure D (step 2), the title compound was isolated without purification as a crude (570 mg, 14 % of unreacted substrate, corrected masse = 490 mg, 62 % yield) from ethyl 6-bromo-3-hydroxy-2-oxo-1,2-dihydroquinoline-4-carboxylate (1.03 g, 3.3 mmol) and NaOH (132 mg, 6.6 mmol) and then acidified with HCl 1 M (10 – 12 mL). The crude was engaged in the next step. Spectroscopic data were consistent with the literature data.[4]

7-Fluoro-3-hydroxyquinolin-2(1H)-one

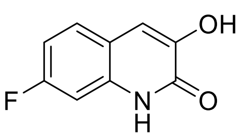

Following the general procedure D (step 2), the title compound (560 mg, 76 % yield) was isolated without purification as a beige solid from ethyl 7-fluoro-3-hydroxy-2-oxo-1,2-dihydroquinoline-4-carboxylate (1 g, 4.1 mmol) and NaOH (328 mg, 8.2 mmol). The resulting solid was then acidified with HCl 1 M (15 mL). Spectroscopic data were consistent with the literature data.[5]

7-Chloro-3-hydroxyquinolin-2(1H)-one

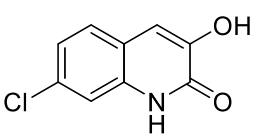

Following the general procedure D (step 2), the title compound (1.32 g, 80 % yield) was isolated without purification as a beige solid from ethyl 7-chloro-3-hydroxy-2-oxo-1,2-dihydroquinoline-4-carboxylate (2.27 g, 4.5 mmol) and NaOH (670 mg, 17 mmol). The resulting solid was then acidified with HCl 1 M (15 mL). Spectroscopic data were consistent with the literature data.[4]

8-Fluoro-3-hydroxyquinolin-2(1H)-one

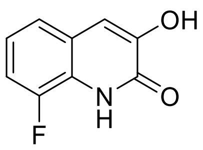

Following the general procedure D (step 2), the title compound (87 mg, 78 % yield) was isolated without purification as a beige solid from ethyl 8-fluoro-3-hydroxy-2-oxo-1,2-dihydroquinoline-4-carboxylate (1.57 g, 6.25 mmol) and NaOH (500 mg, 12.5 mmol). The resulting solid was then acidified with HCl 1 M (20 mL). Spectroscopic data were consistent with the literature data.[4]

8-Methyl-3-hydroxyquinolin-2(1H)-one

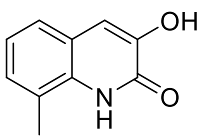

Following the general procedure D (step 2), the title compound (542 mg, 63 % yield) was isolated without purification as a beige solid from ethyl 8-methyl-3-hydroxy-2-oxo-1,2-dihydroquinoline-4-carboxylate (1.2 g, 4.9 mmol) and NaOH (392 mg, 9.8 mmol). The resulting solid was then acidified with HCl 1 M (15 mL). Spectroscopic data were consistent with the literature data.[5]

3-Hydroxy-1,8-naphthyridin-2(1H)-one

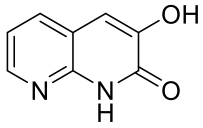

Following the general procedure D (step 2), the title compound (705 mg, 98 %) was isolated without purification as a beige solid from ethyl 3-hydroxy-2-oxo-1,2-dihydro-1,8-naphthyridine-4-carboxylate (1.04 g, 4.4 mmol) and NaOH (355 mg, 8.9 mmol). The resulting solid was then acidified with HCl 1 M (15 mL). Spectroscopic data were consistent with the literature data.[5]

1-Ethyl-3-hydroxyquinolin-2(1H)-one

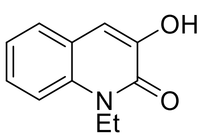

Following the general procedure D (step 2), the title compound (360 mg, 45 % yield) was isolated without purification as a beige solid from ethyl 1-ethyl-3-hydroxy-2-oxo-1,2-dihydroquinoline-4-carboxylate (1.1 g, 4.2 mmol) and NaOH (336 mg, 8.4 mmol). The resulting solid was then acidified with HCl 1 M (15 mL). Spectroscopic data were consistent with the literature data.[6]

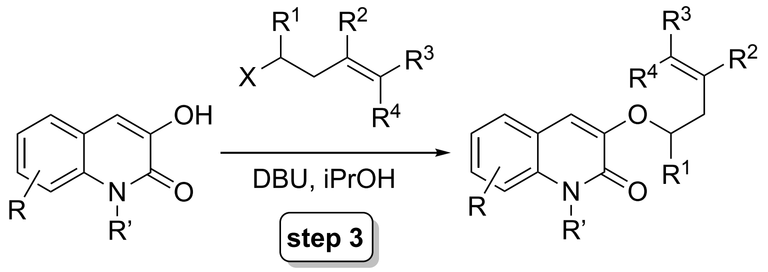

3-(But-3-en-1-yloxy)quinolin-2(1H)-one (1a)

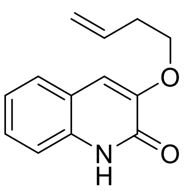

Following the general procedure D (step 3), the title compound (408 mg, 70 % yield) was isolated as white solid by flash chromatography (cyclohexane/EtOAc 7:3) from 3-hydroxyquinolin-2(1H)-one (438 mg, 2.7 mmol) and 4-bromobut-1-ene (345 µL, 3.4 mmol). Spectroscopic data were consistent with the literature data.[4]

3-(But-3-en-1-yloxy)-6-fluoroquinolin-2(1H)-one (1b)

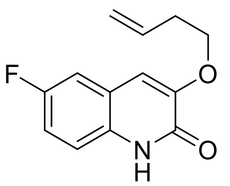

Following the general procedure D (step 3), the title compound (357 mg, 48 % yield) was isolated as white solid by flash chromatography (cyclohexane/acetone 7:3) from 6-fluoro-3-hydroxyquinolin-2(1H)-one (580 mg, 3.2 mmol) and 4-bromobut-1-ene (406 µL, 4 mmol). **^{1}H NMR** (400 MHz, $CDCl_3$) δ 12.16 (s, 1H), 7.41 (dd, *J* = 8.9, 4.7 Hz, 1H), 7.18 – 7.08 (m, 2H), 6.95 (s, 1H), 5.95 (ddt, *J* = 17.1, 10.3, 6.8 Hz, 1H), 5.30 – 5.10 (m, 2H), 4.14 (t, *J* = 7.1 Hz, 2H), 2.72 (qt, *J* = 7.0, 1.4 Hz, 2H). **^{13}C NMR** (101 MHz, $CDCl_3$) δ 159.9, 159.6, 157.5, 148.9, 133.7, 130.1, 121.4 (d, J_{C-F} = 9.2 Hz), 117.9, 117.7 (d, J_{C-F} = 8.7 Hz), 115.6 (d, J_{C-F} = 24.5 Hz), 112.3 (d, J_{C-F} = 3.0 Hz), 111.2 (d, J_{C-F} = 23.3 Hz), 68.5, 33.2. **^{19}F NMR** (376 MHz, $CDCl_3$) δ -119.71. **HRMS** (ESI+) *m/z*: Calcd for $C_{13}H_{13}FNO_2$ $[M+H]^+$: 234.0930 found 234.0921.

3-(But-3-en-1-yloxy)-6-methylquinolin-2(1H)-one (1c)

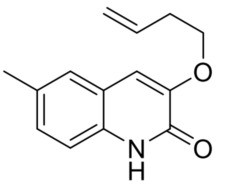

Following the general procedure D (step 3), the title compound (512 mg, 62 % yield) was isolated as white solid by flash chromatography (cyclohexane/acetone 7:3) from 6-methyl-3-hydroxyquinolin-2(1H)-one (600 mg, 3.4 mmol) and 4-bromobut-1-ene (435 µL, 4.3 mmol). Spectroscopic data were consistent with the literature data.[4]

3-(But-3-en-1-yloxy)-6-methoxyquinolin-2(1H)-one (1d)

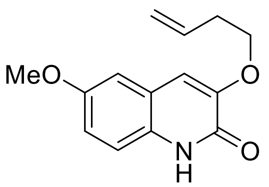

Following the general procedure D (step 3), the title compound (428 mg, 55 % yield) was isolated as white solid by flash chromatography (cyclohexane/acetone 7:3) from 6-methoxy-3-hydroxyquinolin-2(1H)-one (620 mg, 3.2 mmol) and 4-bromobut-1-ene (416 µL, 4.1 mmol). Spectroscopic data were consistent with the literature data.[4]

3-(But-3-en-1-yloxy)-6-chloroquinolin-2(1H)-one (1e)

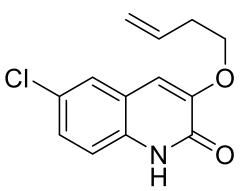

Following the general procedure D (step 3), the title compound (512 mg, 66 % yield) was isolated as white solid by flash chromatography (cyclohexane/acetone 7:3) from 6-chloro-3-hydroxyquinolin-2(1H)-one (610 mg, 3.1 mmol) and 4-bromobut-1-ene (396 µL, 3.9 mmol). Spectroscopic data were consistent with the literature data.[4]

3-(But-3-en-1-yloxy)-6-bromoquinolin-2(1H)-one (1f)

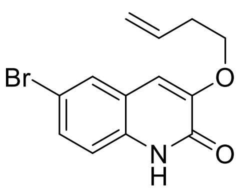

Following the general procedure D (step 3), the title compound (160 mg, 23 % yield) was isolated as white solid by flash chromatography (cyclohexane/acetone 7:3) from 6-bromo-3-hydroxyquinolin-2(1H)-one (570 mg, 2.4 mmol) and 4-bromobut-1-ene (305 µL, 3.0 mmol). Spectroscopic data were consistent with the literature data.[4]

3-(But-3-en-1-yloxy)-7-fluoroquinolin-2(1H)-one (1g)

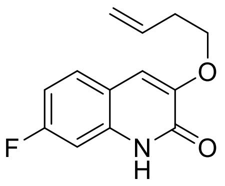

Following the general procedure D (step 3), the title compound (495 mg, 68 % yield) was isolated as white solid by flash chromatography (cyclohexane/acetone 7:3) from 7-fluoro-3-hydroxyquinolin-2(1H)-one (560 mg, 3.1 mmol) and 4-bromobut-1-ene (558 µL, 5.5 mmol). **^{1}H NMR** (400 MHz, $CDCl_3$) δ 11.69 (s, 1H), 7.44 (dd, J = 8.7, 5.7 Hz, 1H), 7.12 (dd, J = 9.4, 2.5 Hz, 1H), 6.99 (s, 1H), 6.95 (td, J = 8.6, 2.5 Hz, 1H), 5.95 (ddt, J = 17.1, 10.3, 6.8 Hz, 1H), 5.28 – 5.13 (m, 2H), 4.12 (t, J = 7.1 Hz, 2H), 2.72 (qt, J = 7.0, 1.4 Hz, 2H). **^{13}C NMR** (101 MHz, $CDCl_3$) δ 163.4, 161.0, 159.9, 147.2, 134.7 (d, J_{C-F} = 11.7 Hz), 133.8, 128.1 (d, J_{C-F} = 9.5 Hz), 117.8, 113.2, 111.5 (d, J_{C-F} = 23.4 Hz), 102.3 (d, J_{C-F} = 25.7 Hz), 68.5, 33.3. **^{19}F NMR** (376 MHz, $CDCl_3$) δ -112.4. **HRMS** (ESI+) m/z: Calcd for $C_{13}H_{13}FNO_2$ $[M+H]^+$: 234.0930 found 234.0922.

3-(But-3-en-1-yloxy)-7-chloroquinolin-2(1H)-one (1h)

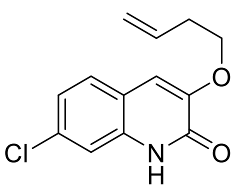

Following the general procedure D (step 3), the title compound (902 mg, 56 % yield) was isolated as white solid by flash chromatography (cyclohexane/acetone 7:3) from 7-chloro-3-hydroxyquinolin-2(1H)-one (1.27 g, 6.5 mmol) and 4-bromobut-1-ene (860 µL, 8.1 mmol). Spectroscopic data were consistent with the literature data.[4]

3-(But-3-en-1-yloxy)-8-fluoroquinolin-2(1H)-one (1i)

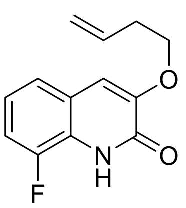

Following the general procedure D (step 3), the title compound (385 mg, 35 % yield) was isolated as white solid by flash chromatography (cyclohexane/acetone 7:3) from 8-fluoro-3-hydroxyquinolin-2(1H)-one (834 mg, 4.65 mmol) and 4-bromobut-1-ene (593 µL, 5.81 mmol). Spectroscopic data were consistent with the literature data.[4]

3-(But-3-en-1-yloxy)-8-methylquinolin-2(1H)-one (1j)

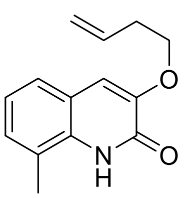

Following the general procedure D (step 3), the title compound (341 mg, 48 % yield) was isolated as white solid by flash chromatography (cyclohexane/acetone 7:3) from 8-methyl-3-hydroxyquinolin-2(1H)-one (530 mg, 3.1 mmol) and 4-bromobut-1-ene (740 µL, 4.6 mmol). **^{1}H NMR** (400 MHz, $CDCl_3$) δ 9.17 (s, 1H), 7.36 – 7.31 (m, 1H), 7.23 – 7.18 (m, 1H), 7.10 (t, *J* = 7.6 Hz, 1H), 6.96 (s, 1H), 5.94 (ddt, *J* = 17.0, 10.2, 6.8 Hz, 1H), 5.21 (dq, *J* = 17.2, 1.6 Hz, 1H), 5.17 – 5.11 (m, 1H), 4.11 (t, *J* = 7.1 Hz, 2H), 2.77 – 2.64 (m, 2H), 2.45 (s, 3H). **^{13}C NMR** (101 MHz, $CDCl_3$) δ 158.5, 148.0, 133.9, 132.0, 128.9, 124.9, 122.8, 122.3, 120.2, 117.7, 113.3, 68.4, 33.3, 16.8. **HRMS** (ESI+) *m/z*: Calcd for $C_{14}H_{16}NO_2$ $[M+H]^+$: 230.1181 found 230.1188.

3-(But-3-en-1-yloxy)-1,8-naphthyridin-2(1H)-one (1k)

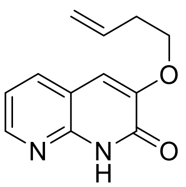

Following the general procedure D (step 3), the title compound (260 mg, 28 % yield) was isolated as beige solid by flash chromatography (cyclohexane/acetone 7:3) from 3-hydroxy-1,8-naphthyridin-2(1H)-one (705 mg, 4.3 mmol) and 4-bromobut-1-ene (560 µL, 5.4 mmol). **^{1}H NMR** (400 MHz, $CDCl_3$) δ 12.77 (s, 1H), 8.65 (dd, *J* = 4.9, 1.7 Hz, 1H), 7.82 (dd, *J* = 7.8, 1.7 Hz, 1H), 7.19 (dd, *J* = 7.8, 4.8 Hz, 1H), 6.85 (s, 1H), 5.93 (ddt, *J* = 17.1, 10.3, 6.8 Hz, 1H), 5.21 (dq, *J* = 17.2, 1.6 Hz, 1H), 5.13 (dq, *J* = 10.3, 1.3 Hz, 1H), 4.10 (t, *J* = 7.0 Hz, 2H), 2.68 (qt, *J* = 7.0, 1.4 Hz, 2H).

^{13}**C NMR** (101 MHz, $CDCl_3$) δ 158.8, 149.4, 146.8, 145.7, 134.6, 133.7, 118.7, 117.7, 115.7, 109.9, 68.5, 33.1. **HRMS** (ESI+) *m/z*: Calcd for $C_{12}H_{13}N_2O_2$ $[M+H]^+$: 217.0977 found 217.0969.

3-((4-Methylpent-3-en-1-yl)oxy)quinolin-2(1H)-one (1l)

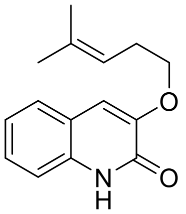

Following the general procedure D (step 3), the title compound (200 mg, 41 % yield) was isolated as white solid by flash chromatography (cyclohexane/acetone 7:3) from 3-hydroxyquinolin-2(1H)-one (322 mg, 2 mmol) and 5-bromo-2-methyl-2-pentene (333 µL, 2.5 mmol). Spectroscopic data were consistent with the literature data.[4]

3-((3-Methylbut-3-en-1-yl)oxy)quinolin-2(1H)-one (1m)

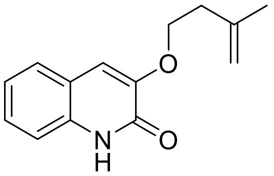

Following the general procedure D (step 3), the title compound (227 mg, 74 % yield) was isolated as white solid by flash chromatography (cyclohexane/acetone 7:3) from 3-hydroxyquinolin-2(1H)-one (215 mg, 1.3 mmol) and 3-methylbut-3-en-1-yl tosylate (400 mg, 1.7 mmol). Spectroscopic data were consistent with the literature data.[4]

3-(Pent-4-en-2-yloxy)quinolin-2(1H)-one (1n)

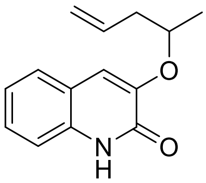

Following the general procedure D (step 3), the title compound (338 mg, 67 % yield) was isolated as white solid by flash chromatography (cyclohexane/acetone 7:3) from 3-hydroxyquinolin-2(1H)-one (350 mg, 2.2 mmol) and pent-4-en-2-tosyl (1 g, 4.4 mmol). ^{1}**H NMR** (400 MHz, $CDCl_3$) δ 11.30 (s, 1H), 7.46 (dd, *J* = 7.7, 1.1 Hz, 1H), 7.41 – 7.32 (m, 2H), 7.23 – 7.16 (m, 1H), 7.03 (s, 1H), 5.99 – 5.86 (m, 1H), 5.22 – 5.16 (m, 1H), 5.15 – 5.09 (m, 1H), 4.61 – 4.51 (m, 1H), 2.75 – 2.63 (m, 1H), 2.53 – 2.40 (m, 1H), 1.45 (d, *J* = 6.1 Hz, 3H). ^{13}**C NMR** (101 MHz, $CDCl_3$) δ 160.3, 147.0, 134.1, 133.8, 127.6, 126.3, 122.9, 120.5, 118.0, 115.9, 115.8, 115.7, 115.6, 74.8, 40.4, 19.2. **HRMS** (ESI+) *m/z*: Calcd for $C_{14}H_{16}NO_2$ $[M+H]^+$: 230.1181 found 230.1175.

3-(But-3-en-1-yloxy)-1-ethylquinolin-2(1H)-one (1p)

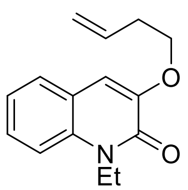

Following the general procedure D (step 3), the title compound (368 mg, 86 % yield) was isolated as beige solid by flash chromatography (cyclohexane/acetone 7:3) from 1-ethyl-3-hydroxyquinolin-2(1H)-one (350 mg, 1.9 mmol) and 4-bromobut-1-ene (290 µL, 2.9 mmol). **^{1}H NMR** (400 MHz, $CDCl_3$) δ 7.51 – 7.31 (m, 3H), 7.24 – 7.17 (m, 1H), 6.89 (s, 1H), 5.95 (ddt, *J* = 17.1, 10.2, 6.8 Hz, 1H), 5.25 – 5.11 (m, 2H), 4.41 (q, *J* = 7.3 Hz, 2H), 4.08 (t, *J* = 7.1 Hz, 2H), 2.73 – 2.65 (m, 2H), 1.37 (t, *J* = 7.2 Hz, 3H). **^{13}C NMR** (126 MHz, $CDCl_3$) δ 157.8, 147.8, 134.5, 134.0, 127.6, 122.5, 121.1, 117.6, 113.9, 111.4, 68.2, 38.0, 33.3, 12.8. **HRMS** (ESI+) *m/z*: Calcd for $C_{15}H_{18}NO_2$ $[M+H]^+$: 244.1337 found 244.1329.

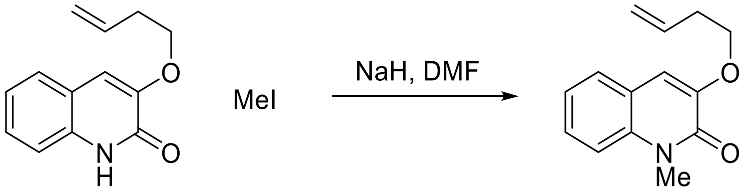

A flamed-dry round bottom flask was charged with 3-(but-3-en-1-yloxy)quinolin-2(1H)-one (1 equiv.) and DMF (0.2 M with respect to substrate). NaH was added (2 equiv.) and the suspension was stirred for 2 h under Argon. MeI was added (1.5 equiv.) and the mixture was stirred at room temperature for 15 h. The reaction was quenched with H_2O and extracted with EtOAc (3 x 25 mL). Organic phase was washed several times with H_2O to remove DMF traces, dry over Na_2SO_4, filtered and concentrated under reduced pressure. The crude was purified by flash chromatography (cyclohexane/acetone) to give the desired N-alkyl quinolinone.

3-(But-3-en-1-yloxy)-1-methylquinolin-2(1H)-one (1o)

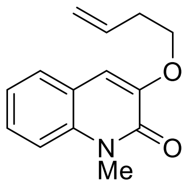

Following previous procedure, the title compound (32 mg, 27 % yield) was isolated by flash chromatography (cyclohexane/acetone 7:3) from 3-(but-3-en-1-yloxy)quinolin-2(1H)-one (100 mg, 0.5 mmol), NaH (45 mg, 1 mmol) and iodomethane (50 µL, 0.75 mmol). Spectroscopic data were consistent with the literature data.[4]

V. Reaction optimization

***Table S4** Single strand DNAzyme optimization*

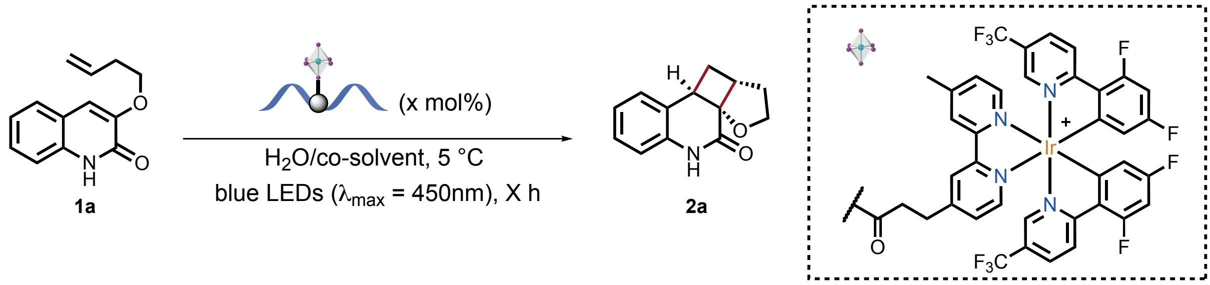

Entry	Photocatalyst[a]	% H_2O	Co-solvent	Time (h)	Conv. (%)[b]	er[b]
1	ODN_1-S_L-[Ir] (1 mol%)	98	THF	6	57	50:50
2	ODN_1-S_L-[Ir] (1 mol%)	98	THF	14	86	50:50
3	ODN_1-S_L-[Ir] (1 mol%)	98	THF	24	94	50:50
4	ODN_1-S_L-[Ir] (0.5 mol%)	98	THF	24	72	50:50
5	ODN_1-S_L-[Ir] (2 mol%)	98	THF	8	76	50:50

[a]ODN_1-S_L = 5'-GCCAGCS$_L$GACCG-3'. [b]Determined by chiral HPLC.

***Table S5** The influence of salts*

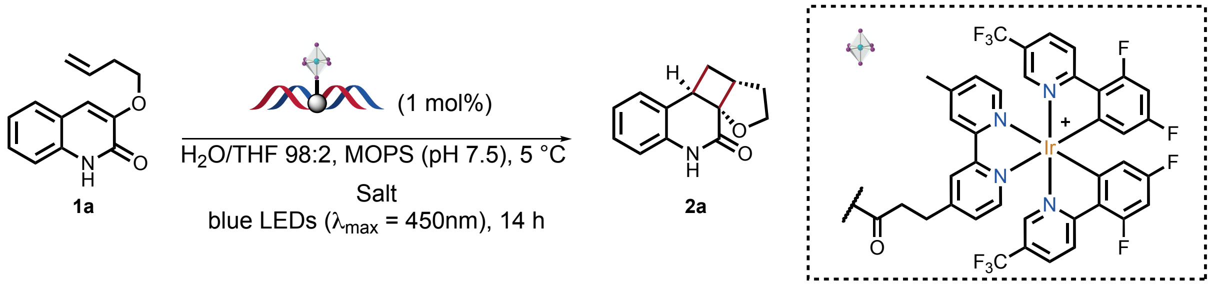

Entry	Lead strand[a]	Counter-strand	Salt conc.	Conv. (%)[b]	er[b]
1	ODN_1-S_L-[Ir]	5'-CGGTCTGCTGGC-3'	-	88	75:25
2[c]	ODN_1-S_L-[Ir]	5'-CGGTCTGCTGGC-3'	-	91	80:20
3[c]	ODN_1-S_L-[Ir]	5'-CGGTCCGCTGGC-3'	NaCl 0.1 M	83	90:10
4[c]	ODN_1-S_L-[Ir]	5'-CGGTCCGCTGGC-3'	NaCl 0.5 M	86	90.5:9.5
5[c]	ODN_1-S_L-[Ir]	5'-CGGTCCGCTGGC-3'	NaCl 1 M	97	91.5:8.5
6[c]	ODN_1-S_L-[Ir]	5'-CGGTCCGCTGGC-3'	$NaBF_4$ 0.1 M	94	89:11
7[c]	ODN_1-S_L-[Ir]	5'-CGGTCCGCTGGC-3'	$NaBF_4$ 1 M	88	89.5:10.5
8[c]	ODN_1-S_L-[Ir]	5'-CGGTCCGCTGGC-3'	$NaPF_6$ 0.1 M	46	50:50
9[c]	ODN_1-S_L-[Ir]	5'-CGGTCCGCTGGC-3'	$MgCl_2$ 0.1 M	97	80:20
10[c]	ODN_1-S_L-[Ir]	5'-CGGTCCGCTGGC-3'	$MgCl_2$ 1 M	91	88:12
11[c]	ODN_1-S_L-[Ir]	5'-CGGTCCGCTGGC-3'	NaBArF 0.1 M	31	55:45

[a]1 mol%, ODN_1-S_L = 5'-GCCAGCS$_L$GACCG-3'. [b]Determined by chiral HPLC. [c]1.5 mol% of counter-strand.

Table S6 *Influence of the buffer, the ODN length and the catalyst charge*

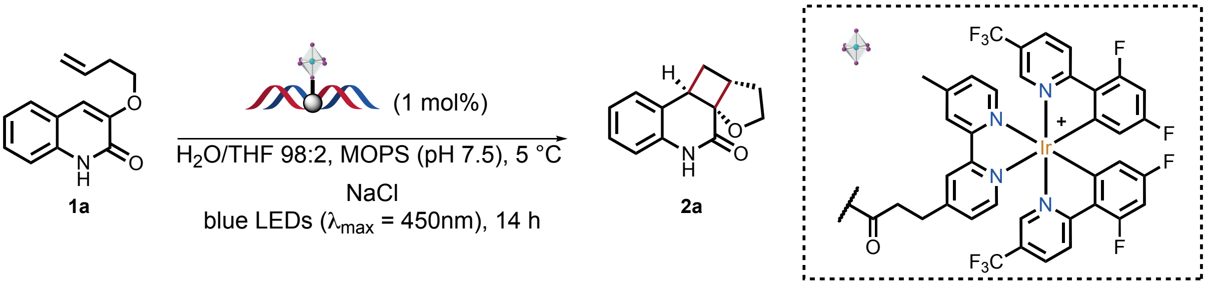

Entry	Lead strand[a]	Counter-strand[b]	Buffer	Conversion (%)[c]	er[c]
1[d]	ODN$_1$-S$_L$-[Ir]	5'-CGGTCTGCTGGC-3'	MOPS pH 5.5	83	80:20
2[d]	ODN$_1$-S$_L$-[Ir]	5'-CGGTCTGCTGGC-3'	MOPS pH 6.5	81	80:20
3[d]	ODN$_1$-S$_L$-[Ir]	5'-CGGTCTGCTGGC-3'	MOPS pH 7.5	91	80:20
4[d]	ODN$_1$-S$_L$-[Ir]	5'-CGGTCTGCTGGC-3'	MOPS pH 8.5	59	80:20
5[d]	ODN$_1$-S$_L$-[Ir]	5'-CGGTCTGCTGGC-3'	MES pH 6.5	74	80:20
6[d]	ODN$_1$-S$_L$-[Ir]	5'-CGGTCTGCTGGC-3'	MES pH 7.5	66	80:20
7[d]	ODN$_1$-S$_L$-[Ir]	5'-CGGTCTGCTGGC-3'	MES pH 8.5	61	80:20
8[e]	ODN$_1$-S$_L$-[Ir]	5'-CGGTCTGCTGGC-3'	MOPS pH 7.5	91	84:16
9[e]	ODN$_2$-S$_L$-[Ir]	5'-GACGGTCTGCTGGCTC-3'	MOPS pH 7.5	86	75:25
10[e]	ODN$_1$-S$_L$-[ΔIr]	5'-CGGTCCGCTGGC-3'	MOPS pH 7.5	95	95.5:4.5
11[e,f]	ODN$_1$-S$_L$-[ΔIr]	5'-CGGTCCGCTGGC-3'	MOPS pH 7.5	95[g]	95.5:4.5
12[e]	ODN$_1$-S$_L$-[ΛIr]	5'-CGGTCCGCTGGC-3'	MOPS pH 7.5	92	56.5:43.5

[a]1 mol%, ODN$_1$-S$_L$ = 5'-GCCAGCS$_L$GACCG-3'. [b]1.5 mol% of counter-strand. [c]Determined by chiral HPLC. [d]0,1M NaCl. [e]1M NaCl. [f]5 mol% catalyst charge. [g]After 8 h.

Table S7 *Evaluation of the influence of the base facing the photocatalyst*

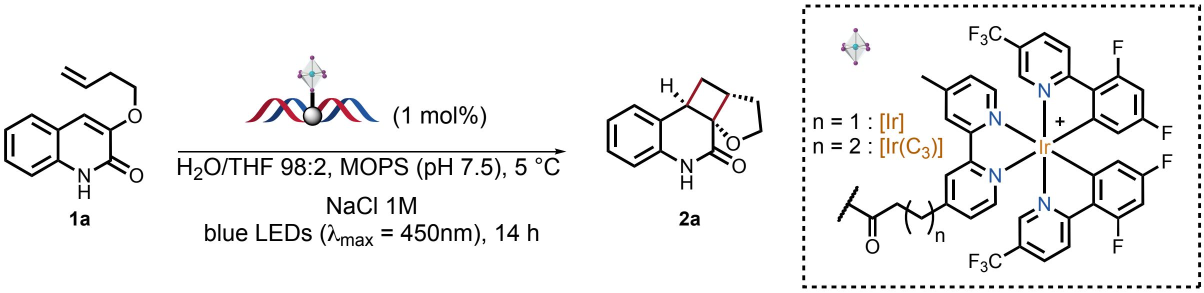

Entry	Lead strand[a]	Counter-strand[b]	Conversion (%)[c]	er[c]
1	ODN$_1$-S$_L$-[Ir]	5'-CGGTCAGCTGGC-3'	88	56:44
2	ODN$_1$-S$_L$-[Ir]	5'-CGGTCGGCTGGC-3'	80	68:32
3	ODN$_1$-S$_L$-[Ir]	5'-CGGTCTGCTGGC-3'	91	84:16
4	ODN$_1$-S$_L$-[Ir]	5'-CGGTCCGCTGGC-3'	97	91.5:8.5
5	ODN$_1$-S$_L$-[Ir]	5'-CGGTCC3GCTGGC-3'	76	69:31
6	ODN$_1$-S$_L$-[Ir]	(r)5'-CGGTCAGCTGGC-3'	88	50:50
7	ODN$_1$-S$_L$-[Ir]	(r)5'-CGGTCGGCTGGC-3'	97	50:50
8	ODN$_1$-S$_L$-[Ir]	(r)5'-CGGTCUGCTGGC-3'	91	50:50
9	ODN$_1$-S$_L$-[Ir]	(r)5'-CGGTCCGCTGGC-3'	94	50:50
10	ODN$_1$-S$_L$-[Ir(C$_3$)]	5'-CGGTCAGCTGGC-3'	81	53:47
11	ODN$_1$-S$_L$-[Ir(C$_3$)]	5'-CGGTCGGCTGGC-3'	88	59:41
12	ODN$_1$-S$_L$-[Ir(C$_3$)]	5'-CGGTCTGCTGGC-3'	81	72:28
13	ODN$_1$-S$_L$-[Ir(C$_3$)]	5'-CGGTCCGCTGGC-3'	88	80:20
14	ODN$_1$-S$_L$-[Ir(C$_3$)]	5'-CGGTCC3GCTGGC-3'	91	63:37

[a]1 mol%, ODN$_1$-S$_L$ = 5'-GCCAGCS$_L$GACCG-3'. [b]1.5 mol% of counter-strand. [c]Determined by chiral HPLC.

Table S8 *Evaluation of the influence of neighboring base pairs*

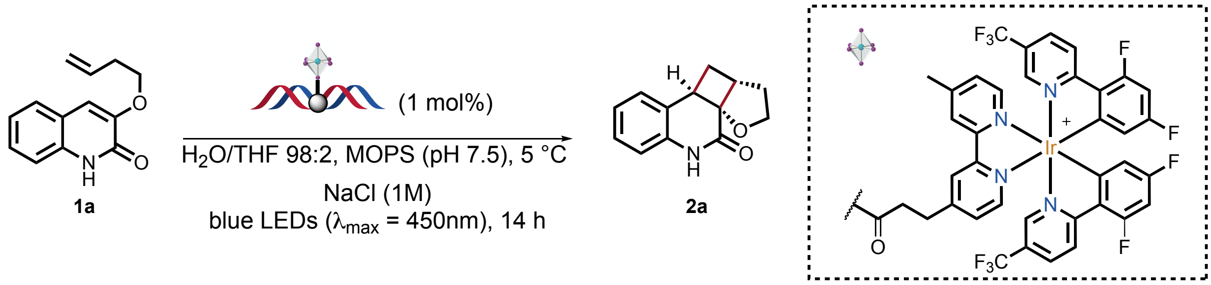

Entry		duplex sequences	conv.[c]	er[c]
1	ODN_1-S_L-[Ir][a]	5' G C C A G C_6 S_LIr G A C C G 3'	97	91.5:8.5
	ODN_{c4}[b]	3' C G G T C G C C_{17} T G G C 5'		
2	ODN_4-S_L-[Ir][a]	5' G C C A G G_6 S_LIr G A C C G 3'	91	66:34
	ODN_{c12}[b]	3' C G G T C C C C_{17} T G G C 5'		
3	ODN_5-S_L-[Ir][a]	5' G C C A G C_6 S_LIr C A C C G 3'	88	62:38
	ODN_{c13}[b]	3' C G G T C G C G_{17} T G G C 5'		
4	ODN_3-S_L-[Ir][a]	5' G C C A G G_6 S_LIr C A C C G 3'	91	51.5:48.5
	ODN_{c11}[b]	3' C G G T C C C G_{17} T G G C 5'		
5	ODN_8-S_L-[Ir][a]	5' G C C A G A S_LIr T A C C G 3'	91	52:48
	ODN_{c16}[b]	3' C G G T C T C A_{17} T G G C 5'		
6	ODN_6-S_L-[Ir][a]	5' G C C A G T_6 S_LIr G A C C G 3'	91	88:12
	ODN_{c14}[b]	3' C G G T C A C C_{17} T G G C 5'		
7	ODN_7-S_L-[Ir][a]	5' G C C A G C_6 S_LIr A A C C G 3'	70	61:39
	ODN_{c15}[b]	3' C G G T C G C T_{17} T G G C 5'		

[a]1 mol% of lead strand ODN_x-S_L-[Ir]. [b]1.5 mol% of counter-strand ODN_{cx}. [c]Determined by chiral HPLC

Entries 1-4: Pyrimidine (C) outperforms purine (G) at both positions 6 and 17

Entry 5: Confirms that purines (A) at positions 6 and 17 is detrimental.

Entrries 6-7: C is superior to T in both positions 6 and 17

VI. [2+2] Photocycloaddition

1. General procedure for racemic [2+2] photocycloaddition

General procedure E

A 2 mL vial was charged with quinolone (0.1 mmol, 1 equiv.) and photocatalyst (1 µmol, 0.01 equiv.), then THF was added (0.1 M according to substrate). The mixture was irradiated (λ_{max} = 450 nm) for 14 – 16 h at 25 °C. Solvent was removed under reduced pressure and the crude was purified by flash chromatography (cyclohexane/acetone 8:2) to give racemate product.

2. General procedure for enantioselective [2+2] photocycloaddition

General procedure F

A 1 mL glass vial was charged with H_2O (X µL, V_{tot} = 100 µL, X = 100 – ($V_{DNAzyme}$+ $V_{conter\ strand}$ + V_{buffer} + V_{NaCl} + $V_{quinolone}$)), DNAzyme (1 mol%, 1 nmol, 10 µM) and counter-strand (1.5 mol%, 1.5 nmol, 15 µM) in a MOPS buffer pH 7.5 (10 µL from a 200 mM solution in milli-Q H_2O, C_f = 20 mM), NaCl (20 µL from a 5 M solution in milli-Q H_2O, C_f = 1 M). The reaction mixture was briefly mixed, heated at 90 °C for 5 min and allowed to cool down at room temperature. Quinolone (2 µL from a 50 mM solution in THF, 100 nmol, C_f = 1 mM) were then added. The mixture was degassed by bubbling argon for 3 minutes and the vial was placed under irradiation (I_{max} = 450 nm) and stirred for 14–16h at 5 °C. The mixture was then transferred into 2 mL Eppendorf®. The reaction vial was rinsed with milli-Q H_2O (500 µL) and diethyl ether (500 µL). The organic phase was poured into a new 2 mL Eppendorf®. The process was repeated twice (2 x 500 µL diethyl ether). After evaporation of diethyl ether, the sample was dissolved in MeOH (100 µL), transferred in HPLC mini vials and injected in chiral HPLC to measure conversion and enantiomeric ratio.

3. Characterization of cycloaddition products

(1aR,4aS,10bR)-1,1a,2,3,6,10b-hexahydro-5H-furo[2',3':1,4]cyclobuta[1,2-c]quinolin-5-one (2a)

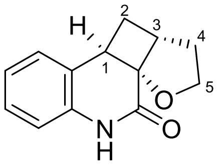

Racemate: 73% isolated yield. Asymmetric catalysis: 97% HPLC conversion, 91.5:8.5 e.r. with **ODN$_1$-S$_L$-[Ir] / ODN$_{C4}$**; 95% HPLC conversion, 95.5:4.5 e.r. with **ODN$_1$-S$_L$-[ΔIr] / ODN$_{C4}$**; 80% HPLC conversion, 4.5:95.5 e.r. with **(L)ODN$_1$-S$_D$-[ΛIr] / (L)ODN$_{C4}$** (Chiralpak IA column, 50 bar, T = 25 °C, *n*-Hexane (0.1 % diethylamine) / *i*-PrOH (0.1 % diethylamine) = 75:25, 1 mL/min, λ = 260 nm, t_{R1} = 7.52 min, t_{R2} = 10.80 min). **^{1}H NMR** (500 MHz, $CDCl_3$) δ 8.58 (s, 1H, NH), 7.19 – 7.10 (m, 1H, CH-Ar), 7.05 – 6.94 (m, 2H, CH-Ar), 6.78 (d, *J* = 7.9 Hz, 1H, CH-Ar), 4.53 (tdd, *J* = 7.7, 2.8, 1.1 Hz, 1H, $^5CH_2$), 4.32 (td, *J* = 9.3, 5.8 Hz, 1H, $^5CH_2$), 3.65 (dd, *J* = 10.1, 5.9 Hz, 1H, ^{1}CH), 3.41 – 3.31 (m, 1H, ^{3}CH), 2.32 – 2.06 (m, 3H, 2 $^2CH_2$ + 1 $^4CH_2$), 1.91 – 1.79 (m, 1H, $^4CH_2$). **^{13}C NMR** (126 MHz, $CDCl_3$) δ 169.3 (C=O), 135.1 (C-Ar), 128.2 (CH-Ar), 127.7 (CH-Ar), 125.5 (C-Ar), 123.8 (CH-Ar), 116.0 (CH-Ar), 81.9 (CO), 70.8 ($^5CH_2$), 45.5 (^{3}CH), 40.3 (^{1}CH), 32.8 ($^4CH_2$), 31.4 ($^2CH_2$). **HRMS** (ESI+) *m/z*: Calcd for $C_{13}H_{14}NO_2$ $[M+H]^+$: 216.1025 found 216.1019.

(1aR,4aS,10bR)-9-fluoro-1,1a,2,3,6,10b-hexahydro-5H-furo[2',3':1,4]cyclobuta[1,2-c]quinolin-5-one (2b)

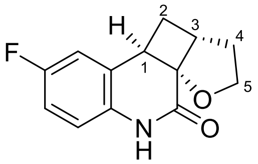

Racemate: 78% isolated yield. Asymmetric catalysis: 74% HPLC conversion, 82.5:17.5 e.r. with **ODN$_1$-S$_L$-[Ir] / ODN$_{C4}$**; 84% HPLC conversion, 90.5:9.5 e.r. with **ODN$_1$-S$_L$-[ΔIr] / ODN$_{C4}$** (Chiralpak IA column, 56 bar, T = 25 °C, *n*-Hexane (0.1 % diethylamine) / *i*-PrOH (0.1 % diethylamine) = 70:30, 1 mL/min, λ = 260 nm, t_{R1} = 6.62 min, t_{R2} = 8.28 min). **^{1}H NMR** (400 MHz, $CDCl_3$) δ 8.73 (s, 1H, NH), 6.85 (td, *J* = 8.4, 2.8 Hz, 1H, CH-Ar), 6.79 – 6.67 (m, 2H, 2 CH-Ar), 4.55 – 4.49 (m, 1H, $^5CH_2$), 4.31 (td, *J* = 9.3, 6.0 Hz, 1H, $^5CH_2$), 3.62 (dd, *J* = 10.3, 6.1 Hz, 1H, ^{1}CH), 3.39 – 3.32 (m, 1H, ^{3}CH), 2.29 – 2.10 (m, 3H, 2 $^2CH_2$ + 1 $^4CH_2$), 1.88 – 1.80 (m, 1H, $^4CH_2$). **^{13}C NMR** (126 MHz, $CDCl_3$) δ 169.3 (C=O), 160.1 (C-Ar), 158.2 (C-Ar), 131.5 (C-Ar), 127.1 (d, J = 7.1 Hz, C-Ar), 117.4 (d, J = 8.4 Hz, CH-Ar), 114.6 (d, J = 6.2 Hz, CH-Ar), 114.4 (d, J = 6.5 Hz, CH-Ar), 81.4 (CO), 70.9 ($^5CH_2$), 45.4 (^{3}CH), 40.4 (^{1}CH), 32.9 ($^4CH_2$), 31.3 ($^2CH_2$). **^{19}F NMR** (377 MHz, $CDCl_3$) δ -119.5. **HRMS** (ESI+) *m/z*: Calcd for $C_{13}H_{13}FNO_2$ $[M+H]^+$: 234.0930 found 234.0931.

(1aR,4aS,10bR)-9-methyl-1,1a,2,3,6,10b-hexahydro-5H-furo[2',3':1,4]cyclobuta[1,2-c]quinolin-5-one (2c)

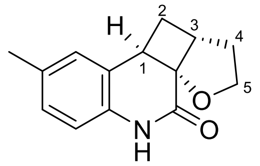

Racemate: 87% isolated yield. Asymmetric catalysis: 76% HPLC conversion, 75.5:24.5 e.r. with **ODN_1-S_L-[Ir] / ODN_{C4}**; 80% HPLC conversion, 83.5:16.5 e.r. with **ODN_1-S_L-[ΔIr] / ODN_{C4}** (Chiralpak IA column, 56 bar, T = 25 °C, *n*-Hexane (0.1 % diethylamine) / *i*-PrOH (0.1 % diethylamine) = 70:30, 1 mL/min, λ = 260 nm, t_{R1} = 7.35 min, t_{R2} = 9.02 min). **^{1}H NMR** (400 MHz, $CDCl_3$) δ 9.00 (s, 1H, NH), 6.92 (d, *J* = 7.3 Hz, 1H, CH-Ar), 6.79 (s, 1H, CH-Ar), 6.72 (d, *J* = 8.0 Hz, 1H, CH-Ar), 4.52 (td, *J* = 8.3, 2.8 Hz, 1H, $^5CH_2$), 4.31 (td, *J* = 9.3, 5.9 Hz, 1H, $^5CH_2$), 3.59 (dd, *J* = 10.0, 5.8 Hz, 1H, ^{1}CH), 3.38 – 3.28 (m, 1H, ^{3}CH), 2.25 (s, 3H, Me), 2.23 – 2.04 (m, 3H, 2 $^2CH_2$ + 1 $^4CH_2$), 1.88 – 1.77 (m, 1H, $^4CH_2$). **^{13}C NMR** (151 MHz, $CDCl_3$) δ 169.4 (C=O), 133.2 (C-Ar), 132.8 (C-Ar), 128.5 (CH-Ar), 128.2 (CH-Ar), 125.3 (C-Ar), 116.1 (CH-Ar), 81.9 (CO), 70.7 ($^5CH_2$), 45.3 (^{3}CH), 40.3 (^{1}CH), 32.8 ($^4CH_2$), 31.5 ($^2CH_2$), 20.8 (Me). **HRMS** (ESI+) *m/z*: Calcd for $C_{14}H_{16}NO_2$ $[M+H]^+$: 230.1181 found 230.1178.

(1aR,4aS,10bR)-9-methoxy-1,1a,2,3,6,10b-hexahydro-5H-furo[2',3':1,4]cyclobuta[1,2-c]quinolin-5-one (2d)

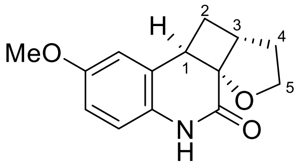

Racemate: 79% isolated yield. Asymmetric catalysis: 81% HPLC conversion, 63.5:36.5 e.r. with **ODN_1-S_L-[Ir] / ODN_{C4}**; 96% HPLC conversion, 70:30 e.r. with **ODN_1-S_L-[ΔIr] / ODN_{C4}** (Chiralpak IA column, 56 bar, T = 25 °C, *n*-Hexane (0.1 % diethylamine) / *i*-PrOH (0.1 % diethylamine) = 75:25, 1 mL/min, λ = 260 nm, t_{R1} = 9.89 min, t_{R2} = 13.40 min). **^{1}H NMR** (600 MHz, $CDCl_3$) δ 8.68 (s, 1H, NH), 6.73 (d, *J* = 8.7 Hz, 1H, CH-Ar), 6.68 (dd, *J* = 8.7, 2.7 Hz, 1H, CH-Ar), 6.53 (d, *J* = 2.7 Hz, 1H, CH-Ar), 4.52 (td, *J* = 8.4, 2.7 Hz, 1H, $^5CH_2$), 4.30 (td, *J* = 9.3, 6.0 Hz, 1H, $^5CH_2$), 3.75 (s, 3H, OMe), 3.60 (dd, *J* = 10.1, 5.9 Hz, 1H, ^{1}CH), 3.40 – 3.29 (m, 1H, ^{3}CH), 2.27 – 2.08 (m, 3H, 2 $^2CH_2$ + 1 $^4CH_2$), 1.86 – 1.79 (m, 1H, $^4CH_2$). **^{13}C NMR** (151 MHz, $CDCl_3$) δ 168.9 (C=O), 156.1 (COMe), 128.8 (C-Ar), 126.7 (C-Ar), 117.0 (CH-Ar), 113.2 (CH-Ar), 113.1 (CH-Ar), 81.7 (CO), 70.8 ($^5CH_2$), 55.7 (OMe), 45.2 (^{3}CH), 40.6 (^{1}CH), 32.8 ($^4CH_2$), 31.4 ($^2CH_2$). **HRMS** (ESI+) *m/z*: Calcd for $C_{14}H_{16}NO_3$ $[M+H]^+$: 246.1130 found 246.1127.

(1aR,4aS,10bR)-9-chloro-1,1a,2,3,6,10b-hexahydro-5H-furo[2',3':1,4]cyclobuta[1,2-c]quinolin-5-one (2e)

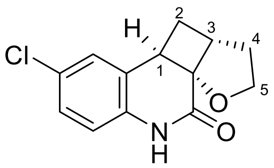

Racemate: 99% isolated yield. Asymmetric catalysis: 84% HPLC conversion, 64:36 e.r. with **ODN$_1$-S$_L$-[Ir] / ODN$_{C4}$**; 87% HPLC conversion, 72:28 e.r. with **ODN$_1$-S$_L$-[ΔIr] / ODN$_{C4}$** (Chiralpak IA column, 45 bar, T = 25 °C, *n*-Hexane (0.1 % diethylamine) / *i*-PrOH (0.1 % diethylamine) = 90:10, 1 mL/min, λ = 260 nm, t_{R1} = 15.65 min, t_{R2} = 20.51 min). **^{1}H NMR** (400 MHz, $CDCl_3$) δ 8.00 (s, 1H, NH), 7.14 – 7.08 (m, 1H, CH-Ar), 7.00 (d, *J* = 2.2 Hz, 1H, CH-Ar), 6.67 (d, *J* = 8.5 Hz, 1H, CH-Ar), 4.52 (ddd, *J* = 8.9, 7.8, 2.9 Hz, 1H, $^5CH_2$), 4.31 (td, *J* = 9.3, 6.0 Hz, 1H, $^5CH_2$), 3.61 (dd, *J* = 10.1, 5.9 Hz, 1H, ^{1}CH), 3.40 – 3.31 (m, 1H, ^{3}CH), 2.32 – 2.08 (m, 3H, 2 $^2CH_2$ + 1 $^4CH_2$), 1.89 – 1.80 (m, 1H, $^4CH_2$). **^{13}C NMR** (126 MHz, MeOD) δ 170.8 (C=O), 135.6 (C-Ar), 129.5 (C-Ar), 128.9 (CH-Ar), 128.8 (C-Ar), 128.6 (CH-Ar), 118.3 (C-Ar), 82.5 (CO), 71.4 ($^5CH_2$), 46.9 (^{3}CH), 41.2 (^{1}CH), 33.4 ($^4CH_2$), 31.9 ($^2CH_2$). **HRMS** (ESI+) *m/z*: Calcd for $C_{13}H_{13}ClNO_2$ $[M+H]^+$: 250.0635 found 250.0626.

(1aR,4aS,10bR)-9-bromo-1,1a,2,3,6,10b-hexahydro-5H-furo[2',3':1,4]cyclobuta[1,2-c]quinolin-5-one (2f)

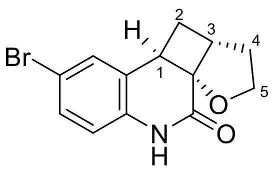

Racemate: 59% isolated yield. Asymmetric catalysis: 99% HPLC conversion, 57:43 e.r. with **ODN$_1$-S$_L$-[Ir] / ODN$_{C4}$**; 93% HPLC conversion, 64:36 e.r. with **ODN$_1$-S$_L$-[ΔIr] / ODN$_{C4}$** (Chiralpak IE column, 56 bar, T = 25 °C, *n*-Hexane (0.1 % diethylamine) / *i*-PrOH (0.1 % diethylamine) = 70:30, 1 mL/min, λ = 260 nm, t_{R1} = 12.75 min, t_{R2} = 15.28 min). **^{1}H NMR** (400 MHz, $CDCl_3$) δ 7.93 (s, 1H, NH), 7.26 – 7.23 (m, 1H, CH-Ar), 7.14 (d, *J* = 2.2 Hz, 1H, CH-Ar), 6.61 (d, *J* = 8.5 Hz, 1H, CH-Ar), 4.52 (ddd, *J* = 9.0, 7.8, 2.9 Hz, 1H, $^5CH_2$), 4.31 (td, *J* = 9.4, 5.9 Hz, 1H, $^5CH_2$), 3.61 (dd, *J* = 10.0, 5.9 Hz, 1H, ^{1}CH), 3.42 – 3.30 (m, 1H, ^{3}CH), 2.32 – 2.09 (m, 3H, 2 $^2CH_2$ + 1 $^4CH_2$), 1.89 – 1.80 (m, 1H, $^4CH_2$). **^{13}C NMR** (126 MHz, $CDCl_3$) δ 169.5 (C=O), 134.4 (CN-Ar), 130.8 (CH-Ar), 130.6 (CH-Ar), 127.5 (C-Ar), 117.9 (CH-Ar), 116.0 (CBr-Ar), 81.5 (CO), 70.9 ($^5CH_2$), 45.6 (^{3}CH), 40.1 (^{1}CH), 32.8 ($^4CH_2$), 31.3 ($^2CH_2$). **HRMS** (ESI+) *m/z*: Calcd for $C_{13}H_{13}BrNO_2$ $[M+H]^+$: 294.0130 found 294. 0124.

(1aR,4aS,10bR)-8-fluoro-1,1a,2,3,6,10b-hexahydro-5H-furo[2',3':1,4]cyclobuta[1,2-c]quinolin-5-one (2g)

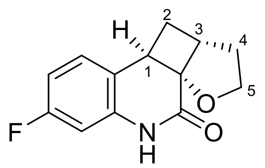

Racemate: 74% isolated yield. Asymmetric catalysis: 87% HPLC conversion, 85:15 e.r. with **ODN$_1$-S$_L$-[Ir] / ODN$_{C4}$**; 86% HPLC conversion, 93:7 e.r. with **ODN$_1$-S$_L$-[ΔIr] / ODN$_{C4}$**; >99% HPLC conversion, 8:92 e.r. with **(L)ODN$_1$-S$_D$-[ΛIr] / (L)ODN$_{C4}$** (Chiralpak IA column, 56 bar, T = 25 °C, *n*-Hexane (0.1 % diethylamine) / *i*-PrOH (0.1 % diethylamine) = 70:30, 1 mL/min, λ = 260 nm, t_{R1} = 6.15 min, t_{R2} = 8.08 min). **^{1}H NMR** (500 MHz, $CDCl_3$) δ 9.00 (s, 1H, NH), 6.94 (dd, *J* = 8.4, 6.0 Hz, 1H, CH-Ar), 6.68 (td, *J* = 8.4, 2.4 Hz, 1H, CH-Ar), 6.58 (dd, *J* = 9.5, 2.6 Hz, 1H, CH-Ar), 4.53 (ddd, *J* = 9.0, 7.9, 3.0 Hz, 1H, $^5CH_2$), 4.32 (td, *J* = 9.2, 6.0 Hz, 1H, $^5CH_2$), 3.60 (dd, *J* = 9.4, 5.9 Hz, 1H, ^{1}CH), 3.42 – 3.33 (m, 1H, ^{3}CH), 2.30 – 2.15 (m, 2H, 1 $^2CH_2$ + 1 $^4CH_2$), 2.14 – 2.04 (m, 1H, $^2CH_2$), 1.89 – 1.81 (m, 1H, $^4CH_2$). **^{13}C NMR** (126 MHz, $CDCl_3$) δ 169.7 (C=O), 163.1 (C-Ar), 161.1 (C-Ar), 136.6 (d, *J* = 10.1 Hz, C-Ar), 129.5 (d, *J* = 9.5 Hz, CH-Ar), 121.3 (d, *J* = 3.7 Hz, C-Ar), 110.6 (d, *J* = 21.7 Hz, CH-Ar), 103.4 (d, *J* = 25.5 Hz, CH-Ar), 81.7 (CO), 71.0 ($^5CH_2$), 45.5 (^{3}CH), 39.8 (^{1}CH), 32.9 ($^4CH_2$), 31.5 ($^2CH_2$). **^{19}F NMR** (376 MHz, $CDCl_3$) δ -114.48. **HRMS** (ESI+) *m/z*: Calcd for $C_{13}H_{13}FNO_2$ $[M+H]^+$: 234.0930 found 234.0924.

(1aR,4aS,10bR)-8-chloro-1,1a,2,3,6,10b-hexahydro-5H-furo[2',3':1,4]cyclobuta[1,2-c]quinolin-5-one (2h)

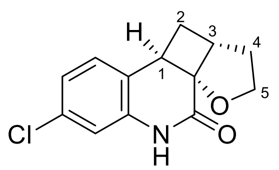

Racemate: 92% isolated yield. Asymmetric catalysis: 77% HPLC conversion, 67:33 e.r. with **ODN$_1$-S$_L$-[Ir] / ODN$_{C4}$**; 86% HPLC conversion, 78.5:21.5 e.r. with **ODN$_1$-S$_L$-[ΔIr] / ODN$_{C4}$** (Chiralpak IA column, 56 bar, T = 25 °C, *n*-Hexane (0.1 % diethylamine) / *i*-PrOH (0.1 % diethylamine) = 70:30, 1 mL/min, λ = 260 nm, t_{R1} = 6.56 min, t_{R2} = 8.70 min). **^{1}H NMR** (400 MHz, $CDCl_3$) δ 8.94 (s, 1H, NH), 6.99 – 6.88 (m, 2H, CH-Ar), 6.84 (d, *J* = 1.9 Hz, 1H, CH-Ar), 4.53 (ddd, *J* = 8.9, 7.8, 2.9 Hz, 1H, $^5CH_2$), 4.32 (td, *J* = 9.3, 6.0 Hz, 1H, $^5CH_2$), 3.60 (dd, *J* = 9.8, 5.7 Hz, 1H, ^{1}CH), 3.41 – 3.30 (m, 1H, ^{3}CH), 2.31 – 2.03 (m, 3H, 2 $^2CH_2$ + 1 $^4CH_2$), 1.89 – 1.79 (m, 1H, $^4CH_2$). **^{13}C NMR** (101 MHz, $CDCl_3$) δ 169.5 (C=O), 136.4 (C-Ar), 133.1 (C-Ar), 129.3 (CH-Ar), 124.0 (C-Ar), 123.8 (CH-Ar), 116.0 (CH-Ar), 81.7 (CO), 71.0 ($^5CH_2$), 45.6 (^{3}CH), 39.9 (^{1}CH), 32.9 ($^4CH_2$), 31.4 ($^2CH_2$). **HRMS** (ESI+) *m/z*: Calcd for $C_{13}H_{13}FNO_2$ $[M+H]^+$: 250.0635 found 250.0637.

(1aR,4aS,10bR)-7-fluoro-1,1a,2,3,6,10b-hexahydro-5H-furo[2',3':1,4]cyclobuta[1,2-c]quinolin-5-one (2i)

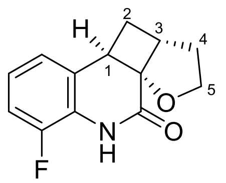

Racemate: 94% isolated yield. Asymmetric catalysis: 88% HPLC conversion, 80:20 e.r. with **ODN_1-S_L-[Ir] / ODN_{C4}**; 76% HPLC conversion, 89.5:10.5 e.r. with **ODN_1-S_L-[ΔIr] / ODN_{C4}** (Chiralpak IA column, 56 bar, T = 25 °C, *n*-Hexane (0.1 % diethylamine) / *i*-PrOH (0.1 % diethylamine) = 70:30, 1 mL/min, λ = 260 nm, t_{R1} = 7.54 min, t_{R2} = 10.52 min). **^{1}H NMR** (400 MHz, $CDCl_3$) δ 7.73 (s, 1H, NH), 6.96 – 6.88 (m, 2H, CH-Ar), 6.82 – 6.75 (m, 1H, CH-Ar), 4.51 (ddd, *J* = 8.9, 7.8, 2.8 Hz, 1H, $^5CH_2$), 4.31 (td, *J* = 9.3, 6.0 Hz, 1H, $^5CH_2$), 3.66 (dd, *J* = 10.0, 5.8 Hz, 1H, ^{1}CH), 3.40 – 3.30 (m, 1H, ^{3}CH), 2.31 – 2.21 (m, 1H, $^2CH_2$), 2.21 – 2.08 (m, 2H, 1 $^2CH_2$ + 1 $^4CH_2$), 1.83 (m, 1H, $^4CH_2$). **^{13}C NMR** (101 MHz, $CDCl_3$) δ 167.9 (C=O), 150.7 (C-Ar), 148.3 (C-Ar), 127.6 (d, *J* = 1.4 Hz, C-Ar), 123.5 (d, *J* = 7.6 Hz, CH-Ar), 123.4 (d, *J* = 3.4 Hz, CH-Ar), 113.6 (d, *J* = 18.1 Hz, CH-Ar), 81.8 (CO), 71.0 ($^5CH_2$), 45.5 (^{3}CH), 40.5 (d, *J* = 2.3 Hz, ^{1}CH), 32.8 ($^4CH_2$), 31.4 ($^2CH_2$). **^{19}F NMR** (376 MHz, $CDCl_3$) δ -135.44. **HRMS** (ESI+) *m/z*: Calcd for $C_{13}H_{13}FNO_2$ $[M+H]^+$: 234.0930 found 234.0925.

(1aR,4aS,10bR)-7-methyl-1,1a,2,3,6,10b-hexahydro-5H-furo[2',3':1,4]cyclobuta[1,2-c]quinolin-5-one (2j)

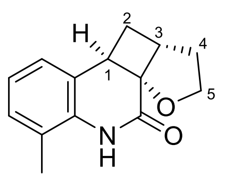

Racemate: 78% isolated yield. Asymmetric catalysis: 84% HPLC conversion, 74:26 e.r. with **ODN_1-S_L-[Ir] / ODN_{C4}**; 94 % HPLC conversion, 82:18 e.r. with **ODN_1-S_L-[ΔIr] / ODN_{C4}** (Chiralpak IB column, 56 bar, T = 25 °C, *n*-Hexane (0.1 % diethylamine) / *i*-PrOH (0.1 % diethylamine) = 55:45, 1 mL/min, λ = 260 nm, t_{R1} = 6.47 min, t_{R2} = 7.21 min). **^{1}H NMR** (600 MHz, $CDCl_3$) δ 7.45 (s, 1H, NH), 7.04 – 6.98 (m, 1H, CH-Ar), 6.94 – 6.85 (m, 2H, CH-Ar), 4.52 (ddd, *J* = 8.9, 7.8, 2.7 Hz, 1H, $^5CH_2$), 4.31 (td, *J* = 9.4, 6.0 Hz, 1H, $^5CH_2$), 3.64 (dd, *J* = 10.1, 5.8 Hz, 1H, ^{1}CH), 3.39 – 3.31 (m, 1H, ^{3}CH), 2.29 – 2.25 (m, 1H, 1 $^2CH_2$), 2.24 (s, 3H, Me), 2.21 – 2.15 (m, 1H, $^4CH_2$), 2.14 – 2.07 (m, 1H, $^2CH_2$), 1.86 – 1.80 (m, 1H, $^4CH_2$). **^{13}C NMR** (151 MHz, $CDCl_3$) δ 168.9 (C=O), 133.4 (C-Ar), 129.2 (CH-Ar), 126.2 (CH-Ar), 125.4 (C-Ar), 123.4 (CH-Ar), 122.4 (C-Ar), 81.9 (CO), 70.8 ($^5CH_2$), 45.5 (^{3}CH), 40.4 (^{1}CH), 32.8 ($^4CH_2$), 31.6 ($^2CH_2$), 17.0 (Me). **HRMS** (ESI+) *m/z*: Calcd for $C_{14}H_{16}NO_2$ $[M+H]^+$: 230.1180 found 230.1174.

(4bR,5aR,8aS)-4b,5,5a,6,7,10-hexahydro-9H-furo[2',3':1,4]cyclobuta[1,2-c][1,8]naphthyridin-9-one (2k)

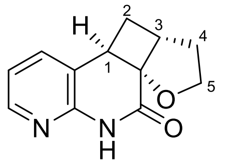

Racemate: 82% isolated yield. Asymmetric catalysis: 92% HPLC conversion, 67.5:32.5 e.r. with **ODN$_1$-S$_L$-[Ir] / ODN$_{C4}$**; 84% HPLC conversion, 85.5:14.5 e.r. with **ODN$_1$-S$_L$-[ΔIr] / ODN$_{C4}$** (Chiralpak IA column, 65 bar, T = 25 °C, *n*-Hexane (0.1 % diethylamine) / *i*-PrOH (0.1 % diethylamine) = 60:40, 1 mL/min, λ = 260 nm, t_{R1} = 10.78 min, t_{R2} = 21.36 min). **^{1}H NMR** (600 MHz, Chloroform-*d*) δ 10.51 (s, 1H, NH), 8.34 (dd, *J* = 5.1, 1.8 Hz, 1H, CH-Ar), 7.36 (d, *J* = 7.5 Hz, 1H, CH-Ar), 6.96 (dd, *J* = 7.6, 5.0 Hz, 1H, CH-Ar), 4.56 – 4.48 (m, 1H, ^{5}CH$_2$), 4.35 – 4.27 (m, 1H, ^{5}CH$_2$), 3.63 – 3.57 (m, 1H, ^{1}CH), 3.41 – 3.33 (m, 1H, ^{3}CH), 2.32 – 2.24 (m, 1H, ^{2}CH$_2$), 2.22 – 2.14 (m, 1H, ^{4}CH$_2$), 2.12 – 2.07 (m, 1H, ^{2}CH$_2$), 1.87 – 1.81 (m, 1H, ^{4}CH$_2$). **^{13}C NMR** (151 MHz, CDCl$_3$) δ 169.5 (C=O), 149.2 (C-Ar), 146.7 (CH-Ar), 136.8 (CH-Ar), 121.1 (C-Ar), 119.3 (CH-Ar), 82.0 (CO), 71.1 (^{5}CH$_2$), 45.6 (^{3}CH), 39.0 (^{1}CH), 32.9 (^{4}CH$_2$), 31.2 (^{2}CH$_2$). **HRMS** (ESI+) *m/z*: Calcd for $C_{12}H_{13}N_2O_2$ [M+H]$^+$: 217.0977 found 217.0969.

(1aR,4aS,10bR)-1,1-dimethyl-1,1a,2,3,6,10b-hexahydro-5H-furo[2',3':1,4]cyclobuta[1,2-c]quinolin-5-one (2l)

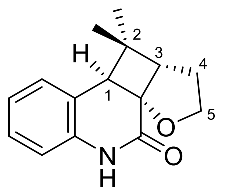

Racemate: 95% isolated yield. Asymmetric catalysis: 67% HPLC conversion, 64:36 e.r. with **ODN$_1$-S$_L$-[Ir] / ODN$_{C4}$**; 80% HPLC conversion, 69:31 e.r. with **ODN$_1$-S$_L$-[ΔIr] / ODN$_{C4}$** (Chiralpak IA column, 65 bar, T = 25 °C, *n*-Hexane (0.1 % diethylamine) / *i*-PrOH (0.1 % diethylamine) = 20:80, 1 mL/min, λ = 260 nm, t_{R1} = 5.22 min, t_{R2} = 10.93 min). **^{1}H NMR** (400 MHz, CDCl$_3$) δ 9.30 (bs, 1H, NH), 7.17 – 7.08 (m, 1H, CH-Ar), 6.96 (t, *J* = 7.4 Hz, 1H, CH-Ar), 6.91 – 6.82 (m, 2H, CH-Ar), 4.45 (ddd, *J* = 9.1, 7.7, 3.1 Hz, 1H, ^{5}CH$_2$), 4.15 (td, *J* = 9.3, 6.5 Hz, 1H, ^{5}CH$_2$), 3.28 (s, 1H, ^{1}CH), 2.97 – 2.90 (ddd, *J* = 9.0, 2.6, 1.3 Hz, 1H, ^{3}CH), 2.18 – 1.99 (m, 2H, ^{4}CH$_2$), 1.15 (s, 3H, CH$_3$), 0.80 (s, 3H, CH$_3$). **^{13}C NMR** (101 MHz, CDCl$_3$) δ 170.7 (bs, C=O), 136.5 (C-Ar), 128.3 (CH-Ar), 127.8 (CH-Ar), 123.5 (CH-Ar), 121.5 (C-Ar), 116.1 (CH-Ar), 78.2 (CO), 70.9 (^{5}CH$_2$), 56.5 (^{3}CH), 50.4 (^{1}CH), 34.9 (^{2}C), 27.8 (^{4}CH$_2$), 26.4 (CH$_3$), 24.7 (CH$_3$). **HRMS** (ESI+) *m/z*: Calcd for $C_{15}H_{18}NO_2$ [M+H]$^+$: 244.1338 found 244.1343.

(1aR,4aS,10bR)-1a-methyl-1,1a,2,3,6,10b-hexahydro-5H-furo[2',3':1,4]cyclobuta[1,2-c]quinolin-5-one (2m)

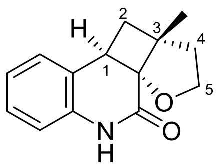

Racemate: 92% isolated yield. Asymmetric catalysis: 66% HPLC conversion, 65.5:34.5 e.r. with **ODN$_1$-S$_L$-[Ir] / ODN$_{C4}$**; 73% HPLC conversion, 77.5:22.5 e.r. with **ODN$_1$-S$_L$-[ΔIr] / ODN$_{C4}$** (Chiralpak IA column, 56 bar, T = 25 °C, *n*-Hexane (0.1 % diethylamine) / *i*-PrOH (0.1 % diethylamine) = 70:30, 1 mL/min, λ = 260 nm, t_{R1} = 6.73 min, t_{R2} = 9.74 min). **^{1}H NMR** (400 MHz, $CDCl_3$) δ 9.28 (s, 1H, NH), 7.17 – 7.04 (m, 1H, CH-Ar), 7.03 – 6.92 (m, 2H, CH-Ar), 6.85 (d, *J* = 7.9 Hz, 1H, CH-Ar), 4.51 – 4.40 (m, 1H, $^5CH_2$), 4.28 – 4.19 (m, 1H, $^5CH_2$), 3.56 (dd, *J* = 10.2, 5.6 Hz, 1H, ^{1}CH), 2.50 (dd, *J* = 12.4, 10.2 Hz, 1H, $^2CH_2$), 1.93 – 1.82 (m, 2H, $^4CH_2$), 1.78 (dd, *J* = 12.4, 5.6 Hz, 1H, $^2CH_2$), 1.28 (s, 3H, CH_3). **^{13}C NMR** (101 MHz, $CDCl_3$) δ 167.7 (C=O), 135.2 (C-Ar), 128.0 (CH-Ar), 127.5 (CH-Ar), 125.9 (C-Ar), 123.7 (CH-Ar), 116.2 (CH-Ar), 84.0 (CO), 69.6 ($^5CH_2$), 52.1 (^{3}C), 40.4 ($^4CH_2$), 38.9 ($^2CH_2$), 38.1 (^{1}CH), 22.5 (CH_3). **HRMS** (ESI+) *m/z*: Calcd for $C_{14}H_{16}NO_2$ $[M+H]^+$: 230.1181 found 230.1175.

(1aR,3R,4aS,10bR)-3-methyl-1,1a,2,3,6,10b-hexahydro-5H-furo[2',3':1,4]cyclobuta[1,2-c]quinolin-5-one (2n)

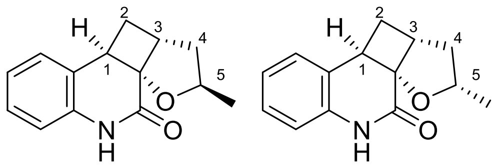

Racemate: 70% isolated yield, 2.5:1 diastereoisomeric ratio. Asymmetric catalysis: 98% HPLC conversion, 1:3 diastereoisomeric ratio, 82:18 e.r.$_1$, 63:37 e.r.$_2$ with **ODN$_1$-S$_L$-[Ir] / ODN$_{C4}$**; 95% HPLC conversion, 1:1.8 diastereoisomeric ratio, 93:7 e.r.$_1$, 78:22 e.r.$_2$ with **ODN$_1$-S$_L$-[ΔIr] / ODN$_{C4}$** (Chiralpak IA column, 36 bar, T = 25 °C, *n*-Hexane (0.1 % diethylamine) / *i*-PrOH (0.1 % diethylamine) = 90:10, 1 mL/min, λ = 260 nm, t_{R1} = 12.72 min, t_{R2} = 16.54 min, t_{R3} = 23.45 min, t_{R4} = 34.48 min). **^{1}H NMR** (500 MHz, $CDCl_3$) δ 8.82 (s, 1H, NH_{maj}), 8.75 (s, 0.4H, NH_{min}), 7.16 – 7.09 (m, 1.3H, CH_{maj} + CH_{min}), 7.02 – 6.93 (m, 2.6H, 2 CH_{maj} + 2 CH_{min}), 6.83 – 6.74 (m, 1.3H, CH_{maj} + CH_{min}), 4.76 – 4.62 (m, 1.4H, $^5CH_{maj}$ + $^5CH_{min}$), 3.70 (dd, *J* = 10.1, 6.0 Hz, 0.5H, $^1CH_{min}$), 3.64 (dd, *J* = 9.5, 6.7 Hz, 1H, $^1CH_{maj}$), 3.39 – 3.33 (m, 0.4H, $^3CH_{min}$), 3.31 – 3.23 (m, 1H, $^3CH_{maj}$), 2.52 (ddd, *J* = 12.4, 8.9, 5.6 Hz, 1H, $^4CH_{2\,maj}$), 2.31 (ddd, *J* = 12.1, 9.6, 4.4 Hz, 1H, $^2CH_{2\,maj}$), 2.25 (ddd, *J* = 12.5, 10.1, 5.5 Hz, 0.4H, $^2CH_{2\,min}$), 2.12 (ddd, *J* = 12.1, 8.6, 6.5 Hz, 1H, $^2CH_{2\,maj}$), 2.10 – 2.05 (m, 0.4H, $^2CH_{2\,min}$), 1.89 (dd, *J* = 12.2, 5.0 Hz, 0.5H, $^4CH_{2\,min}$), 1.72 (ddd, *J* = 12.5, 10.1, 7.8 Hz, 0.5H, $^4CH_{2\,min}$), 1.63 (ddd, *J* = 12.5, 9.5, 6.2 Hz, 1H, $^4CH_{2\,maj}$), 1.51 (d, *J* = 6.1 Hz, 1H, $CH_{3\,min}$), 1.47 (d, *J* = 6.1 Hz, 3H, $CH_{3\,maj}$). **^{13}C NMR** (101 MHz, $CDCl_3$) δ 171.1 (C=O$_{maj}$), 169.0 (C=O$_{min}$), 135.4 (C-Ar$_{maj}$), 135.3 (C-Ar$_{min}$), 128.1 (CH-Ar$_{min}$), 128.0 (CH-Ar$_{maj}$), 127.6 (CH-Ar$_{maj}$ + CH-Ar$_{min}$), 125.6 (C-Ar$_{min}$), 125.5 (C-Ar$_{maj}$), 123.7 (CH-Ar$_{min}$), 123.6

(CH-Ar$_{maj}$), 116.0 (CH-Ar$_{maj}$), 115.9 (CH-Ar$_{min}$), 83.2 (^{5}CH$_{maj}$), 82.2 (CO$_{maj}$), 82.0 (CO$_{min}$), 77.7 (^{5}CH$_{min}$), 46.5 (^{3}CH$_{min}$), 45.2 (^{3}CH$_{maj}$), 43.3 (^{4}CH$_{2\ maj}$), 41.0 (^{1}CH$_{min}$), 40.9 (^{1}CH$_{maj}$), 40.0 (^{4}CH$_{2\ min}$), 32.6 (^{2}CH$_{2\ maj}$), 31.4 (^{2}CH$_{2\ min}$), 21.2 (CH$_{3\ maj}$), 19.9 (CH$_{3\ min}$). **HRMS** (ESI+) *m/z*: Calcd for $C_{14}H_{16}NO_2$ [M+H]$^+$: 230.1182 found 230.1175.

(1aR,4aS,10bR)-6-methyl-1,1a,2,3,6,10b-hexahydro-5H-furo[2',3':1,4]cyclobuta[1,2-c]quinolin-5-one (2o)

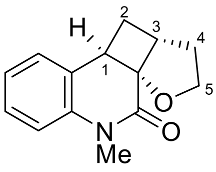

Racemate: 71% isolated yield. Asymmetric catalysis: 72% HPLC conversion, 75:25 e.r. with **ODN$_1$-S$_L$-[Ir] / ODN$_{C4}$**; 88% HPLC conversion, 88:12 e.r. with **ODN$_1$-S$_L$-[ΔIr] / ODN$_{C4}$**; 92% HPLC conversion, 12.5:87.5 e.r. with **(L)ODN$_1$-S$_D$-[ΛIr] / (L)ODN$_{C4}$** (Chiralpak IA column, 36 bar, T = 25 °C, *n*-Hexane (0.1 % diethylamine) / *i*-PrOH (0.1 % diethylamine) = 90:10, 1 mL/min, λ = 260 nm, t_{R1} = 10.20 min, t_{R2} = 12.84 min). **^{1}H NMR** (600 MHz, $CDCl_3$) δ 7.25 – 7.20 (m, 1H, CH-Ar), 7.05 – 7.01 (m, 2H, CH-Ar), 6.98 (d, *J* = 8.4 Hz, 1H, CH-Ar), 4.50 (td, *J* = 8.0, 3.5 Hz, 1H, ^{5}CH$_2$), 4.31 (td, *J* = 8.9, 6.2 Hz, 1H, ^{5}CH$_2$), 3.62 (dd, *J* = 9.5, 6.0 Hz, 1H, ^{1}CH), 3.40 (s, 3H, NMe), 3.28 – 3.20 (m, 1H, ^{3}CH), 2.26 – 2.16 (m, 2H, 1 ^{2}CH$_2$ + 1 ^{4}CH$_2$), 2.12 – 2.05 (m, 1H, ^{2}CH$_2$), 1.87 – 1.81 (m, 1H, ^{4}CH$_2$). **^{13}C NMR** (151 MHz, $CDCl_3$) δ 168.4 (C=O), 138.2 (C-Ar), 128.5 (CH-Ar), 127.6 (CH-Ar), 126.6 (C-Ar), 123.5 (CH-Ar), 114.9 (CH-Ar), 81.5 (CO), 71.0 (^{5}CH$_2$), 45.3 (^{3}CH), 39.6 (^{1}CH), 33.0 (^{4}CH$_2$), 31.4 (^{2}CH$_2$), 29.9 (NMe). **HRMS** (ESI+) *m/z*: Calcd for $C_{14}H_{16}NO_2$ [M+H]$^+$: 230.1181 found 230.1176.

(1aR,4aS,10bR)-6-ethyl-1,1a,2,3,6,10b-hexahydro-5H-furo[2',3':1,4]cyclobuta[1,2-c]quinolin-5-one (2p)

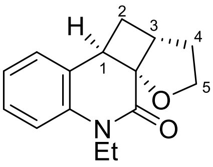

Racemate: 54% isolated yield. Asymmetric catalysis: 93% HPLC conversion, 70:30 e.r. with **ODN$_1$-S$_L$-[Ir] / ODN$_{C4}$**; 88% HPLC conversion, 79.5:20.5 e.r. with **ODN$_1$-S$_L$-[ΔIr] / ODN$_{C4}$** (Chiralpak IA column, 56 bar, T = 25 °C, *n*-Hexane (0.1 % diethylamine) / *i*-PrOH (0.1 % diethylamine) = 70:30, 1 mL/min, λ = 260 nm, t_{R1} = 6.97 min, t_{R2} = 7.52 min). **^{1}H NMR** (600 MHz, $CDCl_3$) δ 7.24 – 7.20 (m, 1H, CH-Ar), 7.03 – 7.01 (m, 2H, CH-Ar), 6.98 (d, *J* = 8.4 Hz, 1H, CH-Ar), 4.49 (ddd, *J* = 8.8, 7.7, 3.7 Hz, 1H, ^{5}CH$_2$), 4.35 – 4.29 (m, 1H, ^{5}CH$_2$), 4.12 (dq, *J* = 14.2, 7.2 Hz, 1H, **CH$_2$**-CH$_3$), 3.95 (dq, *J* = 14.2, 7.1 Hz, 1H, **CH$_2$**-CH$_3$), 3.60 (ddd, *J* = 9.7, 5.8, 1.2 Hz, 1H, ^{1}CH), 3.28 – 3.19 (m, 1H, ^{3}CH), 2.26 – 2.16 (m, 2H, 1 ^{2}CH$_2$ + 1 ^{4}CH$_2$), 2.11 – 2.05 (m, 1H, ^{2}CH$_2$), 1.86 – 1.79 (m, 1H, ^{4}CH$_2$), 1.27 (t, *J* = 7.1 Hz, 3H, CH$_2$-**CH$_3$**). **^{13}C NMR** (151 MHz, $CDCl_3$) δ 167.9 (C=O), 136.8 (C-Ar), 128.9 (CH-Ar), 127.7 (CH-Ar), 126.9 (C-Ar), 123.3 (CH-Ar), 114.9 (CH-Ar), 81.4 (CO), 71.2 (^{5}CH$_2$), 45.3 (^{3}CH), 39.6 (^{1}CH), 37.4 (**CH$_2$**-CH$_3$), 33.1 (^{4}CH$_2$), 31.5 (^{2}CH$_2$), 12.5 (CH$_2$-**CH$_3$**). **HRMS** (ESI+) *m/z*: Calcd for $C_{15}H_{18}NO_2$ [M+H]$^+$: 244.1338 found 244.1332.

4. Scope of visible light TTEnT [2+2] cycloaddition.

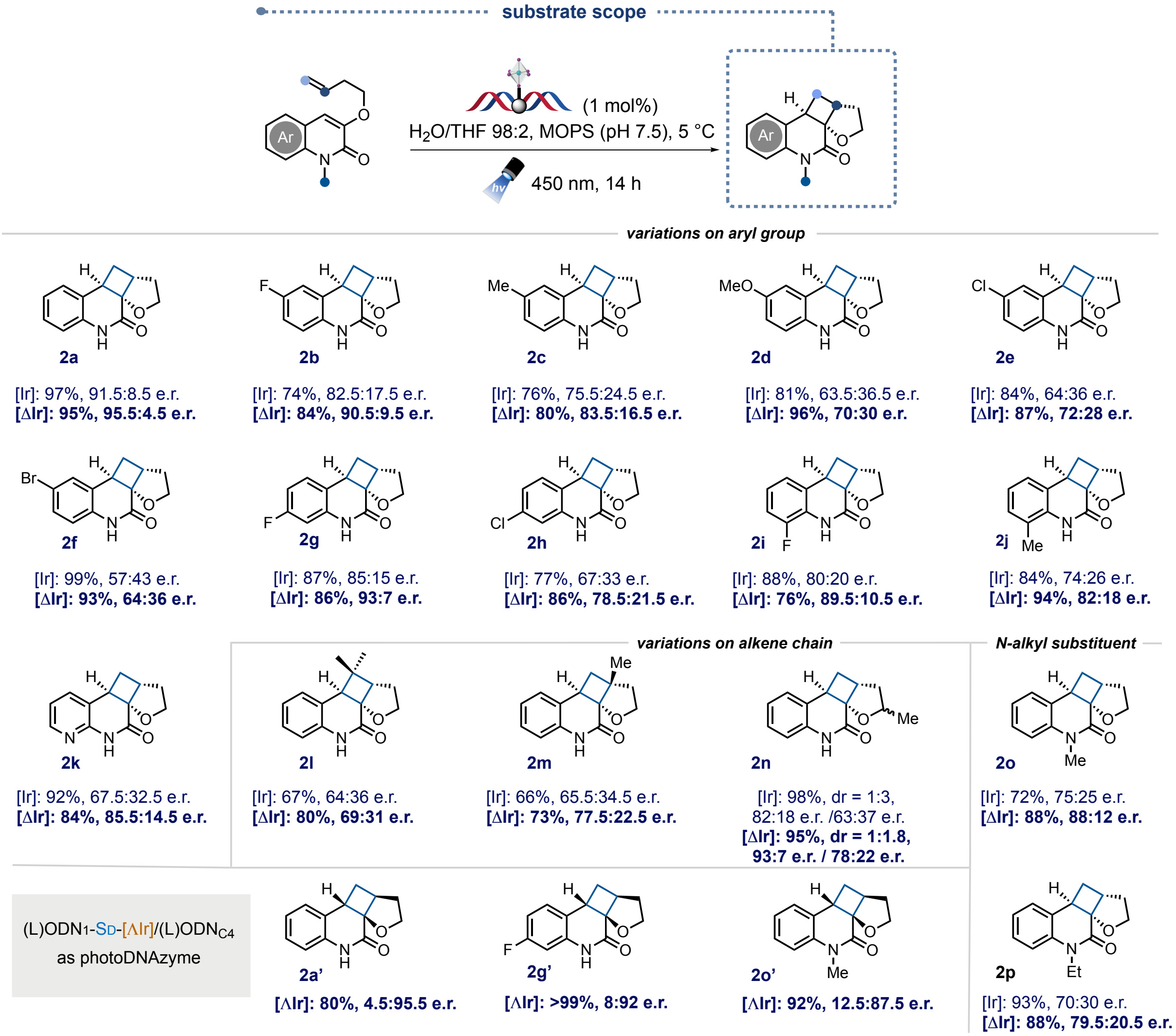

***Figure S3** Scope using either ODN$_1$-S$_L$-[Ir]/ODN$_{C4}$, ODN$_1$-S$_L$-[ΔIr]/ODN$_{C4}$ or ODN$_1$-S$_D$-[ΛIr]/ODN$_{C4}$*

VII. Molecular dynamics simulations

Molecular dynamics protocol for the C18-containing oligonucleotides

A PDB structure for the 12-bp self-complementary DNA sequence $^{5'}$GCCAGCGGACCG$^{3'}$ with canonical B-DNA angles was generated with the NAB (Nucleic Acid Builder) auxiliary package of the Amber2022 suite of programs.[7] The central guanine G7 nucleobase was removed and replaced manually by the serinol modification bearing the iridium complex to produce ODN$_1$-S$_L$-[ΔIr] and ODN$_1$-S$_L$-[ΛIr]: an initial structure was taken from the X-ray structure for the [ΛIr] enantiomer, and the [ΔIr] enantiomer was generated as the mirror image of [ΛIr]. The structures shown in Figure S4 were subsequently optimized at the DFT/ωB97XD/6-311+G(d,p)/LANL2DZ level of theory –using a range-separated hybrid functional and the Los Alamos National Laboratory 2-double ζ LANL2DZ pseudo-potential for the iridium with the Gaussian 16 Rev. B.01 suite of programs.[8] Classical force field parameters for both enantiomers were generated following relying the python MPCB.py protocol proposed by Li and Merz.[9] It was verified that force field parameters (notably atomic charges) were identical for the two enantiomers of the iridium complex.

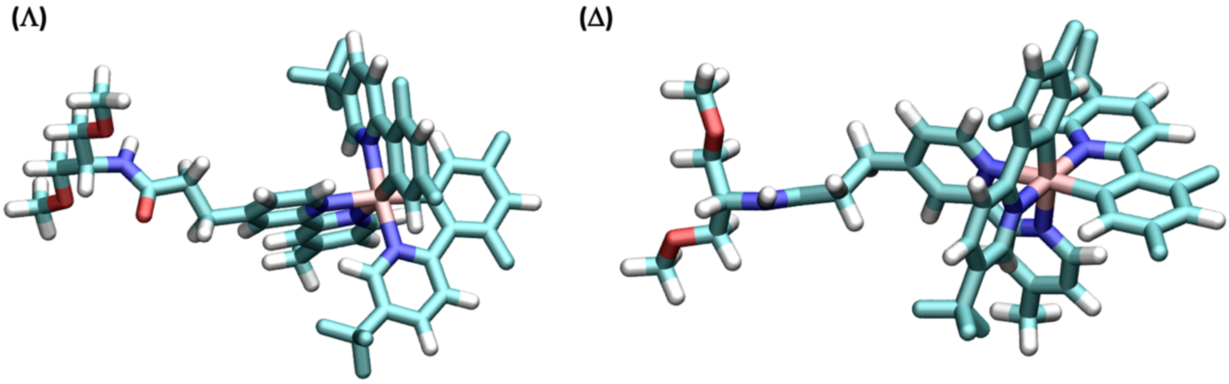

Figure S4. *Optimized structures for S$_L$-[ΛIr] and S$_L$-[ΔIr]: phosphate groups were then added to define this groupment as a non-canonical nucleobases.*

The ODN$_1$-S$_L$-[ΔIr]/ODN$_{C4}$ and ODN$_1$-S$_L$-[ΛIr]/ODN$_{C4}$ duplexes were immersed in TIP3P water boxes: dimensions are listed in Table S9. Potassium and chlorine ions were added to enforce an ionic concentration of 0.15 M. All MD simulations were performed with the Amber and Ambertools 2022 packages.[7] The ff14SB force field[10] was used together with the bsc1 corrections for the nucleic acids.[11] Starting structures for both duplexes were first minimized through 5,000 steps of steepest descent with restraints on the iridium complex of 50 kcal.mol^{-1}. Å^2 followed by 5,000 steps of conjugate gradients. The system was then heated from 0 to 300 K in a 60 ps thermalization run with no restraints. The temperature was subsequently maintained at 300 K along the simulation using the Langevin thermostat with a collision

frequency γln of 1 ps^{-1}. The system was then equilibrated during 10 ns in the NpT ensemble. Finally, production run was carried out for a total of 20 trajectories of 500ns (10 μs in total) for each enantiomer to ensure a large statistical sampling. The timestep was set throughout all simulations to 2 fs using the SHAKE algorithm for integration. Additionally, a 12 Å cut-off was used for non-bonded interactions, and the particle-mesh Ewald method treated long-range interactions.

Clustering of MD ensembles was carried out according to the RMSD (K-means algorithm) of the 12-bp sequence harboring the modified central nucleobase. Post-processing and structural analyses were carried out using AmberTools 2022 and Curves+ programs to identify the four most representative structures which are shown in Figure S5. The NonCovalent Interaction (NCI)[12] analysis, which is a topological tool based on the density (P) and its derivatives to visualize notably π-stacking interactions, on the most representative binding pocket clusters was performed to visualize non-covalent interactions taking place within the photoDNAzyme structures (Figure S6) and the default visualization parameters (density isosurface cut-off set up to 0.3 as the default value) were used to plot the isodensities with the VMD software.

Classical force field parameters were generated through the antechamber module for the substrate (**1a**), relying on the generalized Amber force field (GAFF). Starting structures for each of the representative structures of the photoDNAzymes (with [Λlr] or [Δlr]) and a substrate **1a** molecule placed far from the duplex (at least at 15 Å to avoid biasing the simulations). Systems were immersed in a TIP3P water box for the ODN_1-S_L-[ΔIr]/ODN_{C4} and ODN_1-S_L-[ΛIr]/ODN_{C4} respectively. A similar minimization—heating—equilibration protocol was applied, and production runs of 3 independent replica of 500 ns were performed and post-processed with the same computational protocol. The most stable positioning of **1a** is with ODN_1-S_L-[ΔIr]/ODN_{C4} and is shown in Figure S7a. Substrate **1a** is intercalating the two adjacent C17 and C6 bases in the major groove as allowed by the extrahelical flipping of C18. It can be compared with randomized transient π-stacking between the substrate **1a** and the iridium complex of ODN_1-S_L-[ΛIr]/ODN_{C4} as shown in Figure S7b.

For the ODN_1-S_L-[ΔIr]/ODN_{C4} photoDNazyme, the stability of two additional orientations of the substrate **1a** within the binding pocket were assessed computationally along independent trajectories (3 replica of 100ns each). Structures shown in Figure S8 illustrate that the most stable orientation of the substrate **1a** involves a π-stacking between its homoallyl moiety and the flipped C18 ring, and an interaction between CH and CF_3. The positioning shown rightside in Figure S8 (see also Figure S7a and Figure 5B in the main text) was found to be stable over 1 μs of unbiased MD simulations.

Table S9 *Cluster analysis based on the RMSD over the 5*100 ns concatenated molecular dynamics trajectories of the photoDNAzyme (in absence of substrate).*

	**ODN_1-S_L-[ΔIr]/ODN_{C4}**		**ODN_1-S_L-[ΛIr]/ODN_{C4}**	
Cluster	Fraction	Interaction mode	Fraction	Interaction mode
1	0.208	Minor groove	0.223	Minor groove
2	0.163	Major groove	0.196	Major groove
3	0.136	Solvent exposed	0.192	Major groove
4	0.117	Major groove	0.123	Major groove
5	0.112	Minor groove	0.082	Major groove
6	0.107	Minor groove	0.074	Solvent exposed
7	0.067	Major groove	0.066	Solvent exposed
8	0.050	Minor groove	0.035	Minor groove
9	0.039	Solvent exposed	0.008	Lateral interaction with G12:C13 terminal base pair
10	0.002	Minor Groove	0.001	Major groove

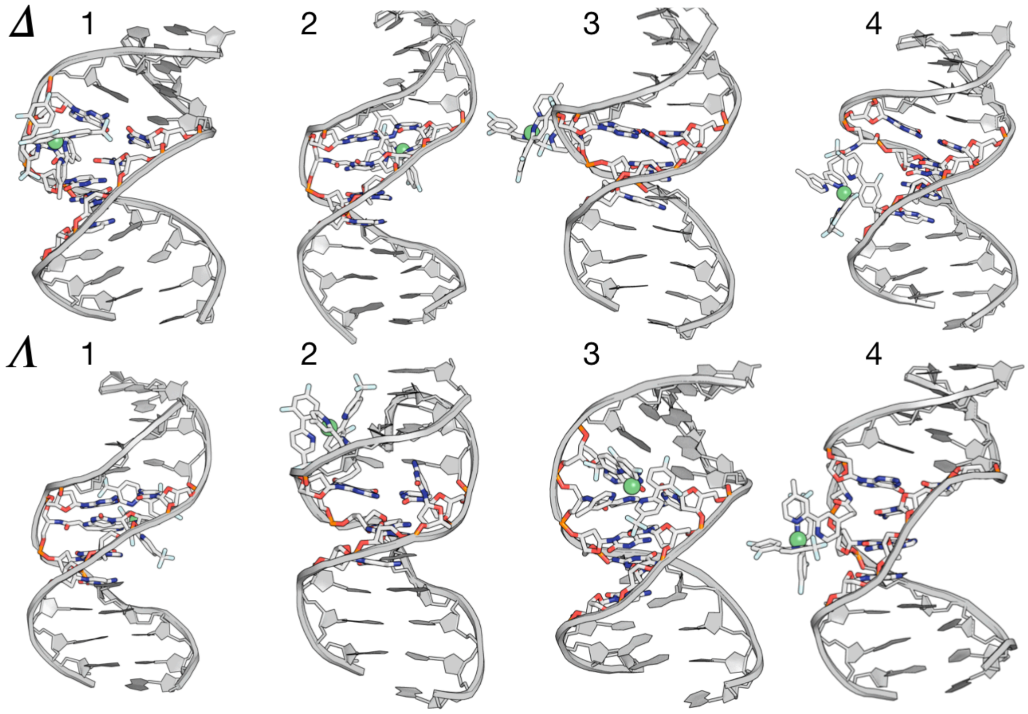

Figure S5. *Representative apo structures for ODN_1-S_L-[ΔIr]/ODN_{C4} (upper panels) and ODN_1-S_L-[ΛIr]/ODN_{C4} (lower panels). Corresponding percentages for the structures 1-4 are listed in Table S9.*

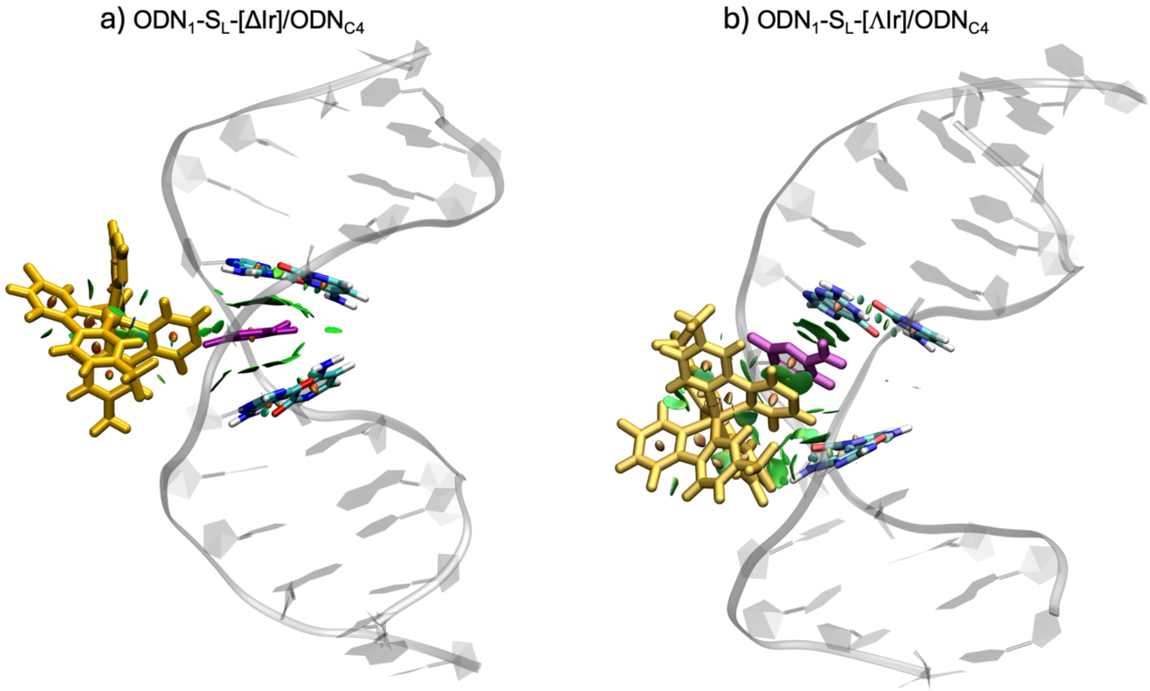

Figure S6. *Cartoon representation of the structures obtained from molecular dynamics simulations for ODN$_1$-S$_L$-[ΔIr]/ODN$_{C4}$ (a) and ODN$_1$-S$_L$-[ΛIr]/ODN$_{C4}$ (b) with NCI plot visualizing cooperative non-covalent interactions (in green) stabilizing the minor groove binding structures (density isosurface cut-off set up to 0.3). C18 in purple, iridium complex in yellow.*

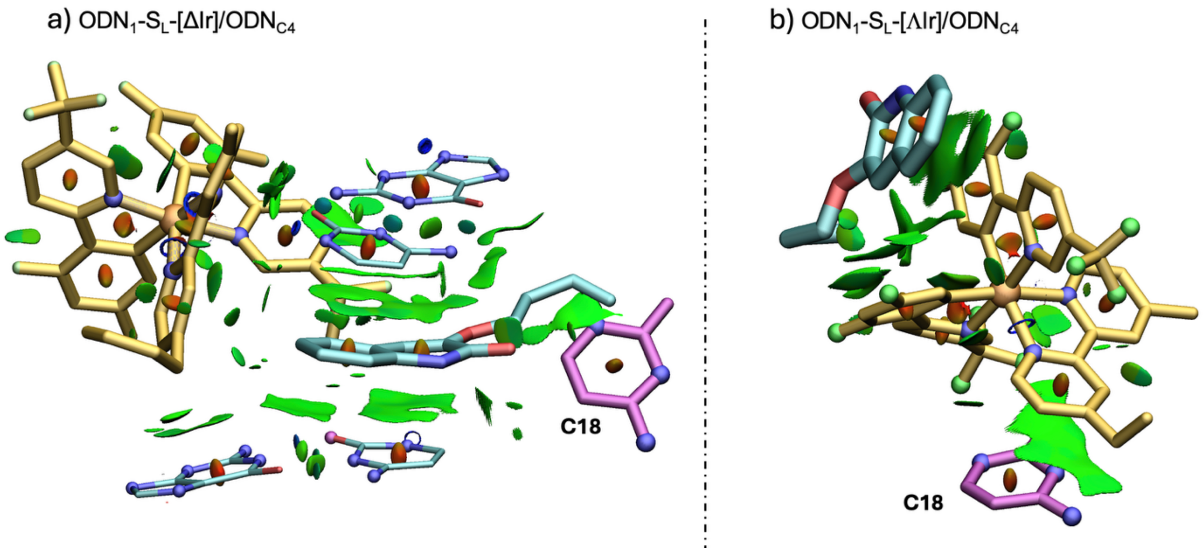

Figure S7. *a) NCI analysis for the substrate **1a** (boldfaced) interacting within the catalytic pocket formed after C18 (in purple) extrusion of ODN$_1$-S$_L$-[ΔIr]/ODN$_{C4}$. b) NCI analysis for the substrate **1a** (boldfaced) interacting within the iridium complex of ODN$_1$-S$_L$-[ΛIr]/ODN$_{C4}$. Density isosurfaces in green denote π-stacking interactions.*

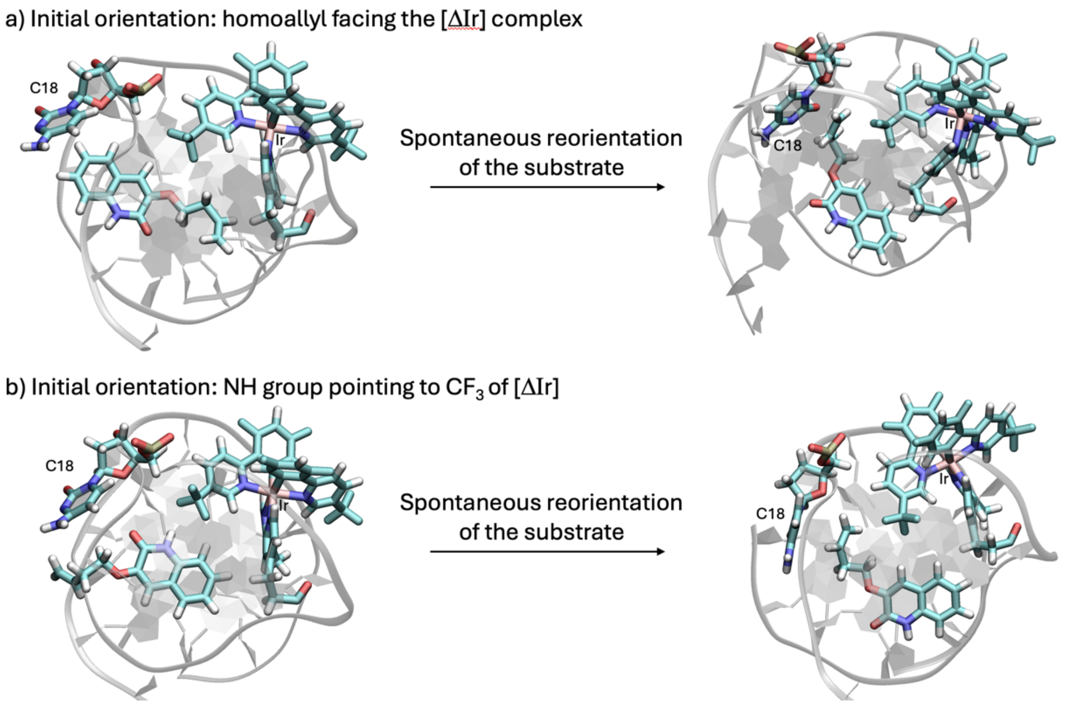

Figure S8. *Reorientations of the substrate **1a** along unbiaised MD simulations (200ns) that illustrate its preferential intercalation in the photoDNAzyme pocket to favor π-stacking interactions with proximal base pairs and between the exocyclic alkene with the extruded cytosine C18. Structures shown in (a) and (b) correspond to the first and final frames obtained after 200ns of MD simulations.*

Molecular dynamics situating the role of the 18th nucleobase

A similar computational protocol was followed to situate the role of the 18th nucleobase, which was either a guanine, an adenine or a thymine. Five replica of 200ns each was simulated at 300K in presence of potassium and chlorine ions corresponding to an ionic concentration of 0.15 M, and in water boxes whose dimensions are listed in Table S10. Cluster analyses were performed in order to situate the most representative structures for the photoDNAzyme shown in Figures S9-S11.

Substrate-photoDNAzyme interactions were situated along explicit solvent, all-atom dynamics with the same protocol as for the C18-containing photoDNAzyme (ODN_1-S_L-[ΔIr]/ODN_{C4}). In all these simulations, the substrate was found to interact mostly at the extremity of the terminal base pair or near the iridium complex as for the C18 containing ODN_1-S_L-[ΛIr]/ODN_{C4} duplex (Figure 5A of the main text). These additional simulations hence corroborate the special role played by the central cytosine C18 in the ODN_1-S_L-[ΔIr]/ODN_{C4} duplex.

***Table S10**: Dimensions and number of water molecules of the eight simulation boxes, for the apo structures (without substrate **1a**) and holo structures (in presence of the substrate **1a**).*

**Apo**	**ODN_1-S_L-[ΔIr]/ODN_{Cx}**		**ODN_1-S_L-[ΛIr]/ODN_{Cx}**	
	Dimensions (Å^3)	Number of water molecules	Dimensions (Å^3)	Number of water molecules
C18	74.0*63.1*80.0	9762	68.5*66.7*80.0	9321
A18	74.5*63.3*78.8	9762	68.8*65.9*79.4	9617
G18	72.8*62.9*78.5	9583	68.0*67.0*79.3	9577
T18	64.5*63.2*75.3	9762	69.5*68.1*81.1	9828
**Holo**	**ODN_1-S_L-[ΔIr]/ODN_{Cx}**		**ODN_1-S_L-[ΛIr]/ODN_{Cx}**	
	Dimensions (Å^3)	Number of water molecules	Dimensions (Å^3)	Number of water molecules
C18	89.1*67.8*81.3	12942	72.5*68.4*95.5	12030
A18	74.1*67.8*90.6	11968	74.1*63.9*90.6	11097
G18	69.2*60.0*84.5	11181	69.2*60.0*84.5	11093
T18	73.4*65.2*88.8	10904	69.2*60.0*79.5	10504

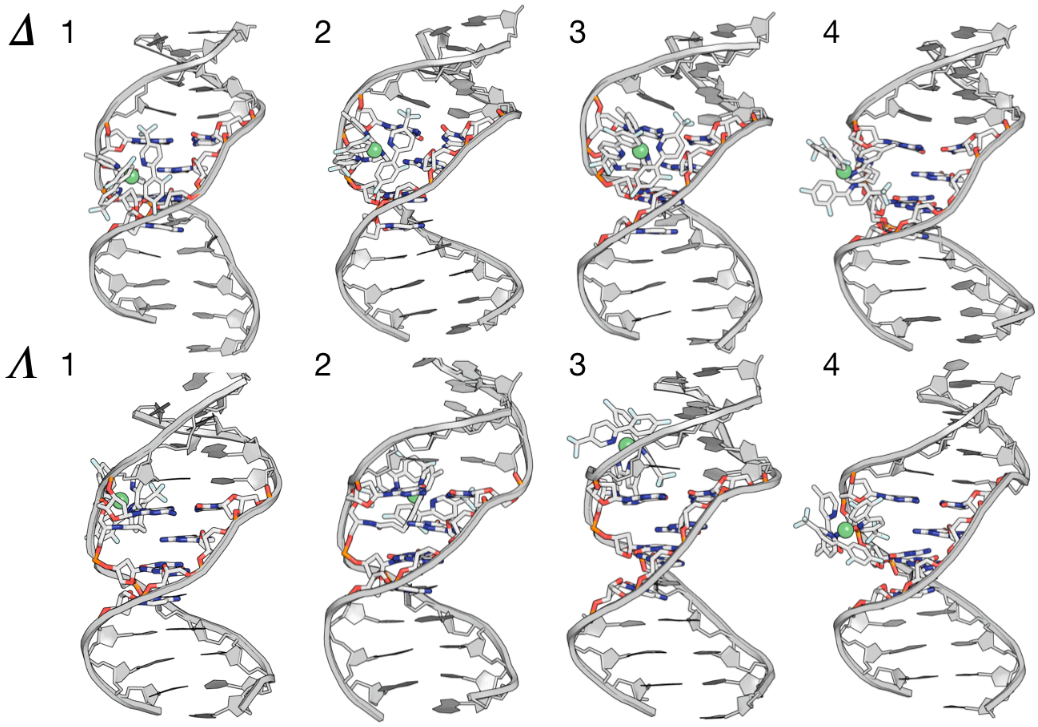

***Figure S9.** Representative apo structures for G18 ODN_1-S_L-[ΔIr]/ODN_{C3} (upper panels) and G18 ODN_1-S_L-[ΛIr]/ODN_{C3} (lower panels). Respective percentages for the structures 1-4 are: 46.3, 26.9, 12.1 and 6.5% for ODN_1-S_L-[ΔIr]/ODN_{C3} and 31.7, 14.8, 12.8 and 12.1 ODN_1-S_L-[ΛIr]/ODN_{C3}.*

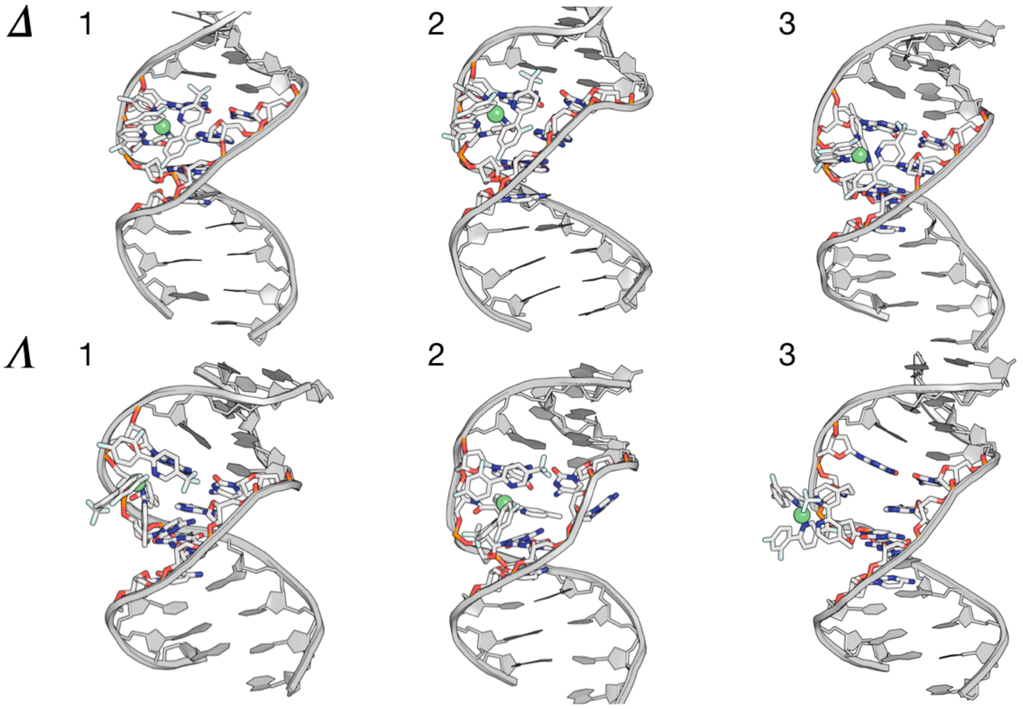

Figure S10. *Representative apo structures for A18 ODN$_1$-S$_L$-[ΔIr]/ODN$_{C2}$ (upper panels) and A18 ODN$_1$-S$_L$-[ΛIr]/ODN$_{C2}$ (lower panels). Respective percentages for the structures 1-4 are: 33.5, 27.9, 21.6% for ODN$_1$-S$_L$-[ΔIr]/ODN$_{C2}$ and 30.9, 28.5, 12.4% for ODN$_1$-S$_L$-[ΛIr]/ODN$_{C2}$.*

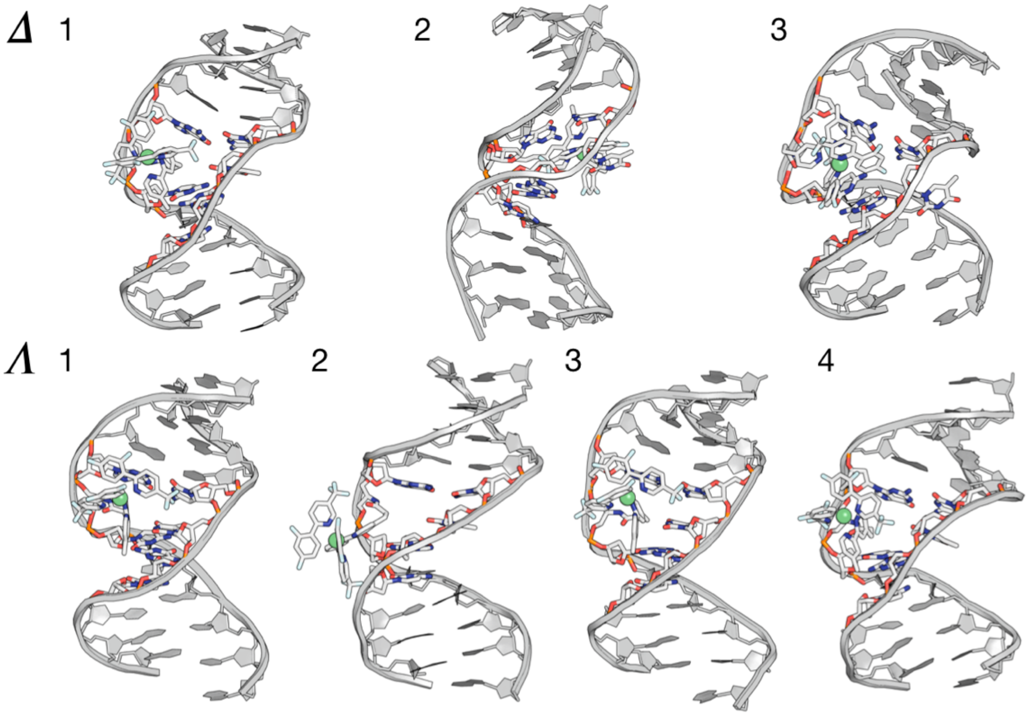

Figure S11. *Representative apo structures for T18 ODN$_1$-S$_L$-[ΔIr]/ODN$_{C1}$ (upper panels) and T18 ODN$_1$-S$_L$-[ΛIr]/ODN$_{C1}$ (lower panels). Respective percentages for the structures are: 20.8, 20.8, 20.4% for ODN$_1$-S$_L$-[ΔIr]/ODN$_{C1}$ and 37.7, 21.7, 16.2 and 6.3% ODN$_1$-S$_L$-[ΛIr]/ODN$_{C1}$.*

VIII. Photophysical Measurements

1. Excited state lifetime measurements

Luminescence lifetime spectra were obtained on Horiba DeltaFlex Modular Fluorescence Lifetime System (Time Correlated Single Photon Counting lifetime measurement) as described in the General informations.

Fitting of ODN$_1$-S$_L$-[ΔIr] / ODN$_{C4}$ duplex

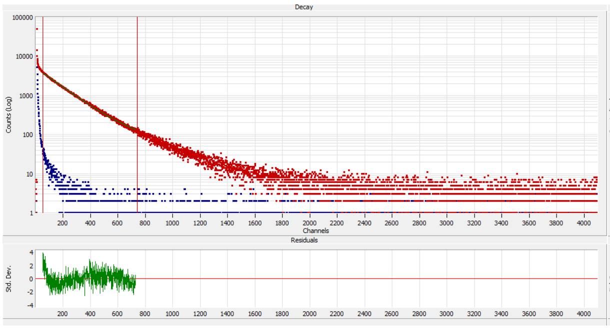

Fitting of ODN$_1$-S$_L$-[ΛIr] / ODN$_{C4}$ duplex

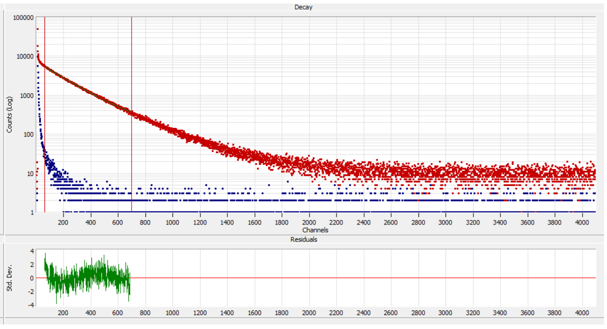

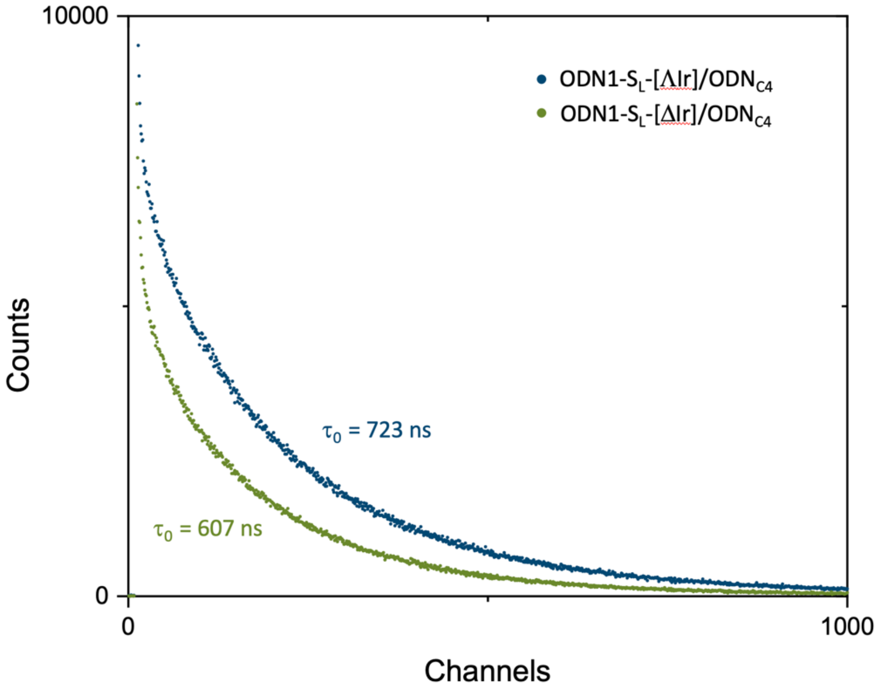

Figure S12 *Excited state lifetime measurement of ODN$_1$-S$_L$-[ΔIr] / ODN$_{C4}$ and ODN$_1$-S$_L$-[ΛIr] / ODN$_{C4}$ duplexes*

2. Stern-Volmer experiment

Luminescence spectra were obtained on a JASCO J-815 as described in the General information.

Procedure for excited-state quenching experiment: A 10 µM solution of ODN$_1$-S$_L$-[ΔIr] / ODN$_{C4}$ or ODN$_1$-S$_L$-[ΛIr] / ODN$_{C4}$ duplex in H_2O was prepared, 100 µL of this solution added into the cuvette and the maximum intensity of luminescence without quencher (I_0) was recorded (at 470 nm). At this sample, 0.5 µL of 30 mM solution of quencher (**2a**) in THF was added and the maximum intensity of luminescence with quencher (I_1) was measured. This step was repeated three times with the addition of 0.5 µL of quencher each time to obtain I_2, I_3 and I_4.

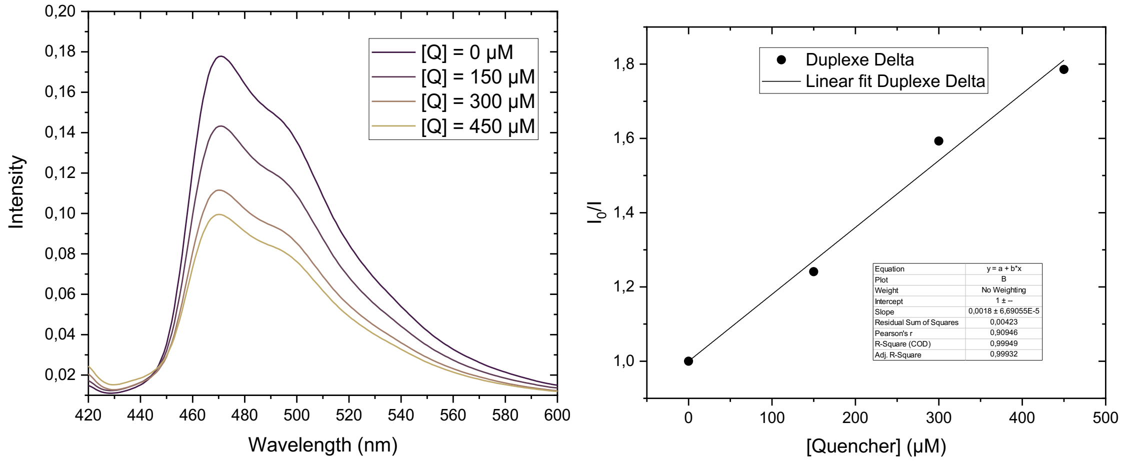

[Quencher]	0 µM	150 µM	300 µM	450 µM
**Intensity**	I_0 = 0,83825	I_1 = 0,690484	I_2 = 0,58759	I_3 = 0,51847

Figure S13 *Stern-Volmer experiment of ODN$_1$-S$_L$-[ΔIr] / ODN$_{C4}$ duplex*

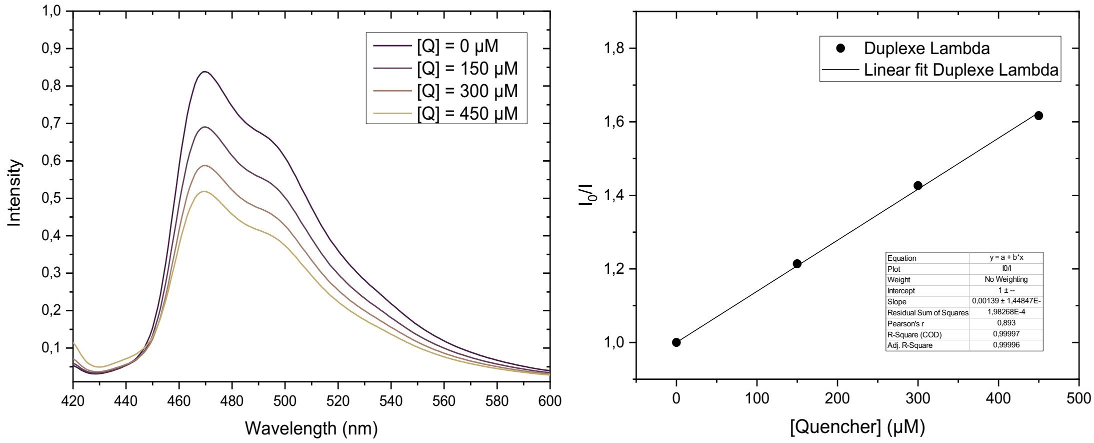

[Quencher]	0 µM	150 µM	300 µM	450 µM
**Intensity**	I_0 = 0.83825	I_1 = 0.69048	I_2 = 0.58759	I_3 = 0.51819

Figure S14. *Stern-Volmer experiment of ODN$_1$-S$_L$-[ΛIr] / ODN$_{C4}$ duplex*

IX. Cyclic voltammetry data

Voltammetric measurements were carried out with a potentiostat Autolab PGSTAT100 controlled by GPES 4.09 software. Experiments were performed at room temperature in a homemade airtight three- electrode cell consisting of a Pt working electrode (d = 0.5 mm), a platinum wire (S = 1 cm^2) as counter electrode, and a saturated calomel electrode (SCE) separated from the solution by a bridge compartment as a reference. Before each measurement, the working electrode was cleaned with a polishing machine (Presi P230, P4000). The measurements were carried out in dry CH_2Cl_2 under argon atmosphere using 0.1 M $[nBu_4N](PF_6)$ as supporting electrolyte and 10^{-3} M sample concentration.

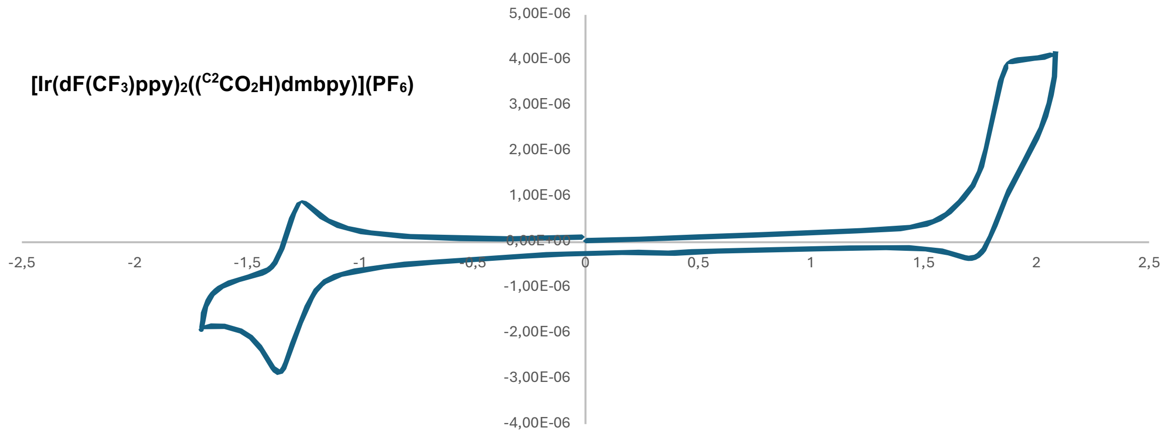

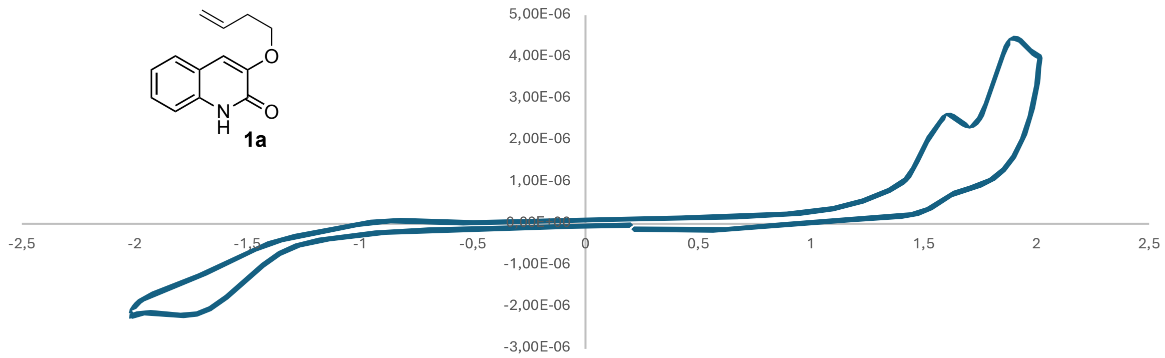

	E_{red}[a]	E_{ox}[a]	E_{00} (eV)[b]	$E(Ir^{III*/II})$ [a]	$E(Ir^{III*/IV})$ [a]
**[Ir]**	-1.32	1.81	2.64	1.32	-0.83
**1a**	-1.71	1.57			

[a]Redox potentials (V) are given *vs* saturated calomel electrode (SCE).
[b]E_{00} was estimated from the λ_{max} value on emission spectrum in CH_2Cl_2 at room temperature.

Figure S15. *Cyclic voltammograms of substrate* ***1a*** *and complex [Ir(dF(CF₃)ppy)₂((C2CO₂H)dmbpy)](PF₆)*

X. Complementay mechanistic tests

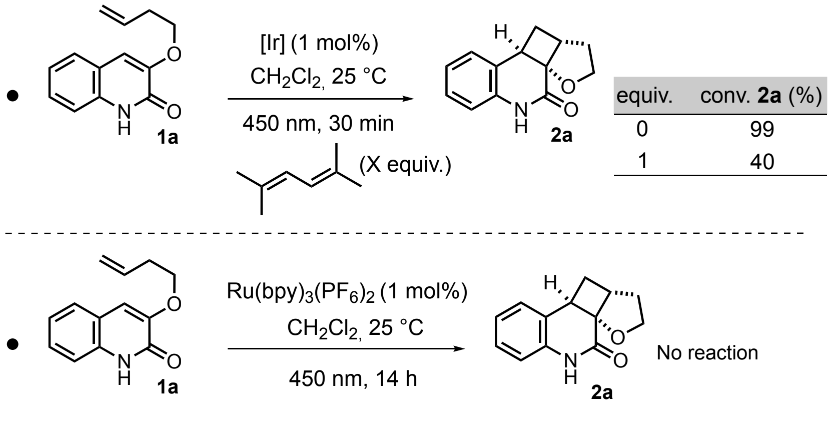

Figure S16. *Inefficiency of the transformation in the presence of 2,5-dimethylhexa-2,4-diene as triplet quencher (above) or in by replacing the iridium photocatalyst by [Ru(bpy)$_3$)(PF$_6$)$_2$ (below).*

XI. Scale-up and determination of absolute stereochemistry

The reaction was performed on 5 µmol of substrate (x 50 scale) following the **General procedure F** using ODN$_1$-S$_L$-[Ir] / ODN$_{C4}$ duplex as photoDNAzyme. A liquid-liquid extraction was done with EtOAc to collect the crude to perform NMR. Crude data showed that the reaction yield was 83 %. The product was purified by preparative TLC because of the very low crude mass (1 mg). Chiral HPLC showed an enantiomeric excess of 81 %, in accordance with the results obtained using ODN$_1$-S$_L$-[Ir] / ODN$_{C4}$ duplex (see XI-Chiral HPLC spectra).

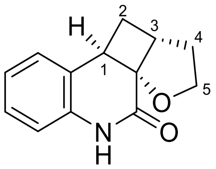

$[\alpha]_D^{20}$ = + 86 (c0.062, CH_2Cl_2)

α_D was performed on the crude due to the very low crude mass. By adjusting the concentration in accordance with NMR yield (0.75 mg/mL, 83 % yield => 0.62 mg/mL), an α_D value in good agreement with the (+)-enantiomer in the literature was found.[13] Determination of the stereochemistry of all other substrates was extended from this analysis.

rt (min)	area	%
11,090	489,632	90,55
16,973	51,085	9,45

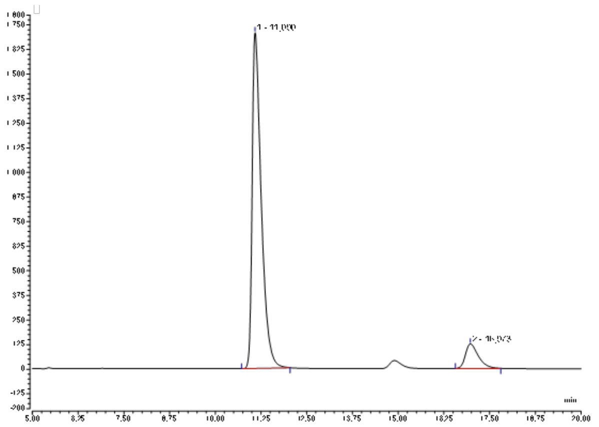

^{1}H NMR (400 MHz, $CDCl_3$), ^{13}C NMR (101 MHz, $CDCl_3$) of **2a** after purification

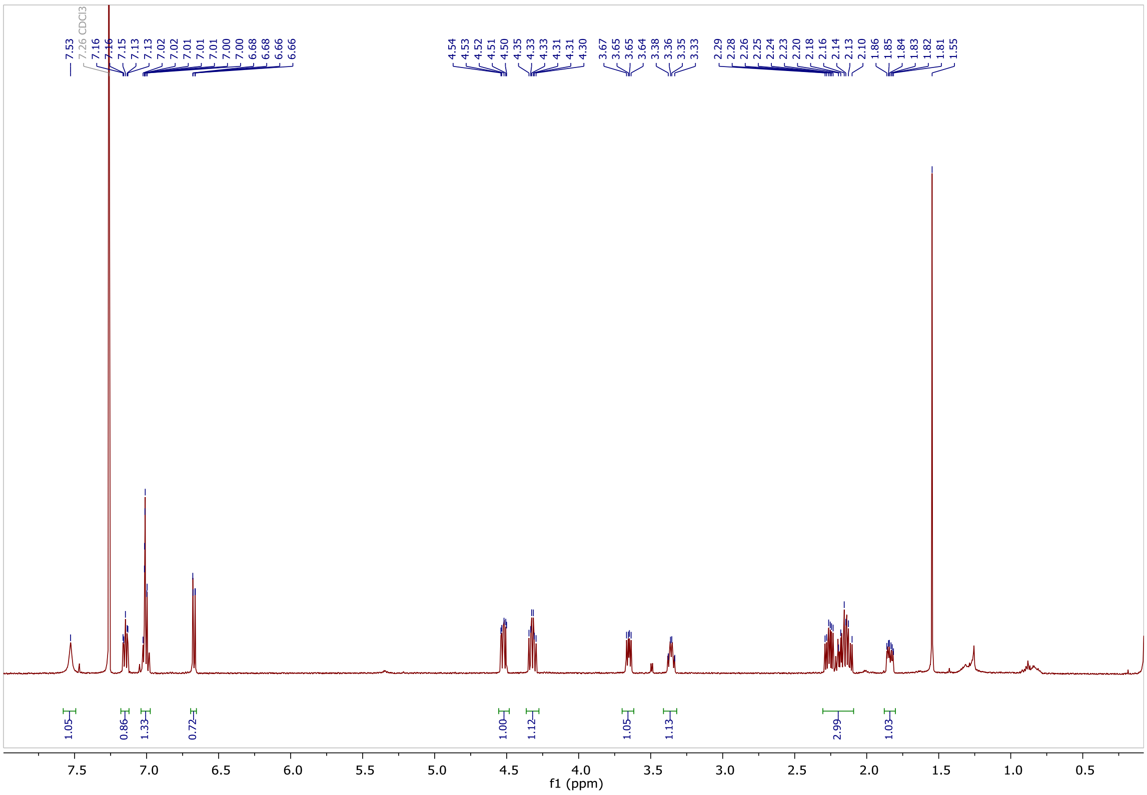

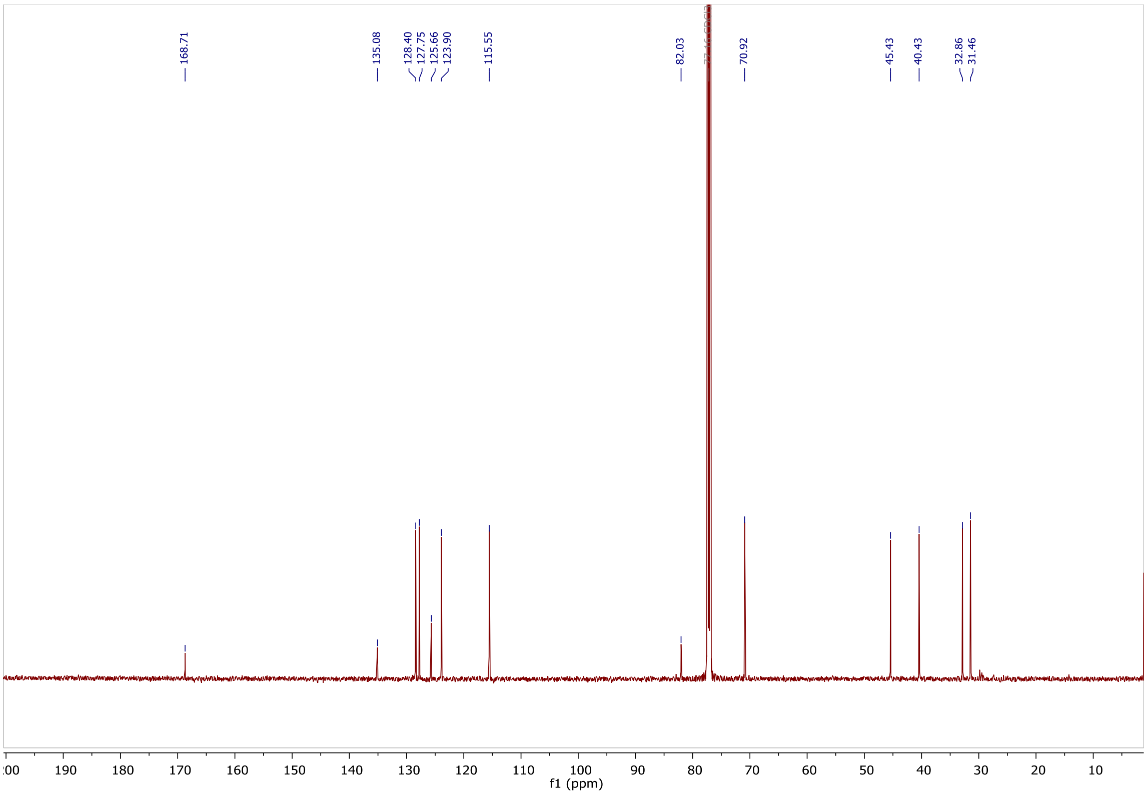

XII. NMR data

1. NMR of bipyridine ligands

^{1}H NMR (400 MHz, $CDCl_3$) of **benzyl 3-(4'-methyl-[2,2'-bipyridin]-4-yl)propanoate**

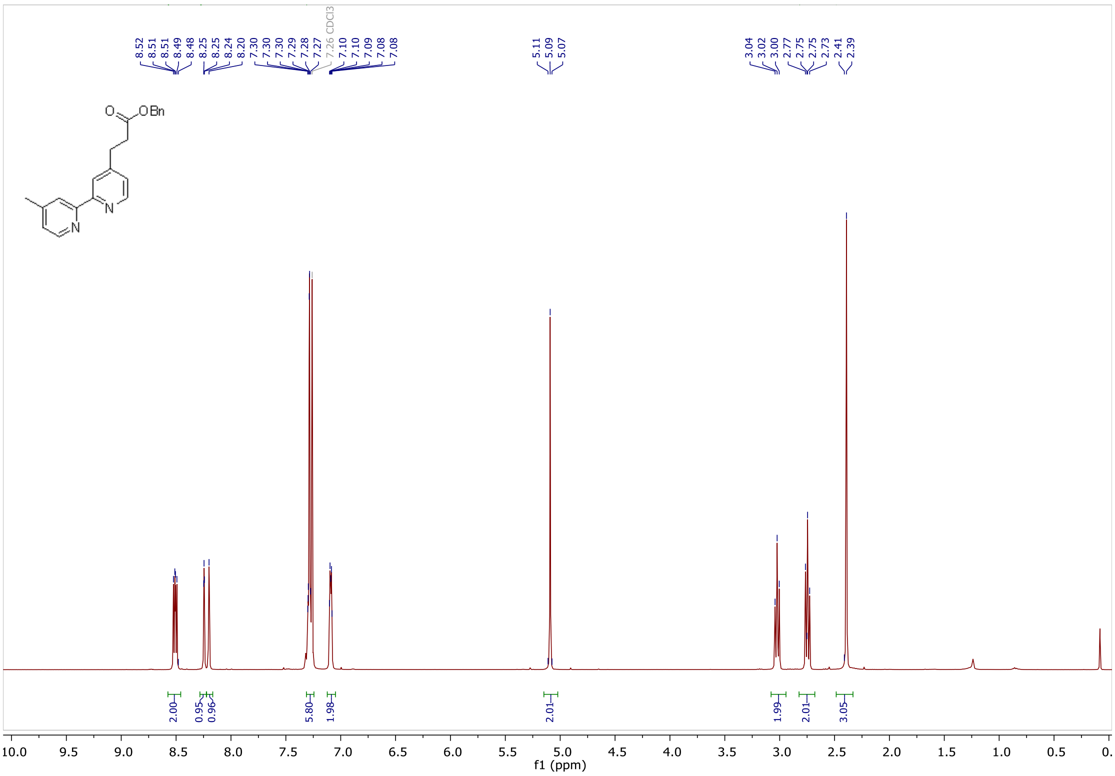

^{13}C NMR (101 MHz, $CDCl_3$) of **benzyl 3-(4'-methyl-[2,2'-bipyridin]-4-yl)propanoate**

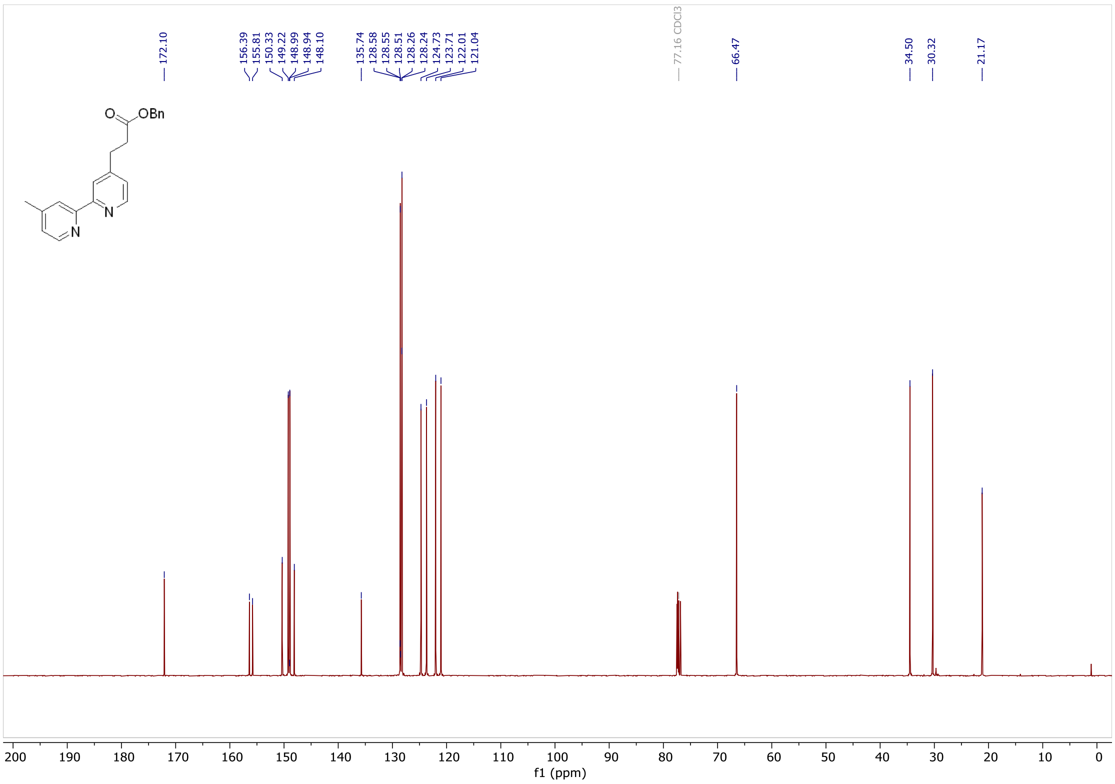

^{1}H NMR (400 MHz, $CDCl_3$) of **benzyl 4-(4'-methyl-[2,2'-bipyridin]-4-yl)butanoate**

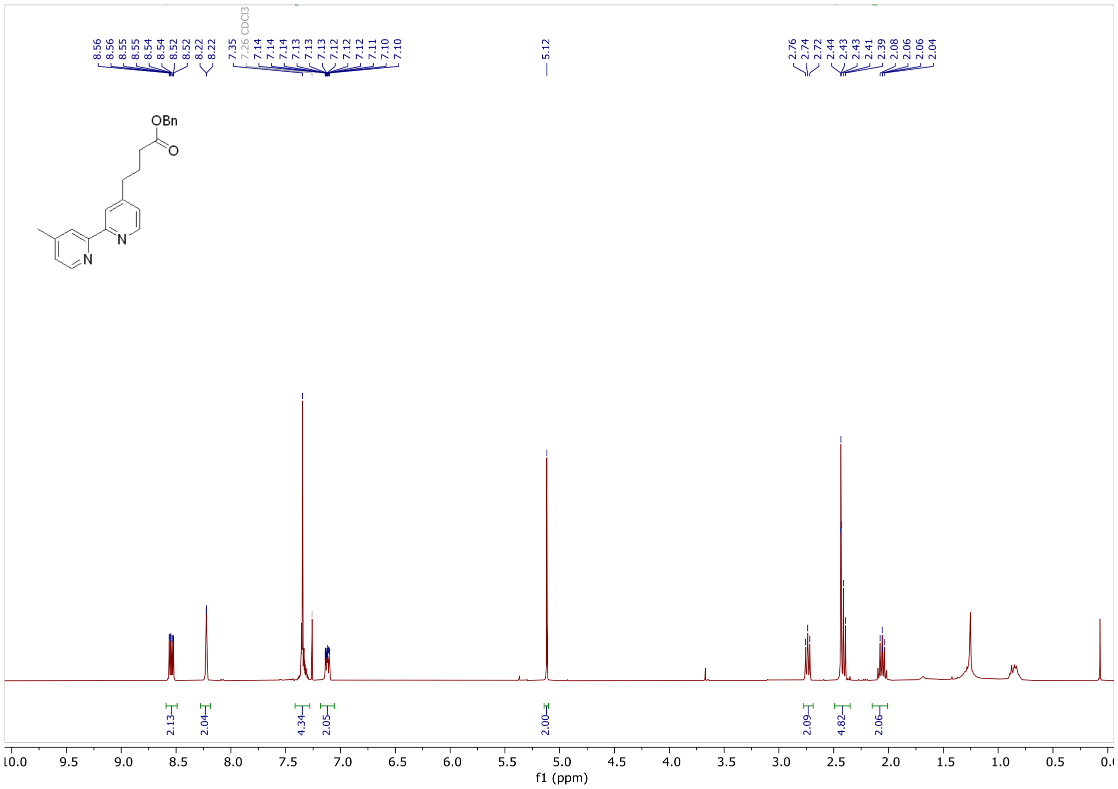

^{13}C NMR (101 MHz, $CDCl_3$) of **benzyl 4-(4'-methyl-[2,2'-bipyridin]-4-yl)butanoate**

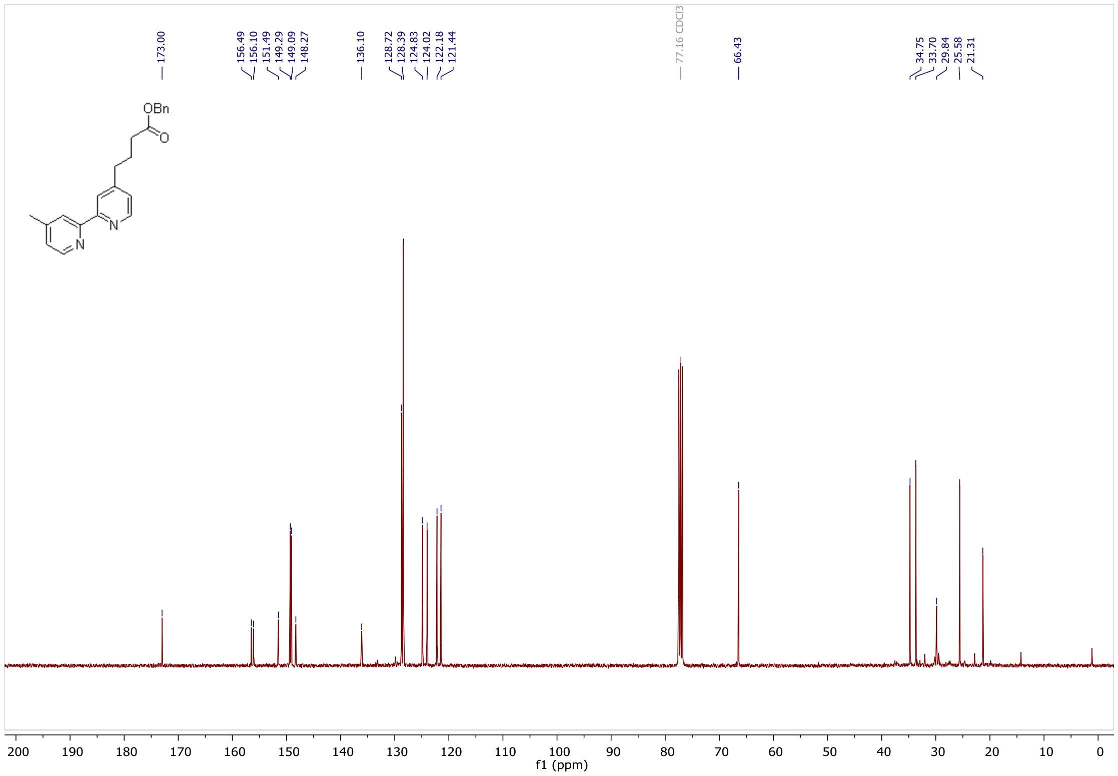

2. NMR of Iridium complexes

^{1}H NMR (400 MHz, Acetone-d_6) of **[Ir(dF(CF$_3$)ppy)$_2$((^{C0}CO$_2$H)dmbpy)](PF$_6$)**

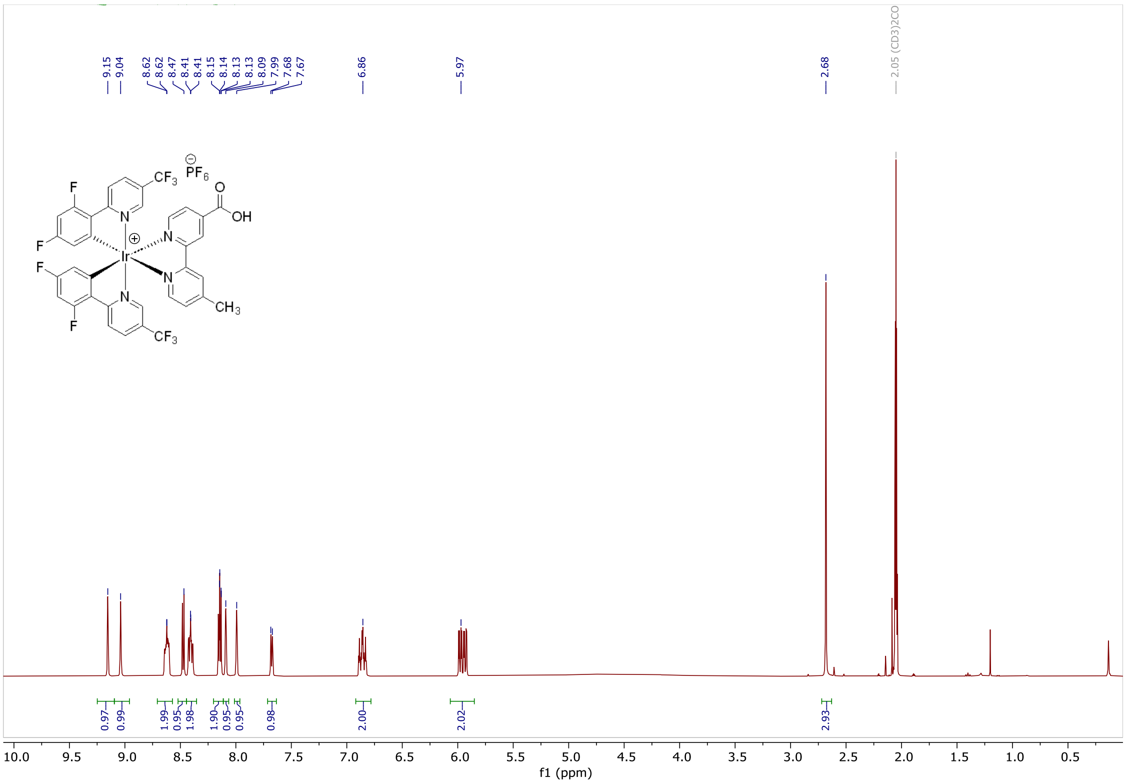

^{13}C NMR (101 MHz, Acetone-d_6) of **[Ir(dF(CF$_3$)ppy)$_2$((^{C0}CO$_2$H)dmbpy)](PF$_6$)**

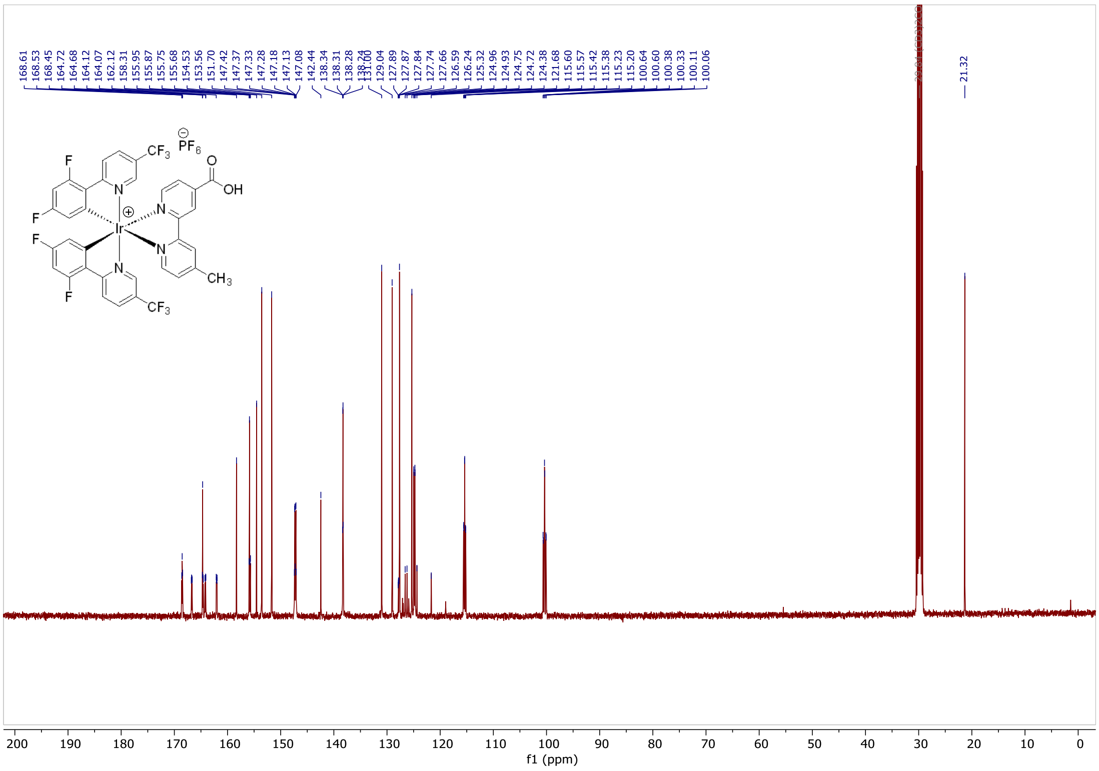

^{19}F (377 MHz, Acetone-*d6*) NMR of **[Ir(dF(CF3)ppy)2((C0CO2H)dmbpy)](PF6)**

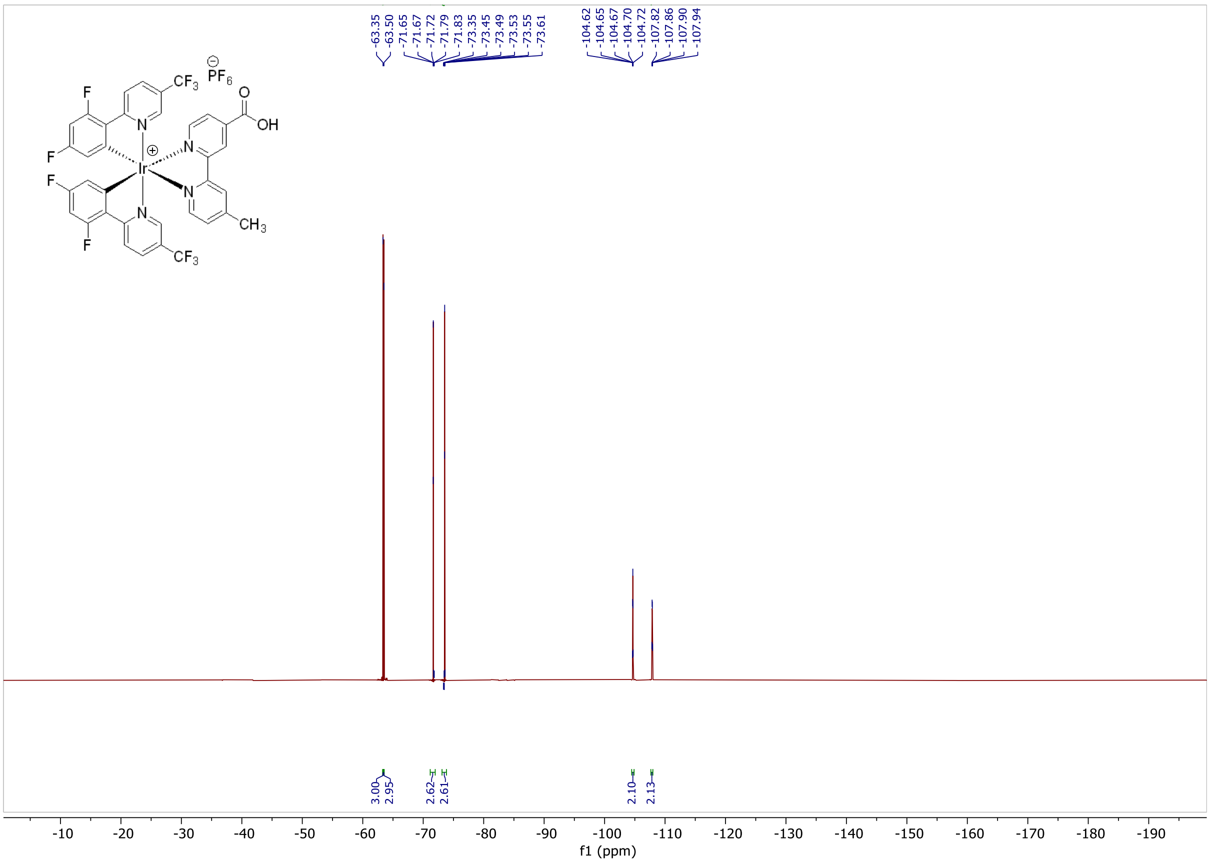

^{1}H NMR (400 MHz, Acetone-*d6*) of **[Ir(dF(CF3)ppy)2((C2CO2H)dmbpy)](PF6)**

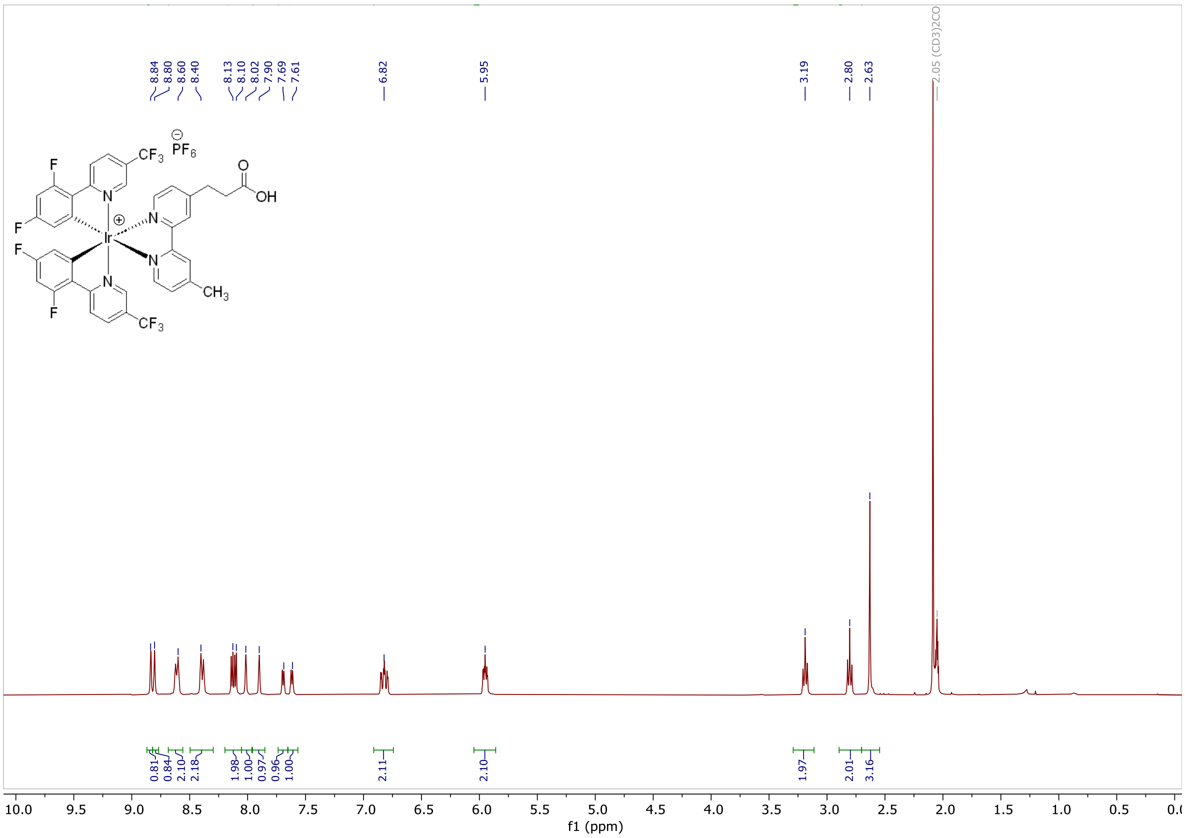

^{13}C NMR (101 MHz, Acetone-d_6) of **[Ir(dF(CF$_3$)ppy)$_2$((^{C2}CO$_2$H)dmbpy)](PF$_6$)**

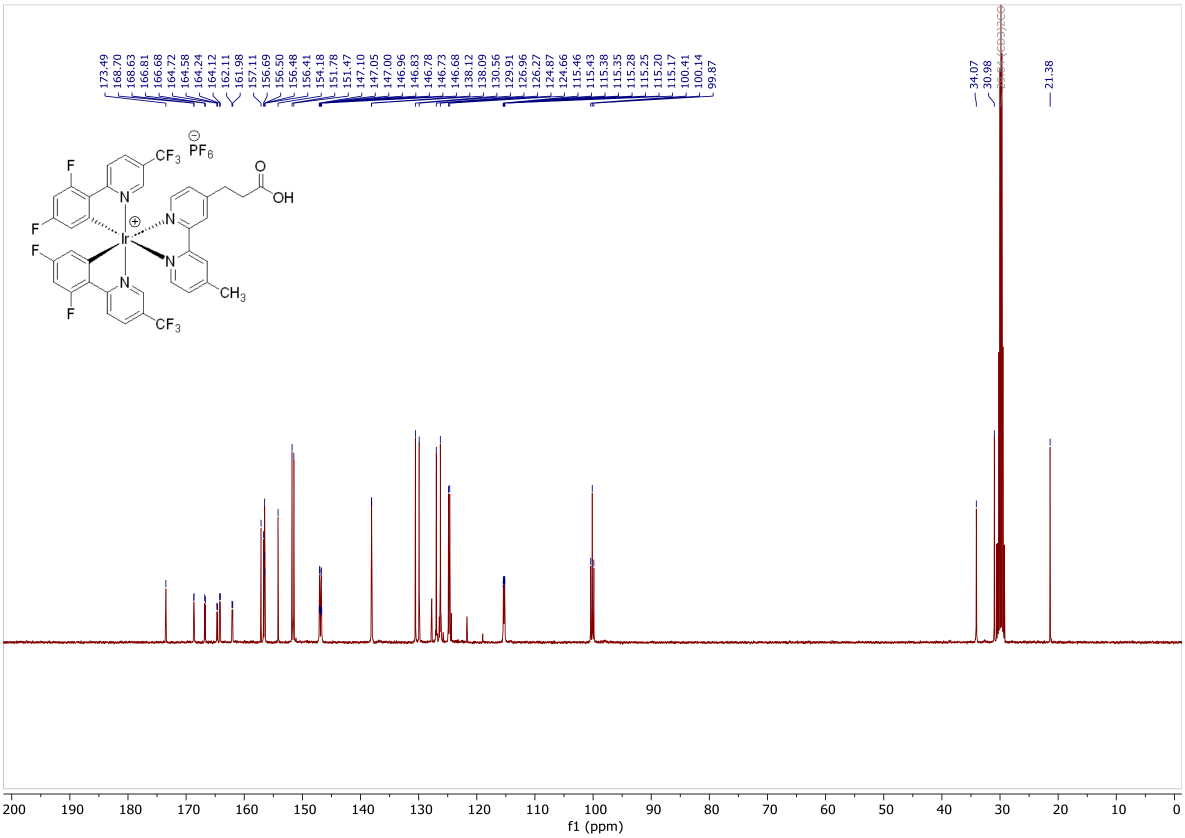

^{19}F NMR (377 MHz, Acetone-d_6) of **[Ir(dF(CF$_3$)ppy)$_2$((^{C2}CO$_2$H)dmbpy)](PF$_6$)**

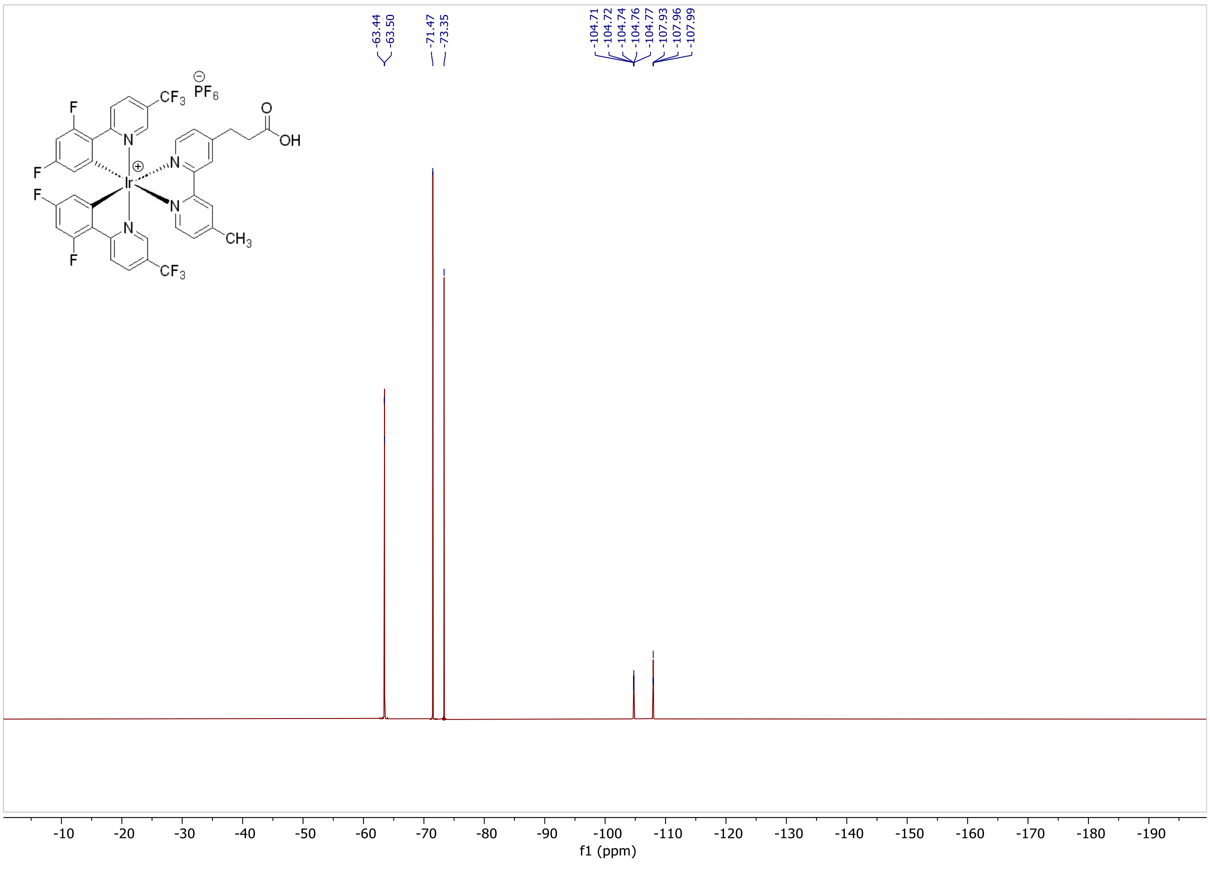

^{1}H NMR (400 MHz, Acetone-d_6) of **[Ir(dF(CF$_3$)ppy)$_2$((^{C3}CO$_2$Bn)dmbpy)](PF$_6$)**

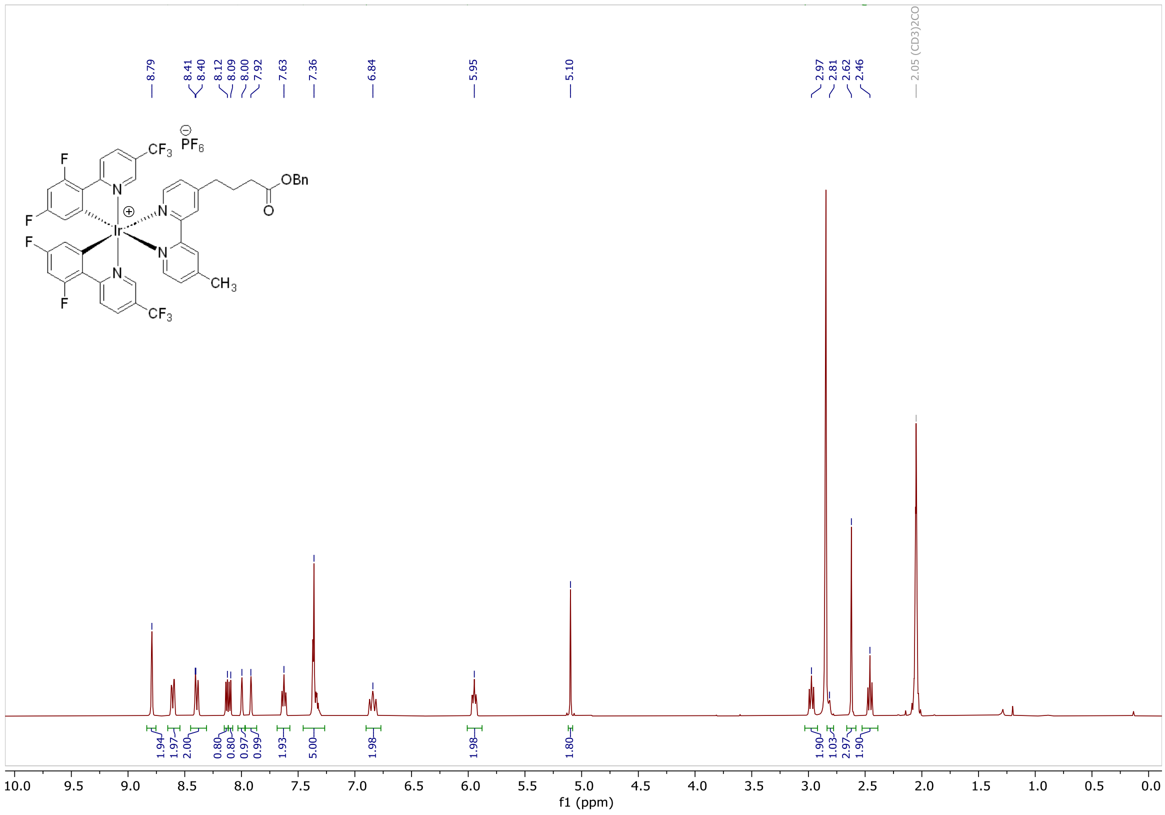

^{13}C NMR (101 MHz, Acetone-d_6) of **[Ir(dF(CF$_3$)ppy)$_2$((^{C3}CO$_2$Bn)dmbpy)](PF$_6$)**

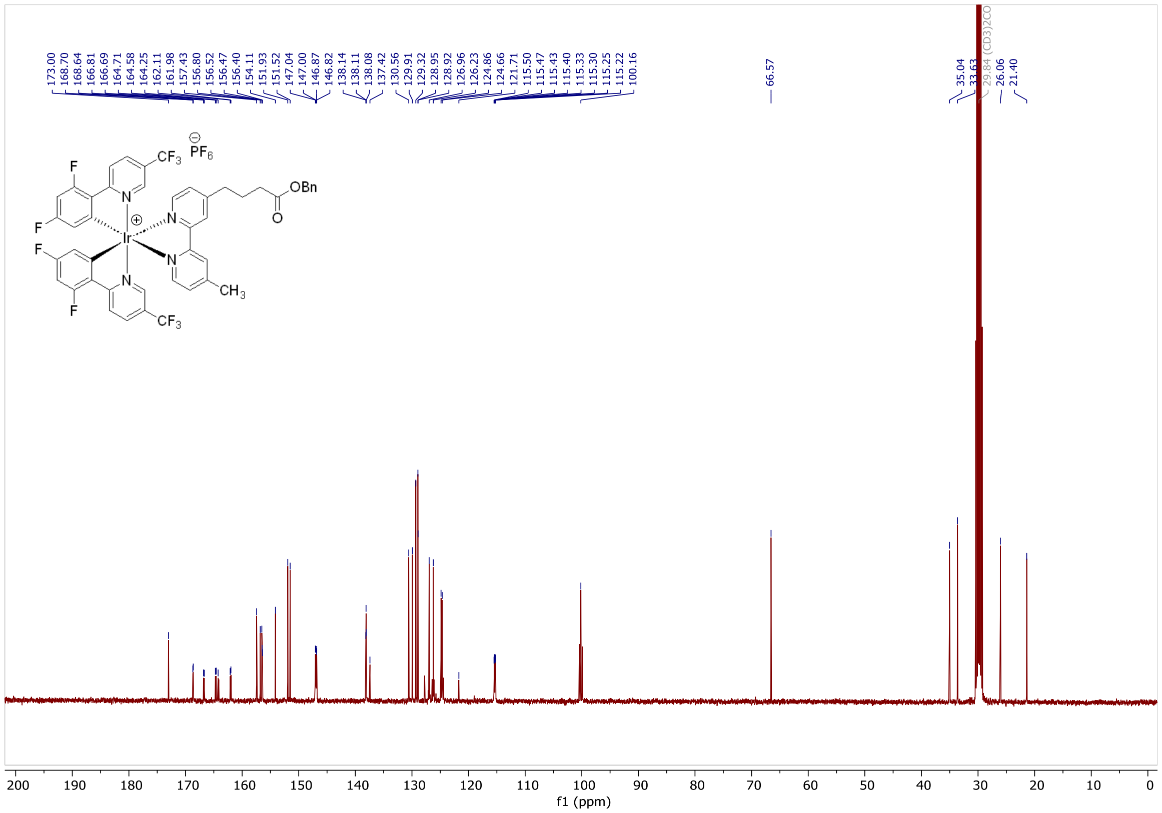

^{19}F (377 MHz, Acetone-d_6) NMR of **[Ir(dF(CF$_3$)ppy)$_2$((^{C3}CO$_2$Bn)dmbpy)](PF$_6$)**

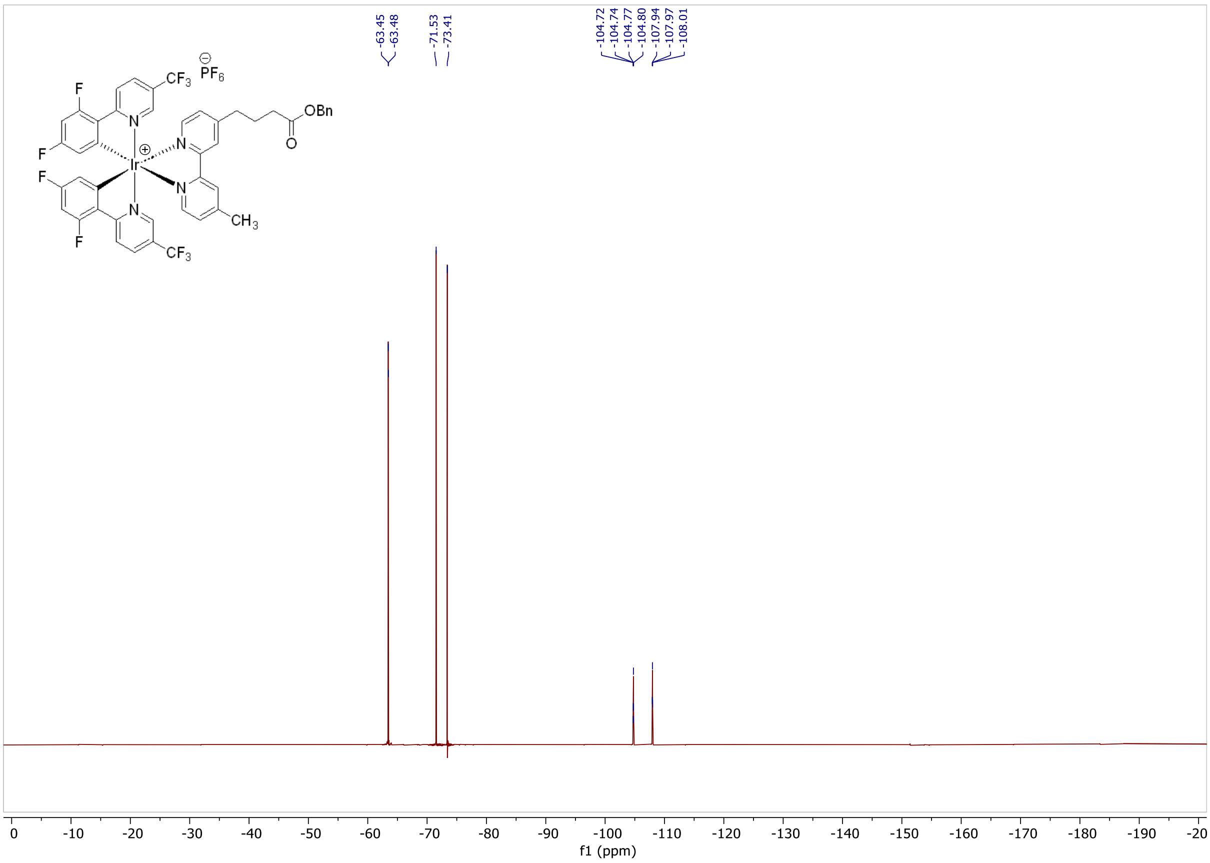

^{1}H NMR (400 MHz, Acetone-d_6) of **[Ir(dF(CF$_3$)ppy)$_2$((^{C3}CO$_2$H)dmbpy)](PF$_6$)**

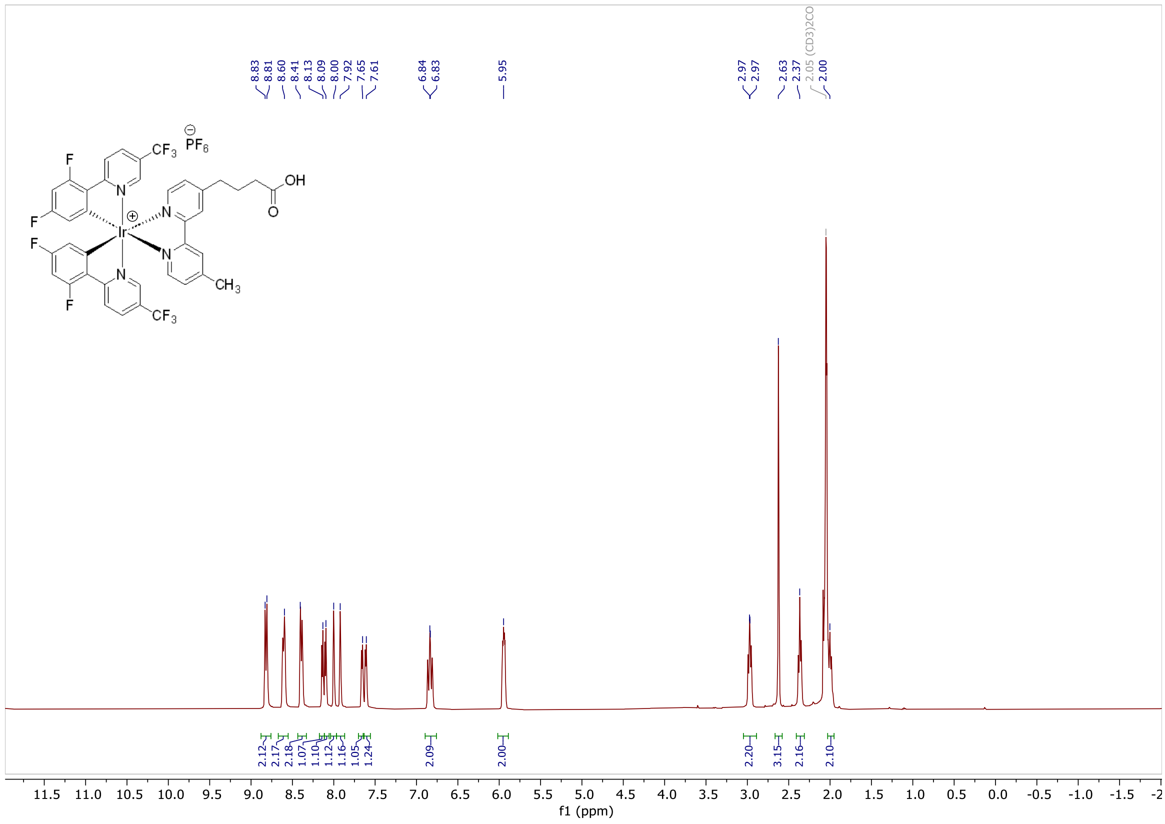

^{13}C NMR (101 MHz, Acetone-d_6) of **[Ir(dF(CF$_3$)ppy)$_2$((^{C3}CO$_2$H)dmbpy)](PF$_6$)**

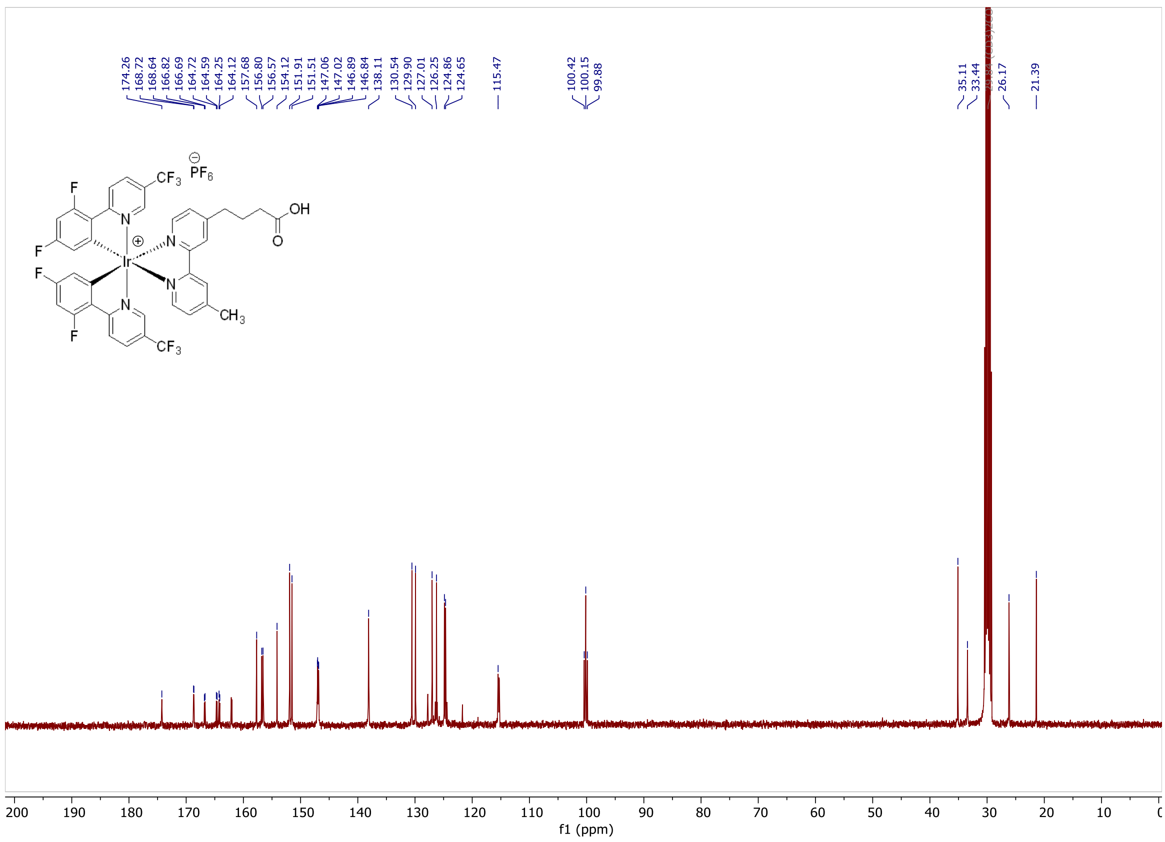

^{19}F NMR (377 MHz, Acetone-d_6) of **[Ir(dF(CF$_3$)ppy)$_2$((^{C3}CO$_2$H)dmbpy)](PF$_6$)**

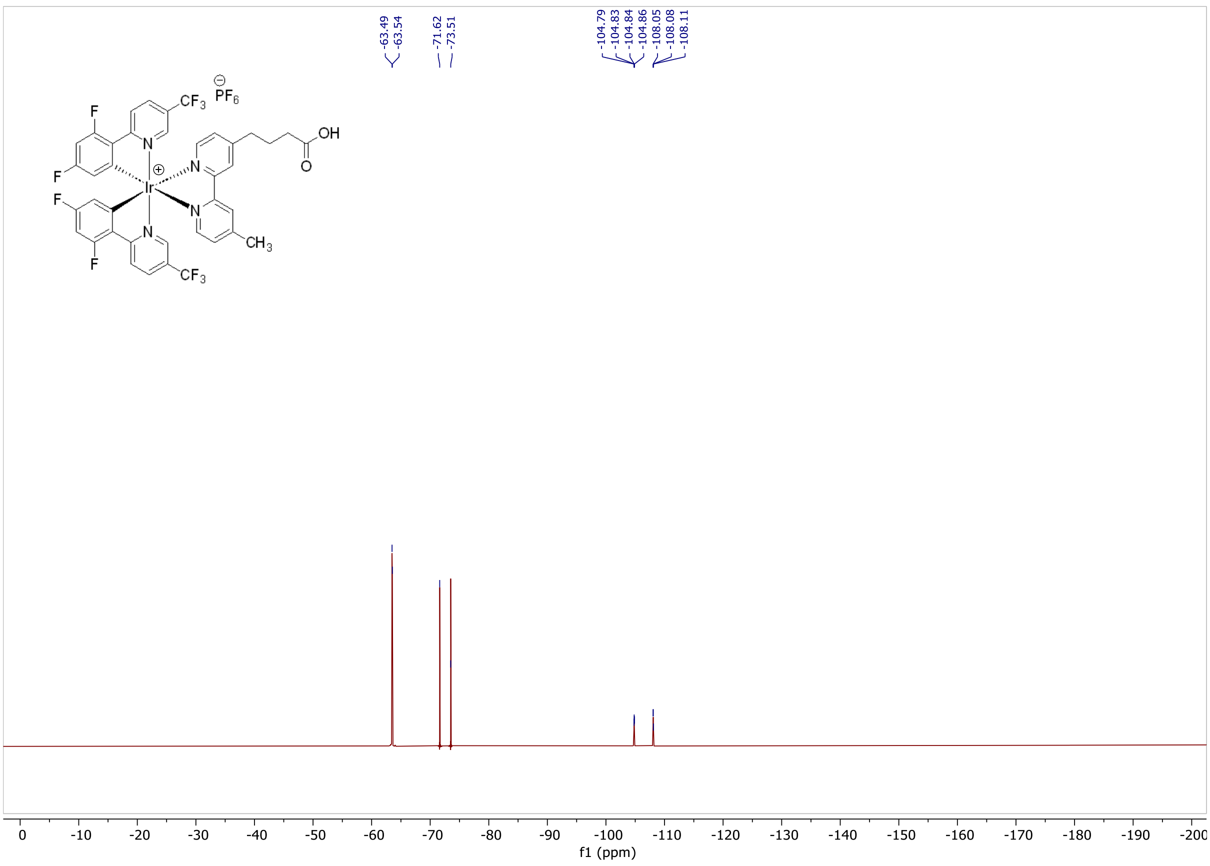

^{1}H NMR (300 MHz, $CDCl_3$) of **Λ[Ir(dF(CF$_3$)ppy)$_2$(*S*-oxazoline)]**

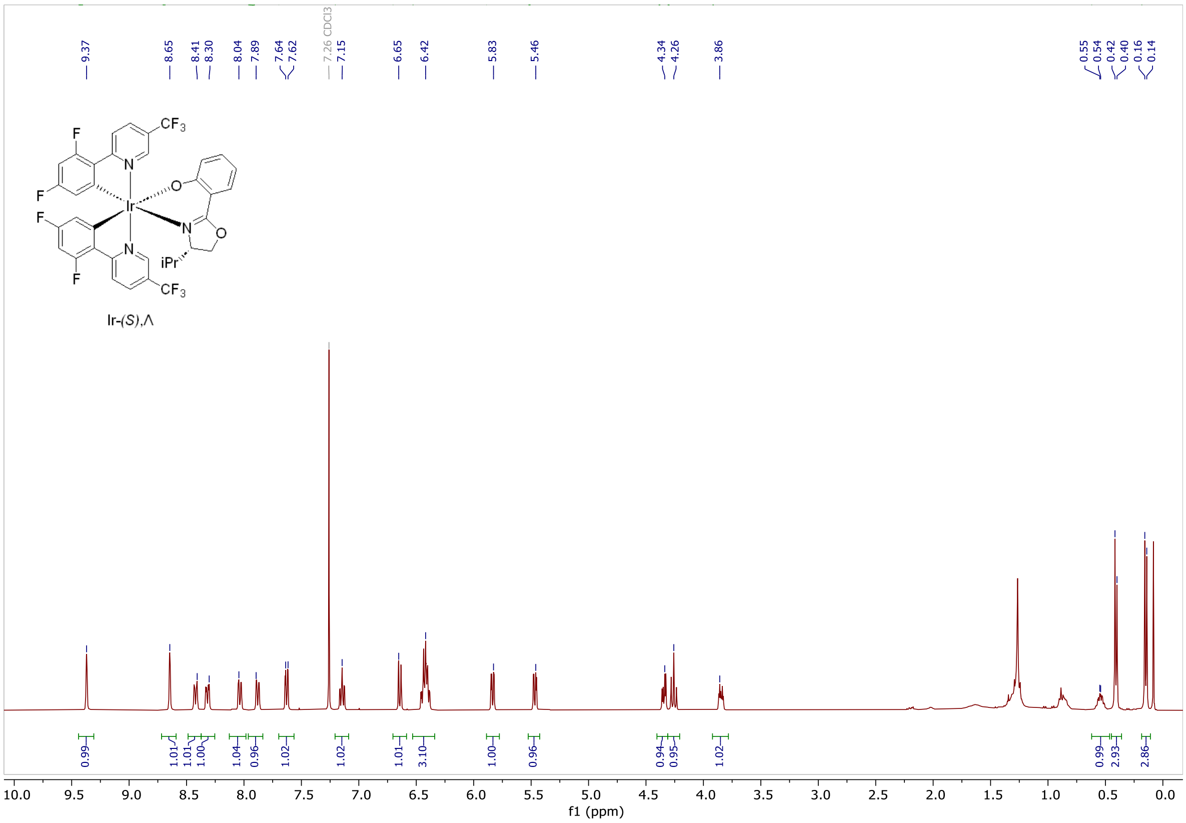

^{13}C NMR (101 MHz, $CDCl_3$) of **Λ[Ir(dF(CF$_3$)ppy)$_2$(*S*-oxazoline)]**

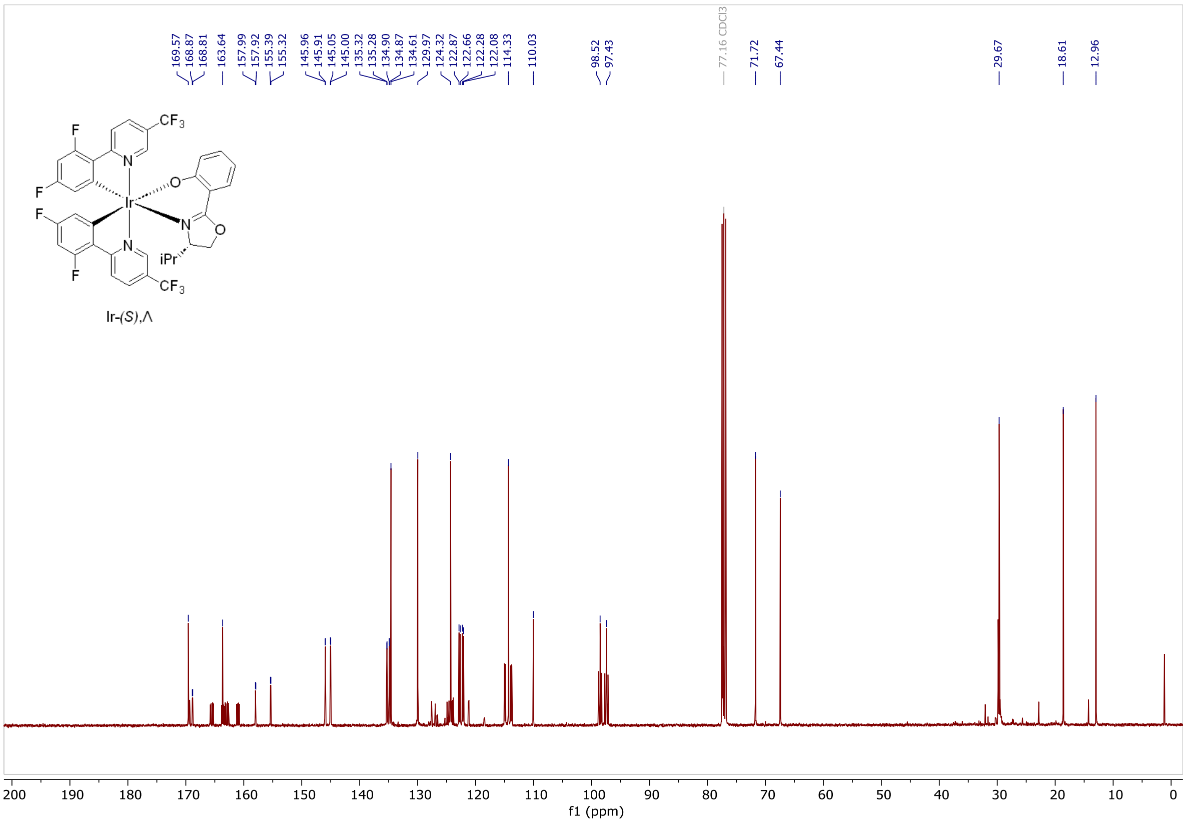

^{19}F NMR (282 MHz, $CDCl_3$) of **Λ[Ir(dF(CF_3)ppy)$_2$(*S*-oxazoline)]**

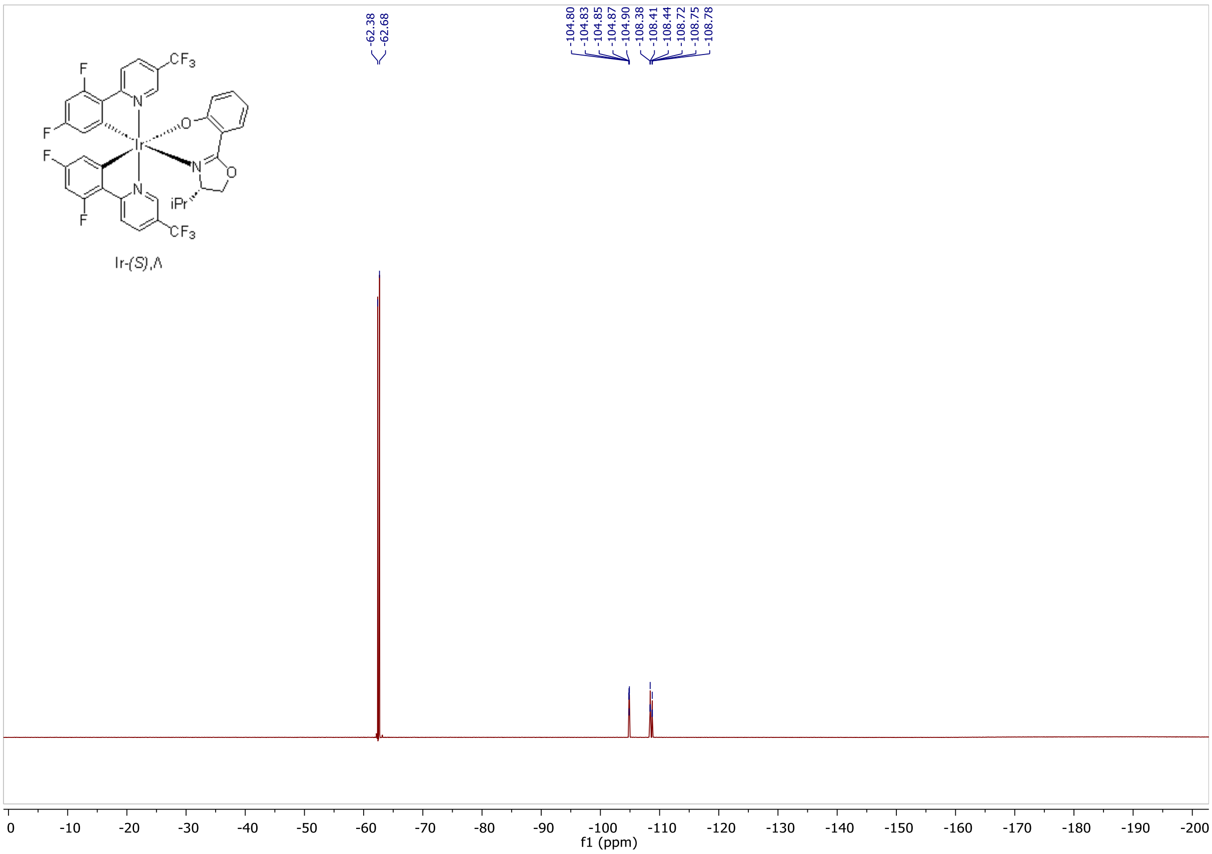

^{1}H NMR (400 MHz, $CDCl_3$) of **Δ[Ir(dF(CF_3)ppy)$_2$(*S*-oxazoline)]**

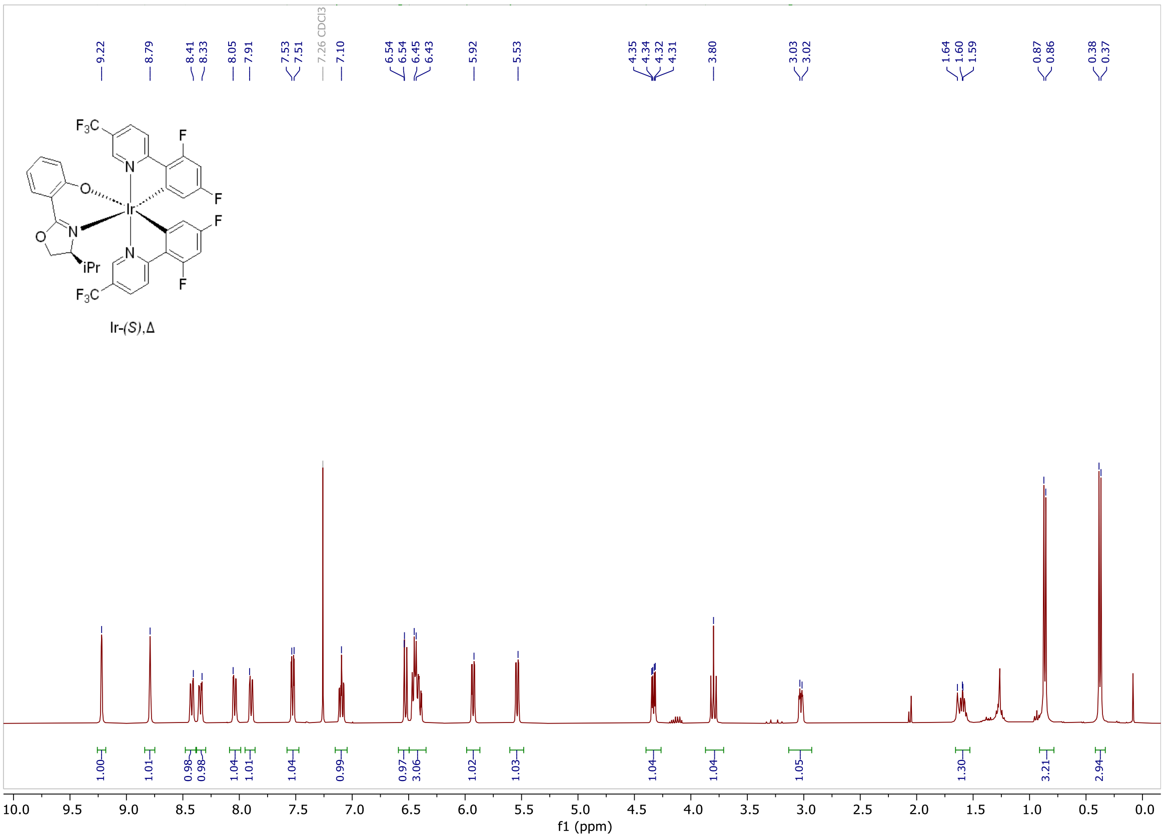

^{13}C NMR (101 MHz, CDCl$_3$) of **Δ[Ir(dF(CF$_3$)ppy)$_2$(*S*-oxazoline)]**

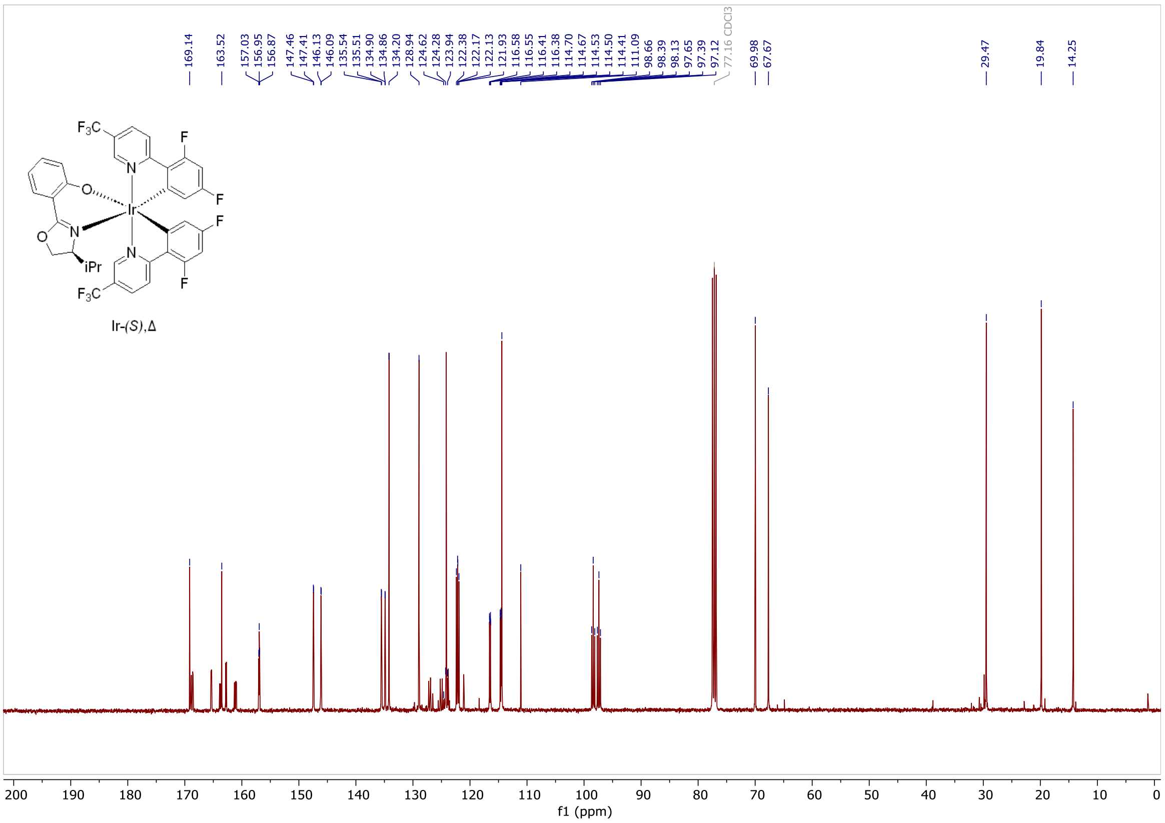

^{19}F NMR (377 MHz, CDCl$_3$) of **Δ[Ir(dF(CF$_3$)ppy)$_2$(*S*-oxazoline)]**

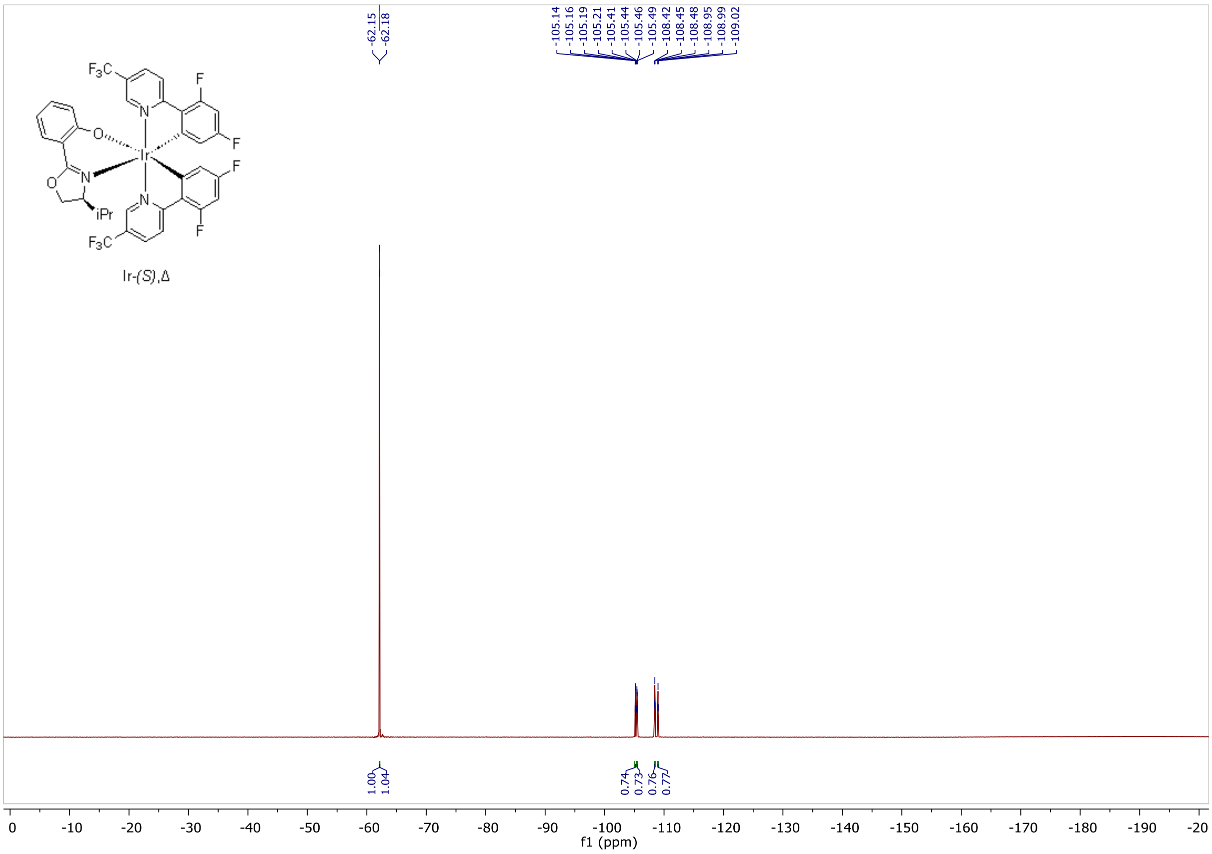

^{1}H NMR (400 MHz, Acetone-d_6) of **Λ[Ir(dF(CF$_3$)ppy)$_2$((^{C2}CO$_2$Bn)dmbpy)](PF$_6$)**

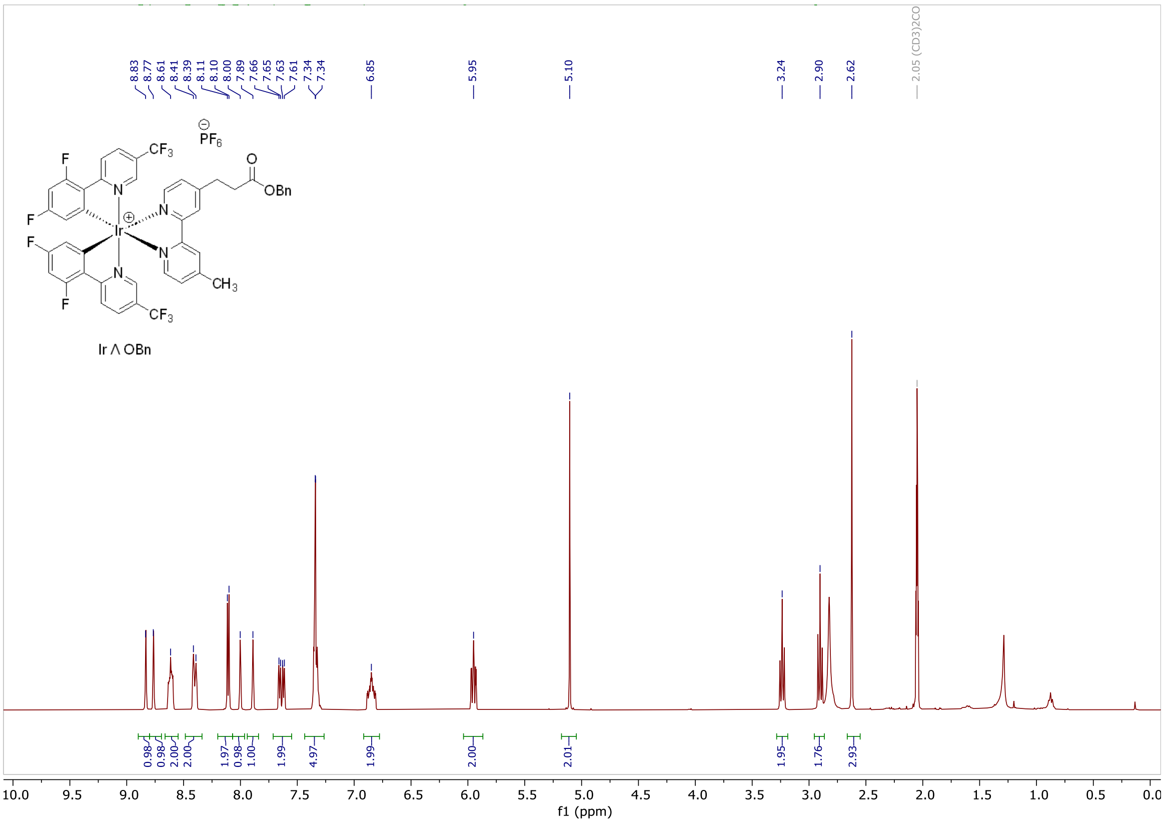

^{13}C NMR (101 MHz, Acetone-d_6) of **Λ[Ir(dF(CF$_3$)ppy)$_2$((^{C2}CO$_2$Bn)dmbpy)](PF$_6$)**

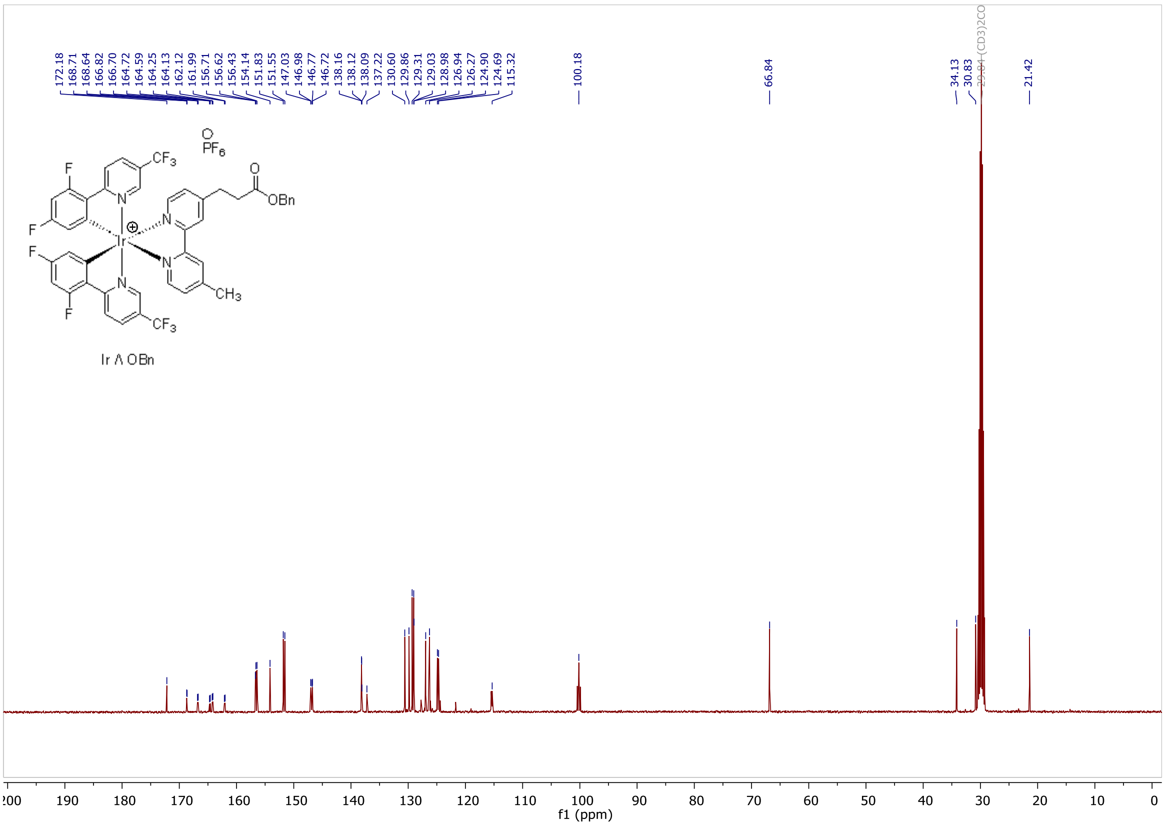

^{19}F NMR (377 MHz, Acetone-d_6) of **Λ[Ir(dF(CF$_3$)ppy)$_2$((^{C2}CO$_2$Bn)dmbpy)](PF$_6$)**

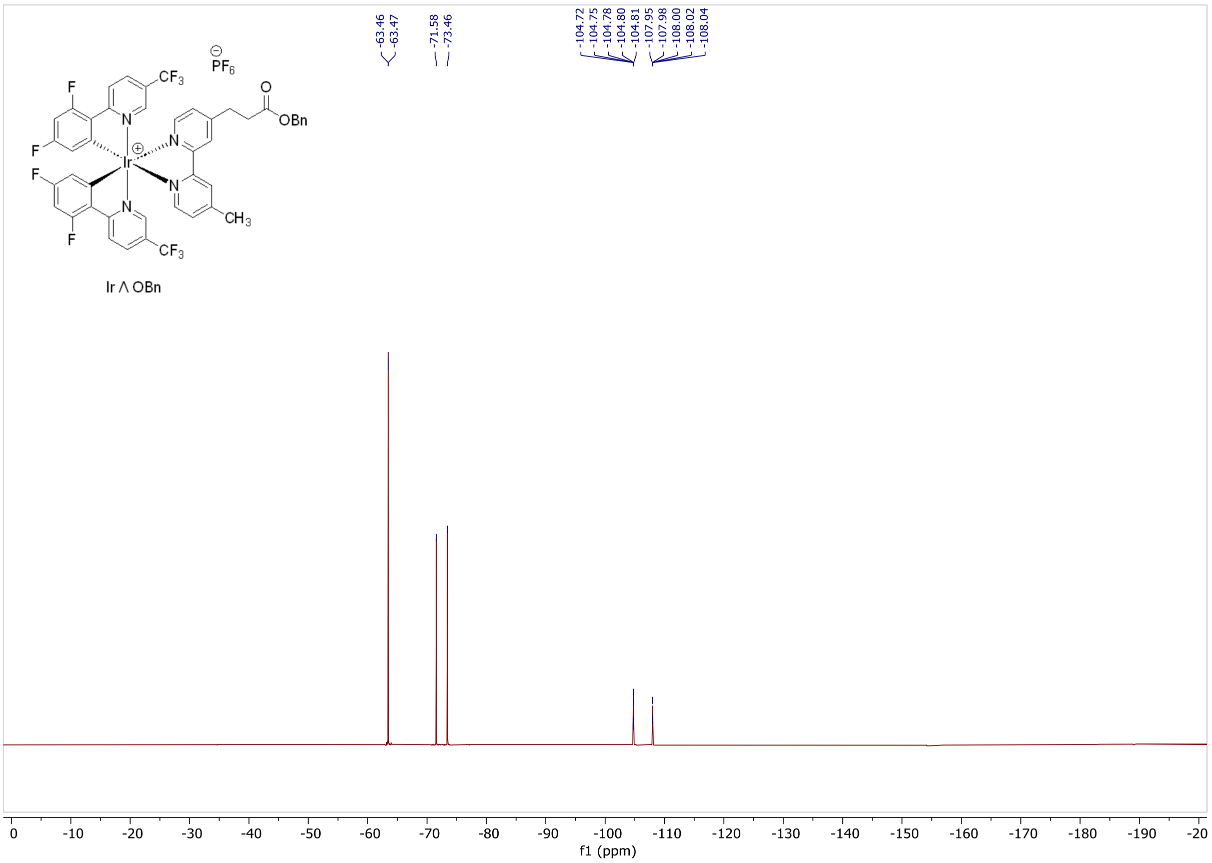

^{1}H NMR (400 MHz, Acetone-d_6) of **Δ[Ir(dF(CF$_3$)ppy)$_2$((^{C2}CO$_2$Bn)dmbpy)](PF$_6$)**

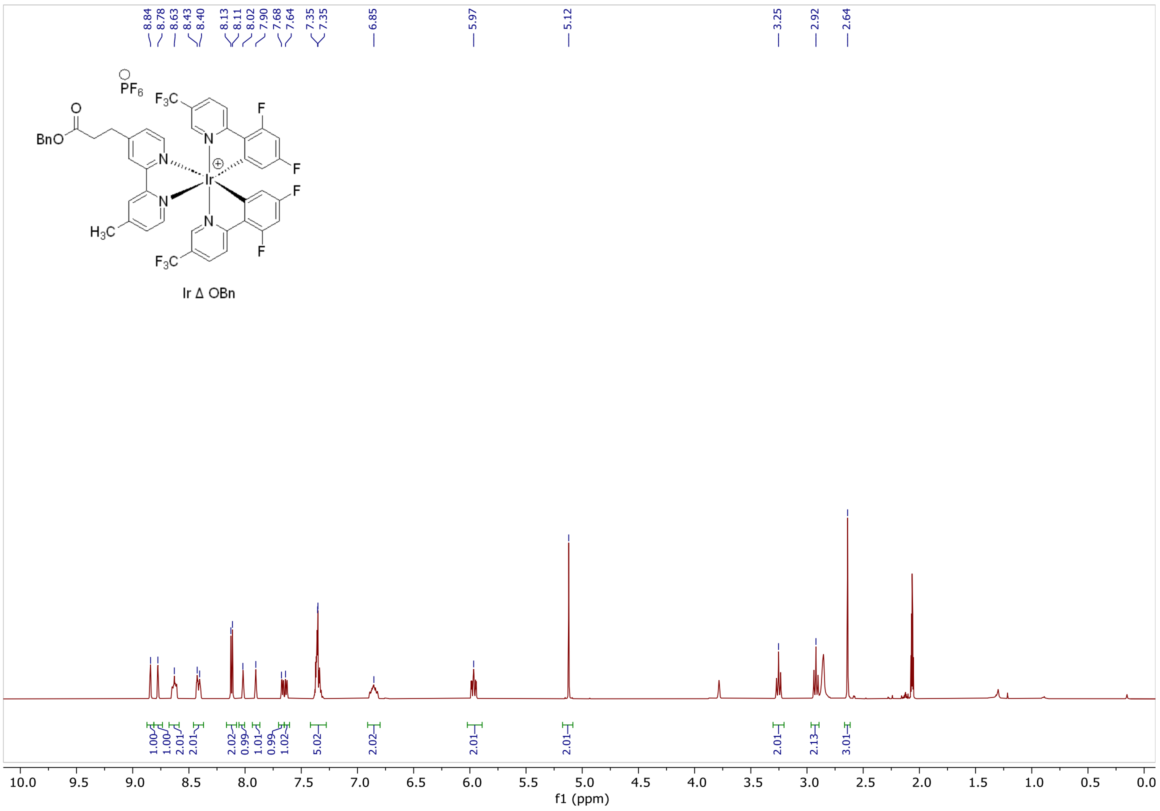

^{13}C NMR (101 MHz, Acetone-d_6) of **Δ[Ir(dF(CF$_3$)ppy)$_2$((C2CO$_2$Bn)dmbpy)](PF$_6$)**

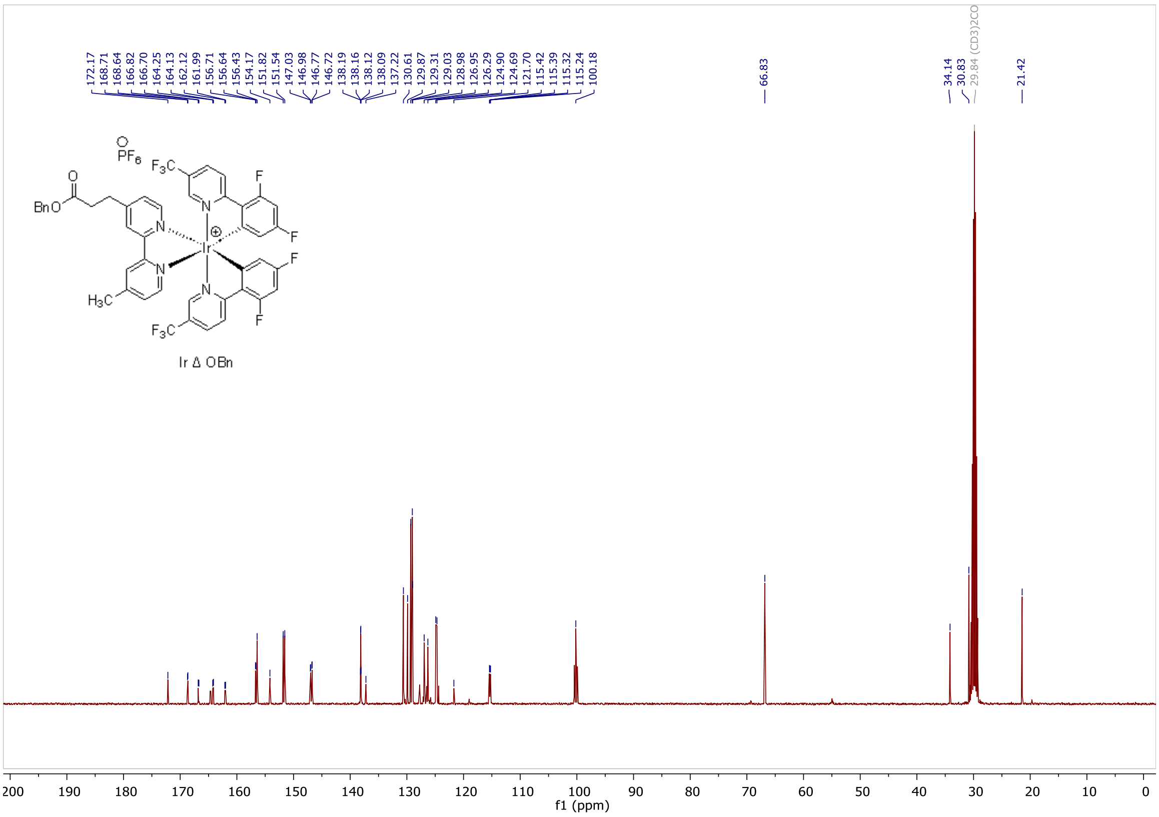

^{19}F NMR (377 MHz, Acetone-d_6) of **Δ[Ir(dF(CF$_3$)ppy)$_2$((C2CO$_2$Bn)dmbpy)](PF$_6$)**

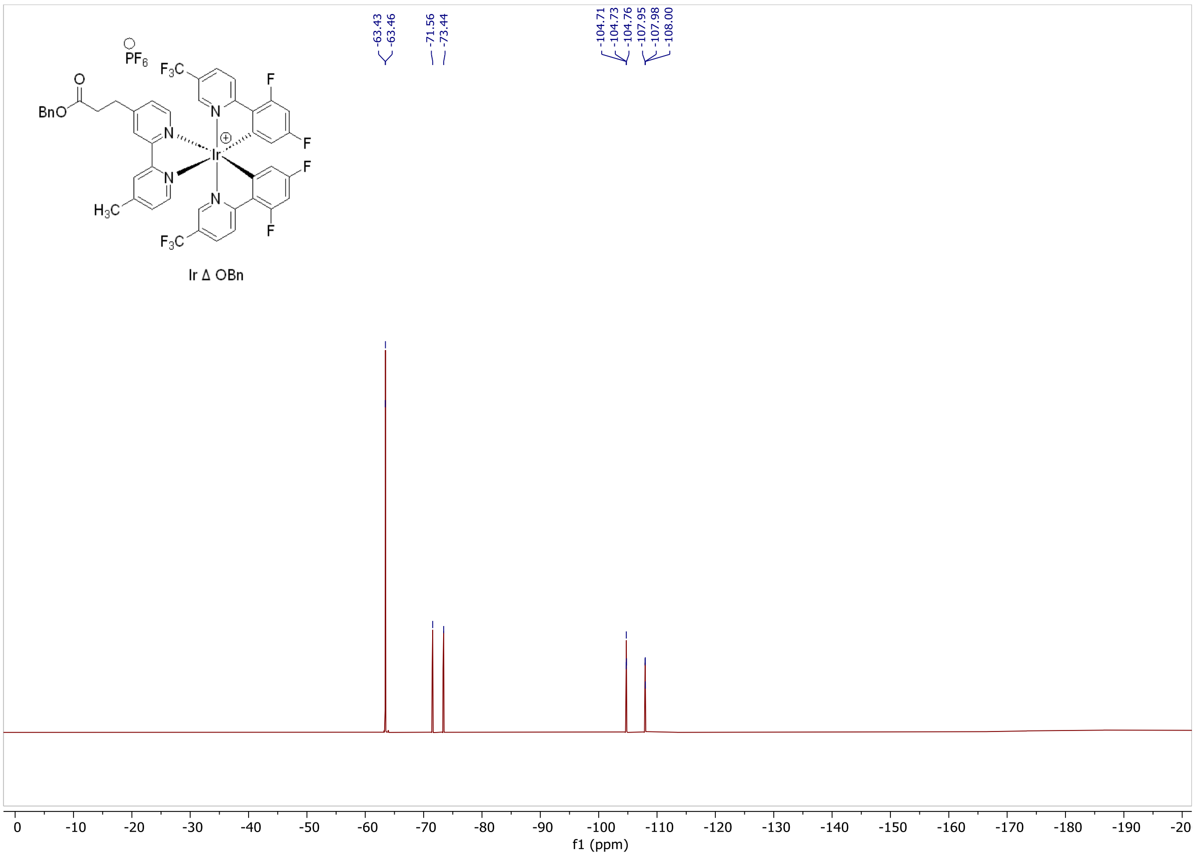

^{1}H NMR (400 MHz, Acetone-d_6) of **Λ[Ir(dF(CF$_3$)ppy)$_2$((C2CO$_2$H)dmbpy)](PF$_6$)**

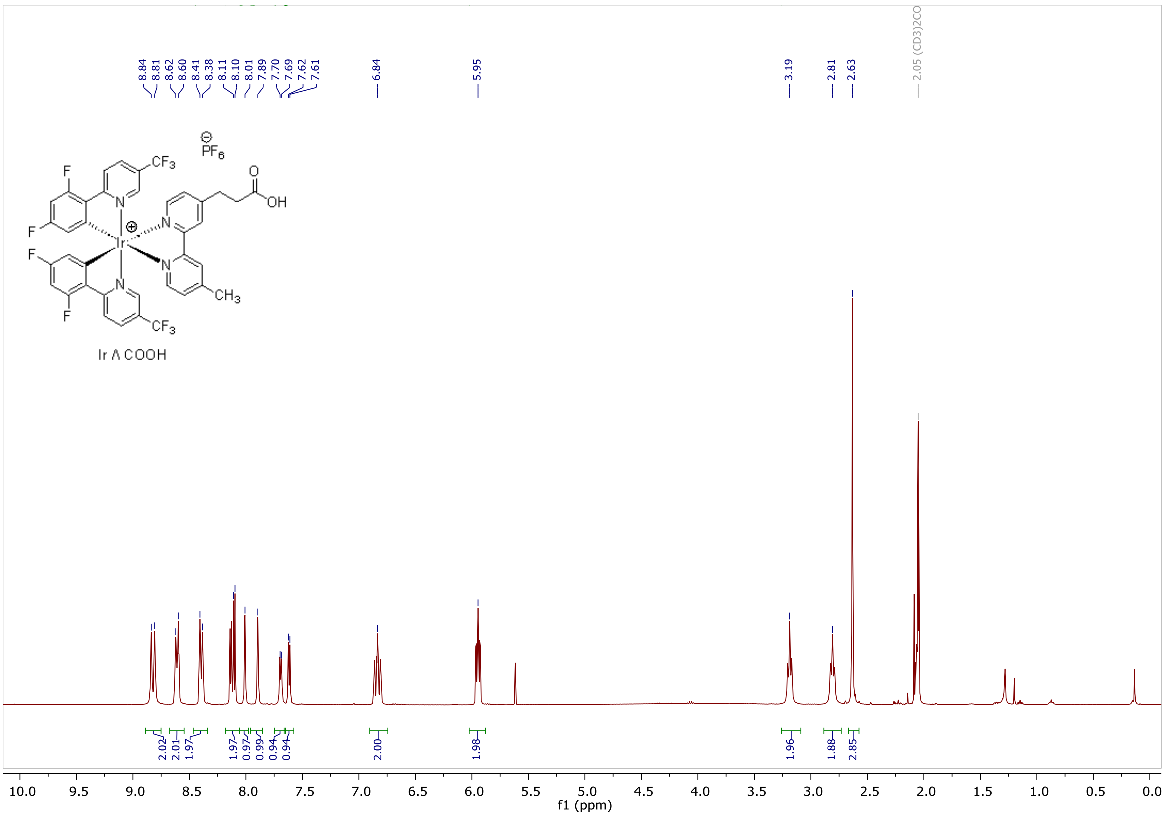

^{13}C NMR (101 MHz, Acetone-d_6) of **Λ[Ir(dF(CF$_3$)ppy)$_2$((C2CO$_2$H)dmbpy)](PF$_6$)**

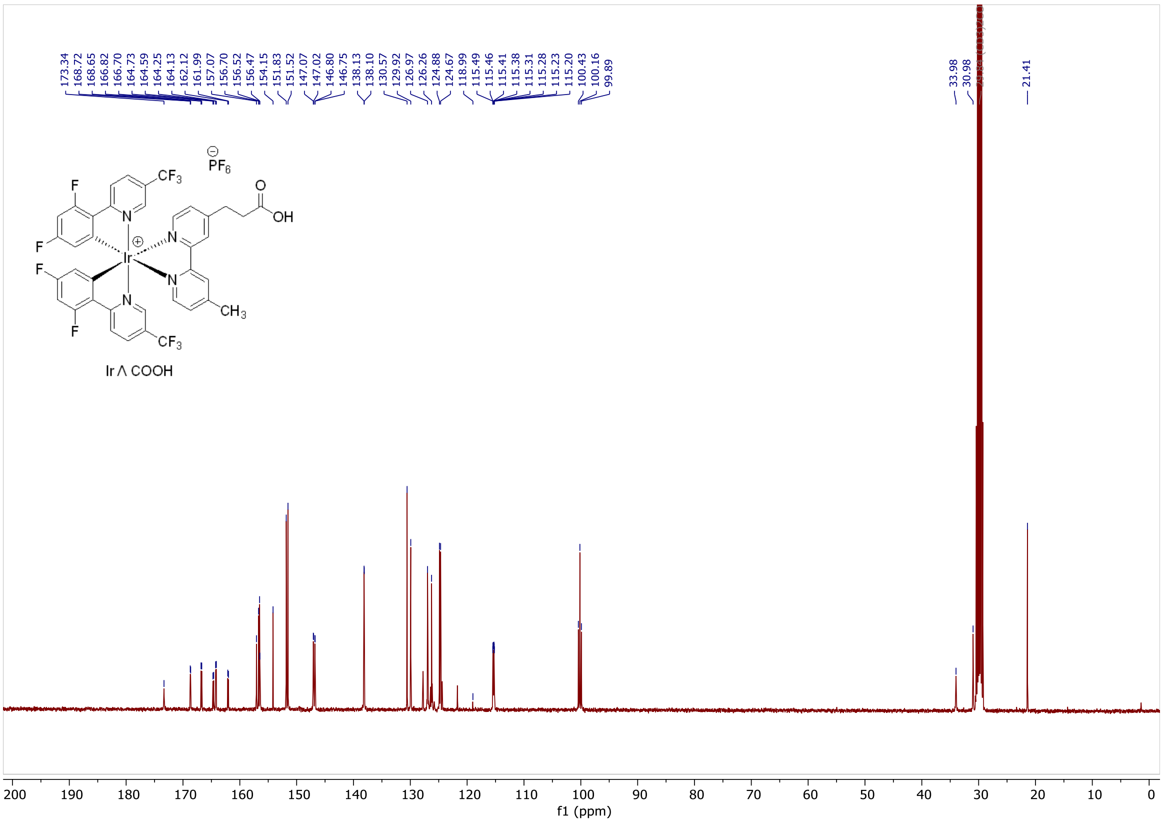

^{19}F NMR (377 MHz, Acetone-*d*$_6$) of **Λ[Ir(dF(CF$_3$)ppy)$_2$((^{C2}CO$_2$H)dmbpy)](PF$_6$)**

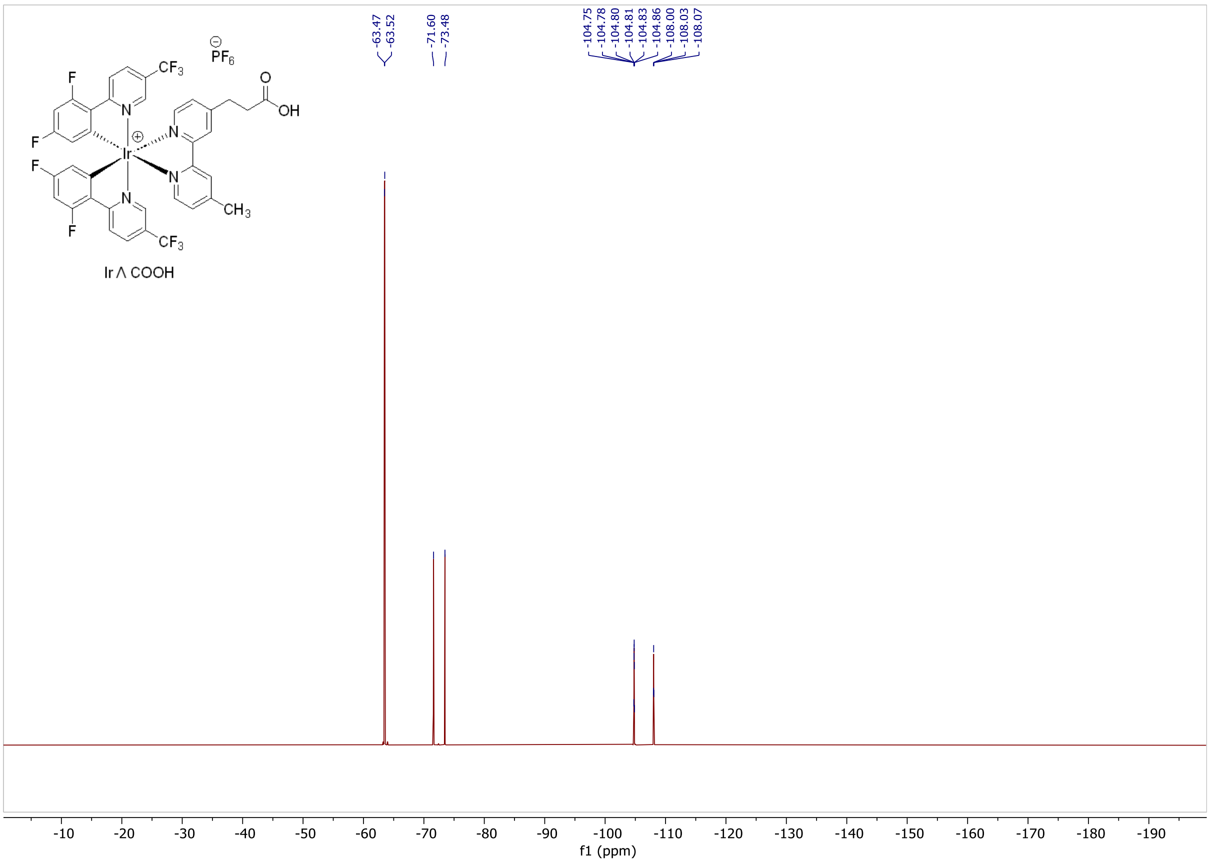

^{1}H NMR (400 MHz, Acetone-*d*$_6$) of **Δ[Ir(dF(CF$_3$)ppy)$_2$(^{C2}CO$_2$H)dmbpy)](PF$_6$)**

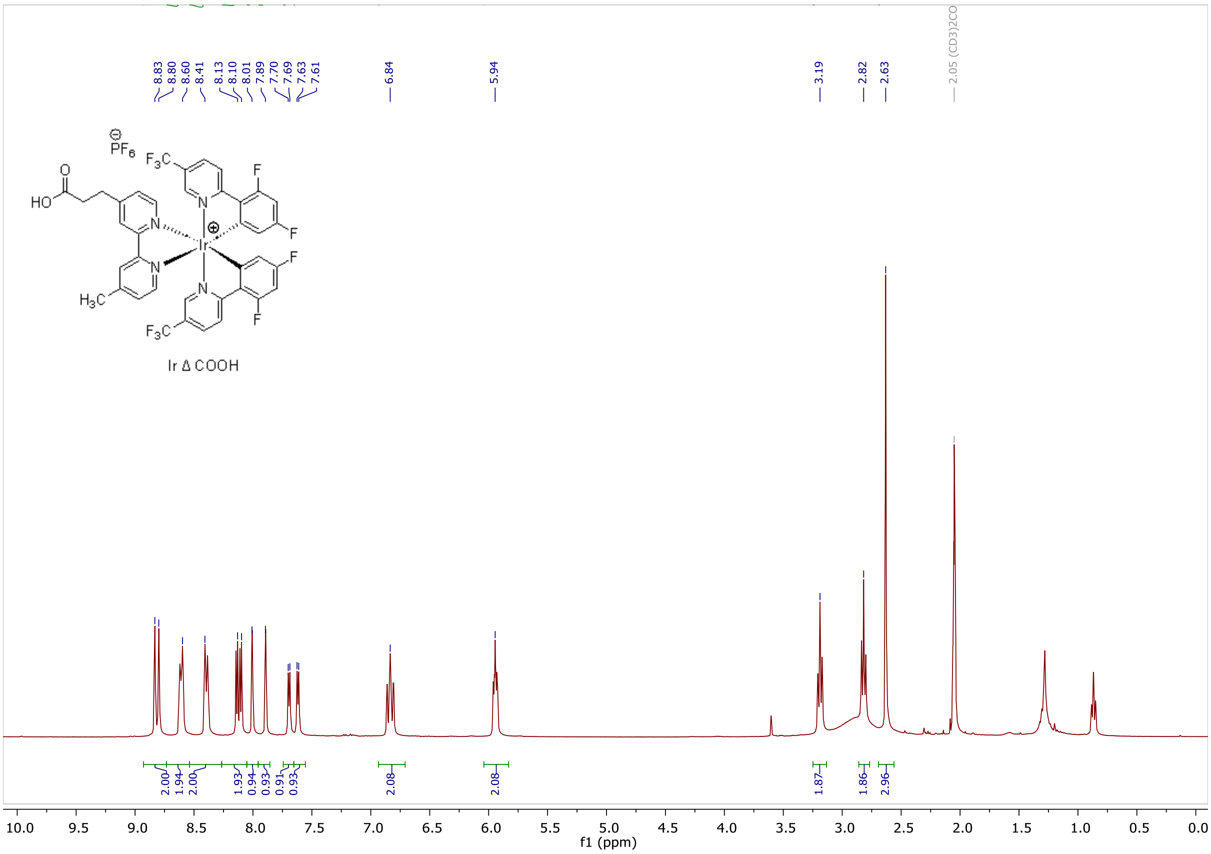

^{13}C NMR (101 MHz, Acetone-d_6) of **Δ[Ir(dF(CF$_3$)ppy)$_2$((^{C2}CO$_2$H)dmbpy)](PF$_6$)**

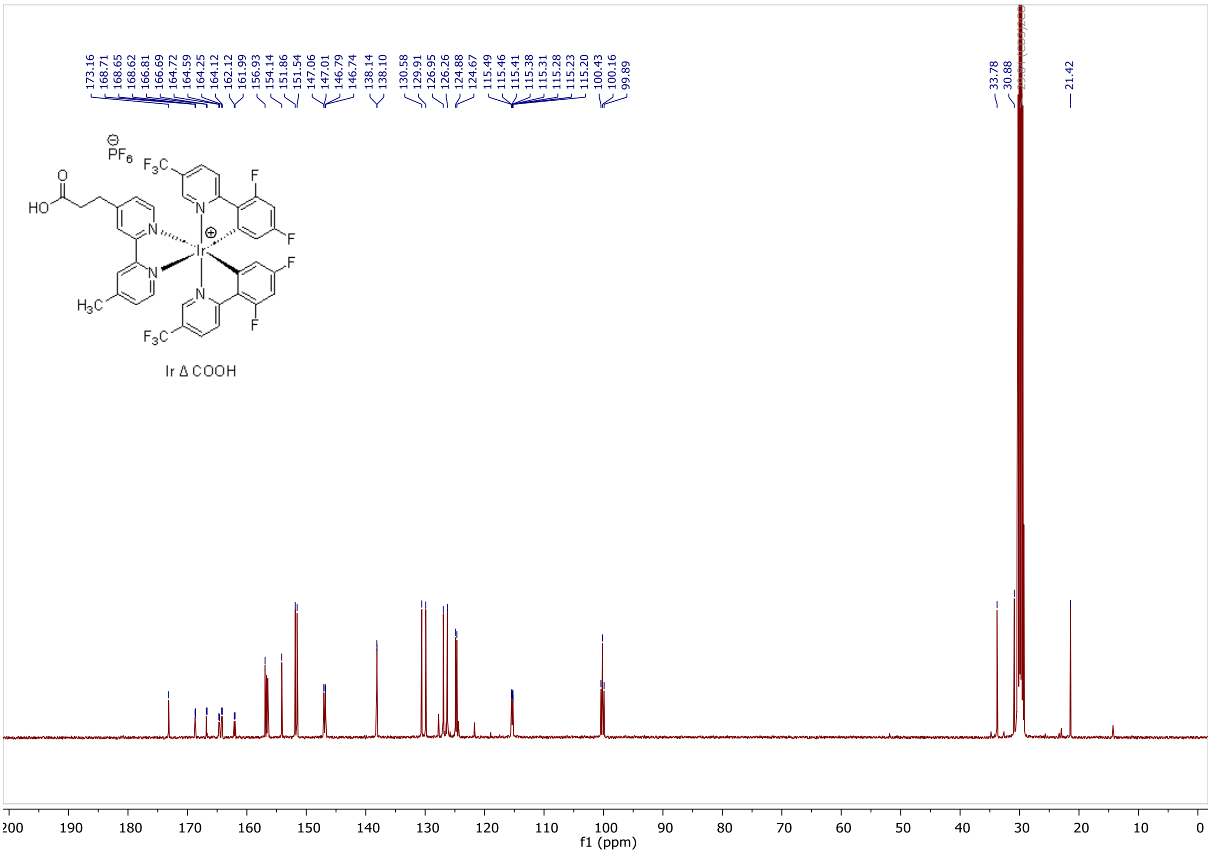

^{19}F NMR (377 MHz, Acetone-d_6) of **Δ[Ir(dF(CF$_3$)ppy)$_2$((^{C2}CO$_2$H)dmbpy)](PF$_6$)**

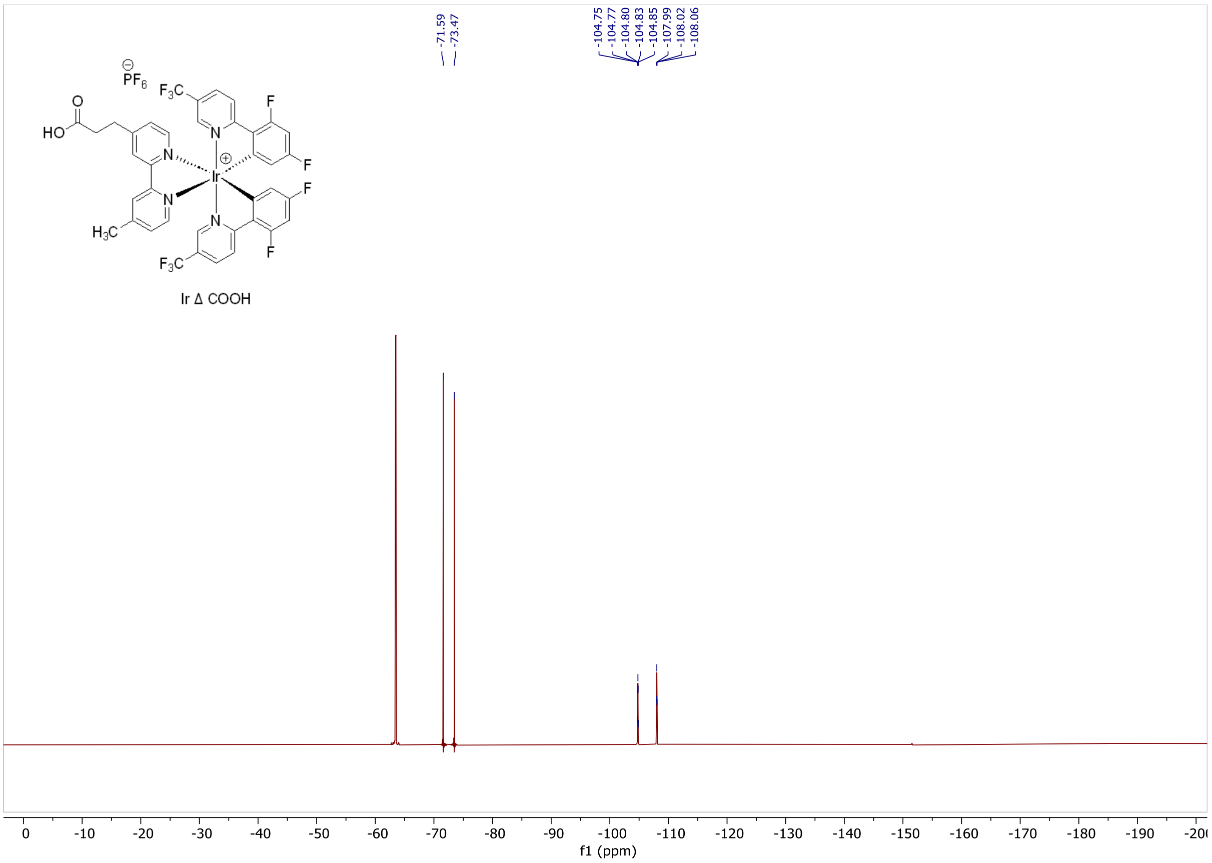

3. NMR of substrates

^1H NMR (400 MHz, DMSO-d_6) of **ethyl 3-hydroxy-2-oxo-1,2-dihydro-1,8-naphthyridine-4-carboxylate**

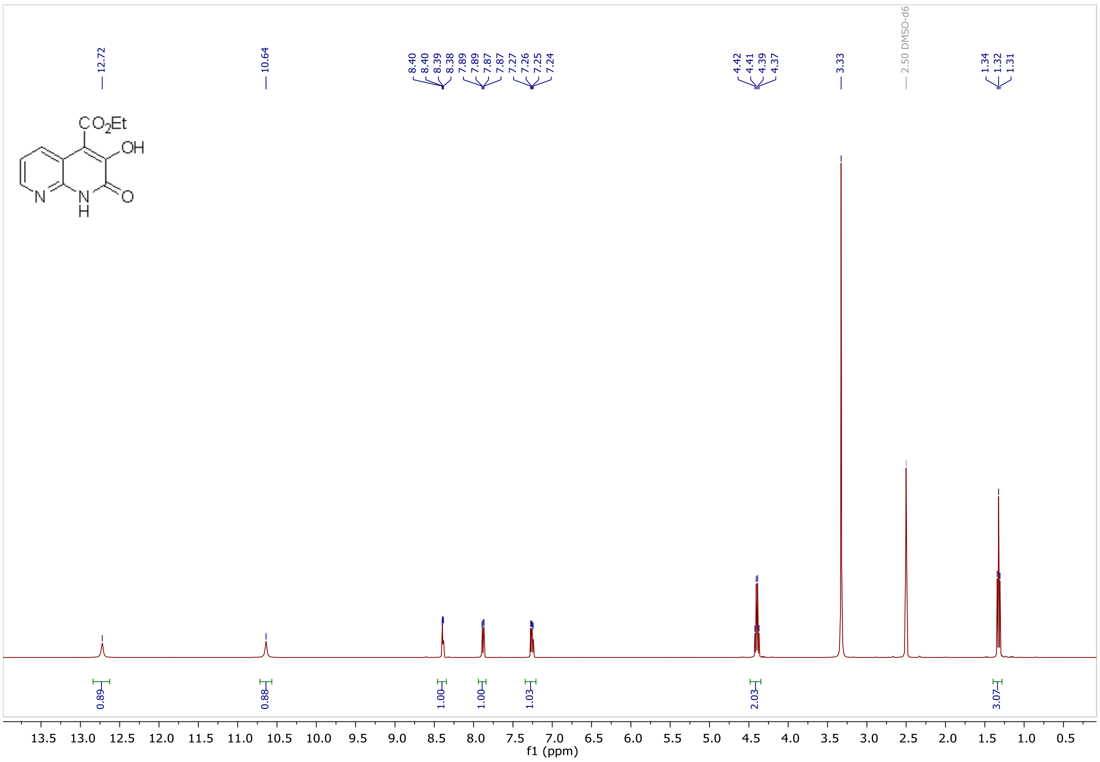

^{13}C NMR (151 MHz, DMSO-d_6) of **ethyl 3-hydroxy-2-oxo-1,2-dihydro-1,8-naphthyridine-4-carboxylate**

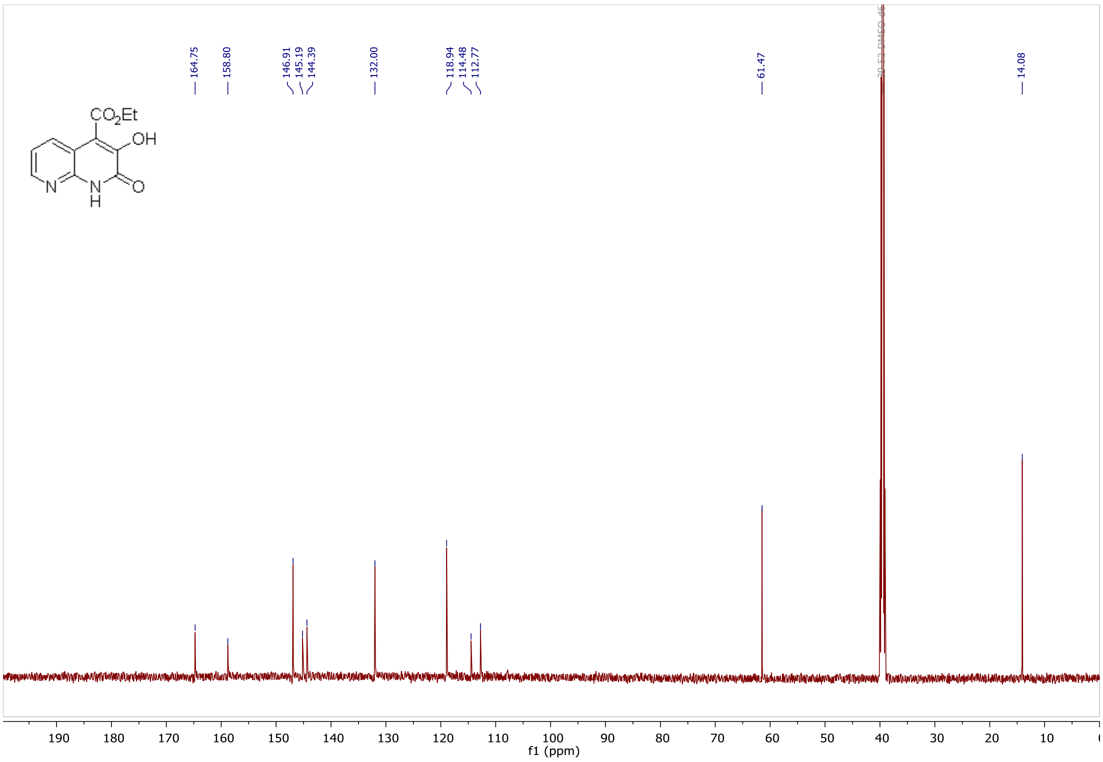

^{1}H NMR (400 MHz, DMSO-d_6) of **ethyl 1-ethyl-3-hydroxy-2-oxo-1,2-dihydroquinoline-4-carboxylate**

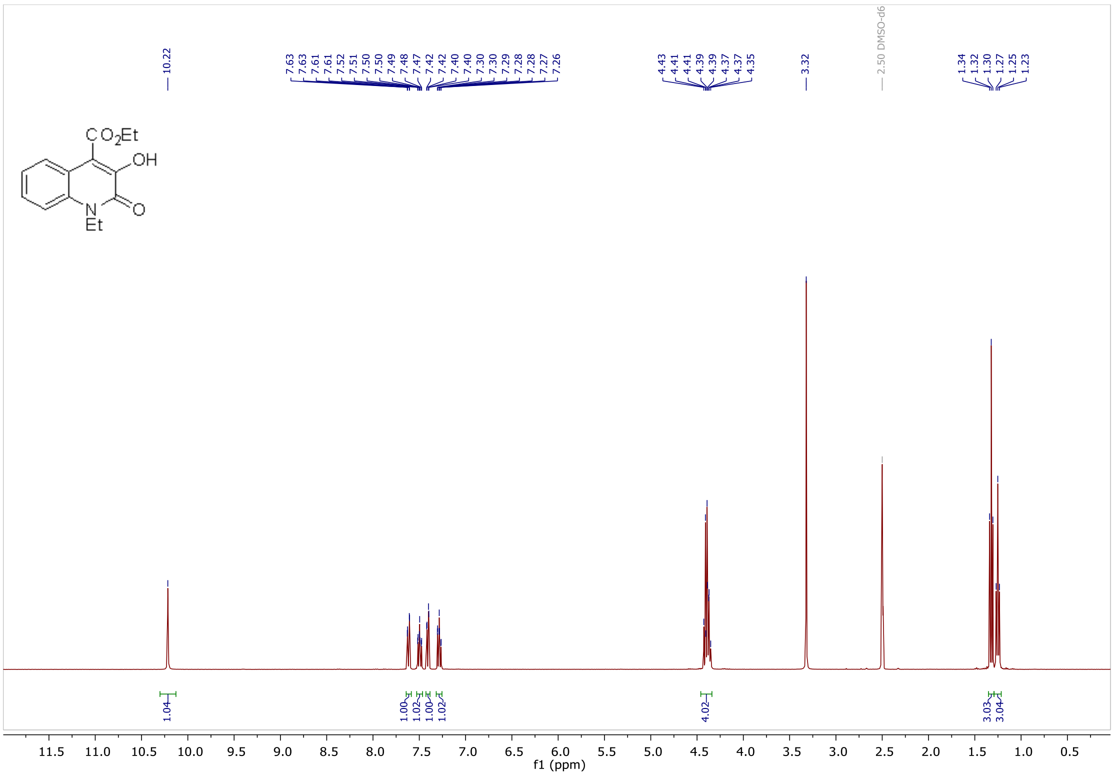

^{13}C NMR (151 MHz, DMSO-d_6) of **ethyl 1-ethyl-3-hydroxy-2-oxo-1,2-dihydroquinoline-4-carboxylate**

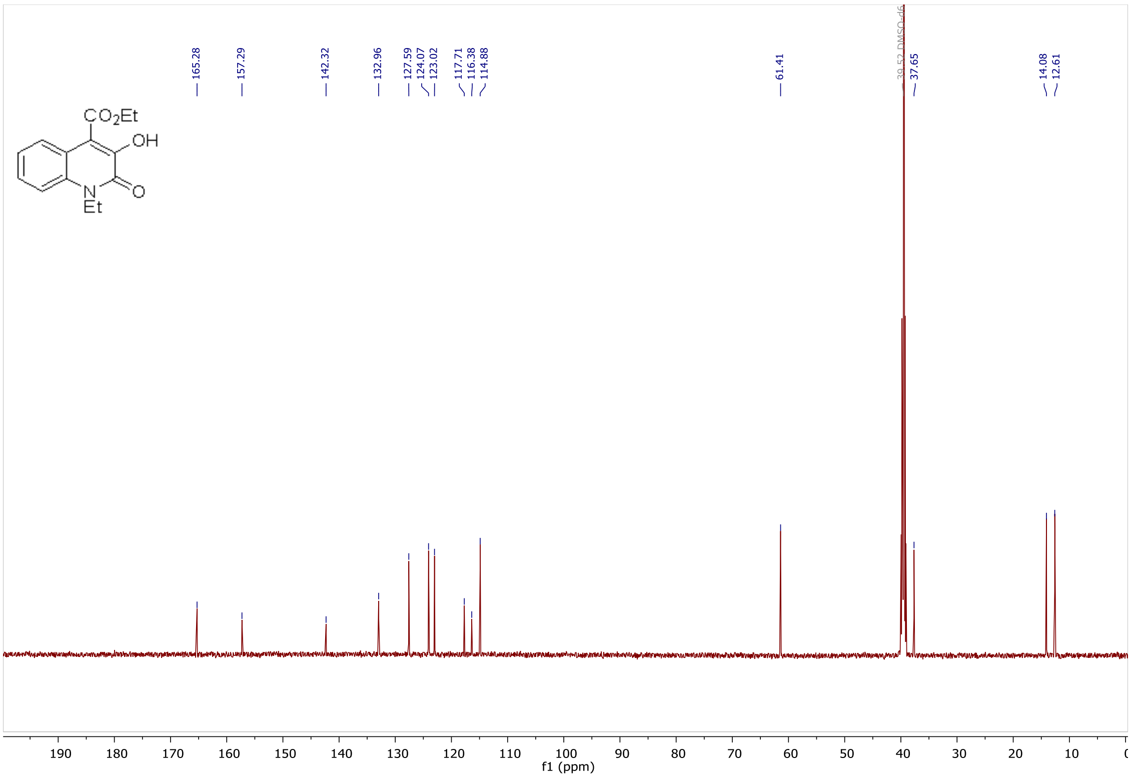

^{1}H NMR (400 MHz, $CDCl_3$) of **1b**

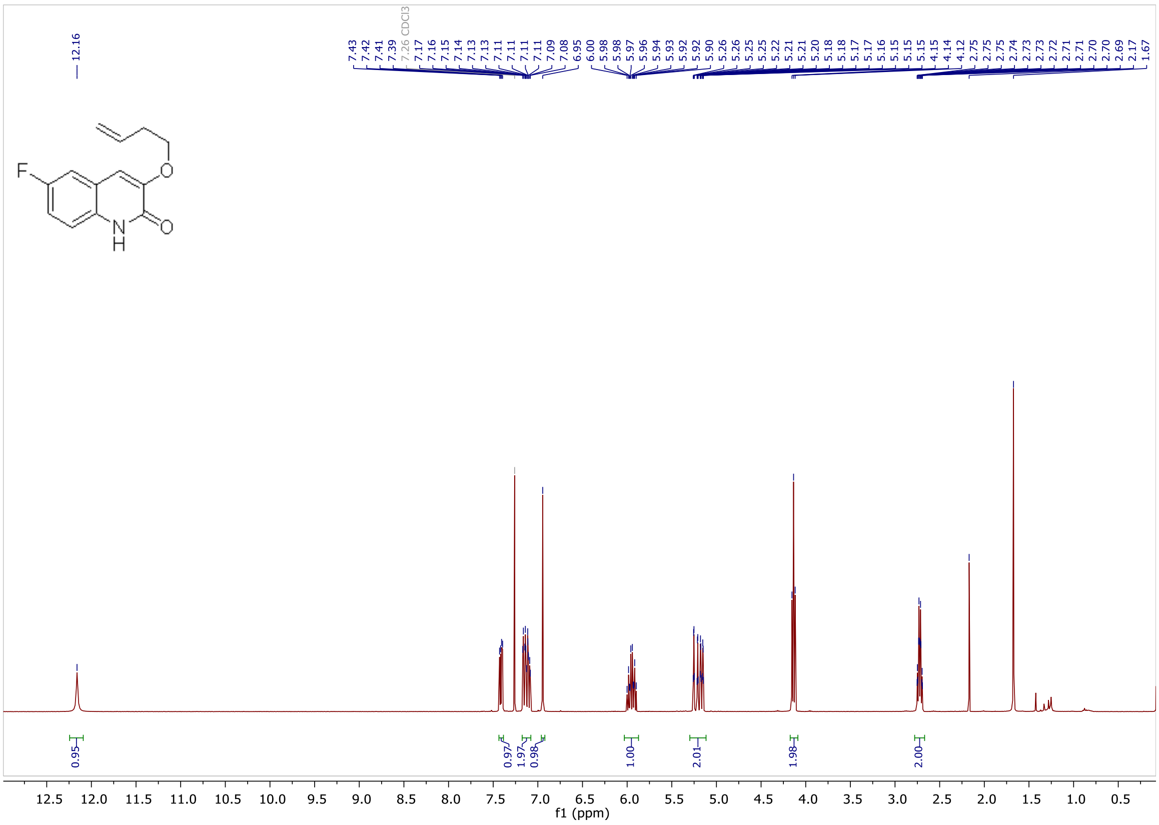

^{13}C NMR (101 MHz, $CDCl_3$) of **1b**

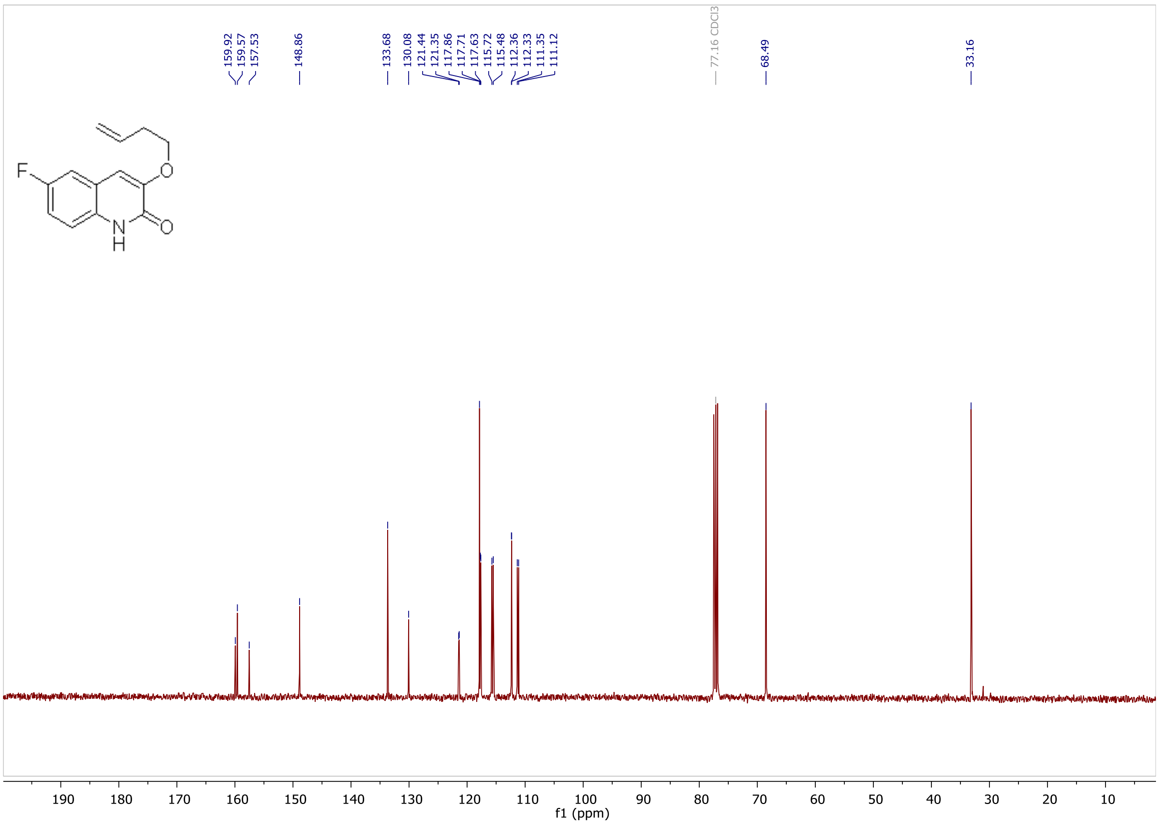

^{19}F NMR (376 MHz, CDCl$_3$) of **1b**

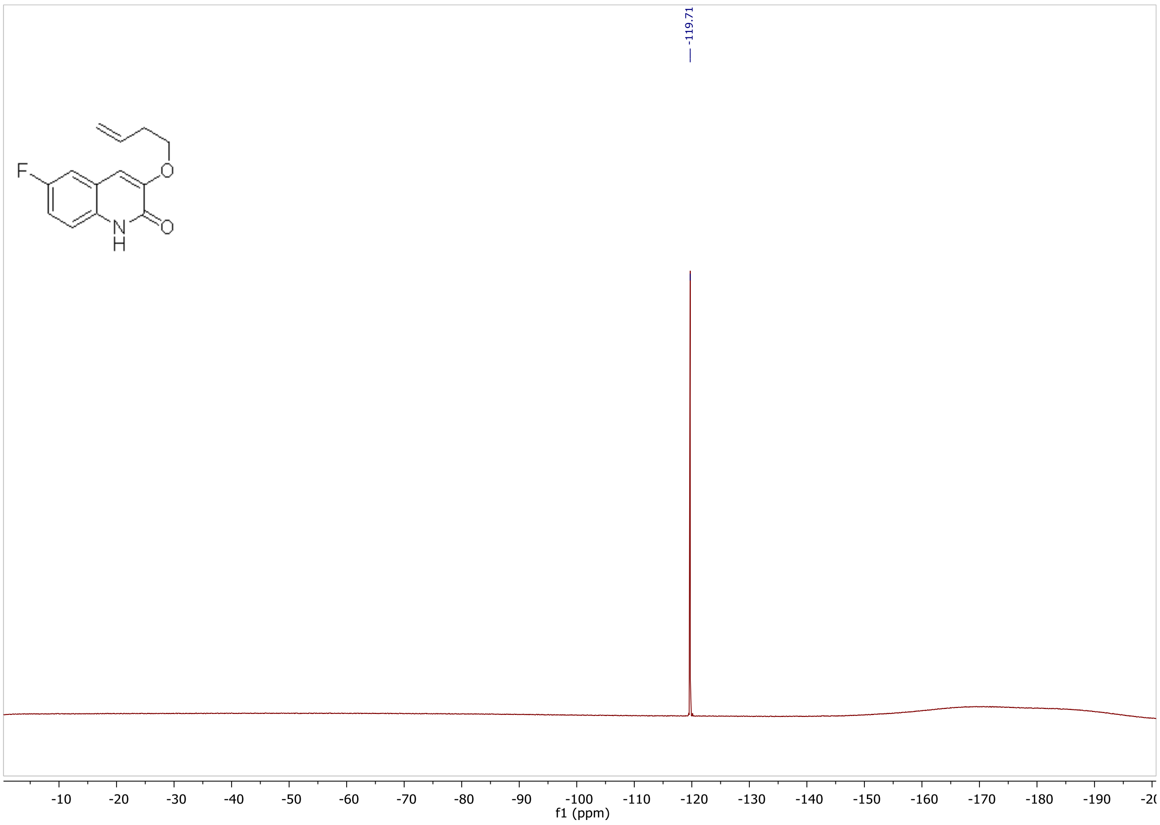

^{1}H NMR (400 MHz, CDCl$_3$) of **1g**

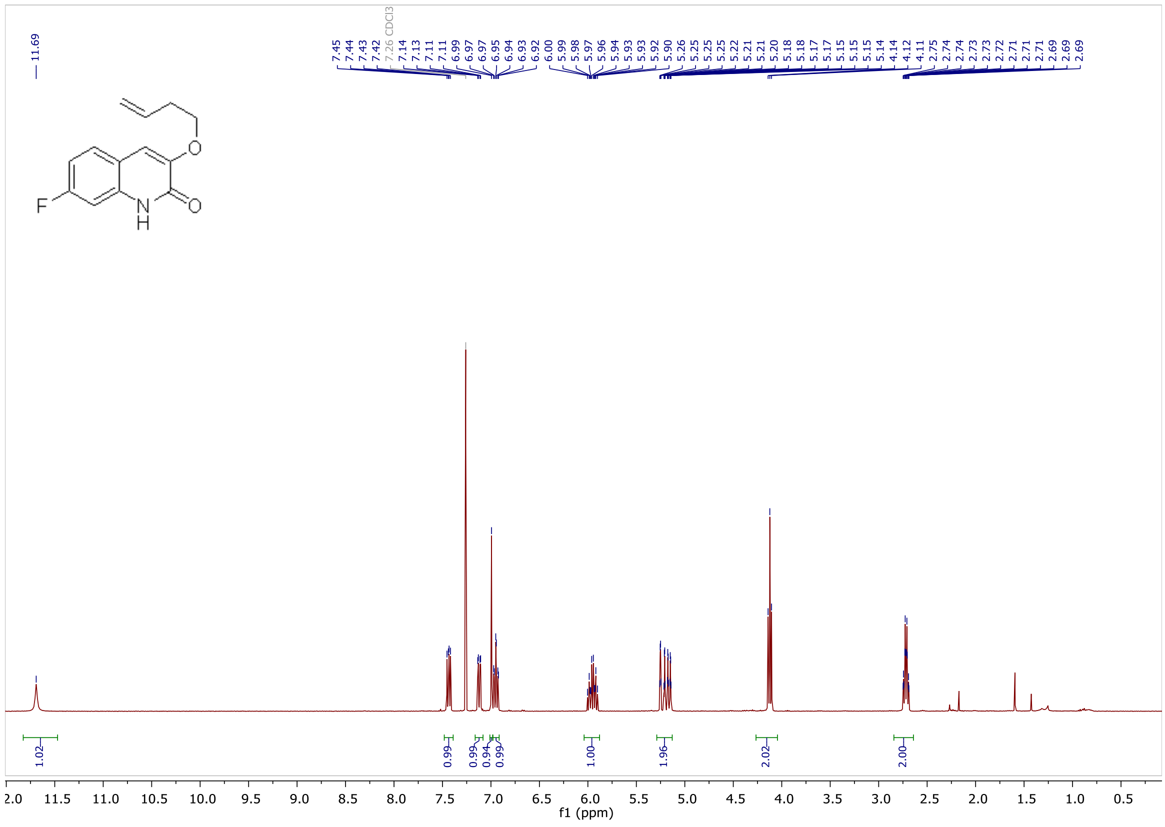

^{13}C NMR (101 MHz, $CDCl_3$) of **1g**

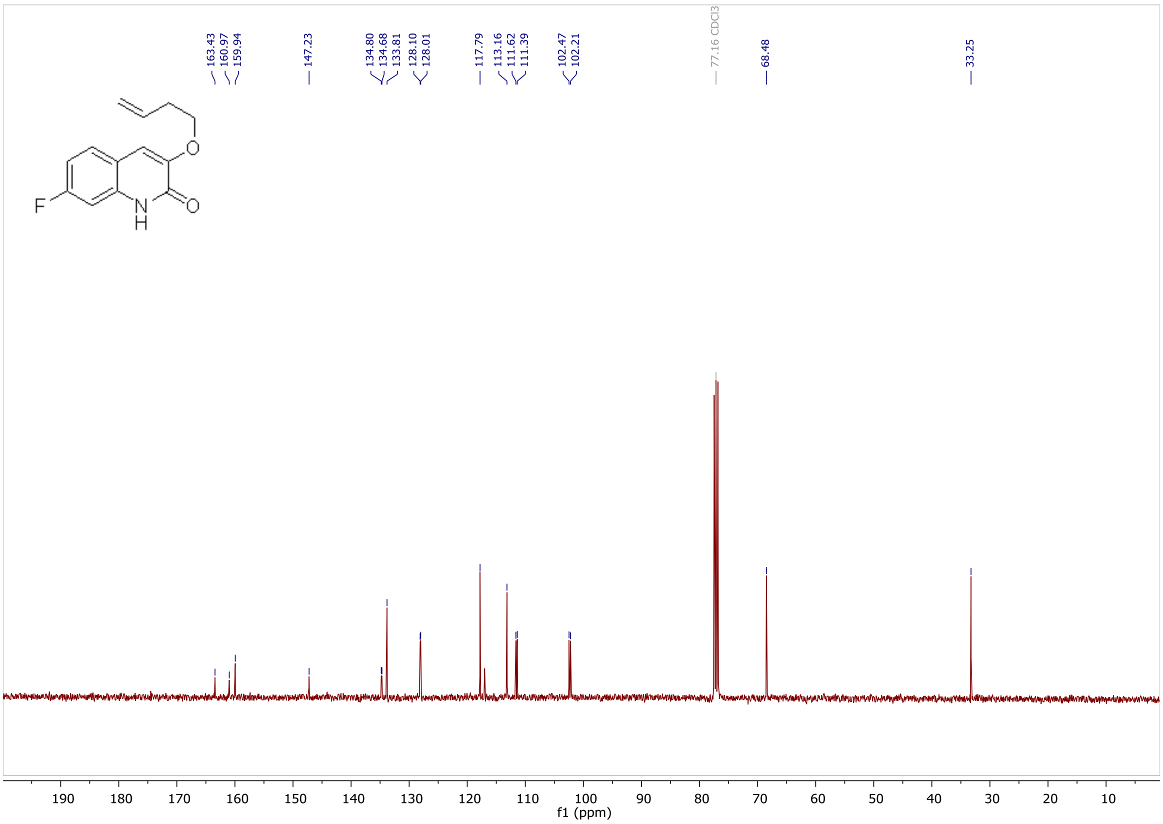

^{19}F NMR (376 MHz, $CDCl_3$) of **1g**

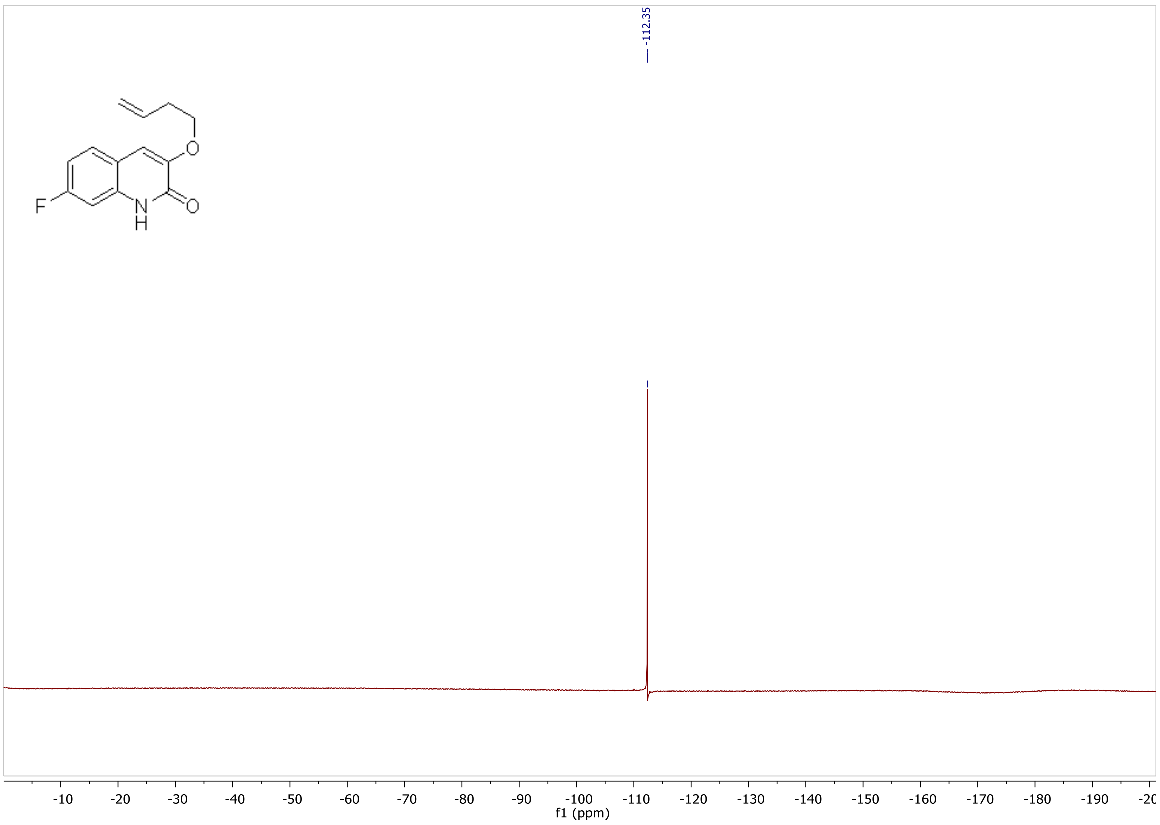

^{1}H NMR (400 MHz, $CDCl_3$) of **1j**

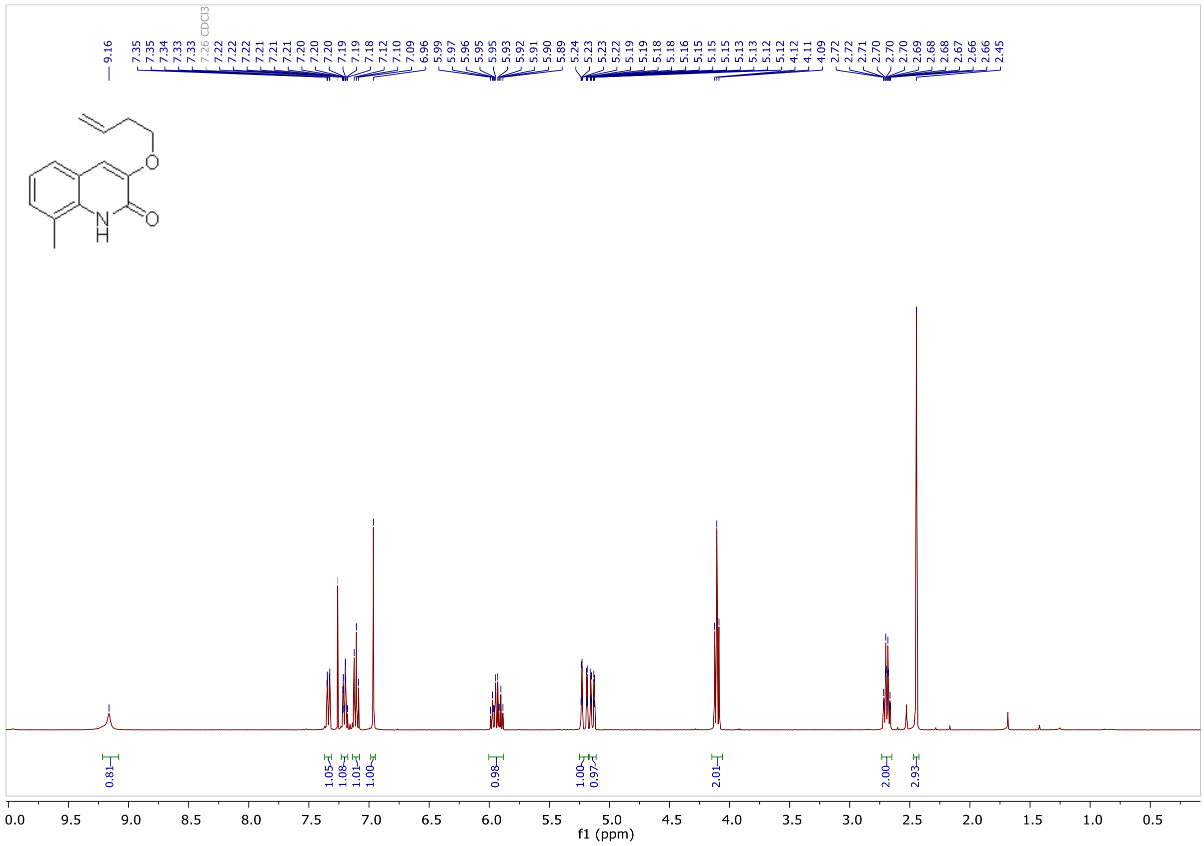

^{13}C NMR (101 MHz, $CDCl_3$) of **1j**

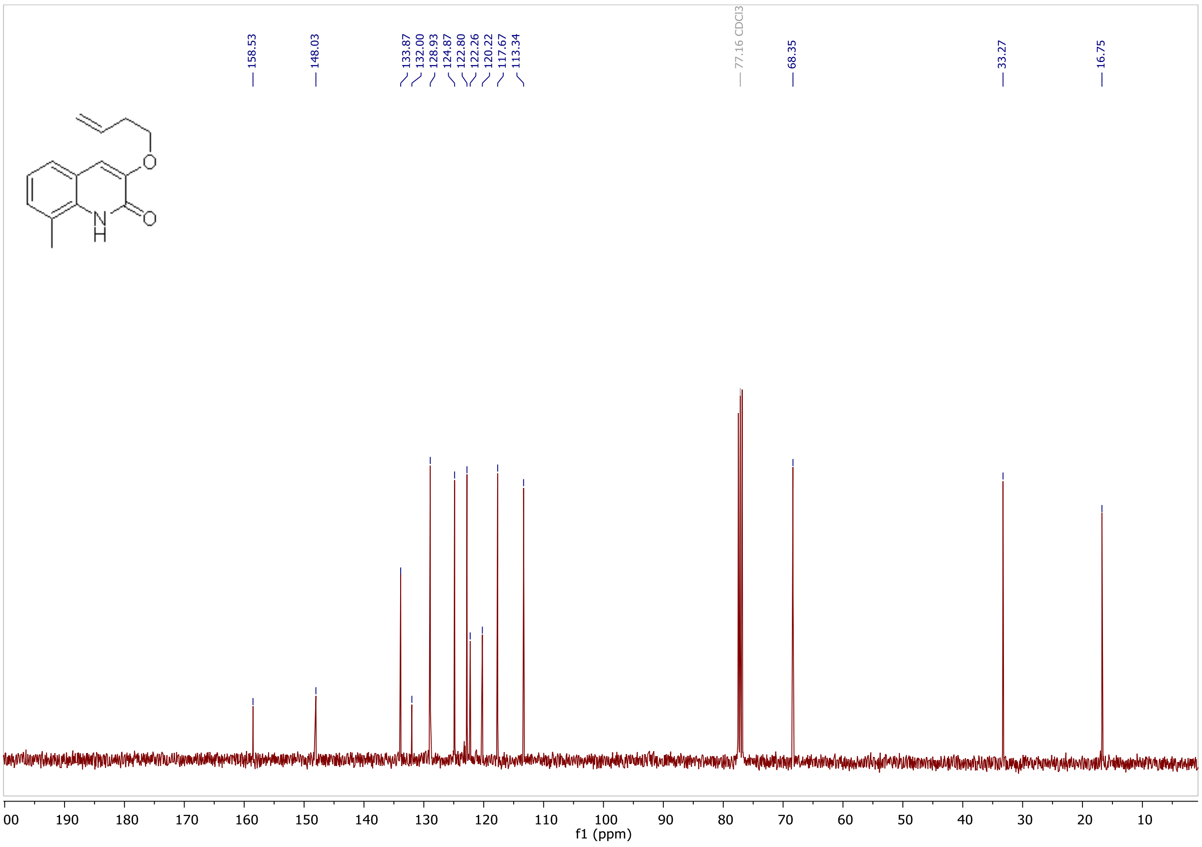

^{1}H NMR (400 MHz, $CDCl_3$) of **1k**

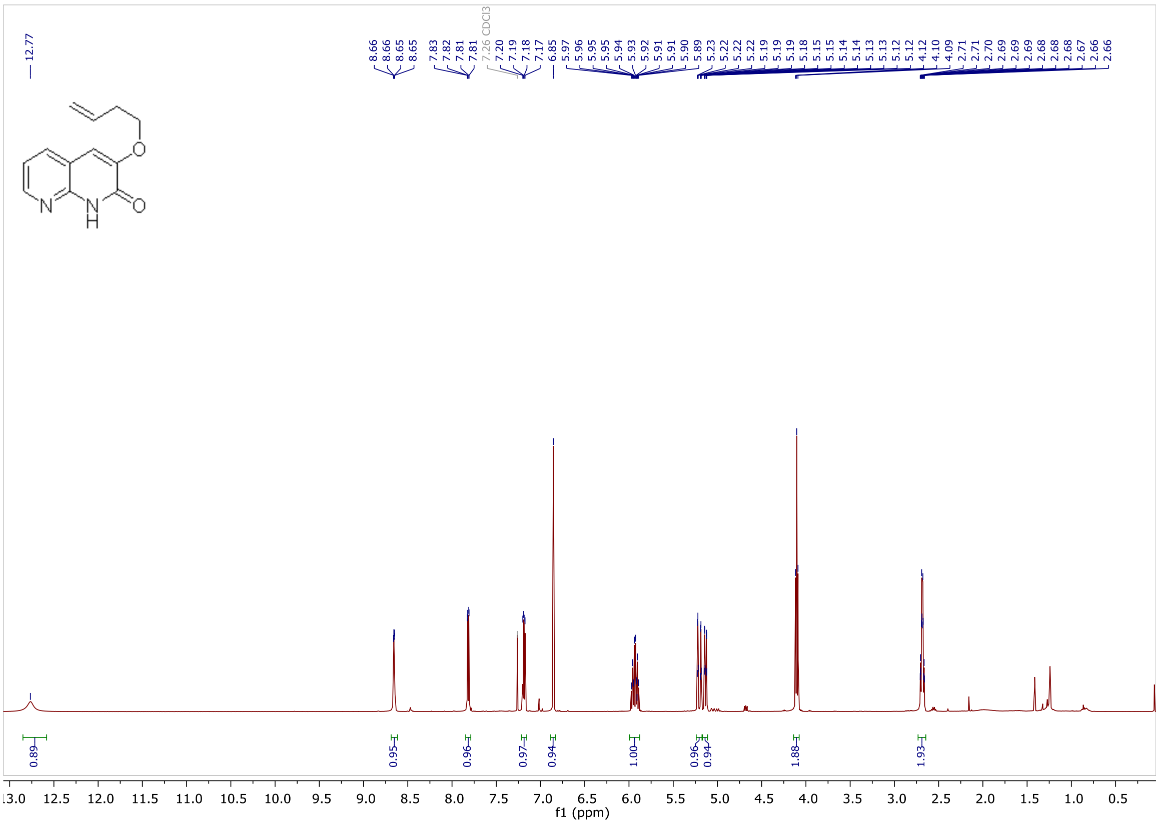

^{13}C NMR (101 MHz, $CDCl_3$) of **1k**

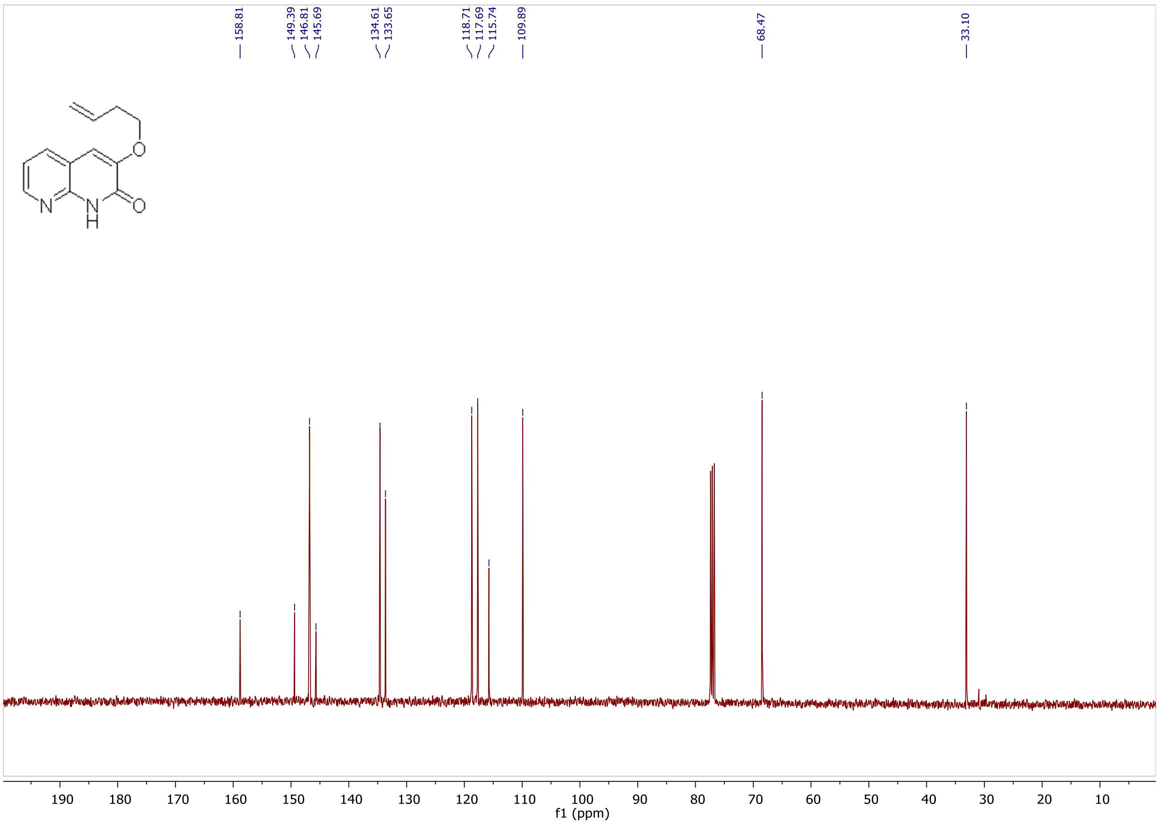

^{1}H NMR (400 MHz, $CDCl_3$) of **1n**

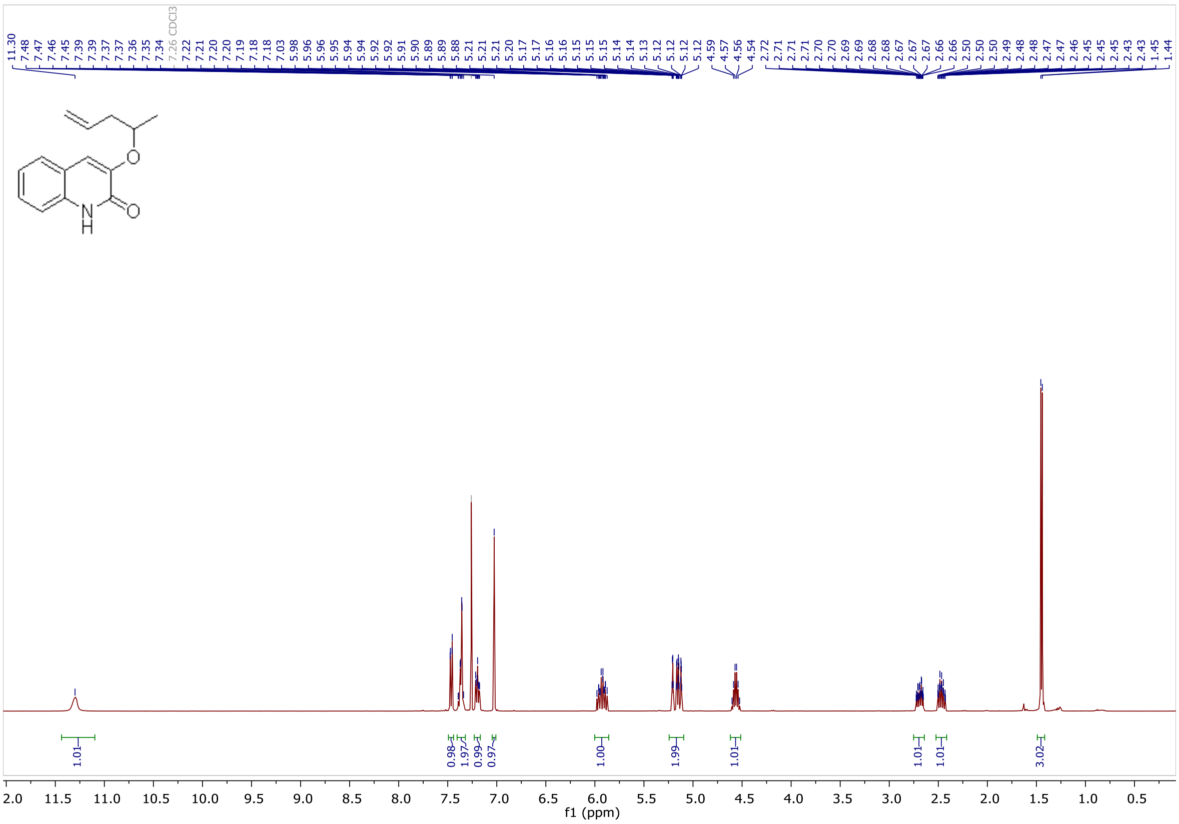

^{13}C NMR (101 MHz, $CDCl_3$) of **1n**

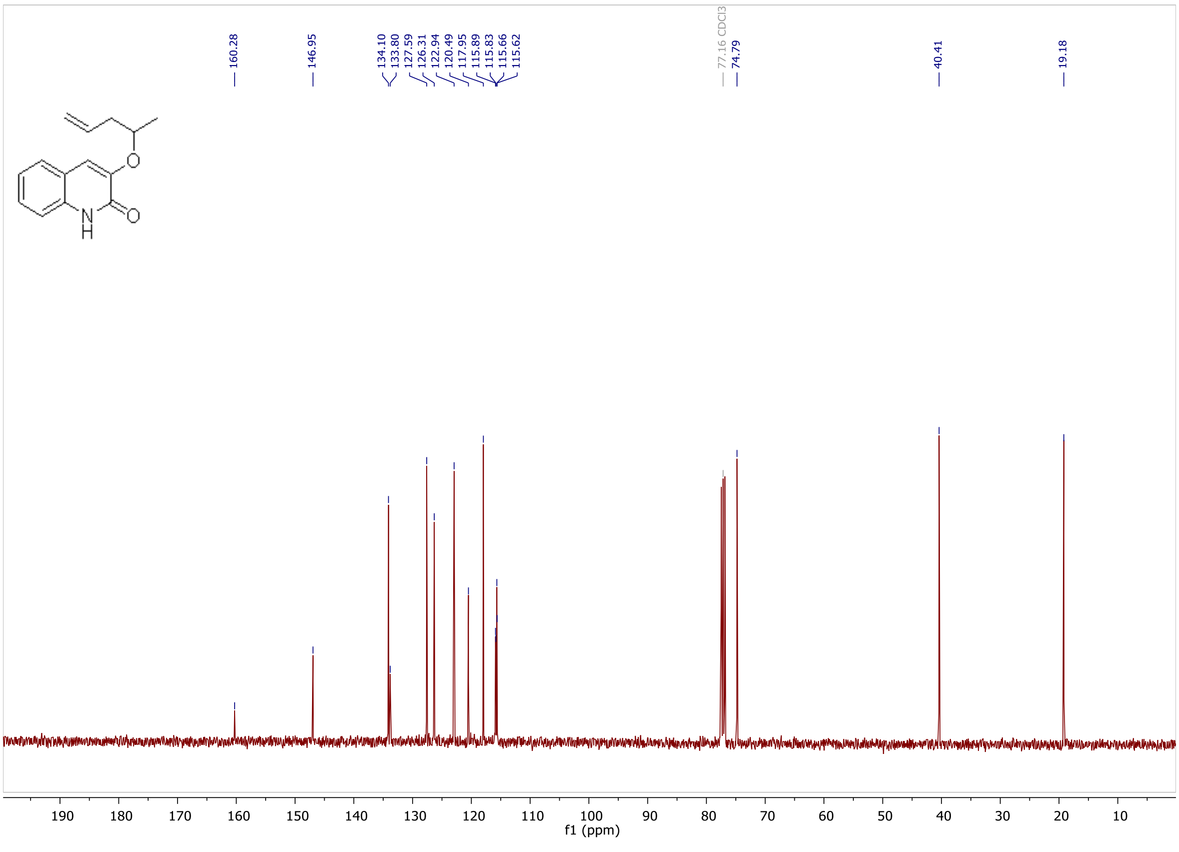

^{1}H NMR (400 MHz, CDCl3) of **1p**

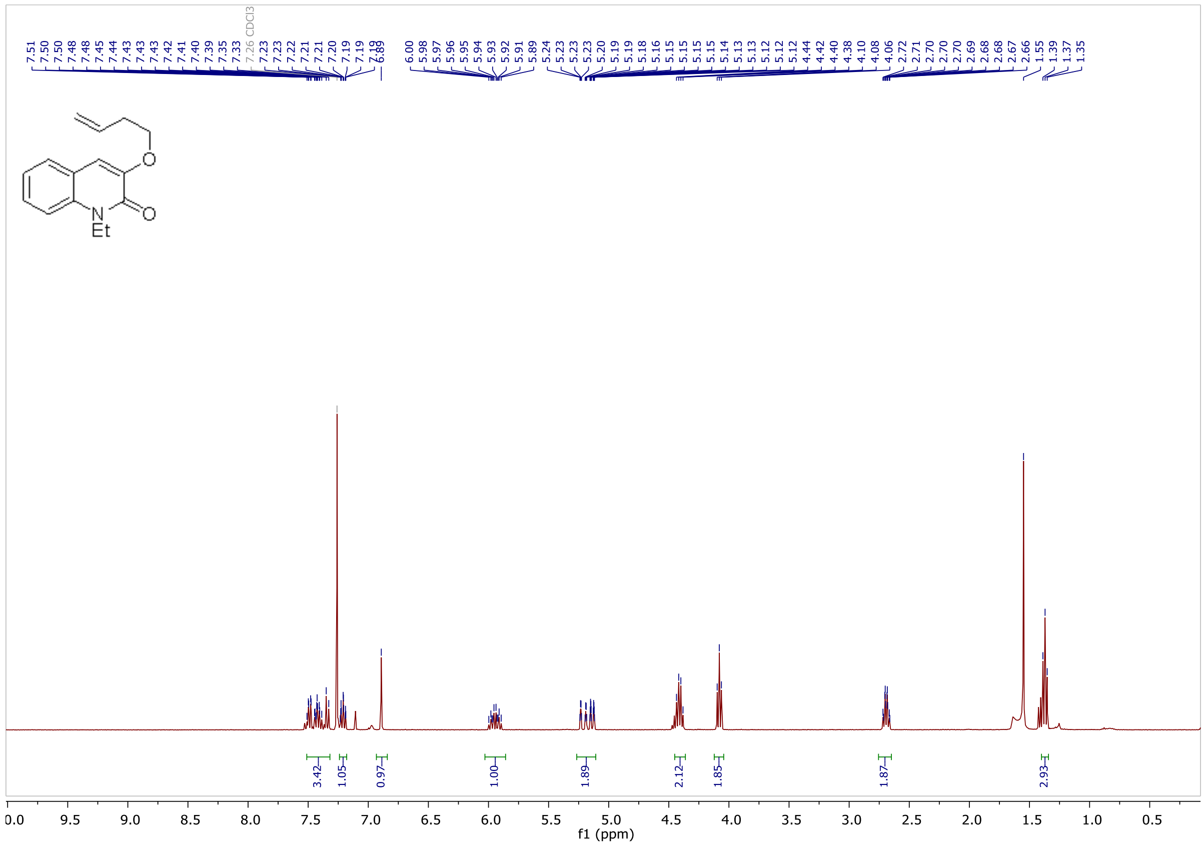

^{13}C NMR (101 MHz, CDCl3) of **1p**

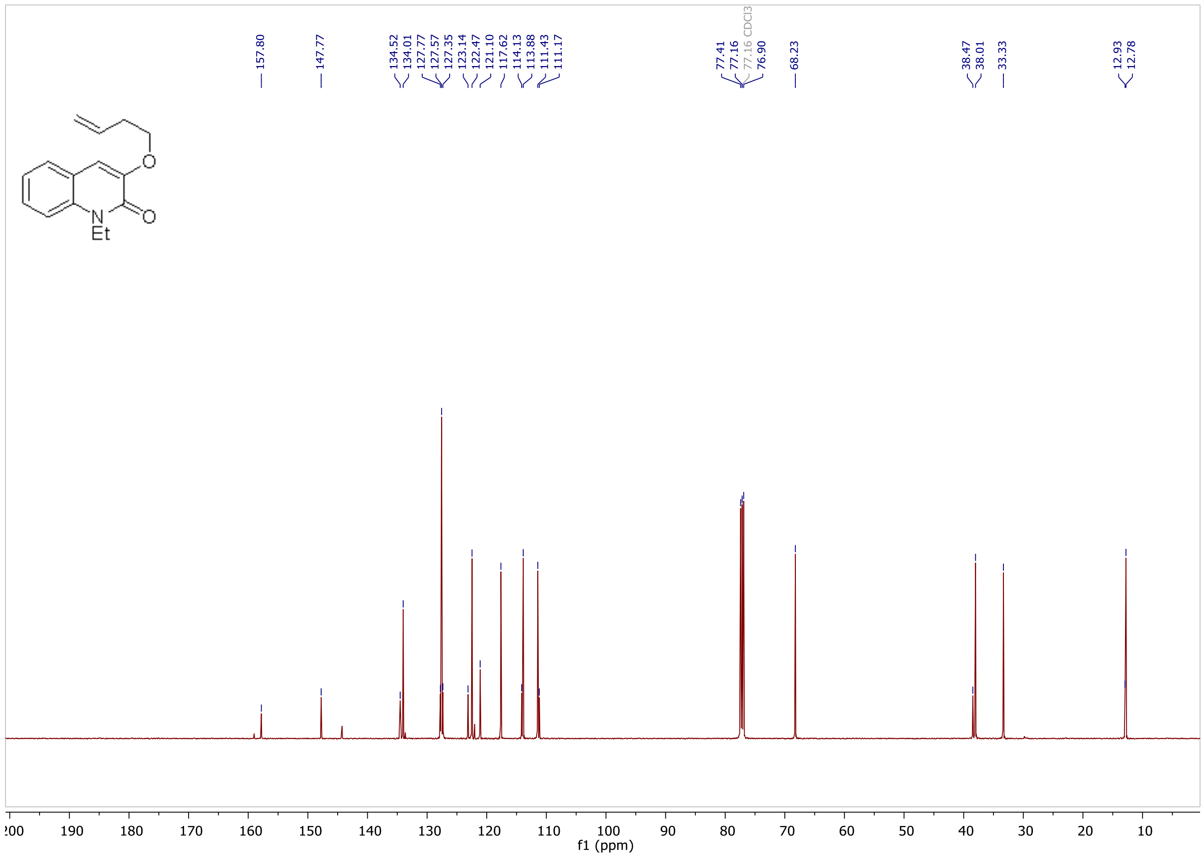

4. NMR of [2+2] photocycloaddition products

^{1}H NMR (500 MHz, $CDCl_3$) of **2a**

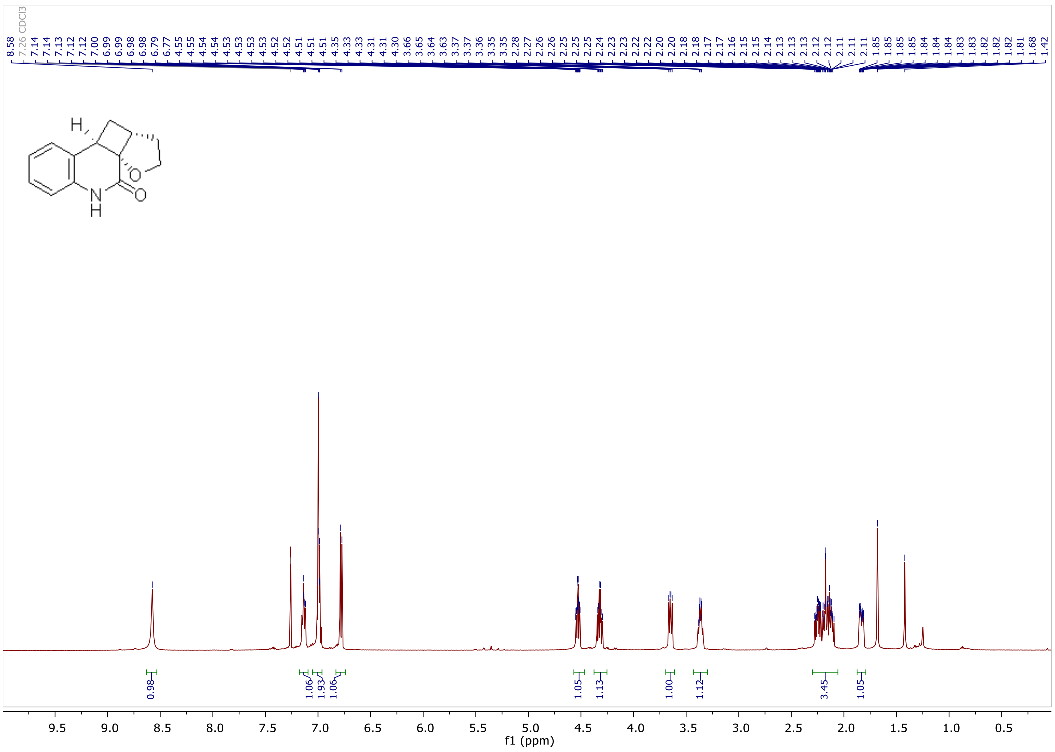

^{13}C NMR (126 MHz, $CDCl_3$) of **2a**

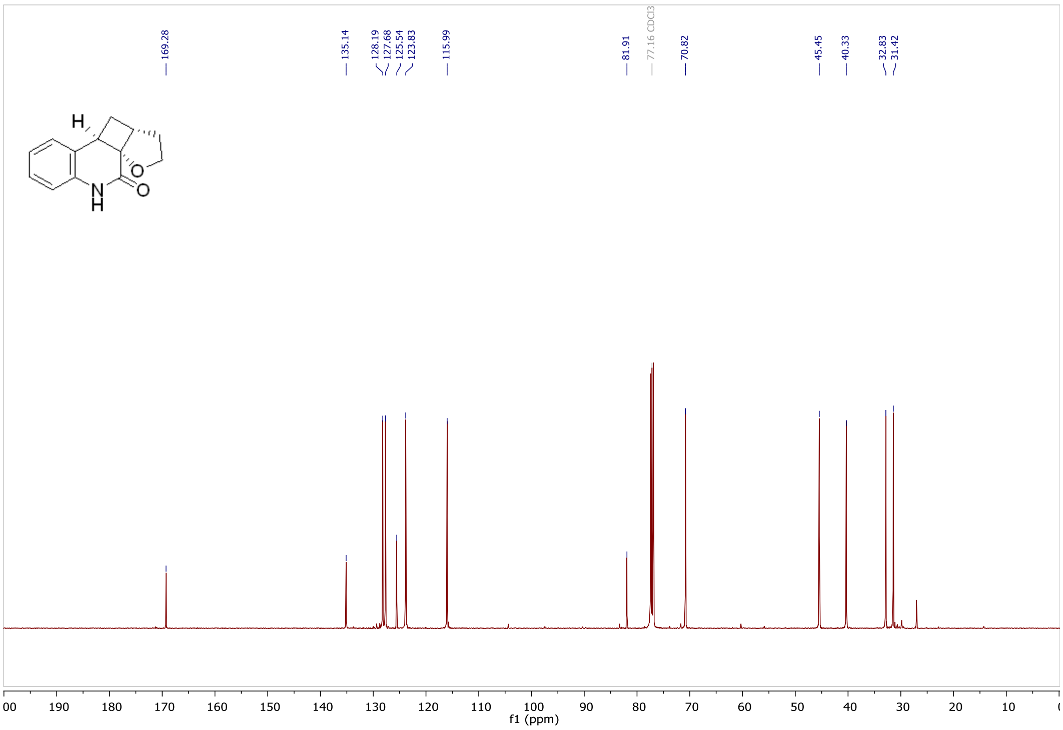

^{1}H NMR (400 MHz, $CDCl_3$) of **2b**

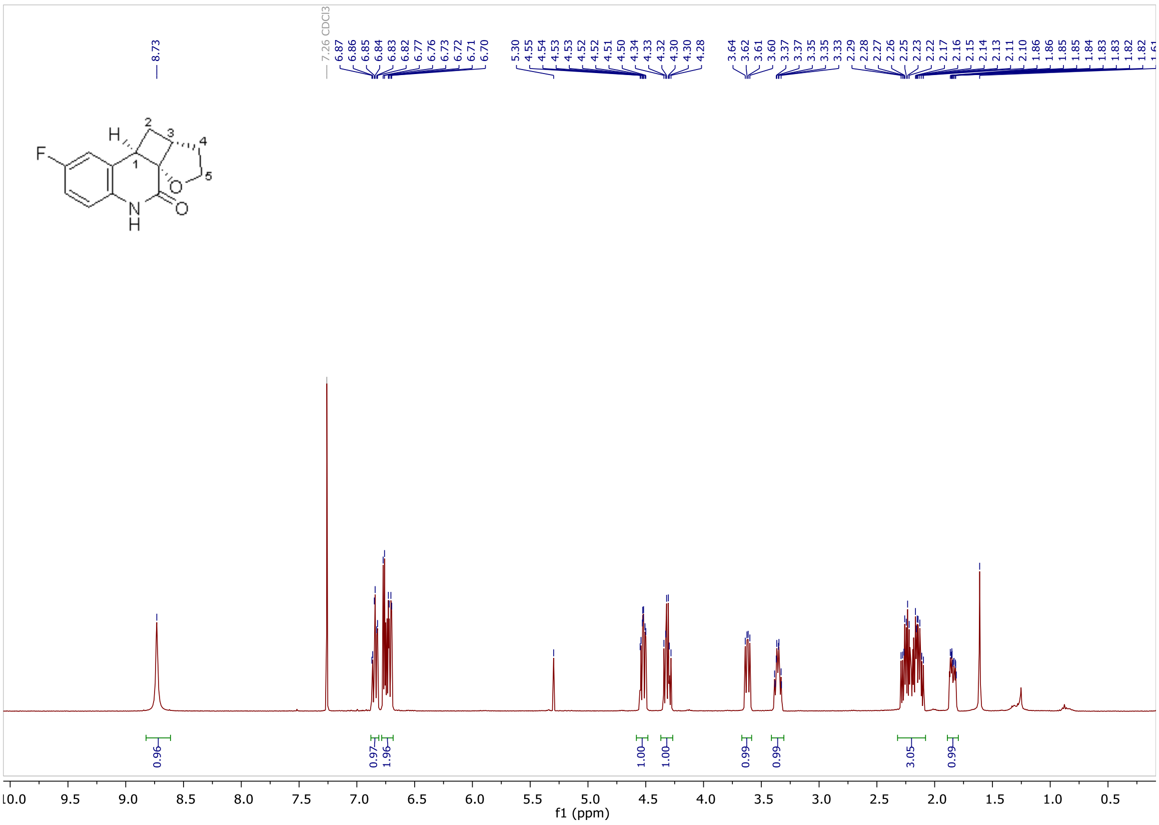

^{13}C NMR (126 MHz, $CDCl_3$) of **2b**

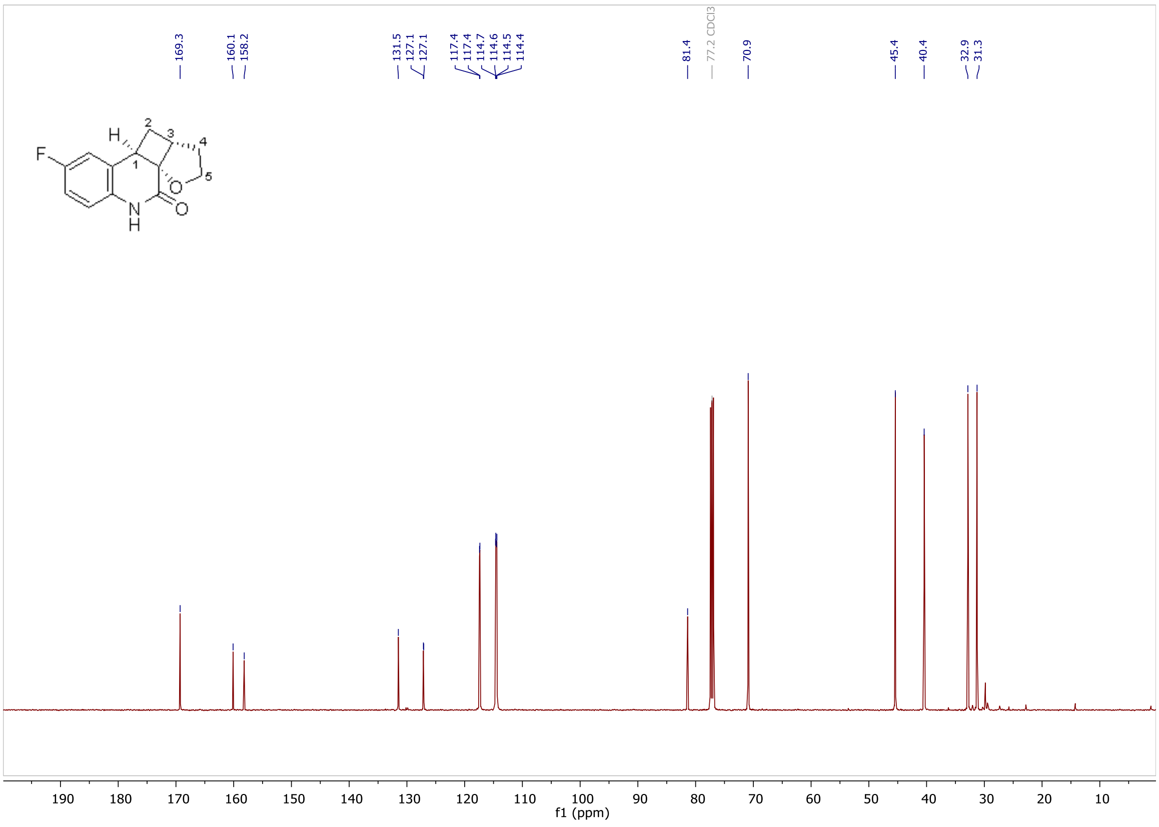

^{19}F NMR (377 MHz, $CDCl_3$) of **2b**

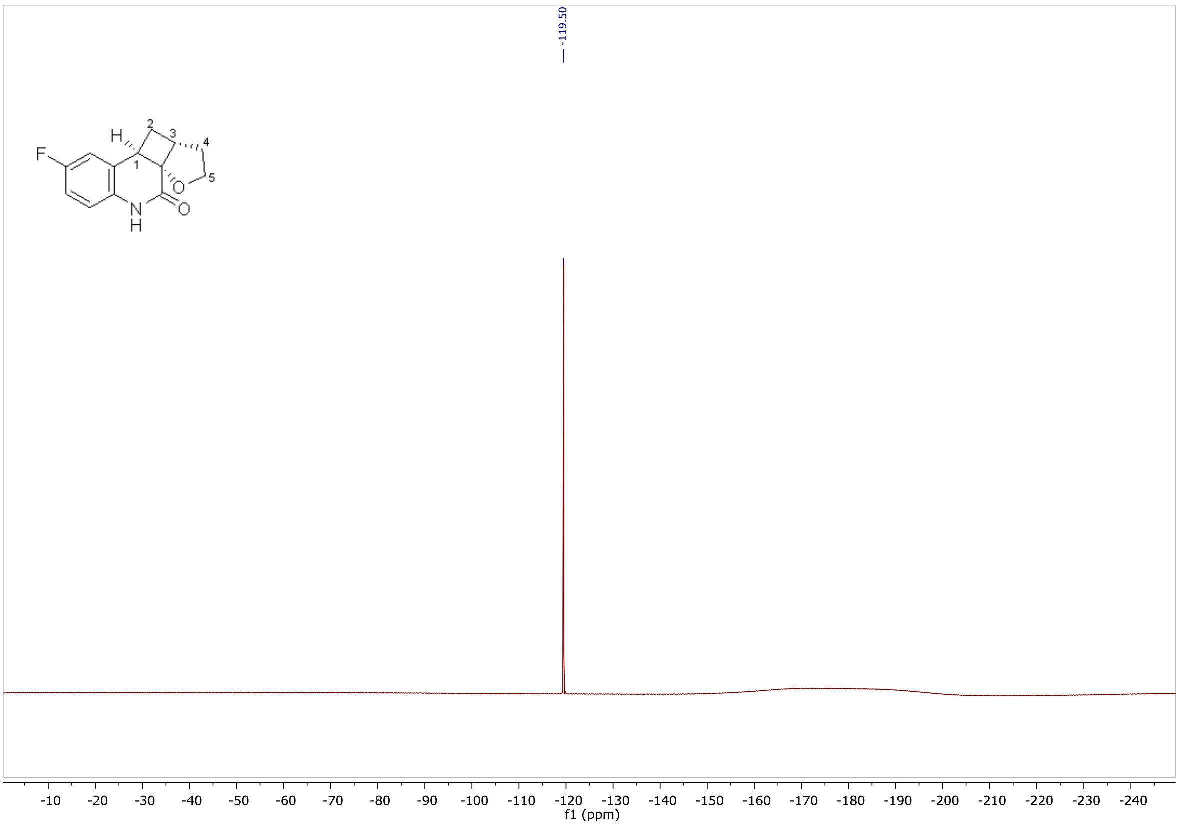

^{1}H NMR (400 MHz, $CDCl_3$) of **2c**

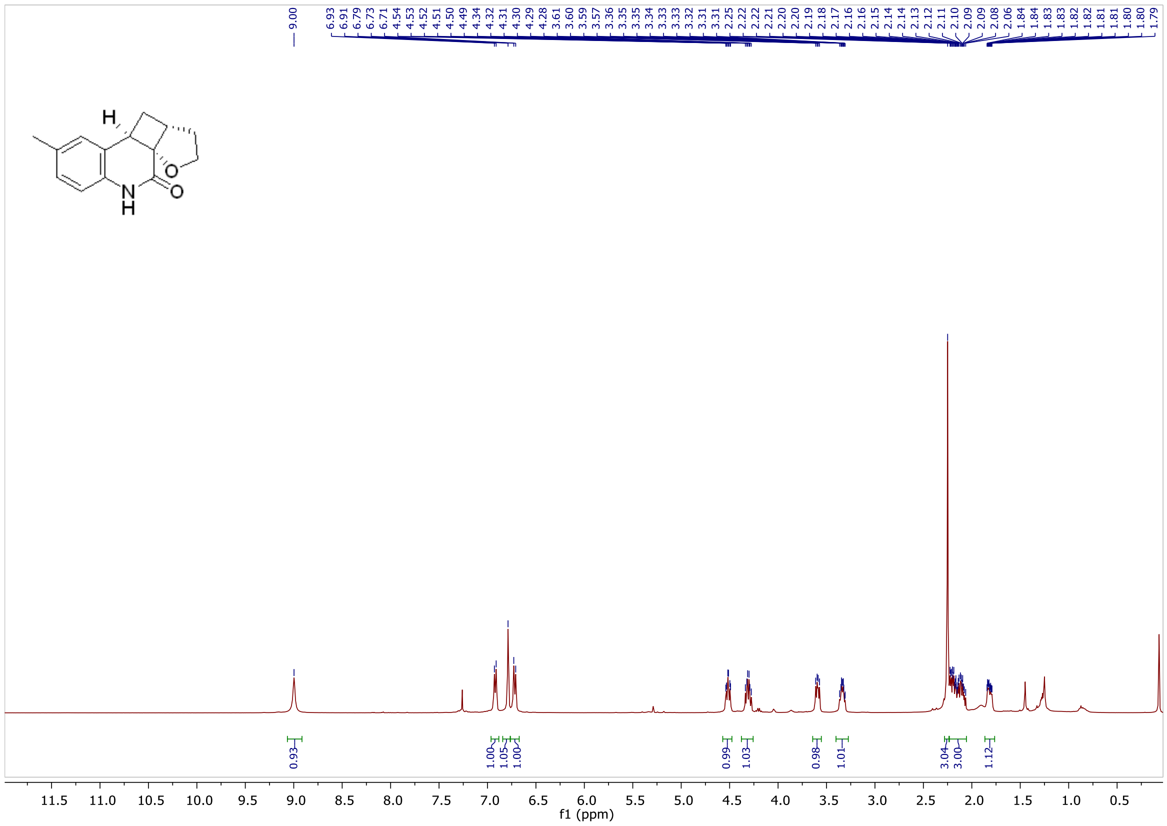

^{13}C NMR (151 MHz, $CDCl_3$) of **2c**

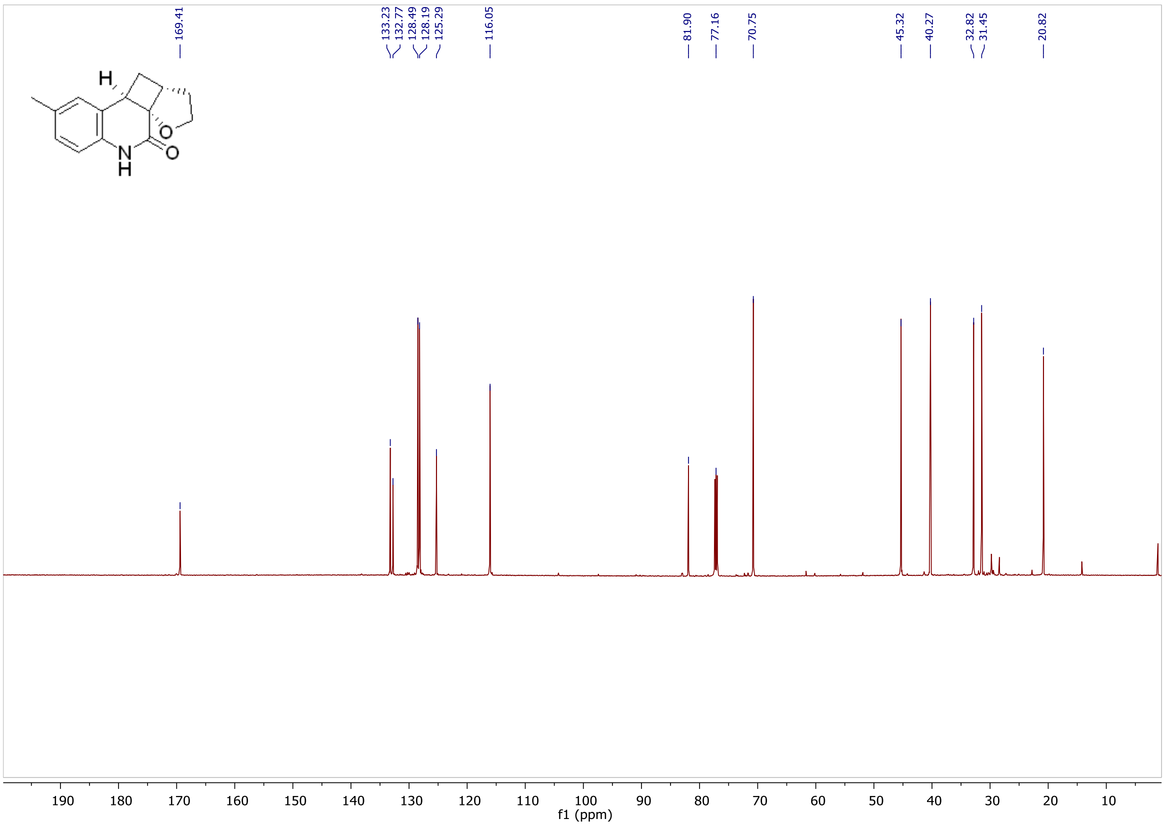

^{1}H NMR (600 MHz, $CDCl_3$) of **2d**

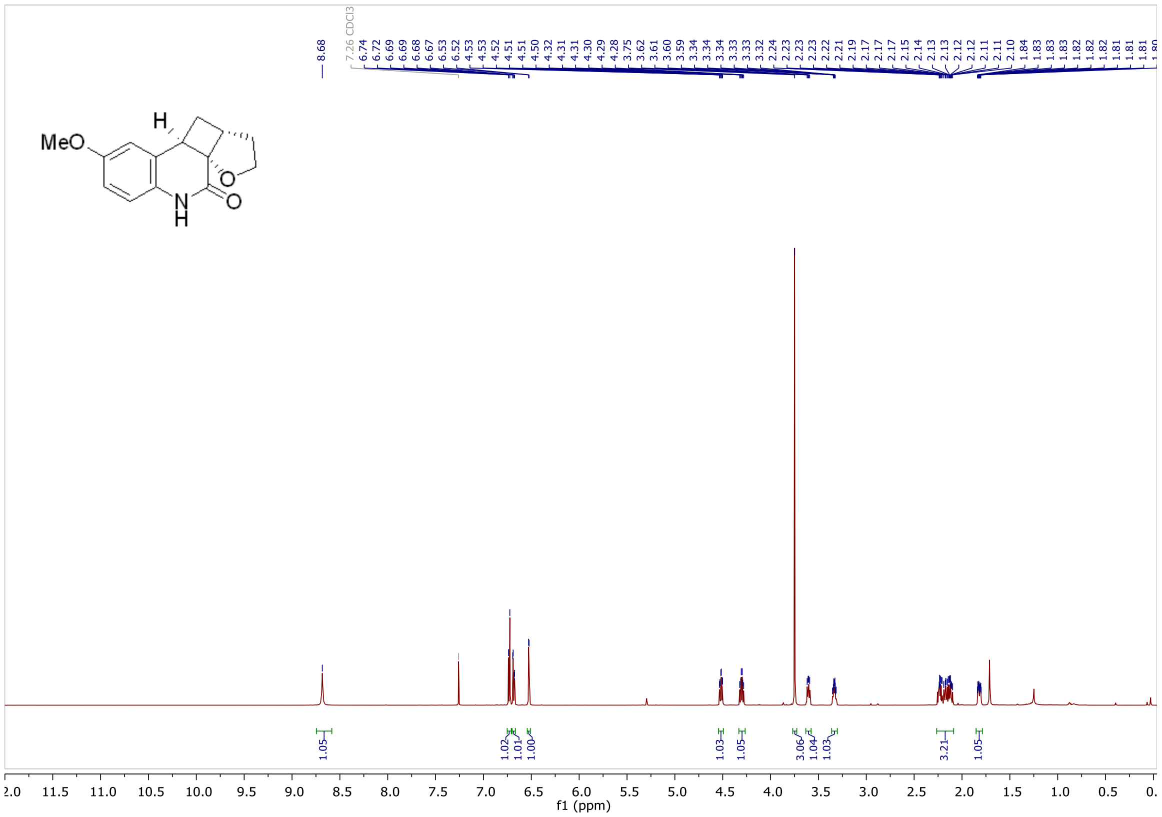

^{13}C NMR (151 MHz, $CDCl_3$) of **2d**

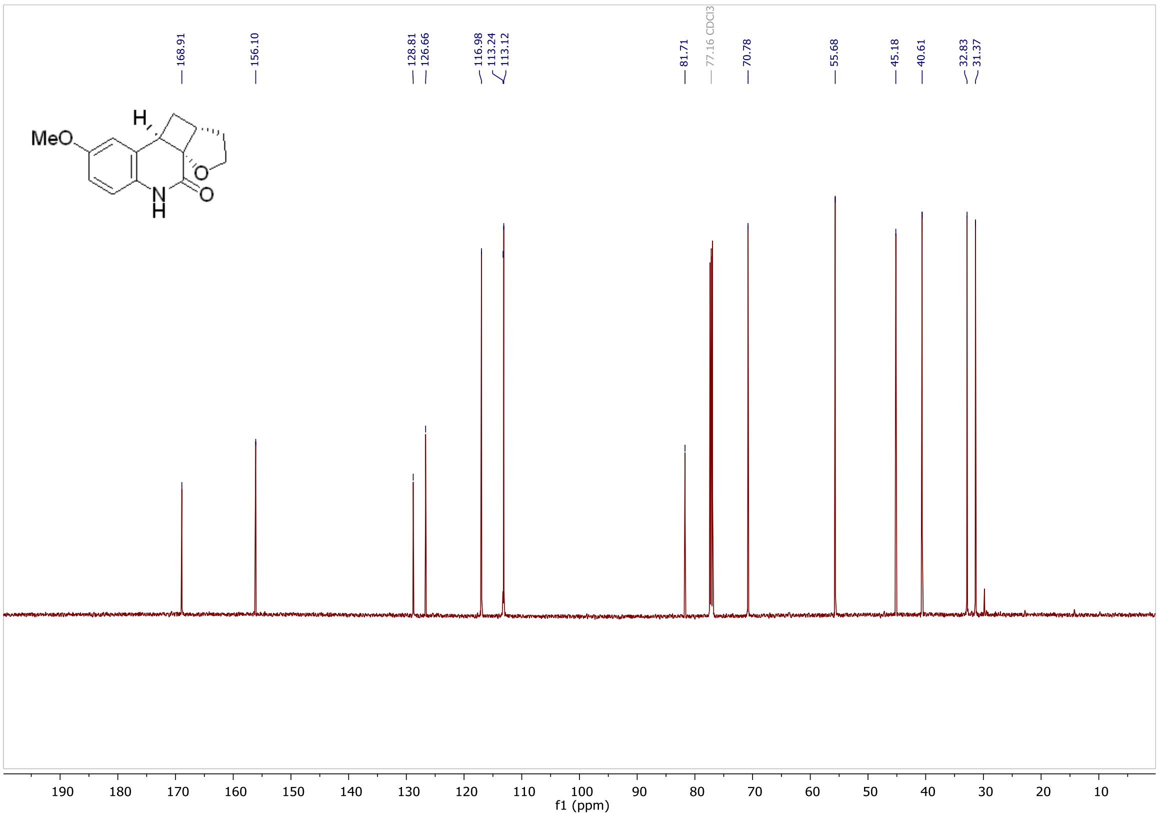

^{1}H NMR (400 MHz, $CDCl_3$) of **2e**

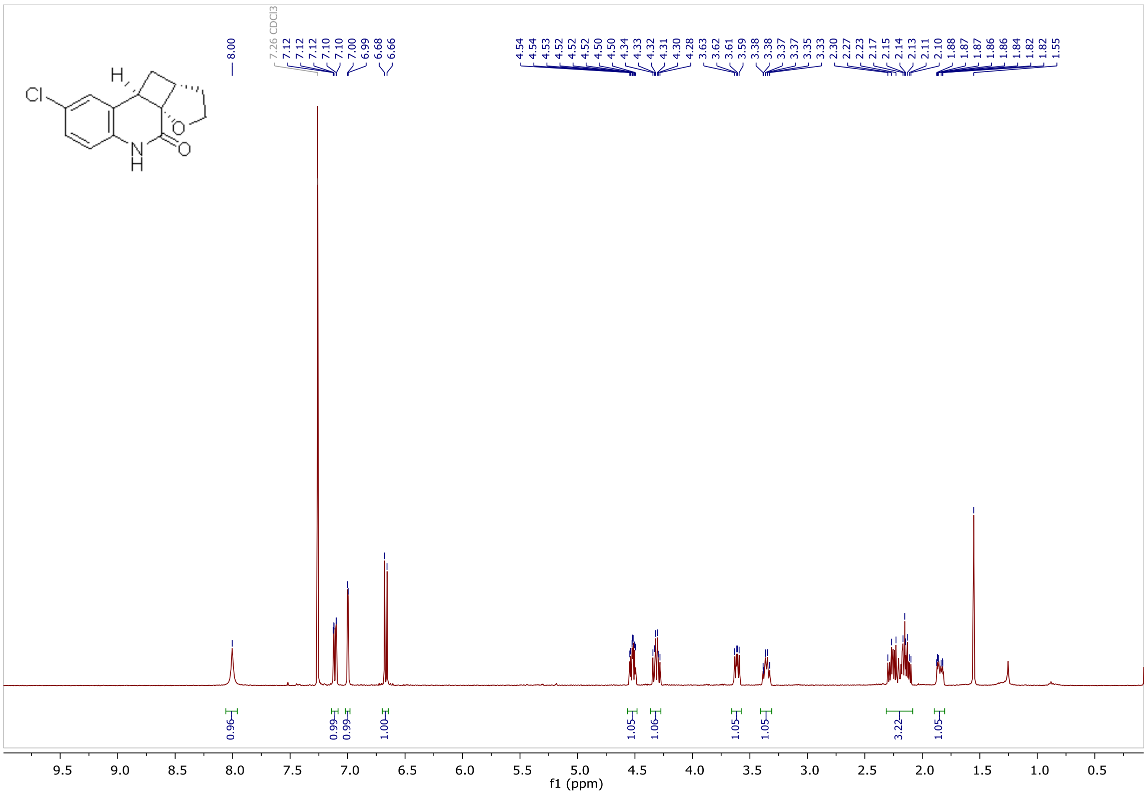

^{13}C NMR (126 MHz, MeOD) of **2e**

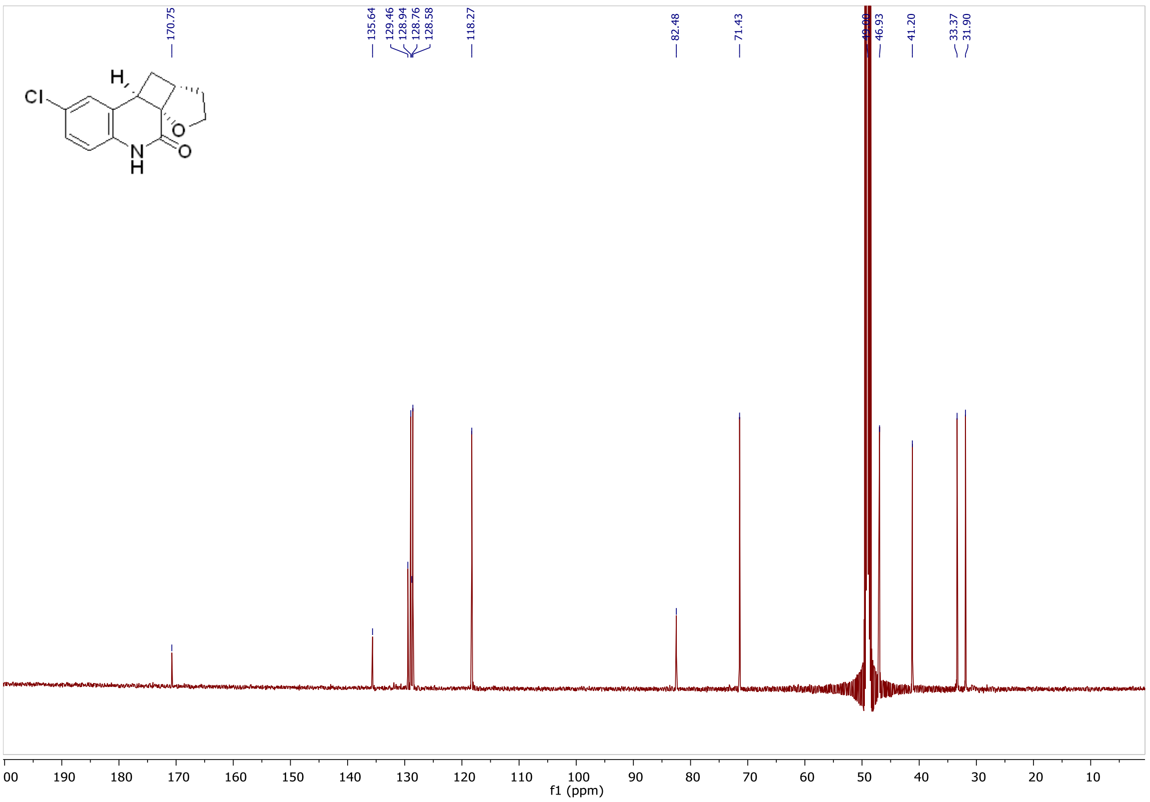

^{1}H NMR (400 MHz, $CDCl_3$) of **2f**

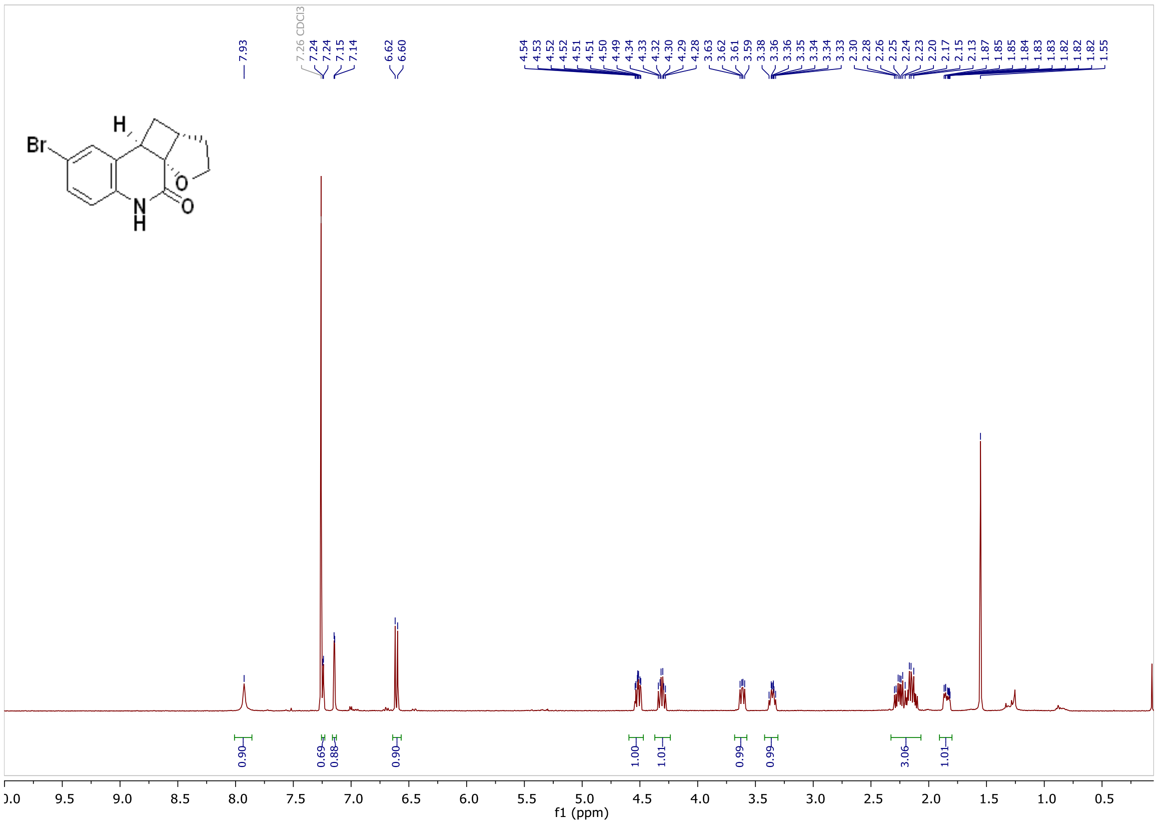

^{13}C NMR (126 MHz, $CDCl_3$) of **2f**

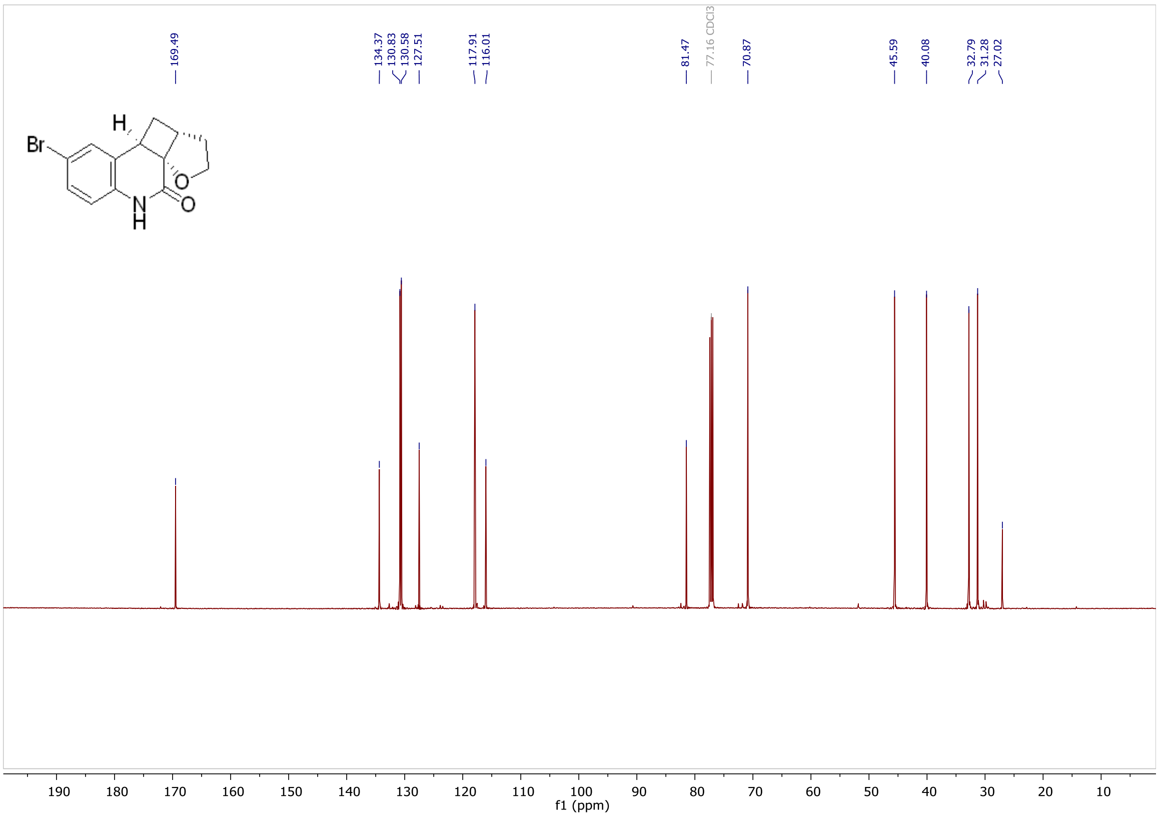

^1H NMR (400 MHz, $CDCl_3$) of **2g**

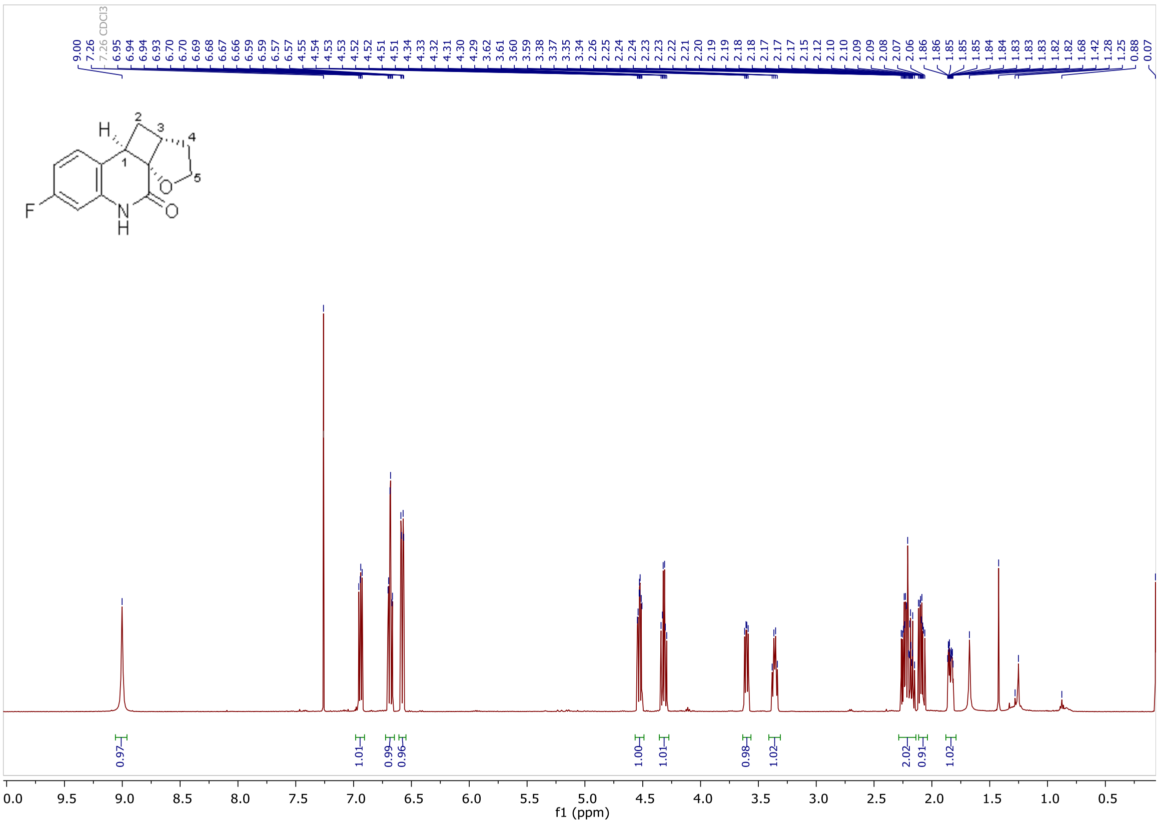

^{13}C NMR (126 MHz, $CDCl_3$) of **2g**

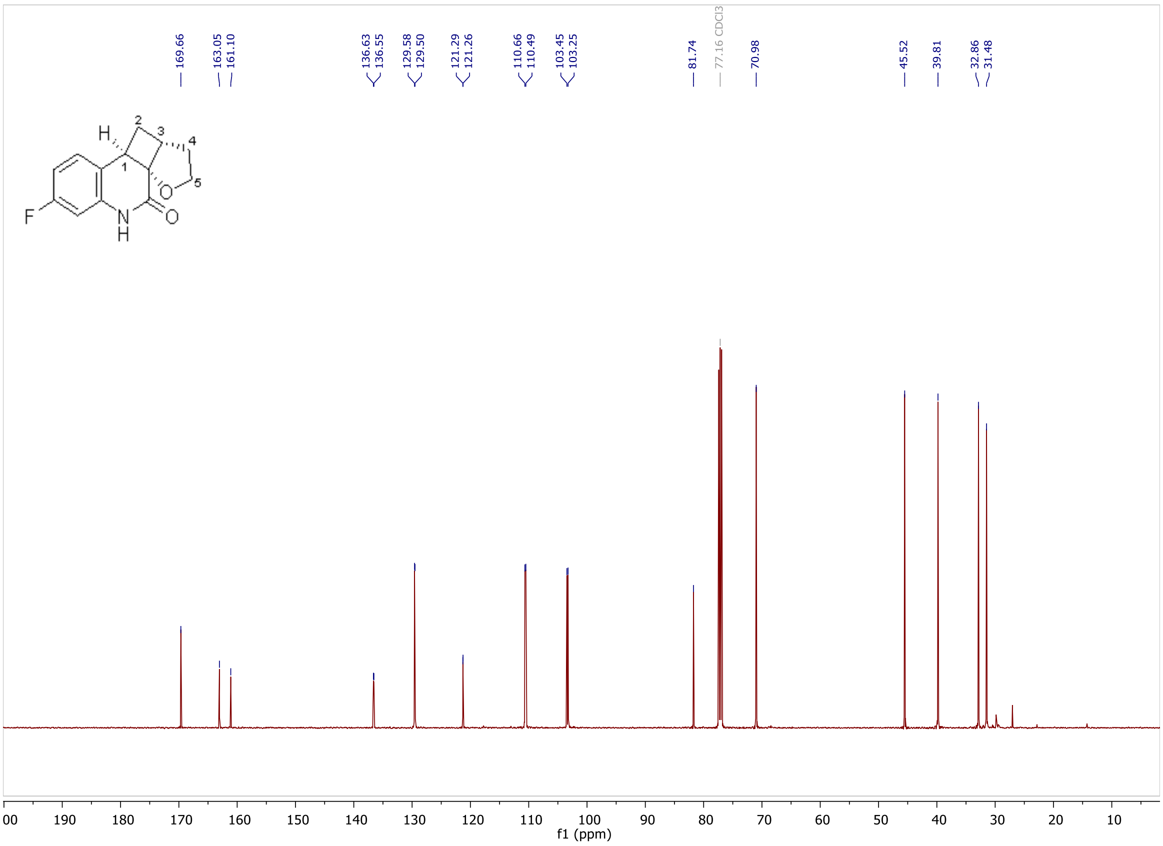

^{19}F NMR (376 MHz, $CDCl_3$) of **2g**

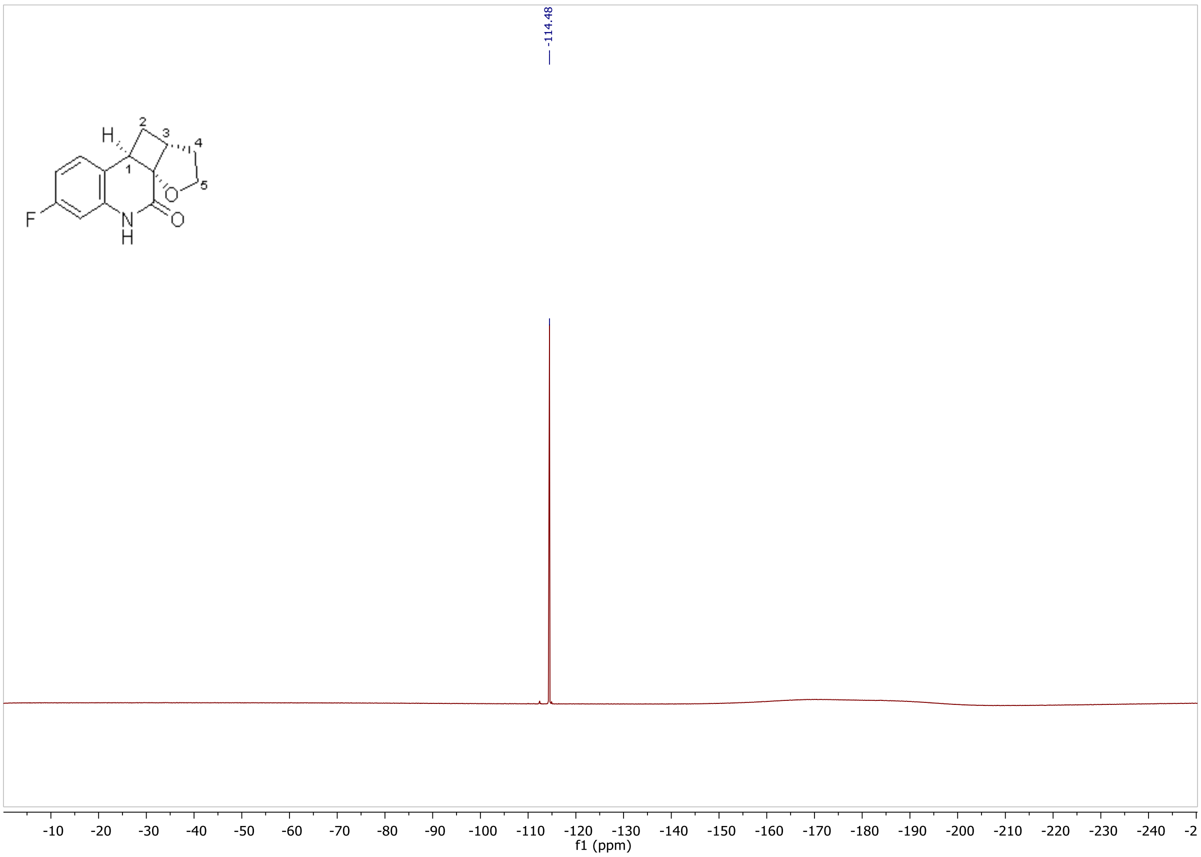

^{1}H NMR (400 MHz, CDCl₃) of **2h**

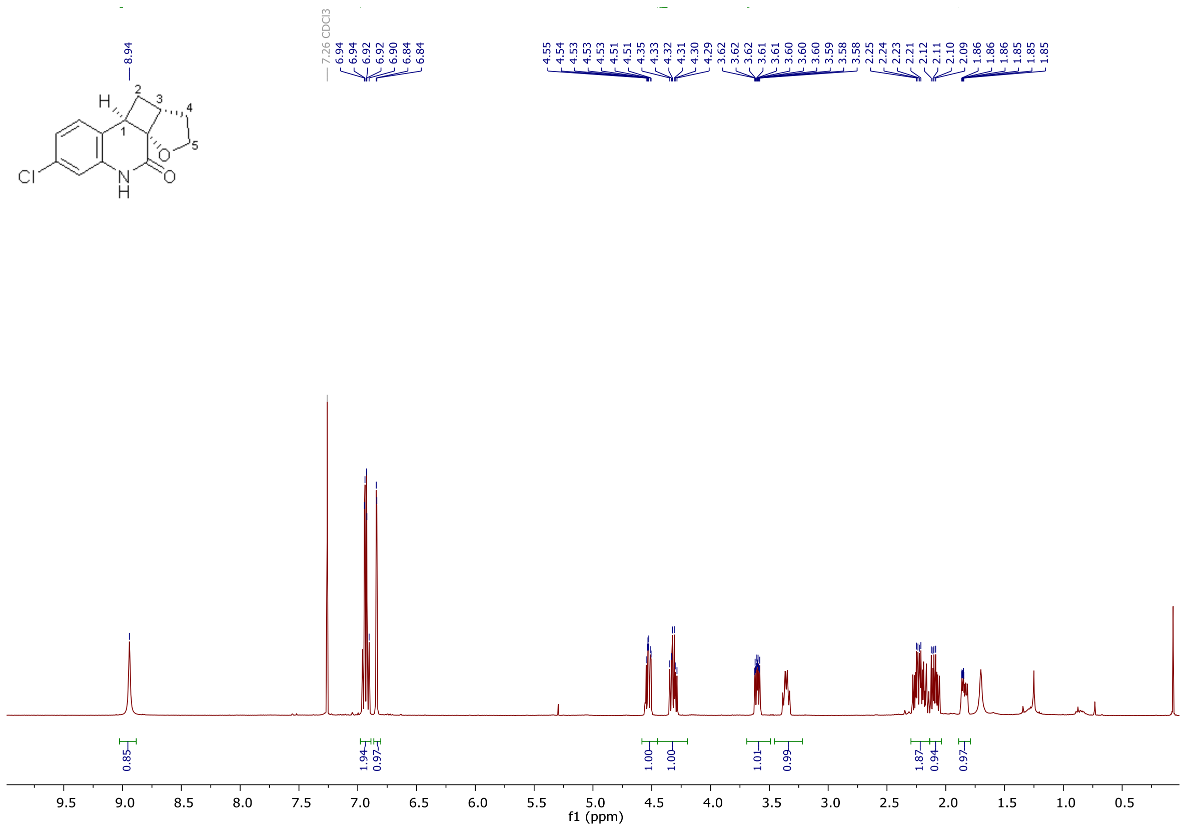

^{13}C NMR (101 MHz, CDCl₃) of **2h**

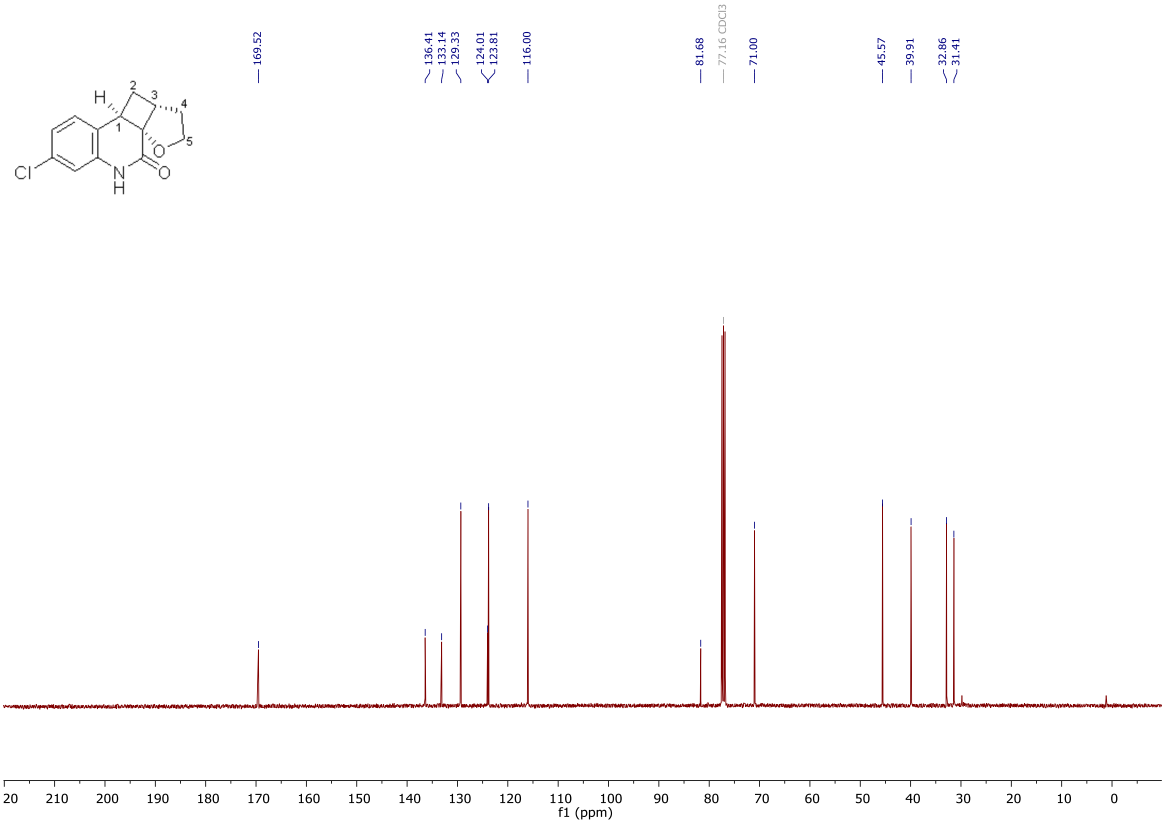

^{1}H NMR (400 MHz, $CDCl_3$) of **2i**

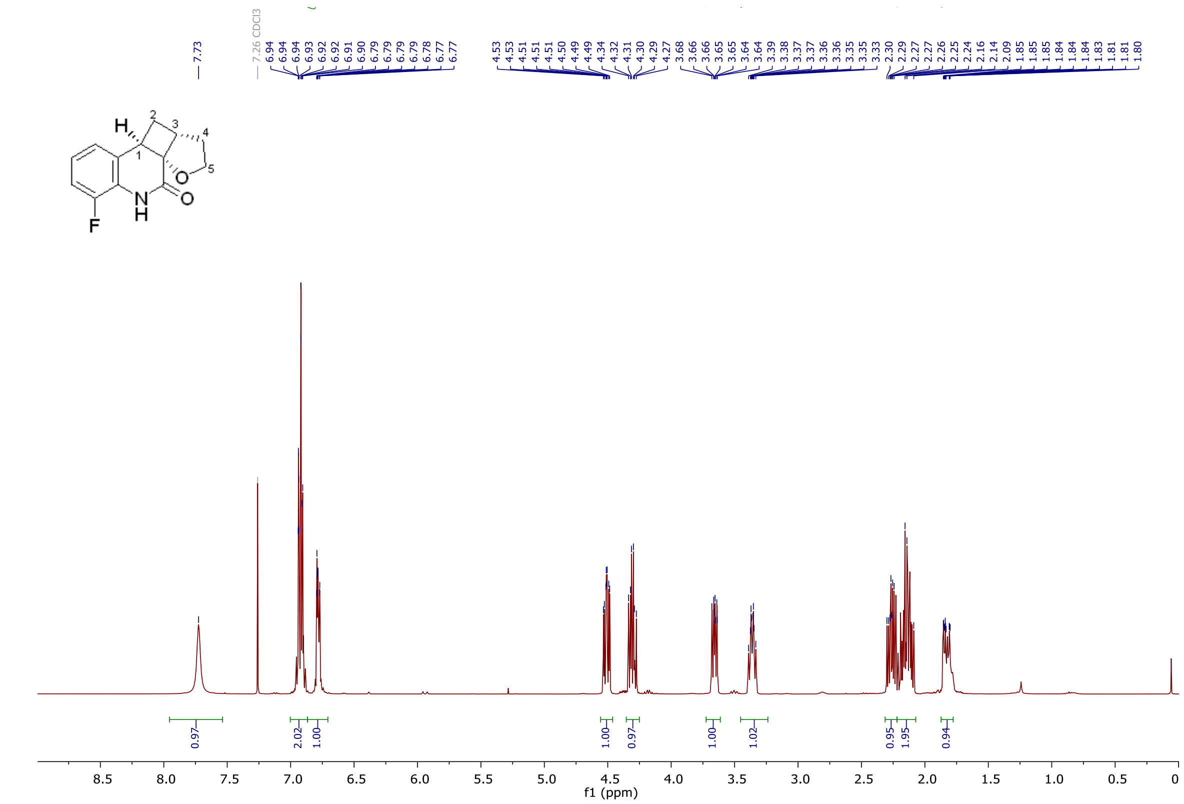

^{13}C NMR (101 MHz, $CDCl_3$) of **2i**

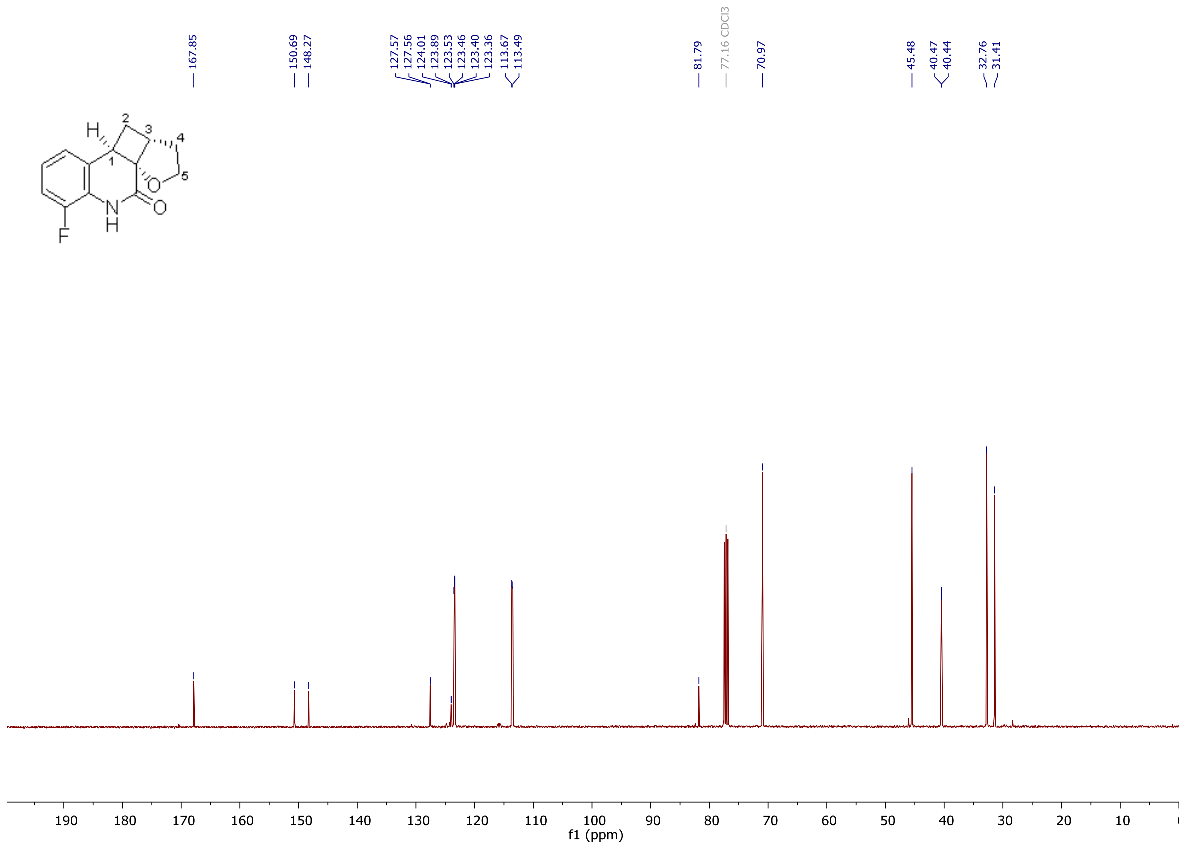

^{19}F NMR (376 MHz, $CDCl_3$) of **2i**

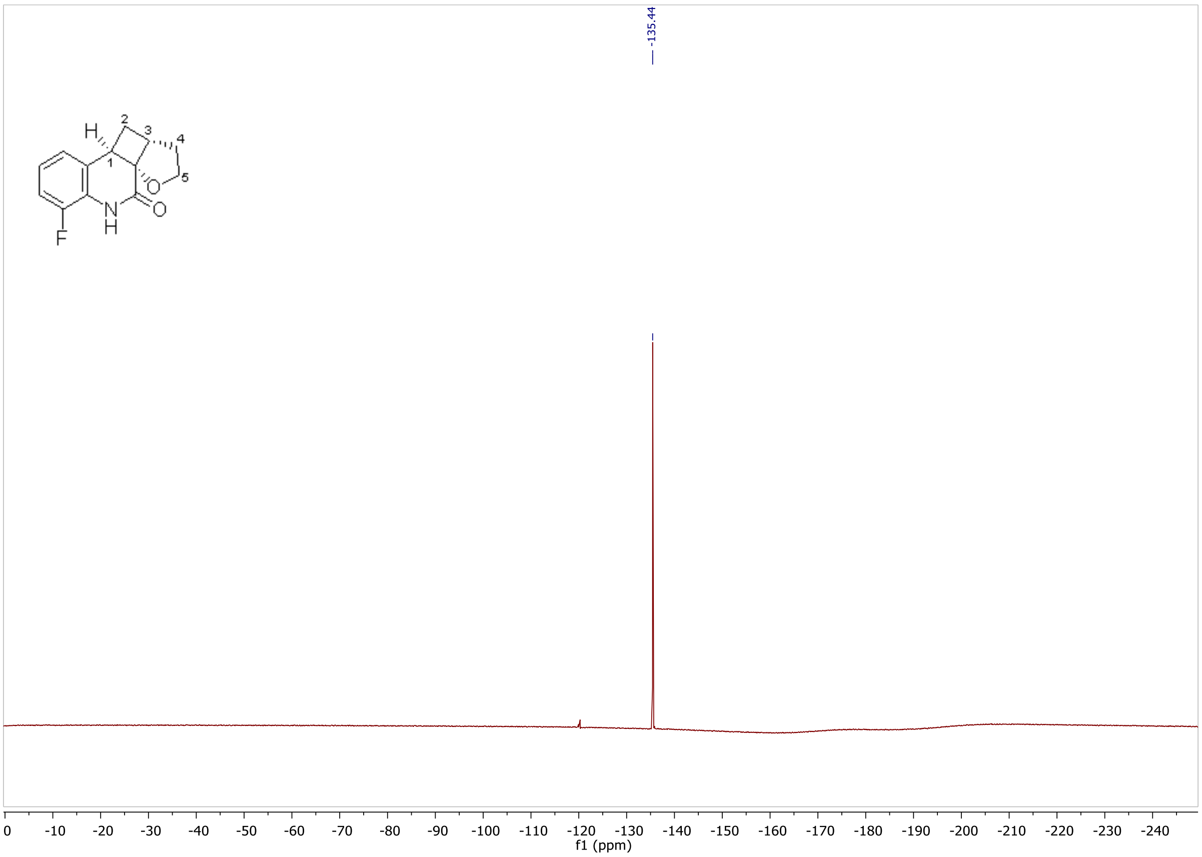

^{1}H NMR (600 MHz, $CDCl_3$) of **2j**

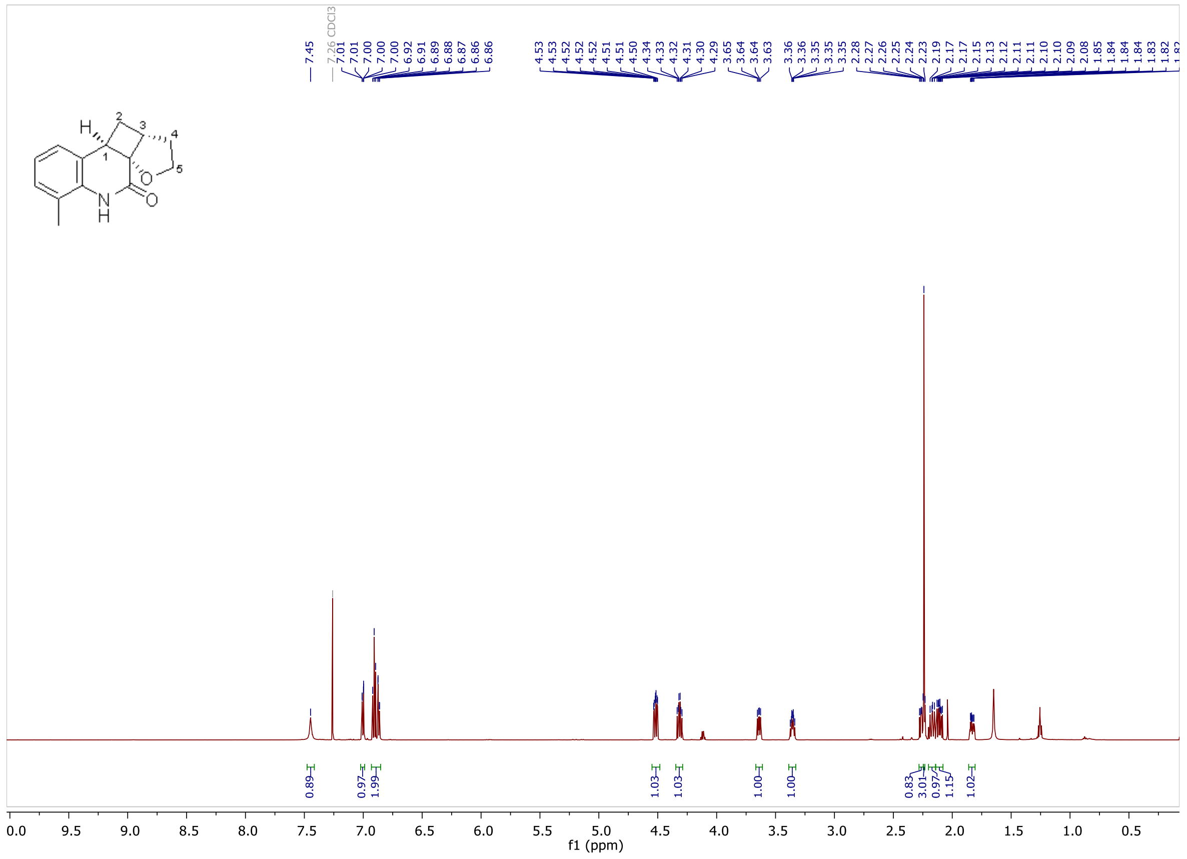

^{13}C NMR (151 MHz, CDCl$_3$) of **2j**

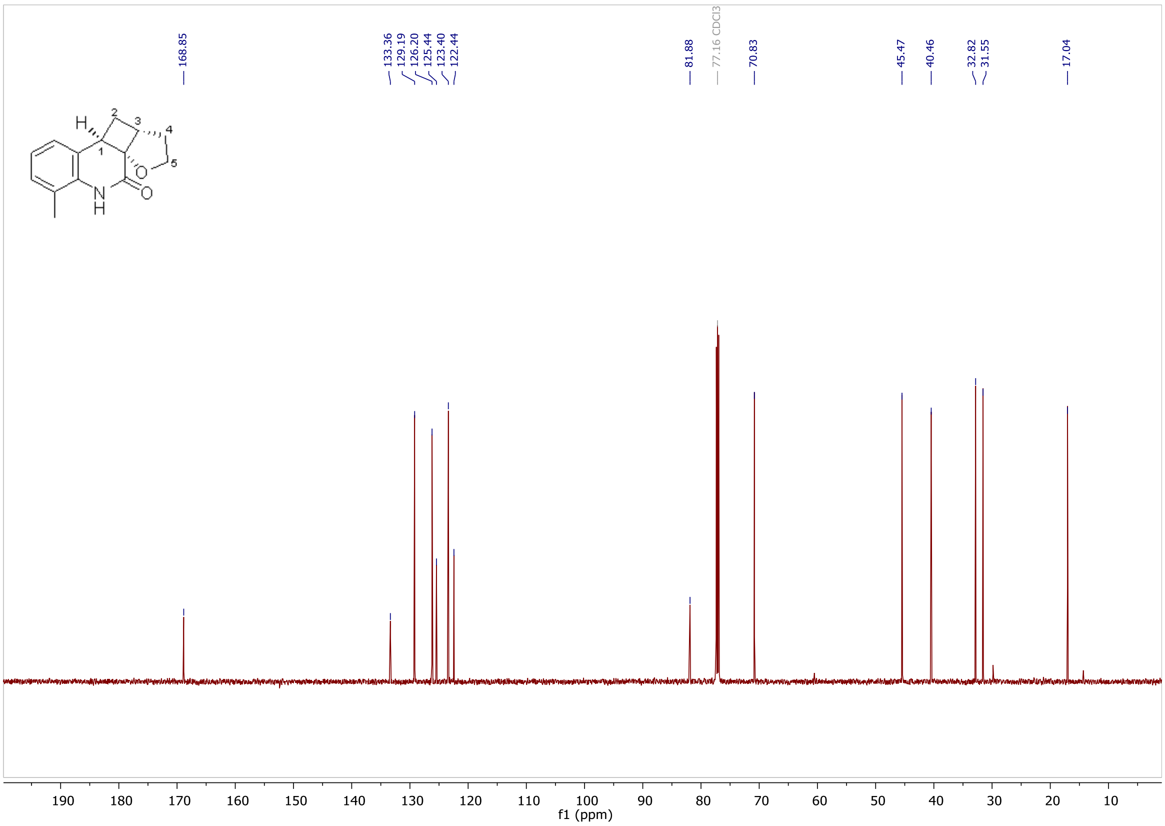

^{1}H NMR (600 MHz, CDCl$_3$) of **2k**

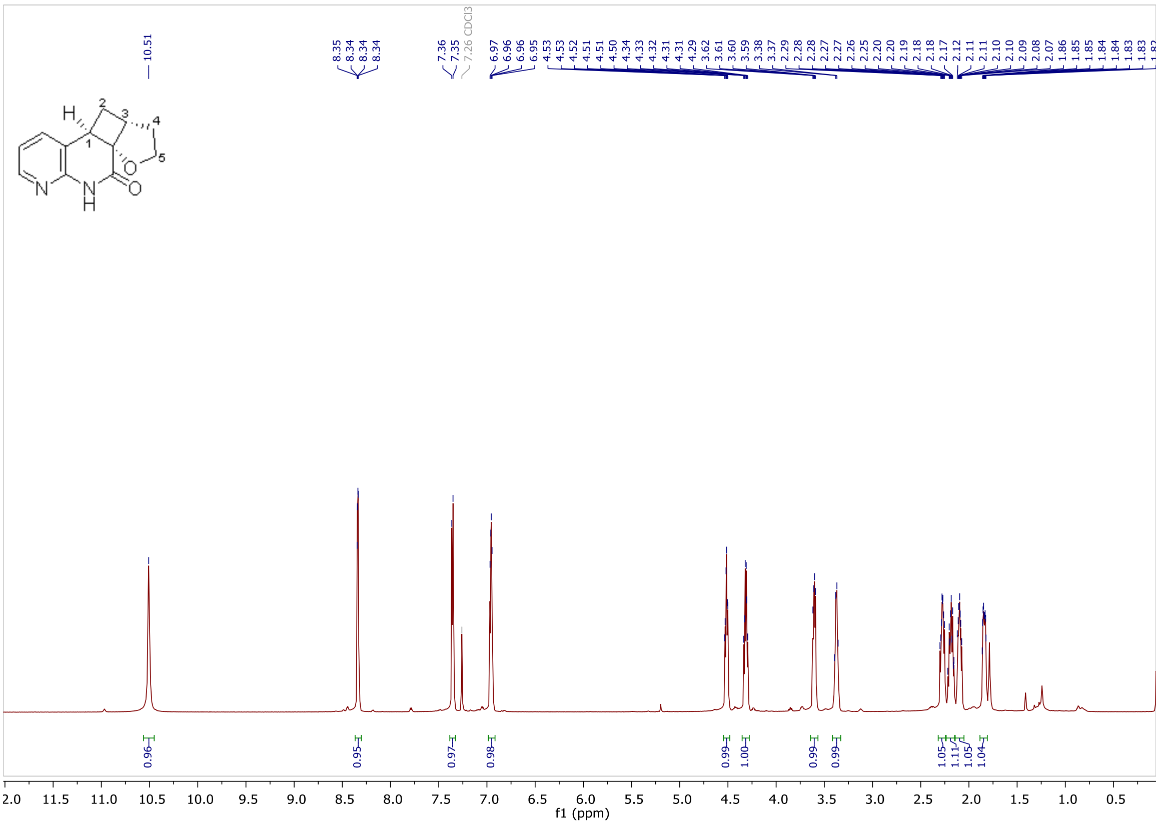

^{13}C NMR (151 MHz, $CDCl_3$) of **2k**

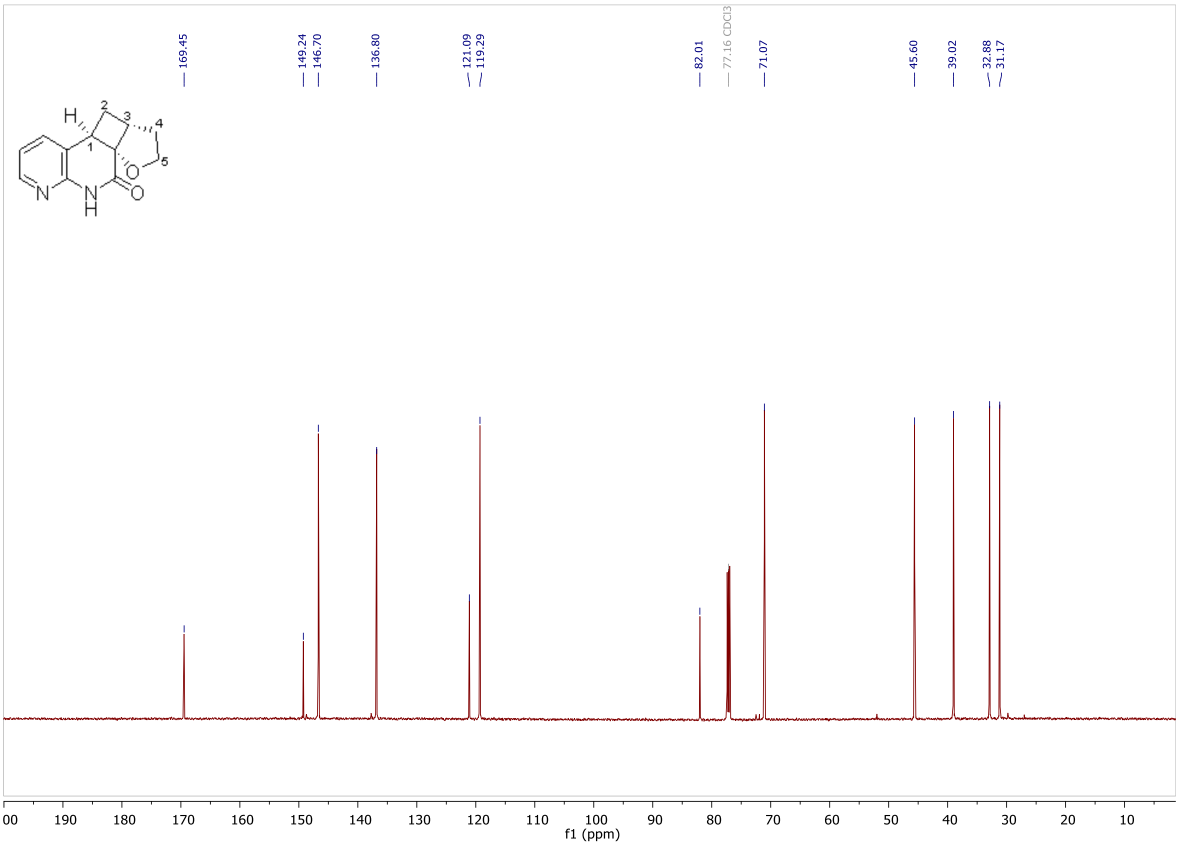

^{1}H NMR (400 MHz, $CDCl_3$) of **2l**

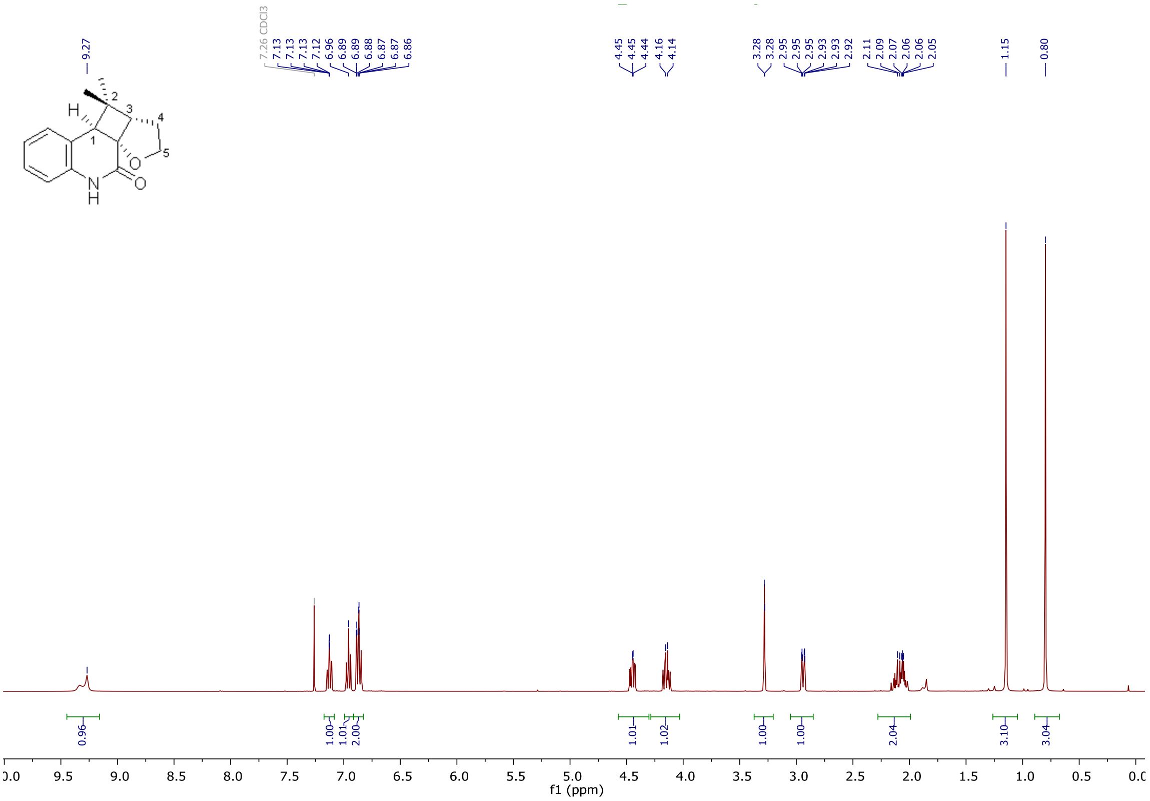

^{13}C NMR (151 MHz, $CDCl_3$) of **2l**

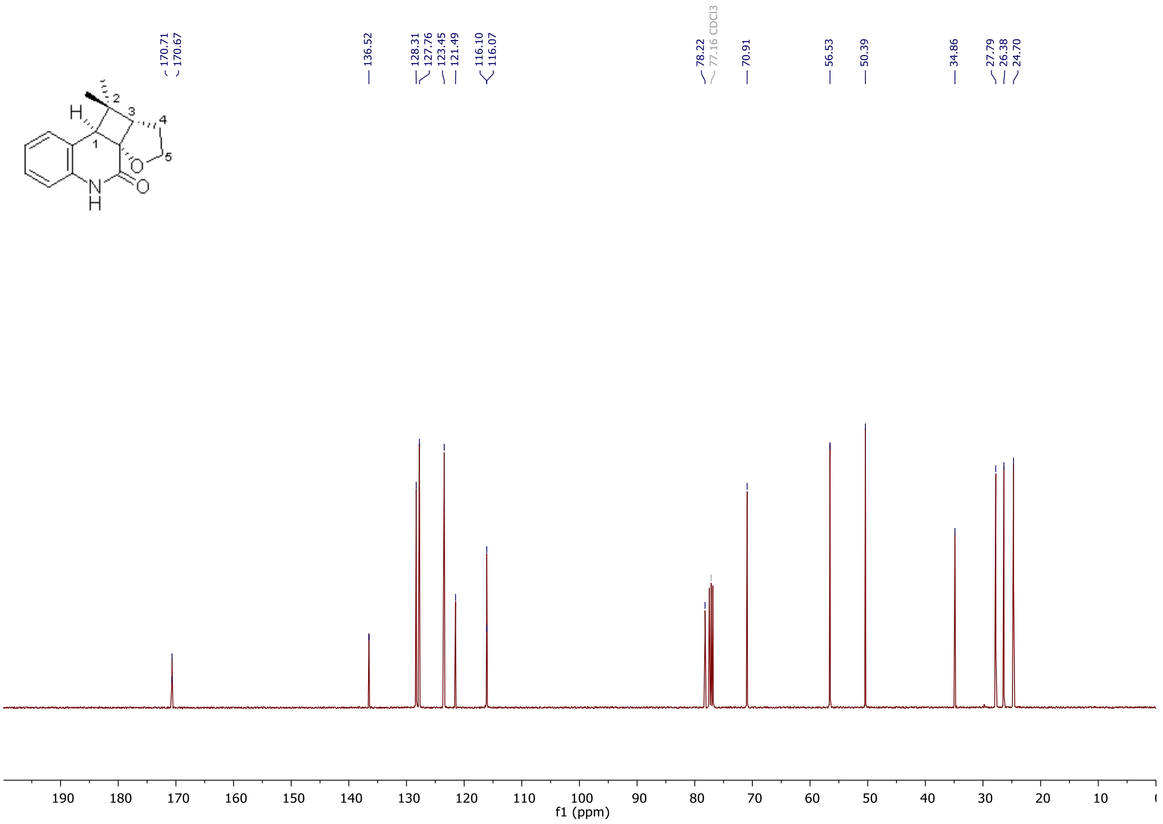

^{1}H NMR (400 MHz, $CDCl_3$) of **2m**

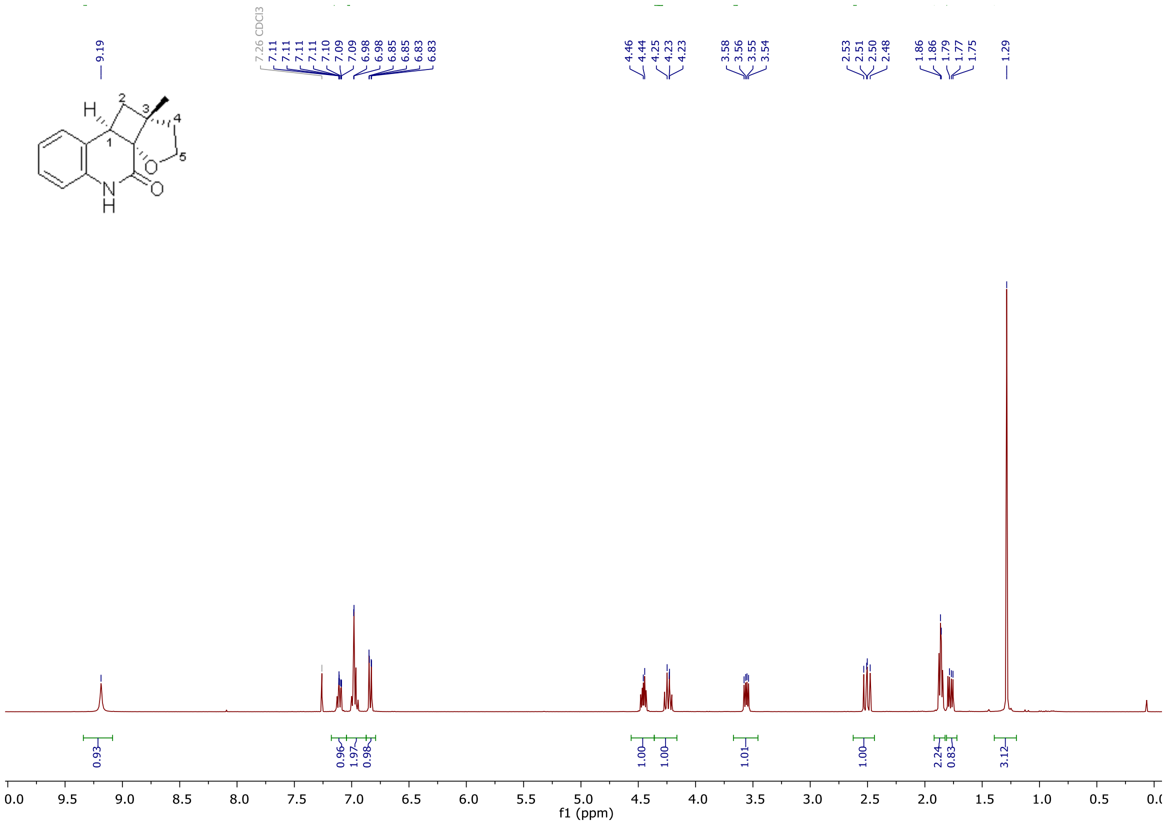

^{13}C NMR (151 MHz, CDCl3) of **2m**

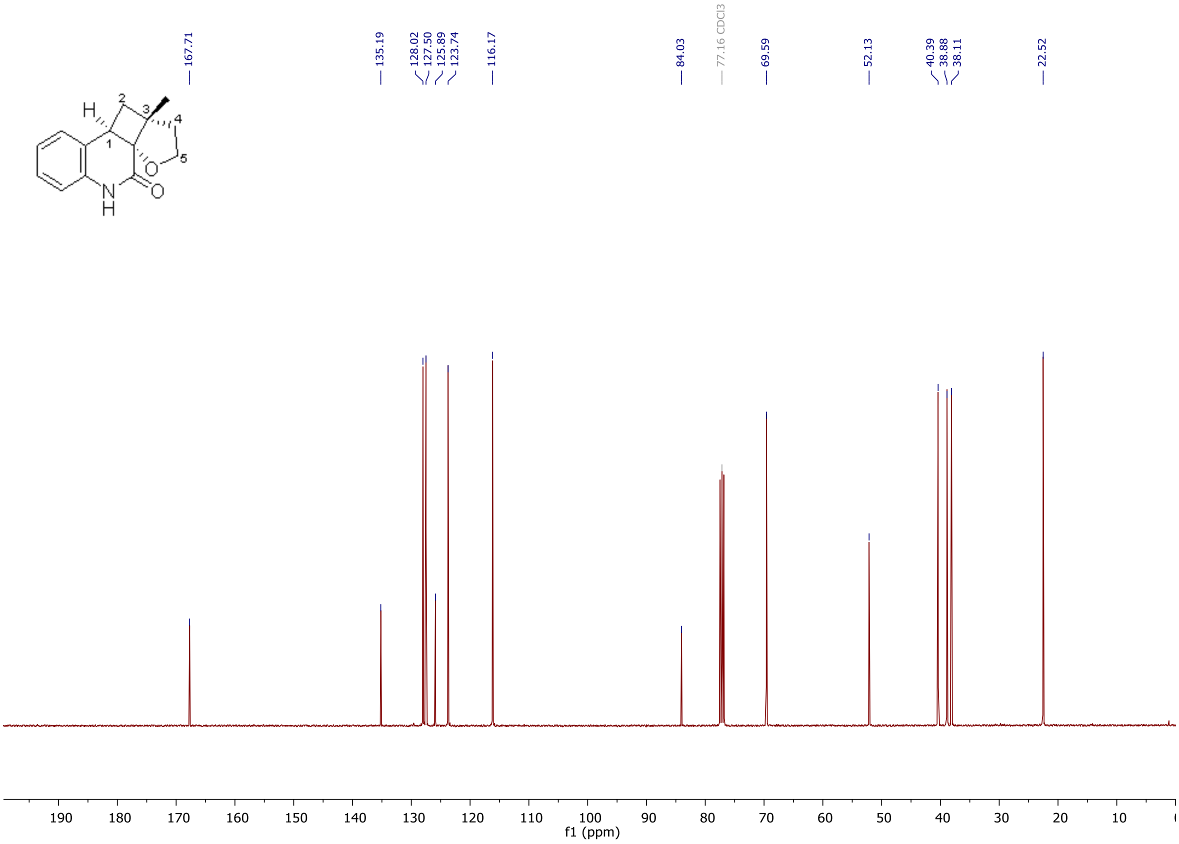

^{1}H NMR (500 MHz, CDCl3) of **2n**

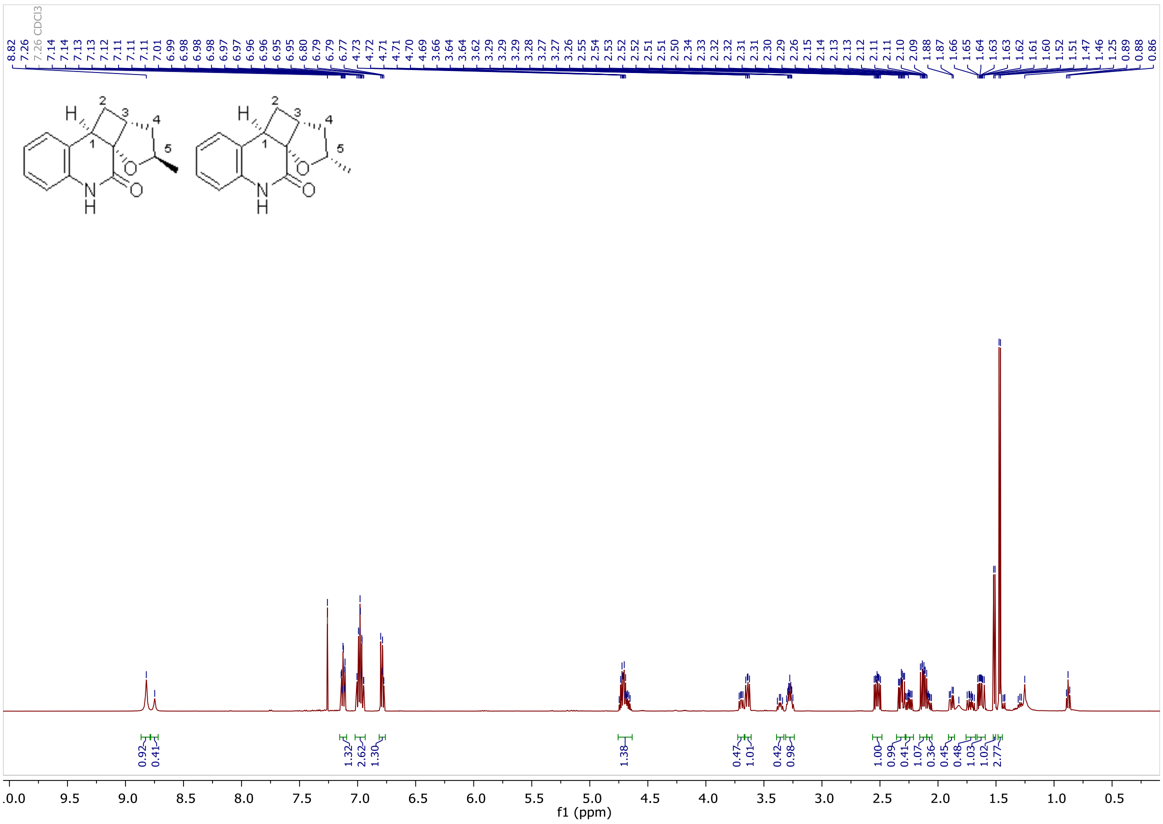

^{13}C NMR (101 MHz, $CDCl_3$) of **2n**

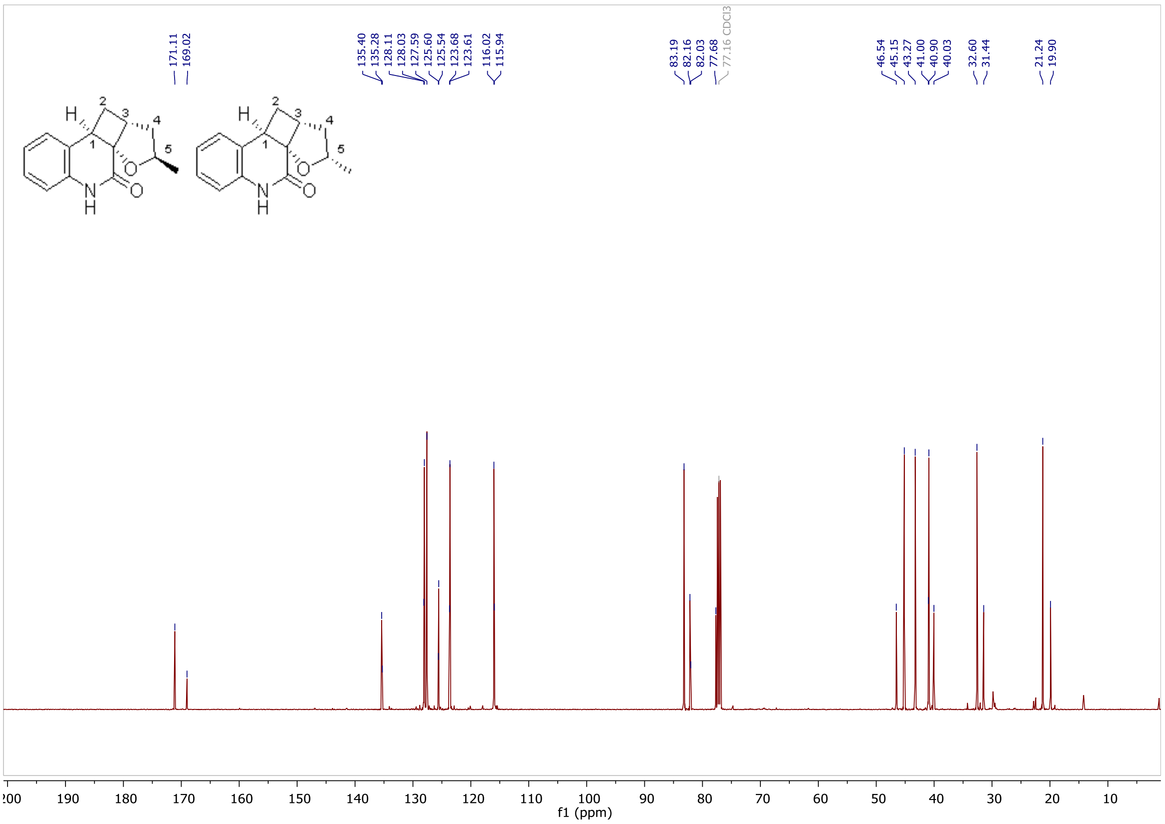

^{1}H NMR (600 MHz, $CDCl_3$) of **2o**

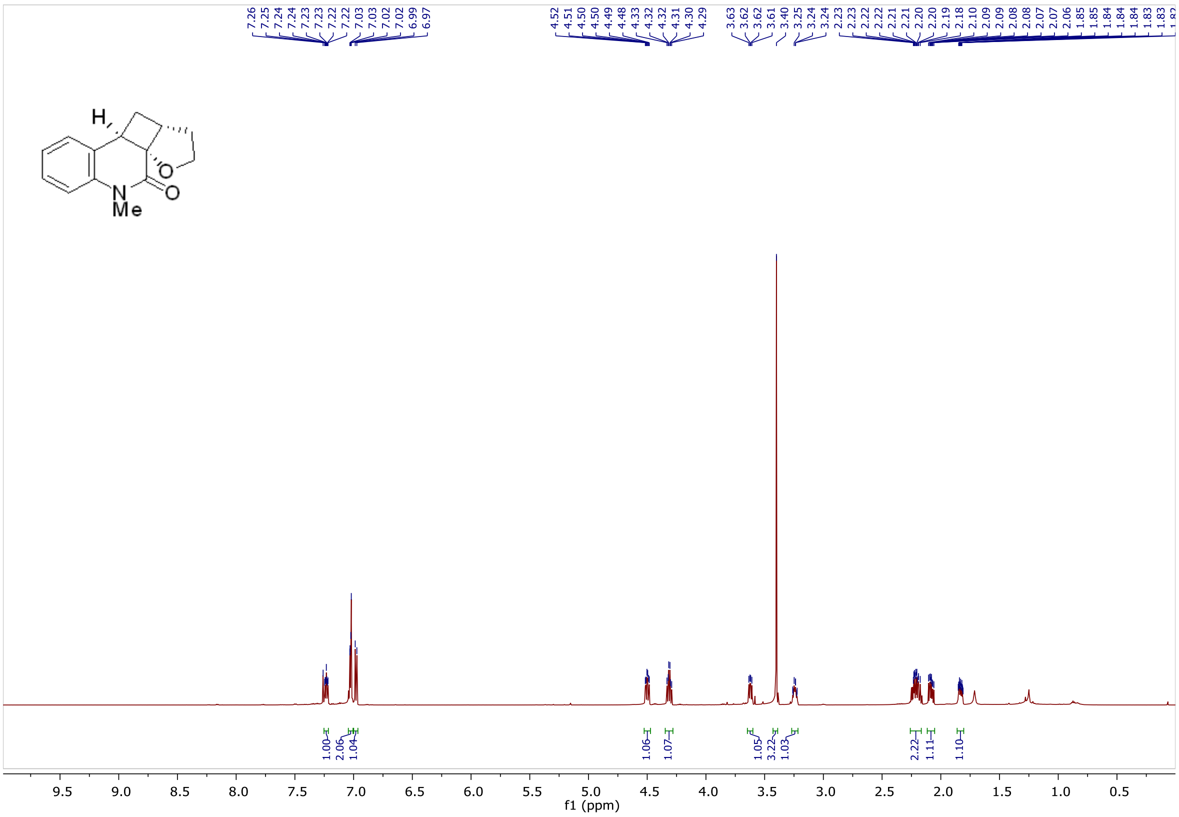

^{13}C NMR (151 MHz, $CDCl_3$) of **2o**

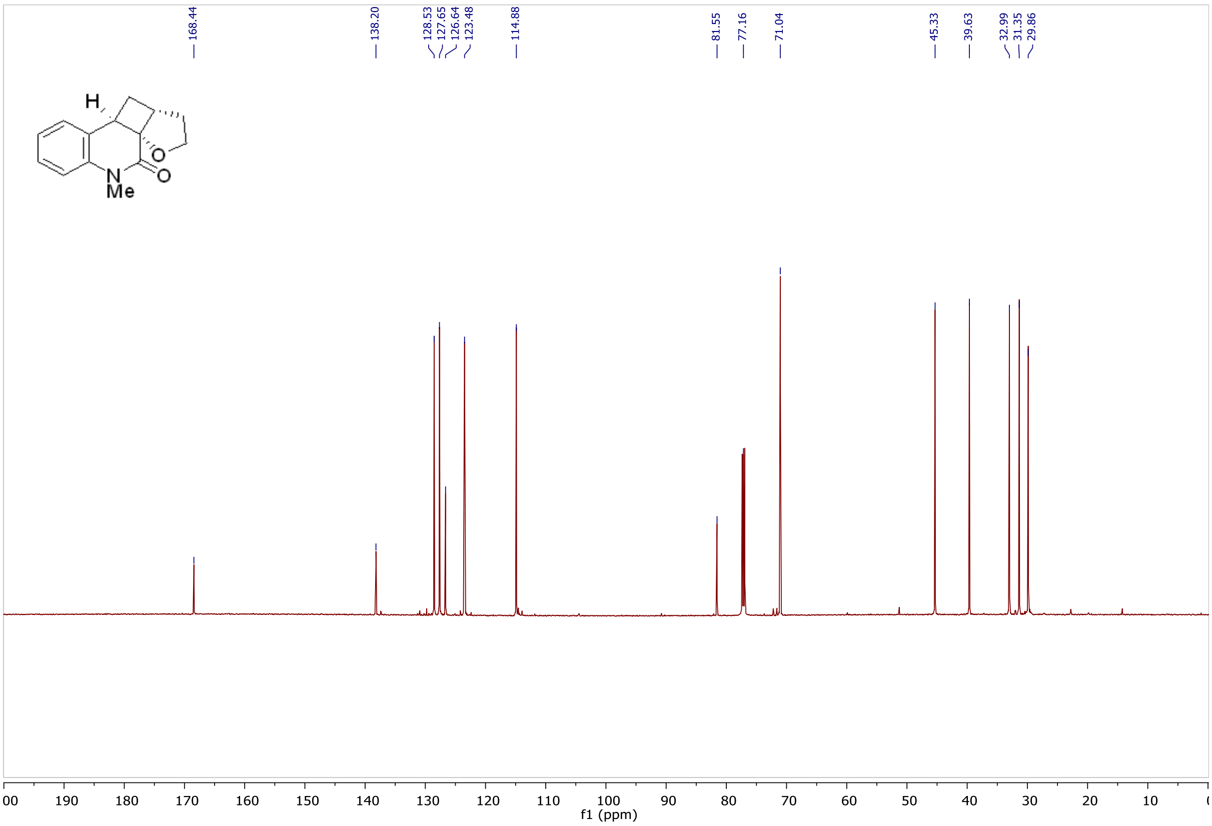

^{1}H NMR (600 MHz, $CDCl_3$) of **2p**

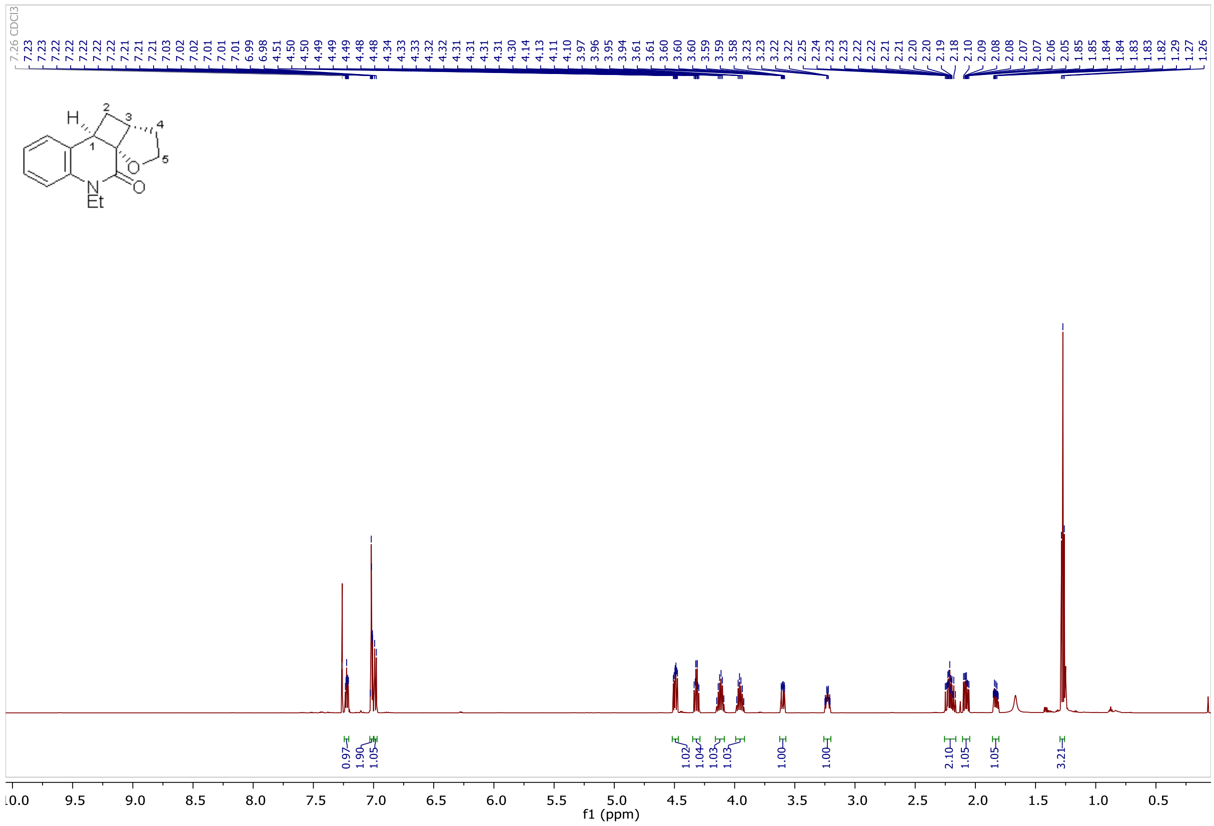

^{13}C NMR (151 MHz, $CDCl_3$) of **2p**

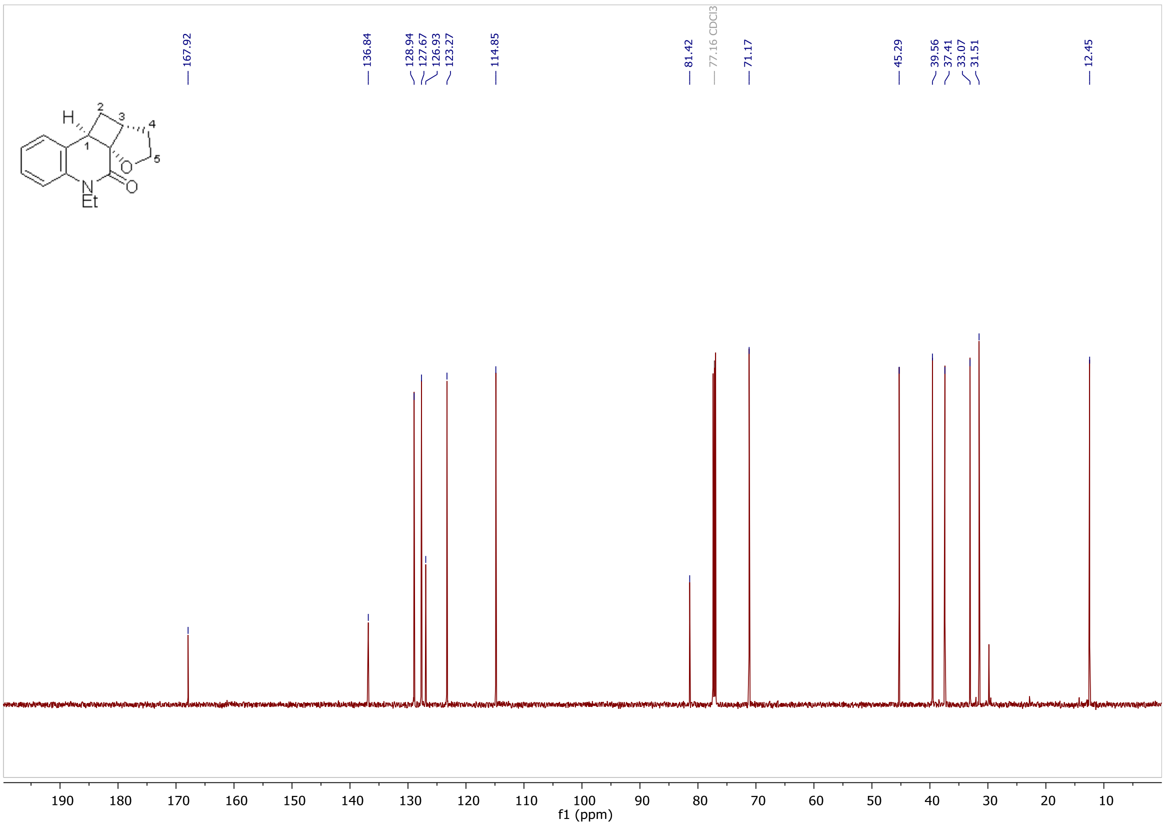

XIII. Chiral HPLC spectra of [2+2] photocycloaddition products

1. General information

HPLC traces at 260 nm were recorded for crude reaction mixtures in which both substrate and product signals were observable.

Conversion was determined using calibration curves and calculated according to the following equation:

$$\text{Conversion} = \frac{A_P}{A_P + kA_S}$$

Where A_p and A_s are the integrated peak areas of the product and substrate, respectively, and k is the relative response factor obtained from the calibration curve.

Enantiomeric ratios (e.r.) were determined by integration of the corresponding enantiomeric peaks.

2. (D)-DNA serie

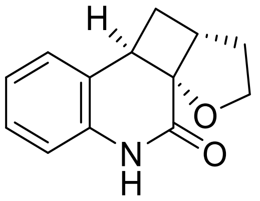

2a

Racemate

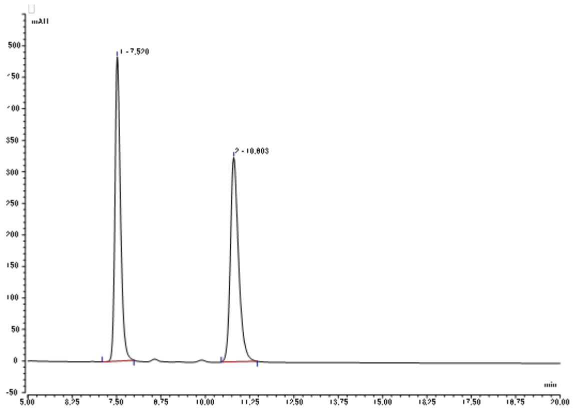

rt (min)	area	%
7,520	87,294	50,17
10,803	86,695	49,83

ODN1-SL-[Ir]

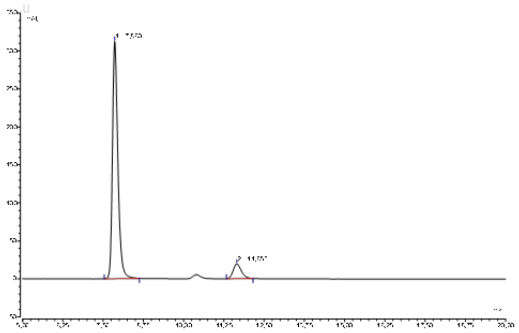

ODN1-SL-[ΔIr]

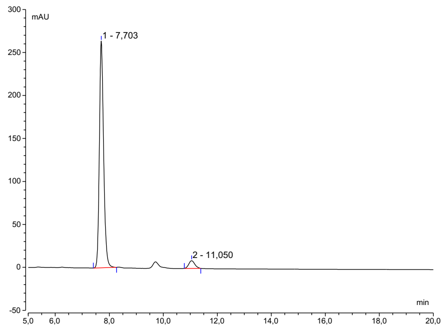

rt (min)	area	%	rt (min)	area	%
7,860	60,645	91,38	7,703	48,274	95,45
10,397(subs.)	1,465	-	9,713 (subs.)	1,732	-
11,653	5,723	8,62	11,050	2,300	4,55

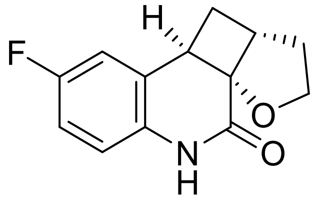

2b

Racemate

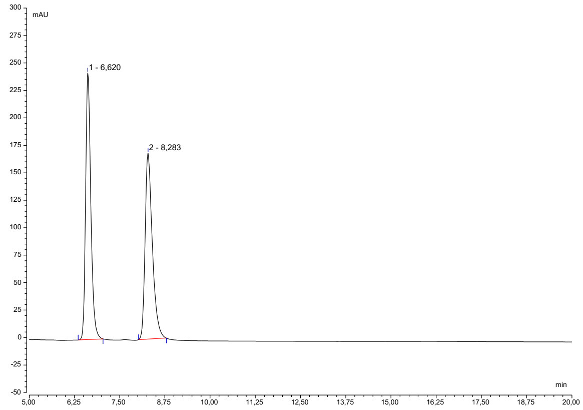

rt (min)	area	%
6,620	40,387	50,71
8,283	39,261	49,29

ODN$_1$-S$_L$-[Ir]

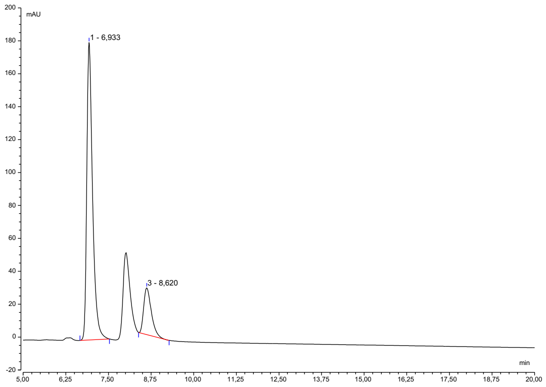

ODN$_1$-S$_L$-[ΔIr]

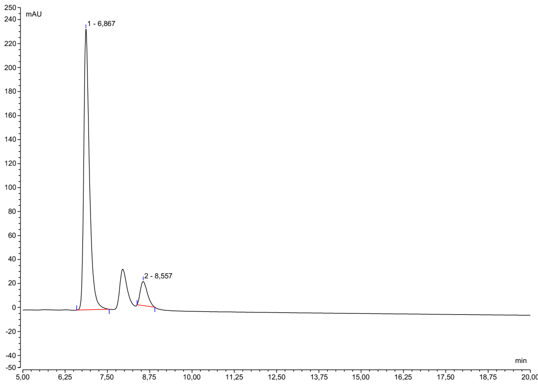

rt (min)	area	%	rt (min)	area	%
6,933	35,231	82,78	6,867	45,580	90,37
8,013 (subs.)	12,146	-	7,953 (subs.)	7,679	-
8,620	7,331	17,22	8,557	4,855	9,63

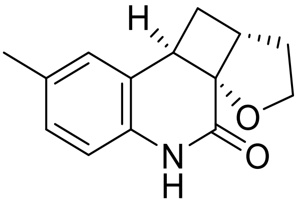

2c

Racemate

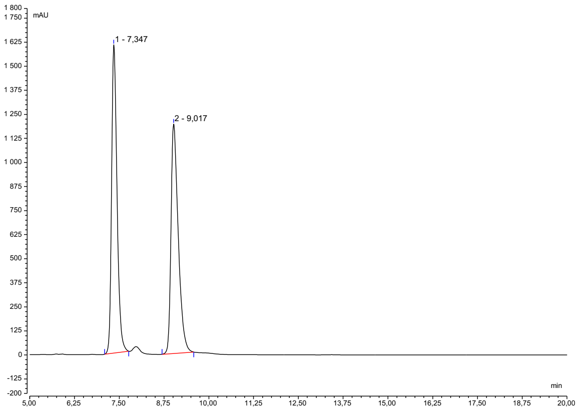

rt (min)	area	%
7,347	282,160	50,15
9,017	280,422	49,85

ODN_1-S_L-[Ir]

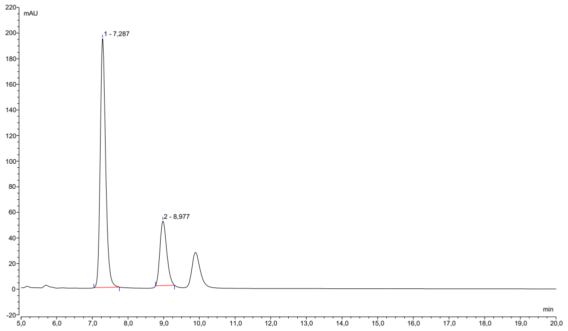

rt (min)	area	%
7,287	34,172	75,45
8,977	11,118	24,55
9,890 (subs.)	6,997	-

ODN_1-S_L-[ΔIr]

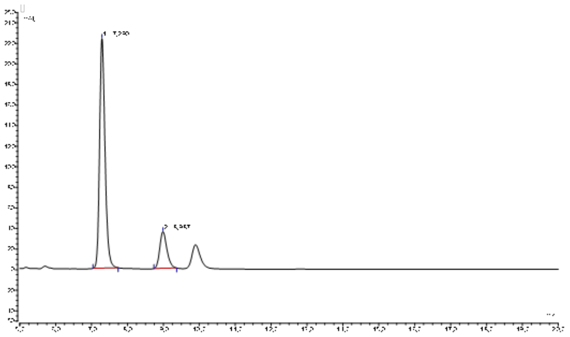

rt (min)	area	%
7,290	39,570	83,47
8,987	7,838	16,53
9,897 (subs.)	5,885	-

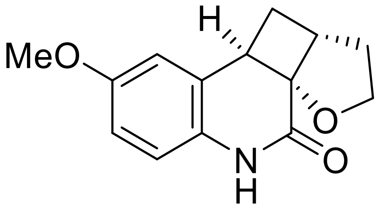

2d

Racemate

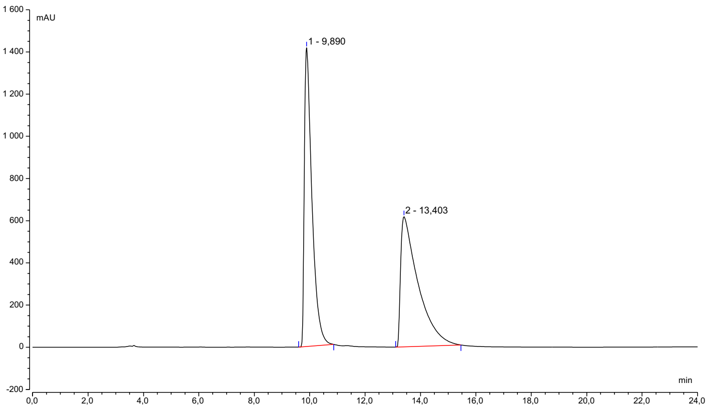

rt (min)	area	%
9,890	450,700	50,03
13,403	450,207	49,97

ODN$_1$-S$_L$-[Ir]

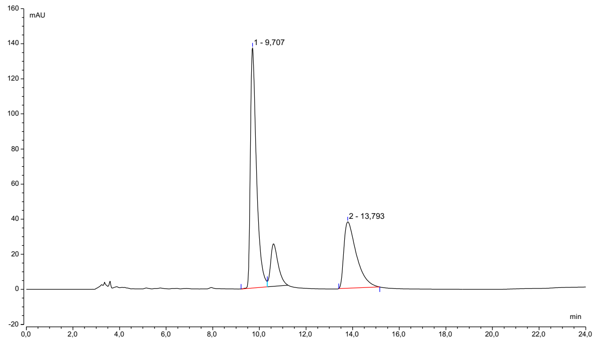

ODN$_1$-S$_L$-[ΔIr]

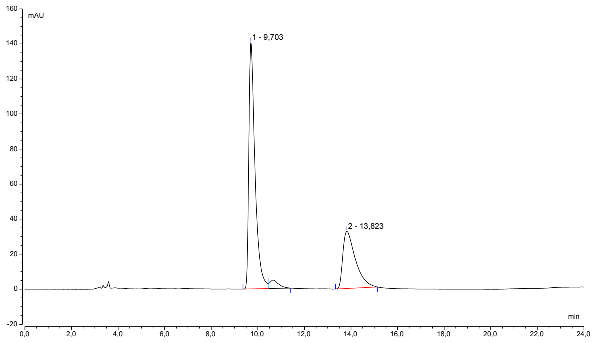

rt (min)	area	%
9,707	41,420	63,53
10,607(subs.)	9.260	-
13,793	23,779	36,47

rt (min)	area	%
9,703	43,294	69.97
10,657(subs.)	1,690	-
13,823	18,579	30.03

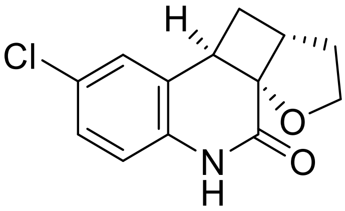

2e

Racemate

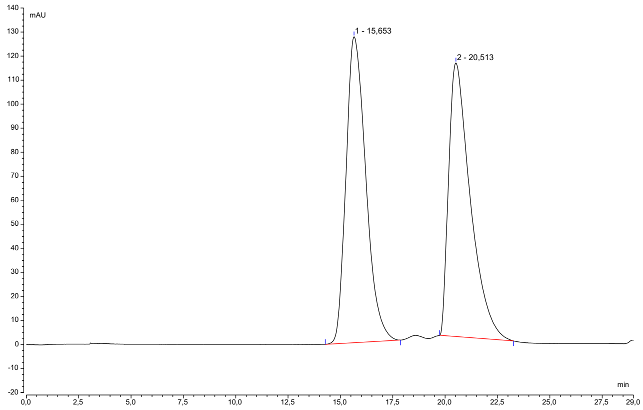

rt (min)	area	%
15,653	139,960	50,64
20,513	136,440	49,36

ODN$_1$-S$_L$-[Ir]

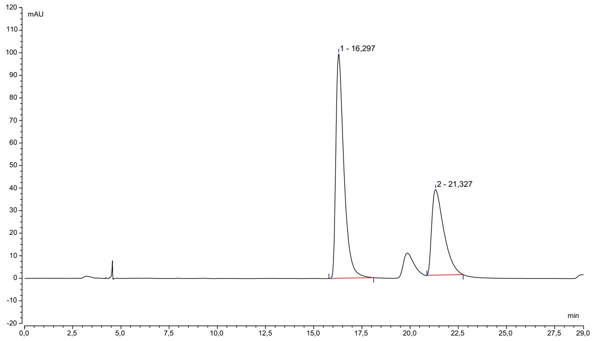

ODN$_1$-S$_L$-[ΔIr]

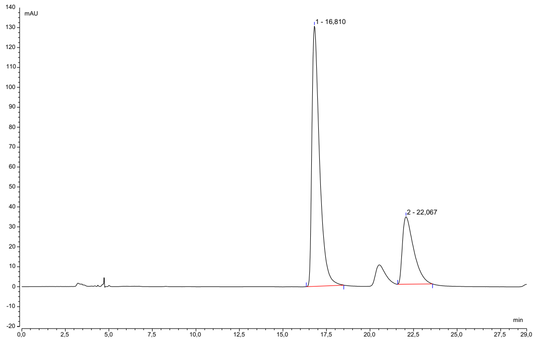

rt (min)	area	%
16,297	48,565	63,73
19.860(subs.)	6,664	-
21,327	27,645	36,27

rt (min)	area	%
16,810	66,202	72,27
20,533(subs.)	6,445	-
22,067	25,398	27,73

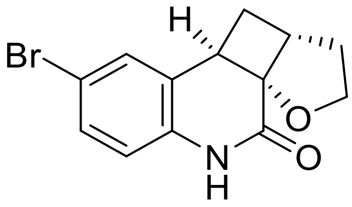

2f

Racemate

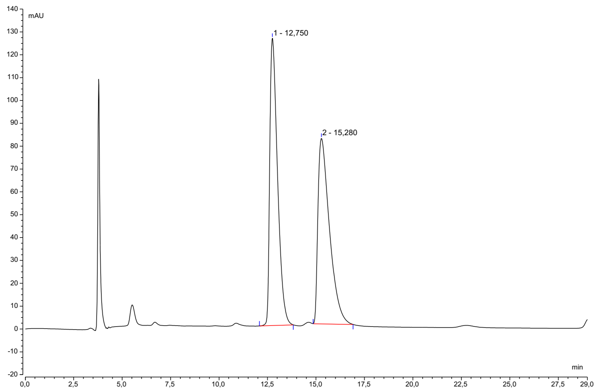

rt (min)	area	%
12,750	58,597	50,91
15,280	56,502	49,09

ODN$_1$-S$_L$-[Ir]

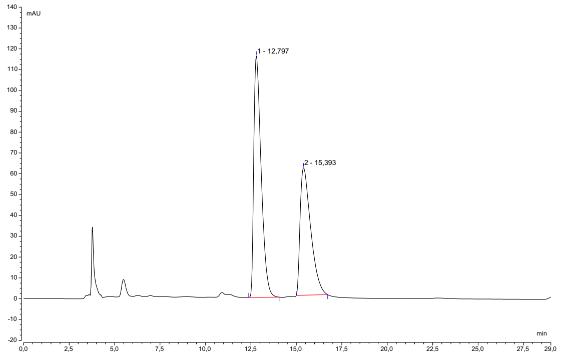

ODN$_1$-S$_L$-[ΔIr]

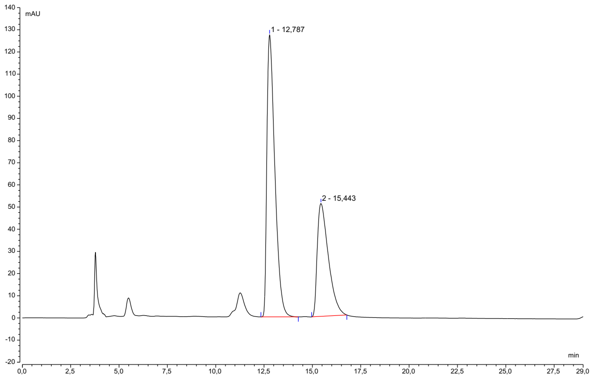

rt (min)	area	%	rt (min)	area	%
10,907 (subs.)	0,880	-	11,267 (subs.)	4,695	-
12,797	54,493	56,98	12,787	60,642	64,05
15,393	41,142	43,02	15,443	34,040	35,95

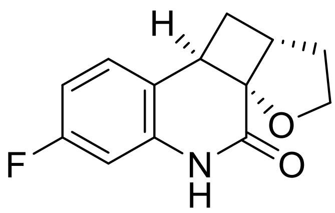

2g

Racemate

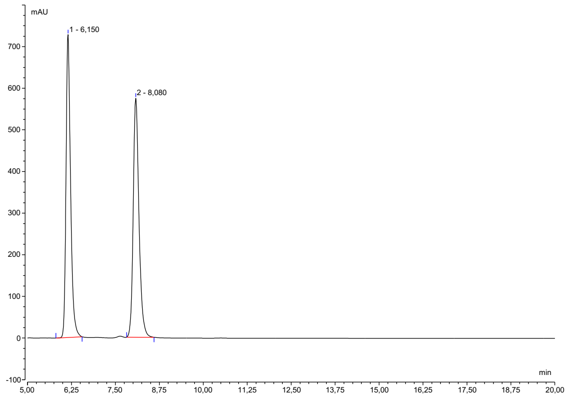

rt (min)	area	%
6,140	109,449	50,14
8,080	108,843	49,86

ODN1-SL-[Ir]

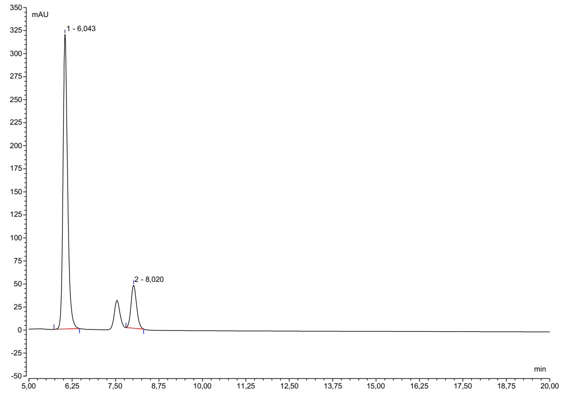

rt (min)	area	%
6,043	48,650	84,89
7,537 (subs.)	5,694	-
8,020	8,662	15,11

ODN1-SL-[ΔIr]

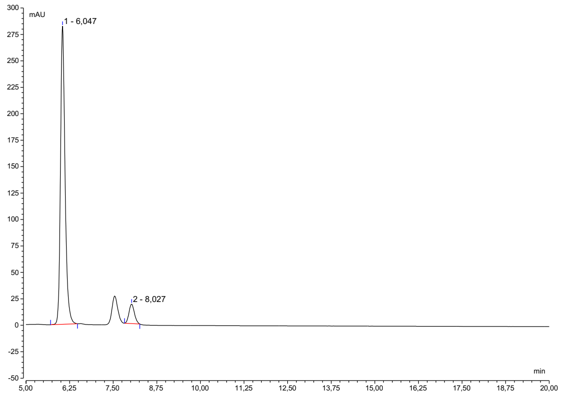

rt (min)	area	%
6,047	42,785	92,80
7,543 (subs.)	4,949	-
8,027	3,321	7,20

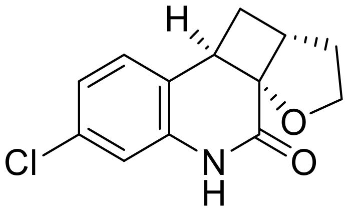

2h

Racemate

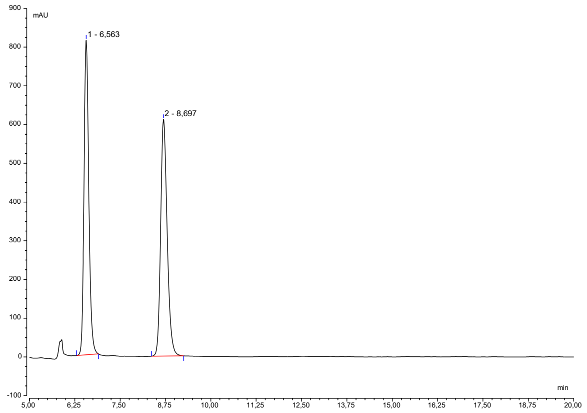

rt (min)	area	%
6,563	124,621	49,59
8,697	126,673	50,41

ODN_1-S_L-[Ir]

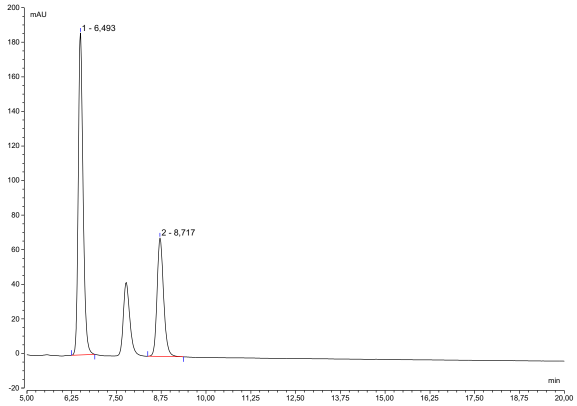

ODN_1-S_L-[ΔIr]

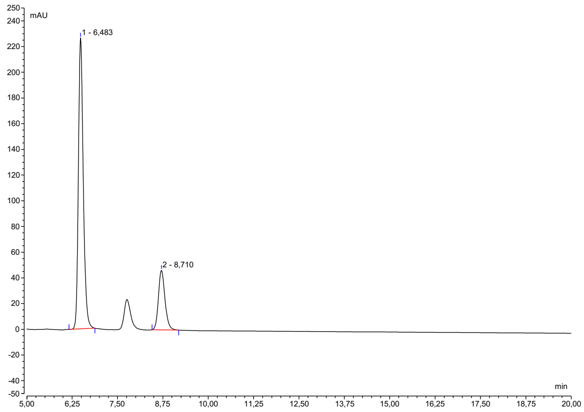

rt (min)	area	%	rt (min)	area	%
6,493	29,114	66,82	6,483	35,189	78,44
7,770 (subs.)	8,263	-	7,763 (subs.)	4,598	-
8,717	14,456	33,18	8,710	9,673	21,56

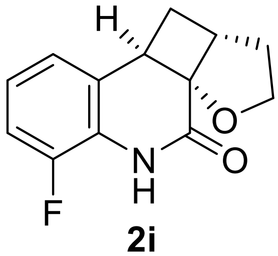

2i

Racemate

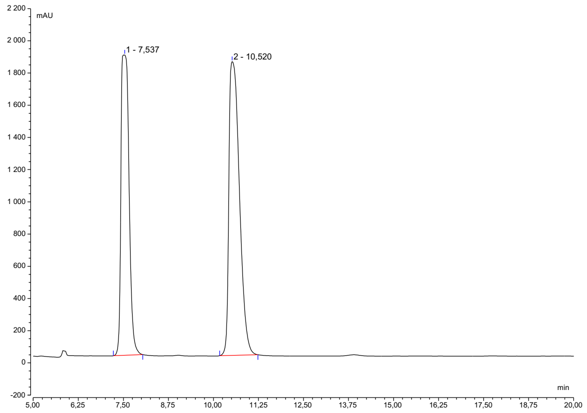

rt (min)	area	%
7,537	484,547	49,91
10,520	486,322	50,09

ODN$_1$-S$_L$-[Ir]

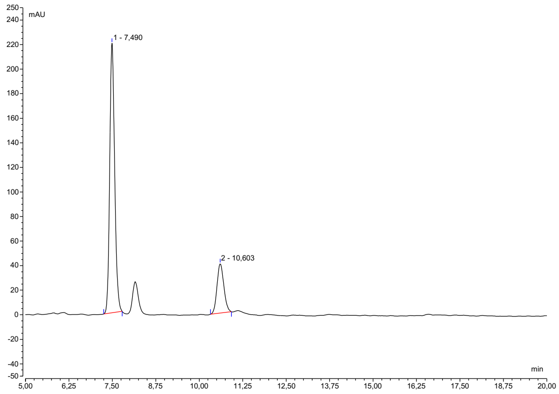

rt (min)	area	%
7,490	36,160	79,88
8,157 (subs.)	5,055	-
10,603	9,110	20,12

ODN$_1$-S$_L$-[ΔIr]

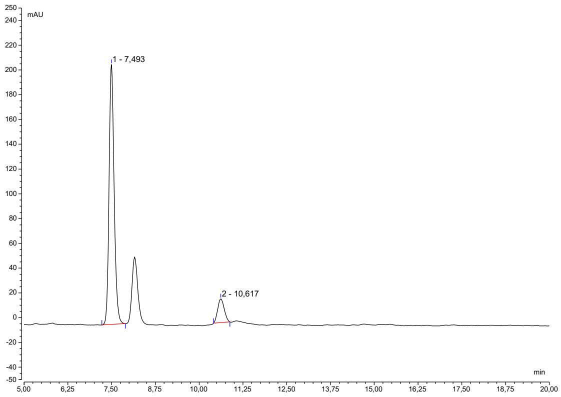

rt (min)	area	%
7,493	35,018	89,43
8,157 (subs.)	10,411	-
10,617	4,139	10,57

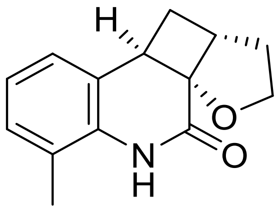

2j

Racemate

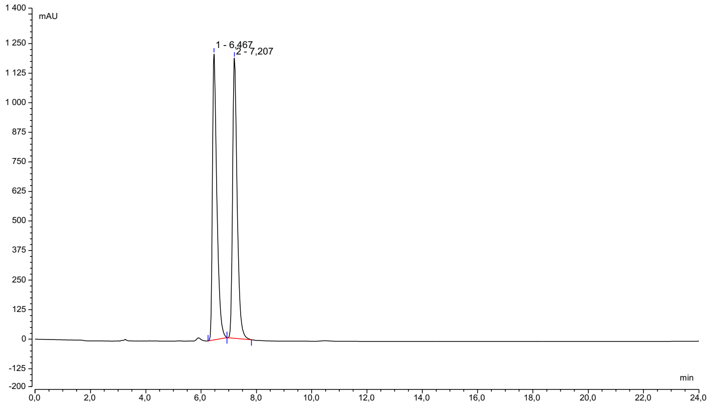

rt (min)	area	%
6,467	225,274	49,83
7,207	226,804	50,17

ODN$_1$-S$_L$-[Ir]

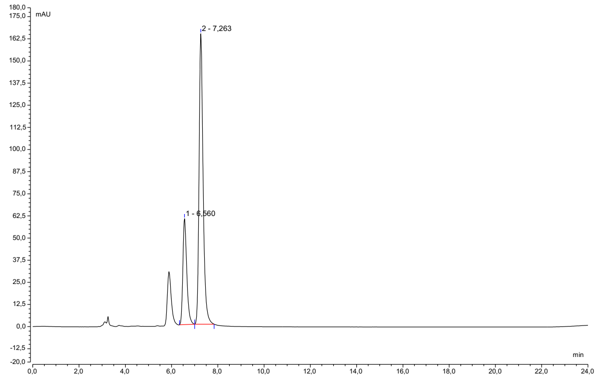

ODN$_1$-S$_L$-[ΔIr]

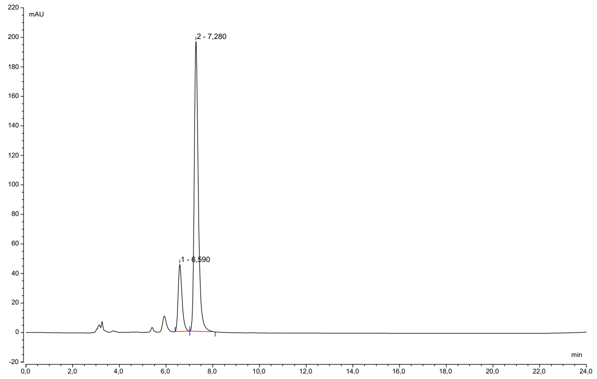

rt (min)	area	%	rt (min)	area	%
5,887 (subs.)	6,007	-	5,927 (subs.)	2,140	-
6,560	11,642	74,01	6,687	8,750	81,93
7,263	33,145	25,99	7,353	39,678	18,07

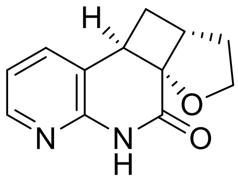

2k

Racemate

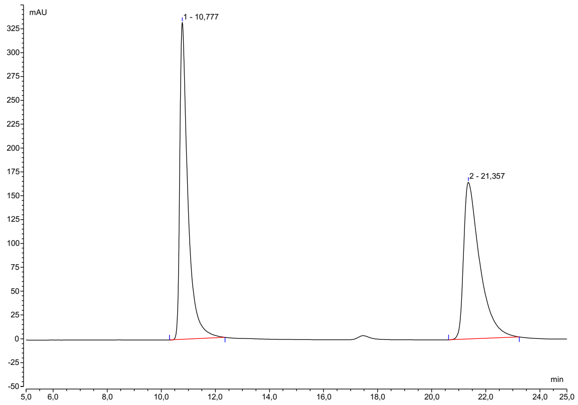

rt (min)	area	%
10,777	115,498	50,39
21,357	113,725	49,61

ODN$_1$-S$_L$-[Ir]

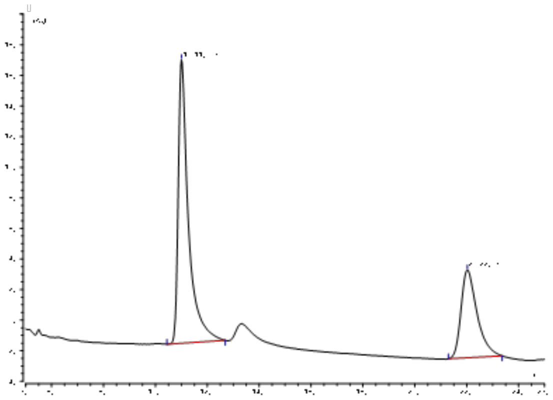

ODN$_1$-S$_L$-[ΔIr]

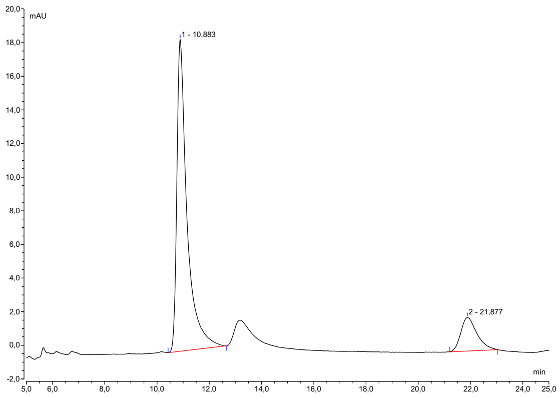

rt (min)	area	%	rt (min)	area	%
11,003	8,445	67,36	10,883	8,374	85,50
13,327 (sub)	0,650	-	13,190 (sub)	1,049	-
22,030	4,093	32,64	21,877	1,420	14,50

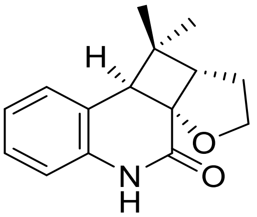

2l

Racemate

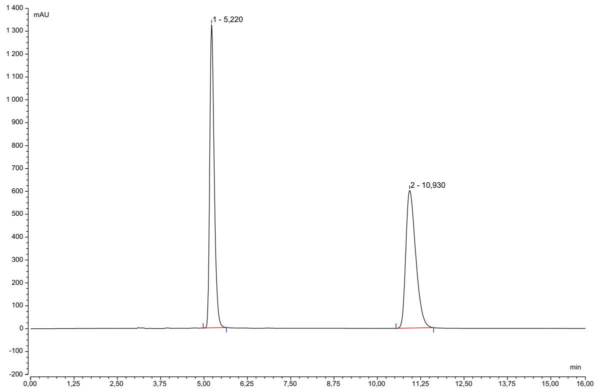

rt (min)	area	%
5,220	199,119	49,97
10,930	199,335	50,03

ODN$_1$-S$_L$-[Ir]

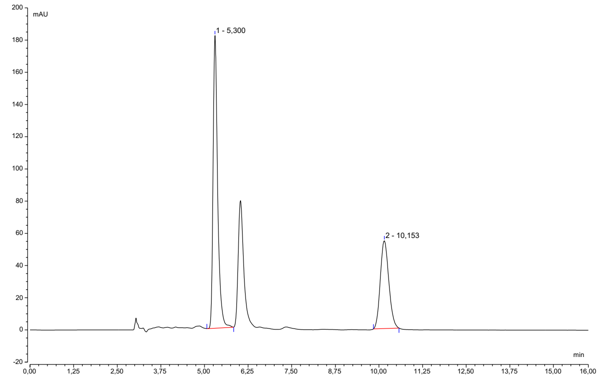

ODN$_1$-S$_L$-[ΔIr]

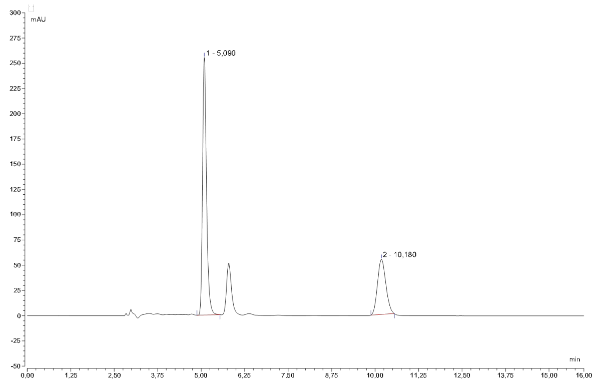

rt (min)	area	%	rt (min)	area	%
5,300	27,468	64,10	5,090	33,599	69,00
6,033 (subs.)	14,073	-	5,790 (subs.)	8,034	-
10,153	15,387	35,90	10,180	15,097	31,00

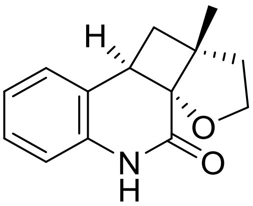

2m

Racemate

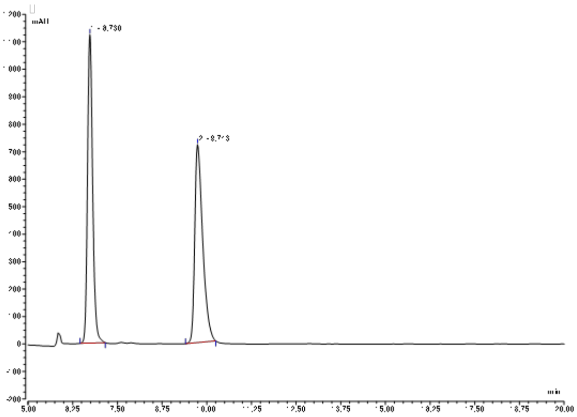

rt (min)	area	%
6,730	187,157	50,13
9,743	186,159	49,87

ODN$_1$-S$_L$-[Ir]

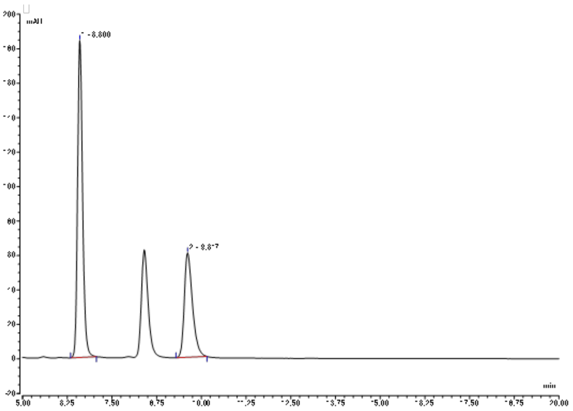

ODN$_1$-S$_L$-[ΔIr]

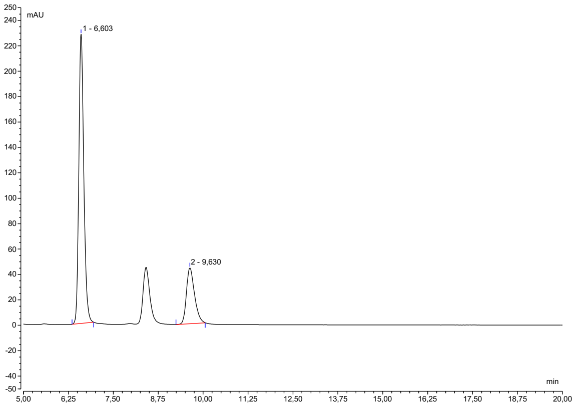

rt (min)	area	%	rt (min)	area	%
6,600	29,855	65,74	6,603	36,546	77,63
8,403 (subs.)	13,625	-	8,410 (subs.)	9,860	-
9,617	15,559	34,26	9,630	10,531	22,37

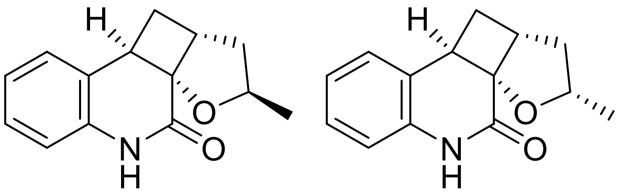

2n

Racemate

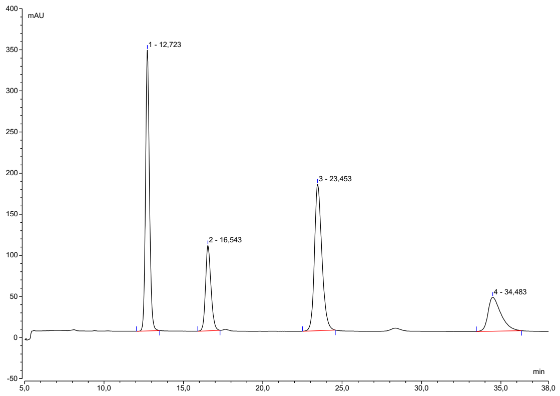

rt (min)	area	%
12,723	97,504	35,73
16,543	40,723	14,92
23,453	96,096	35,21
34,483	38,604	14,14

ODN$_1$-S$_L$-[Ir]

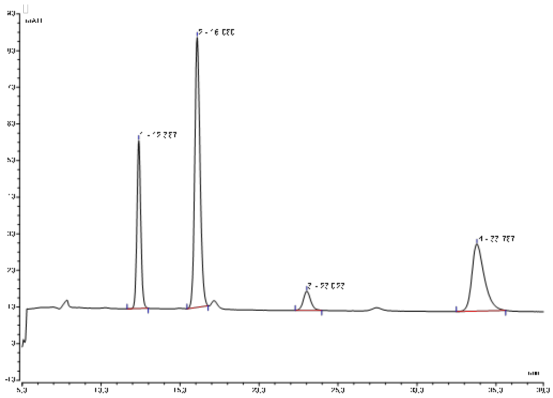

ODN$_1$-S$_L$-[ΔIr]

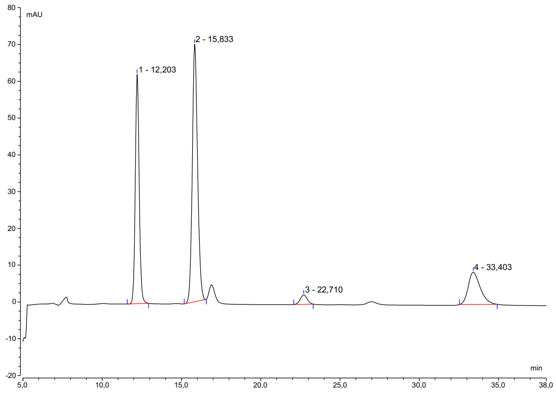

rt (min)	area	%	rt (min)	area	%
12,387	13,001	21,31	12,203	17,530	32,77
16,080	28,549	46,79	15,833	26,968	50,41
17,160 (subs.)	0,657	-	16,893 (subs.)	1,722	-
23,023	2,805	4,60	22,710	1,309	2,45
33,787	16,657	27,30	33,403	7,695	14,38

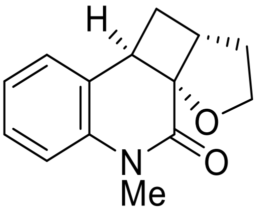

2o

Racemate

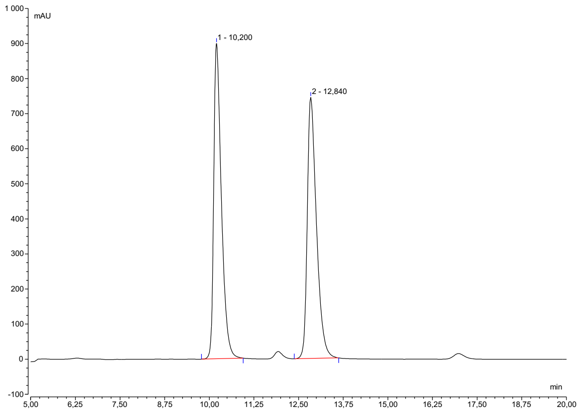

rt (min)	area	%
10,200	222,811	50,04
12,840	222,427	49,96

ODN1-SL-[Ir]

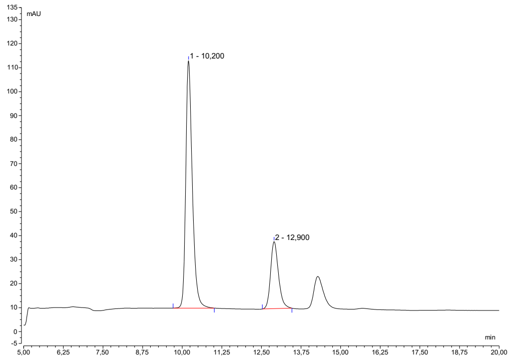

ODN1-SL-[ΔIr]

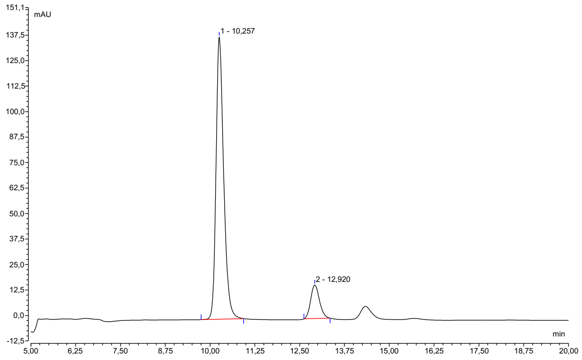

rt (min)	area	%	rt (min)	area	%
10,200	24,714	75,46	10,257	34,180	87,93
12,900	8,039	24,54	12,920	4,693	12,07
14,280 (subs.)	4,548	-	14,333 (subs.)	2,046	-

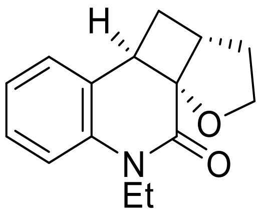

2p

Racemate

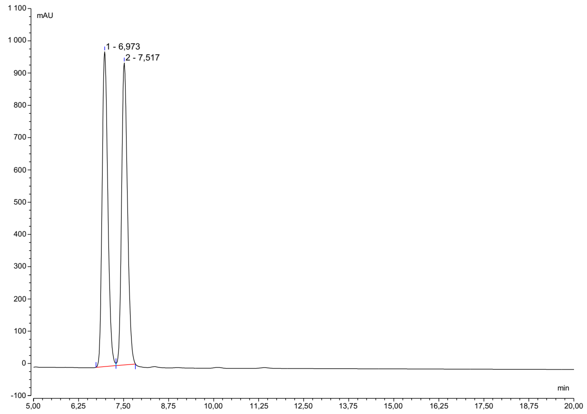

rt (min)	area	%
6,973	179,156	50,29
7,517	177,065	49,71

ODN1-SL-[Ir]

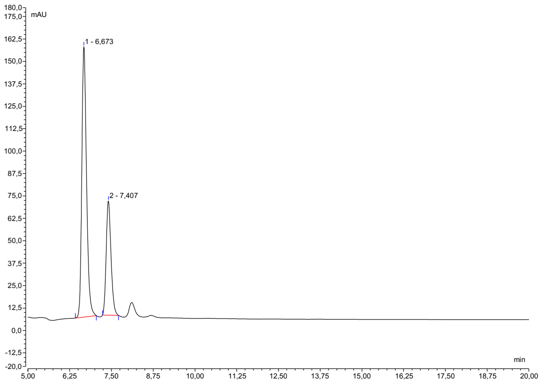

rt (min)	area	%
6,673	24,697	69,91
7,407	10,629	30,09
8,107 (subs.)	1,564	-

ODN1-SL-[ΔIr]

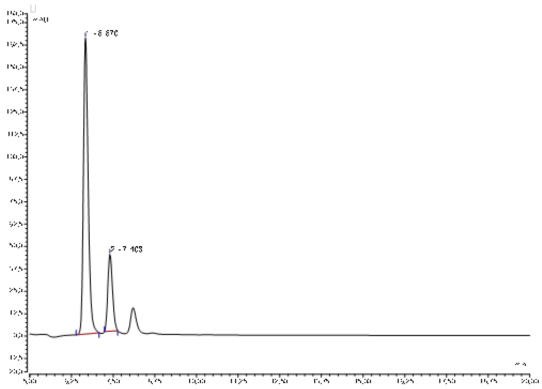

rt (min)	area	%
6,670	27,041	79,51
7,403	6,969	20,49
8,100 (subs.)	2,732	-

3. (L)-DNA serie

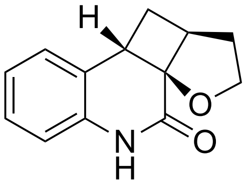

2a’

Racemate

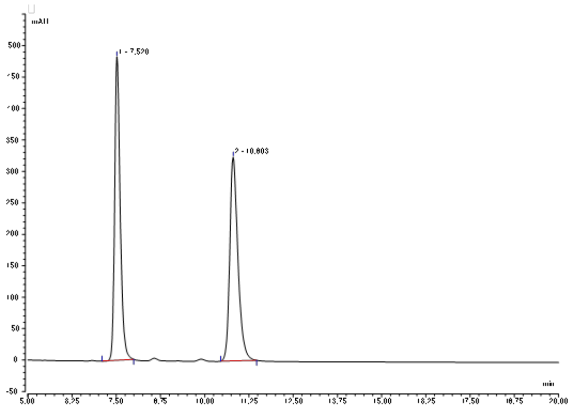

rt (min)	area	%
7,520	87,294	50,17
10,803	86,695	49,83

(D)ODN$_1$-S$_L$-[ΔIr]

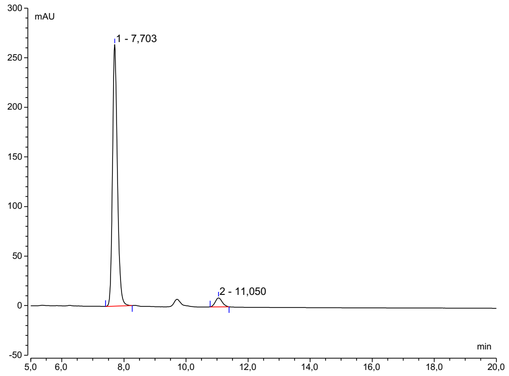

(L)ODN$_1$-S$_D$-[ΛIr]

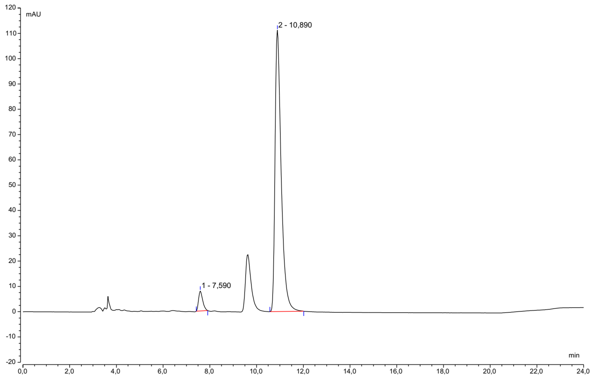

rt (min)	area	%	rt (min)	area	%
7,703	48,274	95,45	7,590	1,615	4,55
9,713 (subs.)	1,732	-	9,620 (subs.)	5,997	-
11,050	2,300	4,55	10,890	33,903	95,45

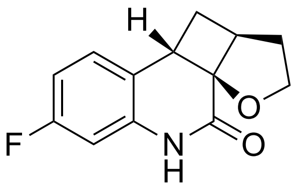

2g’

Racemate

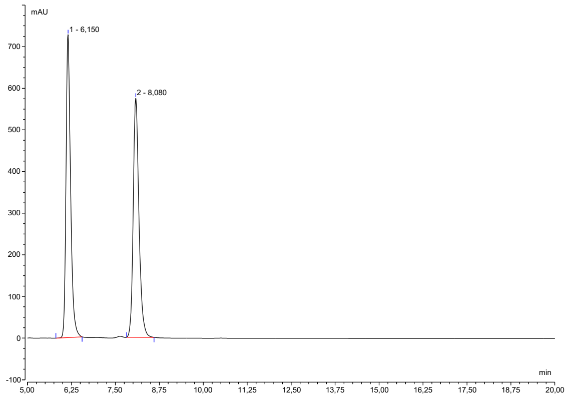

rt (min)	area	%
6,140	109,449	50,14
8,080	108,843	49,86

((D)ODN$_1$-S$_L$-[ΔIr]

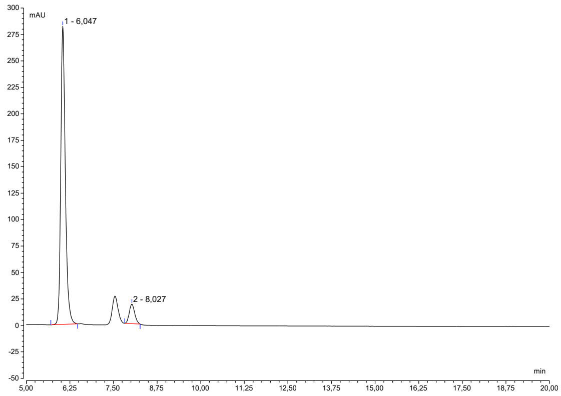

(L)ODN$_1$-S$_D$-[ΛIr]

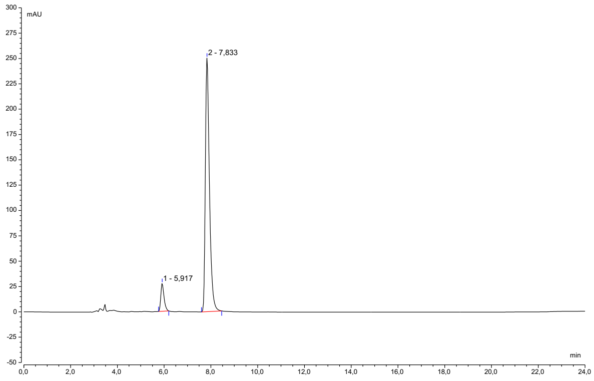

rt (min)	area	%	rt (min)	area	%
6,047	42,785	92,80	5,917	4,348	7,92
7,543 (subs.)	4,949	-	-	-	-
8,027	3,321	7,20	7,833	50,560	92,08

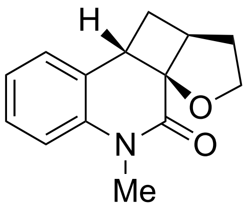

2o’

Racemate

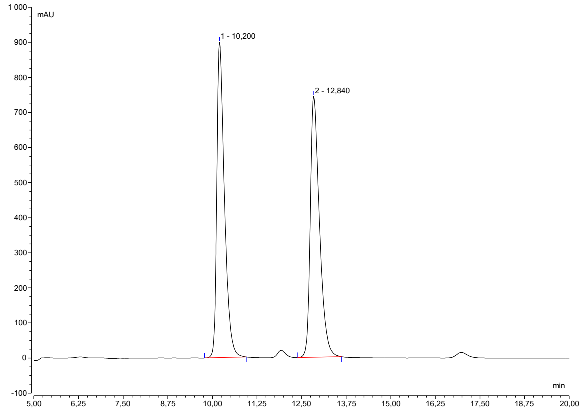

rt (min)	area	%
10,200	222,811	50,04
12,840	222,427	49,96

(D)ODN$_1$-S$_L$-[ΔIr]

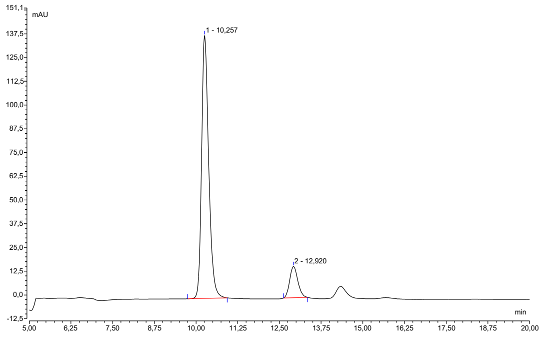

(L)ODN$_1$-S$_D$-[ΛIr]

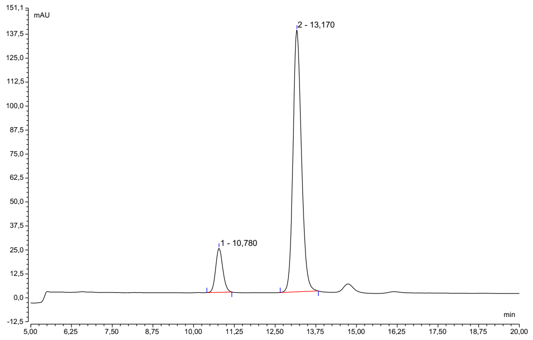

rt (min)	area	%	rt (min)	area	%
10,257	34,180	87,93	10,780	5,845	12,46
12,920	4,693	12,07	13,170	41,063	87,54
14,333 (sub)	2,046	-	14,740 (sub)	1,422	-

XIV. Supplementary References

(1) M. S. Oderinde, J. W. Johannes, Practical Syntheses of [2,2'-Bipyridine]Bis[3,5-Difluoro-2-[5-(Trifluoromethyl)-2-Pyridinyl]Phenyl]Iridium(III) Hexafluorophosphate, [Ir{dF(CF_3)ppy}$_2$(bpy)]PF_6 and [4,4'-Bis(Tert-Butyl)-2,2'-Bipyridine]Bis[3,5-Difluoro-2-[5-(Trifluoromethyl)-2-Pyridinyl]Phenyl]Iridium(III) Hexafluorophosphate, [Ir{dF(CF_3)ppy}$_2$(dtbbpy)]PF_6. *Org. Synth.* **2017**, *94*, 77–92.

(2) M. Á.Martínez, M. P. Carranza, A. Massaguer, L. Santos, J. A. Organero, C. Aliende, R. de Llorens, I. Ng-Choi, L. Feliu, M. Planas, A. M. Rodríguez, B. R. Manzano, G. Espino, F. A. Jalón, Synthesis and Biological Evaluation of Ru(II) and Pt(II) Complexes Bearing Carboxyl Groups as Potential Anticancer Targeted Drugs. *Inorg. Chem.* **2017**, *56*, 13679–13696.

(3) N. Duchemin, S. Aubert, J. V. De Souza, L. Bethge, S. Vonhoff, A. K. Bronowska, M. Smietana, S. Arseniyadis, New Benchmark in DNA-Based Asymmetric Catalysis: Prevalence of Modified DNA/RNA Hybrid Systems. *JACS Au* **2022**, *2*, 1910–1917.

(4) K. L. Skubi, J. B. Kidd, H. Jung, I. A. Guzei, M.-H. Baik, T. P. Yoon, Enantioselective Excited-State Photoreactions Controlled by a Chiral Hydrogen-Bonding Iridium Sensitizer. *J. Am. Chem. Soc.* **2017**, *139*, 17186–17192.

(5) A. J. Duplantier, S. L. Becker, M. J. Bohanon, K. A. Borzilleri, B. A. Chrunyk, J. T. Downs, L.-Y. Hu, A. El-Kattan, L. C. James, S. Liu, J. Lu, N. Maklad, M. N. Mansour, S. Mente, M. A. Piotrowski, S. M. Sakya, S. Sheehan, S. J. Steyn, C. A. Strick, V. A. Williams, L. Zhang, Discovery, SAR, and Pharmacokinetics of a Novel 3-Hydroxyquinolin-2(1H)-One Series of Potent d-Amino Acid Oxidase (DAAO) Inhibitors. *J. Med. Chem.* **2009**, *52*, 3576–3585.

(6) K. C. Majumdar, P. P. Mukhopadhyay, P. K. Basu, Regioselective Synthesis of Coumarin and Quinolone-Annulated Spiro Heterocycles via Aryl Radical Cyclization. *Synth. Commun.* **2005**, *35*, 1291–1299.

(7) D.A. Case, H.M. Aktulga, K. Belfon, I.Y. Ben-Shalom, J.T. Berryman, S.R. Brozell, D.S. Cerutti, T.E. Cheatham, III, G.A. Cisneros, V.W.D. Cruzeiro, T.A. Darden, N. Forouzesh, M. Ghazimirsaeed, G. Giambaşu, T. Giese, M.K. Gilson, H. Gohlke, A.W. Goetz, J. Harris, Z. Huang, S. Izadi, S.A. Izmailov, K. Kasavajhala, M.C. Kaymak, A. Kovalenko, T. Kurtzman, T.S. Lee, P. Li, Z. Li, C. Lin, J. Liu, T. Luchko, R. Luo, M. Machado, M. Manathunga, K.M. Merz, Y. Miao, O. Mikhailovskii, G. Monard, H. Nguyen, K.A. O'Hearn, A. Onufriev, F. Pan, S. Pantano, A. Rahnamoun, D.R. Roe, A. Roitberg, C. Sagui, S. Schott-Verdugo, A. Shajan, J. Shen, C.L. Simmerling, N.R. Skrynnikov, J. Smith, J. Swails, R.C. Walker, J. Wang, J. Wang, X. Wu, Y. Wu, Y. Xiong, Y. Xue, D.M. York, C. Zhao, Q. Zhu, and P.A. Kollman (2024), Amber 2024, University of California, San Francisco.

(8) Gaussian 16, Revision B.01, M. J. Frisch, G. W. Trucks, H. B. Schlegel, G. E. Scuseria, M. A. Robb, J. R. Cheeseman, G. Scalmani, V. Barone, G. A. Petersson, H. Nakatsuji, X. Li, M. Caricato, A. V. Marenich, J. Bloino, B. G. Janesko, R. Gomperts, B. Mennucci, H. P. Hratchian, J. V. Ortiz, A. F. Izmaylov, J. L. Sonnenberg, D. Williams-Young, F. Ding, F. Lipparini, F. Egidi, J. Goings, B. Peng, A. Petrone, T. Henderson, D. Ranasinghe, V. G. Zakrzewski, J. Gao, N. Rega, G. Zheng, W. Liang, M.

Hada, M. Ehara, K. Toyota, R. Fukuda, J. Hasegawa, M. Ishida, T. Nakajima, Y. Honda, O. Kitao, H. Nakai, T. Vreven, K. Throssell, J. A. Montgomery, Jr., J. E. Peralta, F. Ogliaro, M. J. Bearpark, J. J. Heyd, E. N. Brothers, K. N. Kudin, V. N. Staroverov, T. A. Keith, R. Kobayashi, J. Normand, K. Raghavachari, A. P. Rendell, J. C. Burant, S. S. Iyengar, J. Tomasi, M. Cossi, J. M. Millam, M. Klene, C. Adamo, R. Cammi, J. W. Ochterski, R. L. Martin, K. Morokuma, O. Farkas, J. B. Foresman, and D. J. Fox, Gaussian, Inc., Wallingford CT, **2016**.

(9) P. Li. K. M. Merz Jr., MCPB.py: A Python Based Metal Center Parameter Builder. *J. Chem. Inf. Model.,* ***2016***, *56*, 599-604.

(10) J. A. Maier, C. Martinez, K. Kasavajhala, L. Wickstrom, K. E. Hauser, C. Simmerling, ff14SB: Improving the Accuracy of Protein Side Chain and Backbone Parameters from ff99SB. *J. Chem. Theor. Comput.,* **2015***, 11*, 3696-3713.

(11) I. Ivani, P. D. Dans, A. Noy, A. Pérez, I. Faustino, A. Hospital, J. Walther, P. Andrio, R. Goñi, A. Balaceanu, G. Portella, F. Battistini, J. L. Gelpí, C. González, M. Vendruscolo, C. A Laughton, S. A Harris, D.A Case, M. Orozco, Parmbsc1: a refined force field for DNA simulations. *Nature Methods*, **2016**, *13*, 55-58.

(12) T. Novoa, R. Laplaza, F. Peccati, F. Fuster, J. Contreras-Garcia, The NCIWEB Server: A Novel Implementation of the Noncovalent Interactions Index for Biomolecular Systems. *J. Chem. Inf. Model.*, **2023**, *63*, 4483-4489.

(13) M. M. Maturi, M. Wenninger, R. Alonso, A. Bauer, A. Pöthig, E. Riedle, T. Bach, Intramolecular [2+2] Photocycloaddition of 3- and 4-(But-3-Enyl)Oxyquinolones: Influence of the Alkene Substitution Pattern, Photophysical Studies, and Enantioselective Catalysis by a Chiral Sensitizer. *Chem. - Eur. J.* **2013**, *19*, 7461–7472.
