# Supplementary material for: A metal-DNA biohybrid as enantioselective artificial photoDNAzyme
Source: Nat Commun. 2026 May 13;17:7527. doi: 10.1038/s41467-026-72881-z (PMC13409001; doi:10.1038/s41467-026-72881-z)
Supplement: Supplementary file 3 — Description of Additional Supplementary Files [file 41467_2026_72881_MOESM3_ESM.pdf]

Description of Additional Supplementary Files:

Supplementary Movie 1: Molecular dynamics trajectory of the best-performing artificial metallo-photoDNAzyme showing the positioning of the iridium photocatalyst within the DNA double helix and the association of the substrate with the DNAdefined chiral active site.
