# Supplementary material for: A metal-DNA biohybrid as enantioselective artificial photoDNAzyme
Source: Nat Commun. 2026 May 13;17:7527. doi: 10.1038/s41467-026-72881-z (PMC13409001; doi:10.1038/s41467-026-72881-z)
Supplement: Supplementary file 2 — Peer Review file [file 41467_2026_72881_MOESM2_ESM.pdf]

nature portfolio

Peer Review File

A metal-DNA biohybrid as enantioselective artificial photoDNAzyme

Corresponding Author: Dr Olivier BASLE

This file contains all reviewer reports in order by version, followed by all author rebuttals in order by version.

Version 0:

Reviewer comments:

Reviewer #1

(Remarks to the Author)
Smitetana and coworkers report a DNA-based artificial photoenzyme-catalyzed enantioselective intramolecular 2+2 cycloaddition reaction. This work is a clear advancement of the asymmetric DNA catalysis field as the only precedent on photo asymmetric DNA catalysis gave up to 28% ee for a similar 2+2 cycloaddition with a covalently attached benzophenone photocatalyst. In this work, the authors employed a well established Ir catalyst and covalently attached it to DNA. Interestingly, they found one enantiomer of the Ir cat. when attached to DNA gave significantly higher ee than the other enantiomer, which was attributed to different reaction kinetics. The hypothesis is well supported by spectroscopic data and MD simulations. The substrate scope is somewhat similar to enzyme catalysis, ee is sensitive to substituents. Overall, this is a well executed study and represents a clear advancement to the field of asymmetric DNA catalysis. Thus, I recommend its publication in Nat Comm after they successfully address the comments/concerns below.
1. Page 2, 2nd paragraph, "no enantioselective protein-based...", it sounds like no enzyme-catalyzed 2+2 cycloaddition has been reported if not read carefully. Those examples on enzyme catalysis should be mentioned here.
2. Page 3, 3rd paragraph, "changing the nature of the base pairs upstream and downstream of the photocatalyst,..." In Table S7, there is no such information provided. The neighboring base pairs are critical for stereocontrol (J. Am. Chem. Soc. 2024, 146, 16531). It will be useful to systematically evaluate the neighboring base pairs.
3. SI, MALDI and HPLC, should be profile not "profil".
4. Page 8, 1st paragraph, line 3, "evidences that 1a in well..." should be "evidences that 1a is well..."

Reviewer #2

(Remarks to the Author)
This manuscript by Smietana and coworkers details the development and application of the first photocatalytic Ir-metalloenzyme towards asymmetric [2+2] cycloaddition. The manuscript is, on the whole, well written with well-made, clear schemes. There are some minor grammatical and spelling issues (bioconjugaison). The conceptualization of the project, based upon the earlier work of Wagenknecht, is well justified, as is recent work by Green and Wu. The optimization process of both PC chain length, sequence position, and surrounding residues is comprehensive and the use of single-enantiomer Ir photocatalysts to reinforce the transfer of chirality within the duplex is notable. The scope, while sufficiently probed around the basic quinolone scaffold, does raise some questions around generality of the approach – especially considering these similar quinolone substrates have been the subject of similar biophotocatalytic transformations. Can the authors comment on the reactivity of indole-based substrates as employed by Wu (Nature, 2022) and the 3-substituted quinolones used by Green (Nature, 2022).

While the energy transfer mechanism proposed by the authors certainly seems the most likely, the use of an Ir(dFCF3)ppy2 photocatalyst certainly raises the possibility of SET, and may be a source of future studies. However, given the large redox potentials exhibit and large triplet energy of these catalysts, studies to conclusively rule out SET should be looked into. Tethering a photocatalyst with similar redox potentials but a triplet energy lower than 55 kcal/mol should result in no conversion.

The SI is well written and comprehensive. I have some reservations about the generalization method employed when assessing conversion and the interpretation of e.r.'s. In other comparable studies, calibration curves are included for every substrate. While the difference may be small, given that you are comparing yields and ee's within each substrate (at similar

but not consistent concentrations), this may be worthwhile. I would also note that the separation of enantiomers with overlapping starting material signals is generally good, but in my view not acceptable for compounds 2F or 2L, and some further HPLC optimization needs to be conducted here.

Overall, I think this is a good piece of work and suitable for publication in Nature Communications upon minor revisions addressing the issues raised.

Reviewer #3

(Remarks to the Author)
This manuscript reports the design of an artificial photoDNAzyme created by covalently embedding an iridium (Ir) metal photocatalyst within a DNA double helix, which is applied to [2+2] photocycloaddition reactions, with a focus on controlling enantioselectivity. Although the authors' research is well-supported by experimental design and data, from the perspectives of innovation and academic value, it does not provide sufficient justification for publication in Nature Communications.
1. Limited Innovation：The primary novelty of this work lies in the incorporation of an Ir metal photocatalyst into DNA. However, Ir-based photocatalysts have already been widely used in photochemistry and asymmetric catalysis by research groups such as Yoon and Meggers. Therefore, while embedding the Ir photocatalyst in DNA is novel in form, the core innovation is limited.
2. No Clear Advantage Over Existing Research：Ir photocatalysts have already been applied in [2+2] photocycloaddition reactions, and the artificial photoDNAzyme developed in this manuscript does not demonstrate significant advantages over existing systems in terms of substrate scope and catalytic efficiency. In particular, the enantioselectivity and reaction yields reported here do not surpass those from previous studies.
3. Asymmetric [2+2] photocycloaddition reactions have been widely studied and reported (e.g., J. Am. Chem. Soc. 2011, 133, 16689; Chem. Eur. J. 2013, 19, 7461; Angew. Chem. Int. Ed. 2014, 53, 4368; J. Am. Chem. Soc. 2017, 139, 17186), with excellent results in catalytic efficiency and enantioselectivity. The system described in this manuscript does not demonstrate a clear advantage and lacks the potential to lead the field in a new direction.
4. The authors emphasize that current protein-based photoenzyme systems cannot achieve dual enantioselectivity control, while their approach can achieve controlled enantiomer generation. However, dual enantioselectivity control is easily achievable in small molecule catalysis systems (e.g., J. Am. Chem. Soc. 2022, 144, 4206), making the advantage of this catalyst less significant.
While this manuscript presents some exploratory experimental designs and ideas, it falls short in terms of innovation, the advantages of the proposed research system, and its contribution to the existing literature. Therefore, I do not believe the article is suitable for publication in Nature Communications at this stage. I suggest the authors consider submitting the manuscript to a more specialized journal that is better suited to this field.

Version 1:

Reviewer comments:

Reviewer #1

(Remarks to the Author)
The authors have successfully addressed my comments. Thus, I recommend the publication of the revised work on Nature Communications.

Reviewer #2

(Remarks to the Author)
Based on the author's response to the questions, I am satisfied and recommend this manuscript for publication.

Reviewer #3

(Remarks to the Author)
The manuscript has been thoroughly revised and is now suitable for publication in Nature Communications.

Open Access This Peer Review File is licensed under a Creative Commons Attribution 4.0 International License, which permits use, sharing, adaptation, distribution and reproduction in any medium or format, as long as you give appropriate credit to the original author(s) and the source, provide a link to the Creative Commons license, and indicate if changes were made.
In cases where reviewers are anonymous, credit should be given to 'Anonymous Referee' and the source.
The images or other third party material in this Peer Review File are included in the article's Creative Commons license, unless indicated otherwise in a credit line to the material. If material is not included in the article's Creative Commons license and your intended use is not permitted by statutory regulation or exceeds the permitted use, you will need to obtain permission directly from the copyright holder.
To view a copy of this license, visit https://creativecommons.org/licenses/by/4.0/

A metal-DNA biohybrid as enantioselective artificial photoDNAzyme

Response to Reviewers

Reviewer #1:

Smietana and coworkers report a DNA-based artificial photoenzyme-catalyzed enantioselective intramolecular 2+2 cycloaddition reaction. This work is a clear advancement of the asymmetric DNA catalysis field as the only precedent on photo asymmetric DNA catalysis gave up to 28% ee for a similar 2+2 cycloaddition with a covalently attached benzophenone photocatalyst. In this work, the authors employed a well established Ir catalyst and covalently attached it to DNA. Interestingly, they found one enantiomer of the Ir cat. when attached to DNA gave significantly higher ee than the other enantiomer, which was attributed to different reaction kinetics. The hypothesis is well supported by spectroscopic data and MD simulations. The substrate scope is somewhat similar to enzyme catalysis, ee is sensitive to substituents. Overall, this is a well executed study and represents a clear advancement to the field of asymmetric DNA catalysis. Thus, I recommend its publication in Nat Comm after they successfully address the comments/concerns below.

Response: We sincerely thank the reviewer for the enthusiastic support of our work.

Q1. Page 2, 2nd paragraph, "no enantioselective protein-based...", it sounds like no enzyme-catalyzed 2+2 cycloaddition has been reported if not read carefully. Those examples on enzyme catalysis should be mentioned here.

Response: Thanks for the suggestion. Previous works on protein-based enantioselective artificial photoenzymes are now cited here (see ref 27-32).

Q2. Page 3, 3rd paragraph, "changing the nature of the base pairs upstream and downstream of the photocatalyst,..." In Table S7, there is no such information provided. The neighboring base pairs are critical for stereocontrol (J. Am. Chem. Soc. 2024, 146, 16531). It will be useful to systematically evaluate the neighboring base pairs.

Response: We thank the reviewer for pointing this out. In fact, the outcomes of neighboring base pairs effect were omitted from the submitted SI version and we apologize for this. The results are now presented in Table S8 of the revised version of the SI. Based on a rational selection of representative substitutions comparing purines and pyrimidines, the data reveal clear trends: pyrimidines, particularly cytosine (C), perform better than purines (G or A) at positions 6 and 17, while C is also more favorable than T. These observations indicate that the identity of neighboring base pairs is critical for the stereochemical outcome in our system, in line with molecular dynamics studies.

Q3. SI, MALDI and HPLC, should be profile not "profil".

Response: The word “profile” has been corrected in the SI

Q4. Page 8, 1st paragraph, line 3, "evidences that 1a in well..." should be "evidences that 1a is well..."

Response: the typo is now corrected

Reviewer #2:

This manuscript by Smietana and coworkers details the development and application of the first photocatalytic Ir-metalloenzyme towards asymmetric [2+2] cycloaddition. The manuscript is, on the whole, well written with well-made, clear schemes. There are some minor grammatical and spelling issues (bioconjugaison) (Q1). The conceptualization of the project, based upon the earlier work of Wagenknecht, is well justified, as is recent work by Green and Wu. The optimization process of both PC chain length, sequence position, and surrounding residues is comprehensive and the use of single-enantiomer Ir photocatalysts to reinforce the transfer of chirality within the duplex is notable. The scope, while sufficiently probed around the basic quinolone scaffold, does raise some questions around generality of the approach – especially considering these similar quinolone substrates have been the subject of similar biophotocatalytic transformations. Can the authors comment on the reactivity of indole-based substrates as employed by Wu (Nature, 2022) and the 3-substituted quinolones used by Green (Nature, 2022) (Q2).

Response: We thank the referee for their positive comments.

In response to Q1: We are confident that all the minor typographical and grammatical errors have now been corrected.

In response to Q2: In both previous reports by Wu *et al.* (*Nature*, 2022, ref 28) and Green *et al.* (*Nature* 2022, ref 27), benzophenone with a high-energy excited state (E_T = 69.1 kcal·mol^{-1}) was used as photocatalyst. This enables triplet energy transfer sensitization of substrates beyond the reach of common iridium-based photocatalysts.

Regarding indole substrates, Yu and co-workers demonstrated that the model intramolecular [2+2] cycloaddition of indole derivatives did not proceed with Ir-based photosensitizers (*J. Am. Chem. Soc.* **141**, 2636 (2019)). The calculated triplet excited-state energy of the model indole substrate (65 kcal·mol^{-1}) exceeds the triplet energy of $[Ir(dF(CF_3)ppy)_2(dtbbpy)]^+$, that is similar to our photocatalyst ($E_T \approx 61$ kcal·mol^{-1}). Although C2-phenyl-substituted indoles with lower triplet energies might potentially undergo the transformation under our photocatalytic conditions, the overall reaction scope would likely remain narrow, and the reaction selectivity uncertain. Therefore, a substantial reoptimization of the photoDNAzyme sequence would likely be required, similar to what is necessary for protein-based artificial photoenzymes (see ref 27-32), and this lies beyond the scope of the present study.

On the other hand, for quinolone substrates, the triplet energy of 4-(hex-3-enyl)oxyquinolones (Green *et al.*, *Nature* 2022, ref. 28) is predicted to be higher than that of the parent 2(1H)-quinolone, whose triplet energy is reported as 66 kcal·mol^{-1} (ref. 36). Such an energy level lies beyond the accessible range of Ir-based photocatalysts, as we confirmed experimentally in the early stages of our investigation while screening potential substrates: the 4-(hex-3-enyl)oxyquinolone model substrate showed no conversion in organic solution using either $[Ir(dF(CF_3)ppy)_2(dtbbpy)]^+$ or our (non-immobilized) iridium photocatalyst (data not included in the Supporting Info).

Q3. While the energy transfer mechanism proposed by the authors certainly seems the most likely, the use of an Ir(dFCF3)ppy2 photocatalyst certainly raises the possibility of SET, and may be a source of future studies. However, given the large redox potentials exhibit and large triplet energy of these catalysts, studies to conclusively rule out SET should be looked into. Tethering a photocatalyst with similar redox potentials but a triplet energy lower than 55 kcal/mol should result in no conversion.

Response: As previously demonstrated by Yoon and coworkers (ref 32), electrochemical studies (Figure S15) indicate a substrate **1a** oxidation potential of +1.57 V and a reduction potential of -1.71 V vs SCE, which lie both outside the potential window of our photoexcited catalyst (+1.32 and -0.83 respectively), therefore demonstrating that both SET oxidative or reductive quenching of the photoexcited catalyst is not thermodynamically feasible. Moreover, the photocatalytic activity of the

[Ir] photocatalyst was significantly inhibited in the presence of 1 equiv. of 2,5-dimethylhexa-2,4-diene as triplet quencher ($E_T \approx$ 58kcal/mol) (Figure S16). Finally, replacing Ir photocatalyst by $[Ru(bpy)_3](PF_6)_2$ (E_{red}* = +0.77, E_{ox}* =-0.81) with a lower triplet energy ($E_T \approx$ 49 kcal·mol^{-1}) results in no reaction under the standard conditions to synthetize the racemic product **2a** (Figure S16). The above new results (Figures S15-S16) are therefore consistent with a TTEnT mechanism involving sensitization of quinolone substrates.

Q4. The SI is well written and comprehensive. I have some reservations about the generalization method employed when assessing conversion and the interpretation of e.r.'s. In other comparable studies, calibration curves are included for every substrate. While the difference may be small, given that you are comparing yields and ee's within each substrate (at similar but not consistent concentrations), this may be worthwhile. I would also note that the separation of enantiomers with overlapping starting material signals is generally good, but in my view not acceptable for compounds 2F or 2L, and some further HPLC optimization needs to be conducted here.

Response: Following the reviewer's comment, we have now measured actual calibration curves for each substrate. This re-evaluation indeed led to noticeable differences in some cases, while the overall trends and conclusions of the study remain unchanged. We thank the reviewer for pointing out this important methodological aspect, which has strengthened the manuscript. The revised conversion values are now reported in the main text and Supporting Information.

Regarding the separation of enantiomers, additional HPLC optimization was carried out for compounds **2f** and **2l**, as requested by the reviewer. In addition, we extended this re-optimization to compounds **2d**, **2e, 2j, 2a'** and **2g'**. These improved separations resulted in minor adjustments to the initially reported enantiomeric excesses. Both the main manuscript and the Supporting Information have been updated accordingly. Here again, the overall trends and conclusions of the study remain unchanged.

Overall, I think this is a good piece of work and suitable for publication in Nature Communications upon minor revisions addressing the issues raised.

Response: We thank the referee once again for their overall positive evaluation of our work.

Reviewer #3:

This manuscript reports the design of an artificial photoDNAzyme created by covalently embedding an iridium (Ir) metal photocatalyst within a DNA double helix, which is applied to [2+2] photocycloaddition reactions, with a focus on controlling enantioselectivity. Although the authors' research is well-supported by experimental design and data, from the perspectives of innovation and academic value, it does not provide sufficient justification for publication in Nature Communications.

Response: We thank the reviewer for their careful evaluation of our work and for providing thoughtful comments. We appreciate the opportunity to clarify the novelty, significance, and conceptual advances of our study. Our responses to each point are provided below.

1. Limited Innovation： The primary novelty of this work lies in the incorporation of an Ir metal photocatalyst into DNA. However, Ir-based photocatalysts have already been widely used in photochemistry and asymmetric catalysis by research groups such as Yoon and Meggers. Therefore, while embedding the Ir photocatalyst in DNA is novel in form, the core innovation is limited.

Response: We respectfully disagree. Our work represents the first example of a highly enantioselective artificial photoDNAzyme, and more broadly, the first example of an enantioselective metallo-photoenzyme. While Ir-based photocatalysts are indeed well-established in small-molecule

photochemistry, their integration into a DNA scaffold to achieve asymmetric photochemical transformations is unprecedented. This combination merges transition-metal photochemistry and biopolymer-based asymmetric catalysis, leading to a new concept for designing chiral photocatalytic systems. Our approach thus opens a new direction in the emerging field of artificial metallo-photoenzymes, distinct from traditional small-molecule catalysis.

2. No Clear Advantage Over Existing Research: Ir photocatalysts have already been applied in [2+2] photocycloaddition reactions, and the artificial photoDNAzyme developed in this manuscript does not demonstrate significant advantages over existing systems in terms of substrate scope and catalytic efficiency. In particular, the enantioselectivity and reaction yields reported here do not surpass those from previous studies.

Response: We appreciate the reviewer's observation and agree that a number of Ir-based photocatalysts have been successfully applied to [2+2] photocycloaddition reactions with excellent efficiency and selectivity. However, our study is not intended as an incremental improvement of existing Ir-based catalytic systems, but rather as a conceptual breakthrough in the design of artificial metallo-photoenzymes. The present work establishes the first example of an enantioselective artificial metallo-photoenzyme, integrating a transition-metal photosensitizer into a DNA scaffold to achieve asymmetric photochemical transformations. Importantly, this system operates enantioselectively in aqueous medium, a capability that remains inaccessible to state-of-the-art iridium-based photocatalysts. Hence, while the catalytic efficiency may not yet surpass that of optimized small-molecule systems, our approach clearly surpasses previous efforts to develop protein-based artificial metallo-photoenzymes (see ref 24 & 25).

3. Asymmetric [2+2] photocycloaddition reactions have been widely studied and reported (e.g., J. Am. Chem. Soc. 2011, 133, 16689; Chem. Eur. J. 2013, 19, 7461; Angew. Chem. Int. Ed. 2014, 53, 4368; J. Am. Chem. Soc. 2017, 139, 17186), with excellent results in catalytic efficiency and enantioselectivity. The system described in this manuscript does not demonstrate a clear advantage and lacks the potential to lead the field in a new direction.

Response: We respectfully note that our study does not aim to compete directly with classical asymmetric [2+2] photocycloadditions. Rather, it introduces a new catalytic paradigm that of artificial photoenzymes based on DNA scaffolds. The novelty lies not in the [2+2] reaction itself, but in achieving enantioselective photoinduced transformations within a DNA-based chiral environment. This represents a distinct research direction that differs fundamentally from conventional asymmetric TM-based catalysis and/or organocatalysis.

4. The authors emphasize that current protein-based photoenzyme systems cannot achieve dual enantioselectivity control, while their approach can achieve controlled enantiomer generation. However, dual enantioselectivity control is easily achievable in small molecule catalysis systems (e.g., J. Am. Chem. Soc. 2022, 144, 4206), making the advantage of this catalyst less significant.

Response: We believe this comment reflects a misunderstanding of the term "dual enantioselectivity control" as used in our manuscript. While our system can indeed display cooperative effects similar to those described by Yoon and co-workers (JACS 2022), the expression was originally intended to refer to the ability to selectively obtain either enantiomer of the photoproduct using mirror-image artificial photoDNAzymes, which remains a current limitation of protein-based artificial photoenzymes. To avoid any ambiguity, we have decided to remove the expression "dual enantioselectivity control" from the revised version of the manuscript.

We also thank the reviewer for drawing our attention to the work of Yoon and co-workers on cooperative stereoinduction in asymmetric dual photocatalysis (J. Am. Chem. Soc. 2022, 144, 4206), which we now cite when discussing match/mismatch effects observed with diastereomeric photoDNAzymes (ref 35).

While this manuscript presents some exploratory experimental designs and ideas, it falls short in terms of innovation, the advantages of the proposed research system, and its contribution to the existing literature. Therefore, I do not believe the article is suitable for publication in Nature Communications at this stage. I suggest the authors consider submitting the manuscript to a more specialized journal that is better suited to this field.

Response: We respectfully disagree. The concept of an artificial photoDNAzyme constitutes a significant interdisciplinary advance that unites DNA catalysis, photochemistry, and asymmetric synthesis. The demonstration of enantioselective photocycloadditions catalyzed by a DNA–Ir hybrid catalyst establishes a new platform for metallo-photoenzyme design, expanding the conceptual boundaries of artificial enzyme research. We believe this innovation aligns well with the broad interdisciplinary scope and impact expected of *Nature Communications*.
